# Enzymatic Cascades for Stereoselective and Regioselective Amide Bond Assembly

**DOI:** 10.1002/anie.202422185

**Published:** 2025-01-21

**Authors:** Daniele Torri, Luis Bering, Luke R. L. Yates, Stuart M. Angiolini, Guangcai Xu, Sebastian Cuesta‐Hoyos, Sarah A. Shepherd, Jason Micklefield

**Affiliations:** ^1^ Department of Chemistry and Manchester Institute of Biotechnology The University of Manchester Manchester M1 7DN UK; ^2^ Department of Chemistry, Molecular Science Research Hub Imperial College London W12 0BZ UK

**Keywords:** biocatalysis, green chemistry, amides, ligases, cascades

## Abstract

Amide bond formation is fundamental in nature and is widely used in the synthesis of pharmaceuticals and other valuable products. Current methods for amide synthesis are often step and atom inefficient, requiring the use of protecting groups, deleterious reagents and organic solvents that create significant waste. The development of cleaner and more efficient catalytic methods for amide synthesis remains an urgent unmet need. Herein, we present novel biocatalytic cascade reactions for synthesising various amides under mild aqueous conditions from readily available organic nitriles combining nitrile hydrolysing enzymes and amide bond synthetase enzymes. These cooperative biocatalytic cascades enable kinetic resolution of racemic nitriles and provide a highly enantioselective biocatalytic extension of the Strecker reaction. The regioselective non‐directed C−H bond amidation of simple arenes was demonstrated through the incorporation of photoredox catalysis to the front end of the cascade. C−H bond amidation of simple aromatic precursors was also achieved via a CO_2_ fixation cascade combining enzymatic carboxylation and amide bond synthesis in one‐pot.

## Graphical abstract

Amide bond synthetase (ABS) enzymes catalyse amide formation in an environmentally friendly manner. This study advances the application of ABS by integrating these enzymes with other compatible biocatalysts, creating enantioselective and scalable cascade reactions to produce valuable amide products from abundant nitrile precursors. Furthermore, two innovative methods for C−H bond amidation of aromatic compounds are introduced.

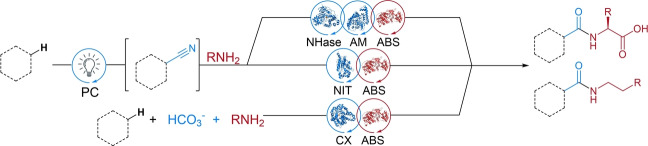

## Introduction

Due to their exquisite selectivity, evolvability and mild aqueous operating conditions, enzymes can provide attractive routes to pharmaceuticals and other molecules that would otherwise necessitate polluting chemical synthesis.[^1^, ^2^, ^3^, ^4^, ^5^, ^6^] The advantages of biocatalytic processes are not only limited to the activity of single enzymes but can be broadened by using multiple enzymes in a single process. Combining enzymes from different origins, can lead to new cascade reactions, and products, not present in nature's biochemical pathways.[^7^, ^8^, ^9^] These artificial biocatalytic cascades are particularly advantageous as the synergistic activity of multiple enzymes remove the need to work‐up and isolate potentially unstable intermediates.[^7^, ^8^, ^9^] The synthetic utility of enzymes can be further expanded through integration with chemocatalysis, resulting in processes that combine the advantages of enzymatic transformation with the generality of chemical reactions.[^10^, ^11^]

The acylation of amines to form amides is a widely used synthetic transformation.[^12^, ^13^] Many drug candidates contain at least one amide bond and amide bond formation is reported to be the most widely used reaction in medicinal chemistry.[^12^, ^13^] Despite the fundamental and practical importance of amides, the synthesis of amide bonds is still inefficient and expensive.[^14^, ^15^, ^16^] Current methods for amide synthesis require protection groups and rely on toxic coupling reagents and solvents, leading to waste and purification difficulties.[^17^, ^18^] The need for improved and sustainable methods for catalytic amide bond formation has resulted in the development of various biocatalytic approaches for amide synthesis.[^19^, ^20^, ^21^, ^22^, ^23^, ^24^, ^25^, ^26^, ^27^, ^28^] For example, we recently discovered and characterized various amide bond synthetase and ATP‐Grasp enzymes, which activate carboxylic acid (donors) with ATP and couple amine (acceptor) substrates to produce amides including important pharmaceutical intermediates and products.[^27^, ^28^] Moreover, the necessity for high concentrations of expensive ATP in ligase catalysed amidation can be mitigated using enzymatic ATP‐recycling strategies, further improving the green credentials of such transformations. Distinct enzyme pairings have been successfully implemented to regenerate ATP for ABS catalysed amide formation, in work published by our group and an independent laboratory group respectively.[^27^, ^29^]

**Figure 1:**
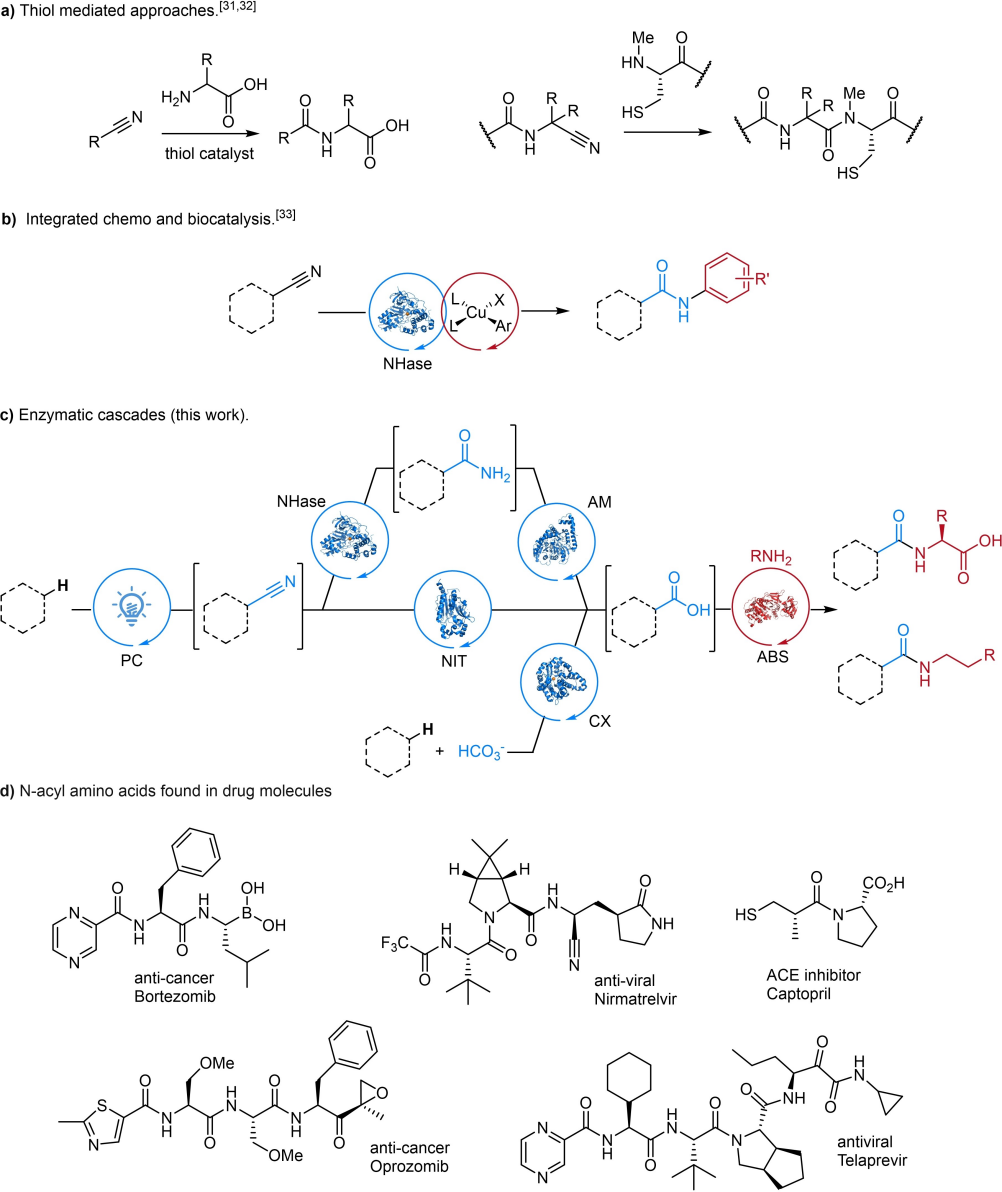
(a) Thiol catalysed pre‐biotic conversion of nitrile to amide and peptide chemical ligation using nitrile and thiols. (b) Integrated catalysis approach combining nitrile hydratase and copper catalysis for the synthesis of amides. (c) New enzymatic cascades towards amides synthesis in this work. (d) Examples of N‐acyl amino acid motifs in pharmaceuticals. Legend: NHase=nitrile hydratase, AM=amidase, ABS=amide bond synthetase, NIT=nitrilase, CX=(de)carboxylase.

Although carboxylic acids are common precursors for amide synthesis, their polarity and reactivity often necessitate the use of protecting group strategies. Utilising nitriles for amide synthesis is an attractive alternative as they are widely available and unreactive under typical amide coupling conditions.^[15]^ The conversion of nitriles to amides, beyond hydration to primary amides, is however limited in scope and requires harsh reaction conditions or transition‐metal catalysts.^[30]^ Recently, methodology was developed for the ligation of peptide fragments possessing C‐terminal nitrile functionality and N‐terminal cysteine residues (Figure 1a).^[31]^ Prebiotic chemistry studies also show how nitriles can be coupled with amines employing thiols as catalysts to produce peptides in water.^[32]^ Our laboratory also developed an integrated multi‐catalytic approach in which bacterial nitrile hydratase enzymes (NHase) and a transition‐metal catalyst (Cu) were merged in the same reaction vessel to convert nitriles into corresponding amides (Figure 1b).^[33]^ Motivated by this, we sought to establish a fully biocatalytic approach utilising nitriles for amide bond synthesis avoiding the requirement for any transition‐metal catalyst, which can be expensive and difficult to remove to the low levels required for pharmaceutical applications.^[34]^ Herein we report a biocatalytic cascade for the direct conversion of organic nitriles into amide products by combining nitrile hydrolysing enzymes with amide bond synthetase enzymes.^[27]^ (Figure 1c). We demonstrate highly stereoselective kinetic resolution (KR) of racemic nitrile donor and α‐aminonitrile (Strecker‐type) acceptor substrates. These cascades provide a very direct and highly stereoselective route to N‐acyl amino acids that are found in many drugs (Figure 1d). Regioselective formal C−H bond amidation of simple aromatic substrates was also achieved using a photochemical C−H bond cyanation to provide the nitrile precursors, or using a (de)carboxylase enzyme to fix CO_2_ for subsequent amide bond formation.

## Results and Discussion

### Biocatalytic Cascade Development

**Figure 2:**
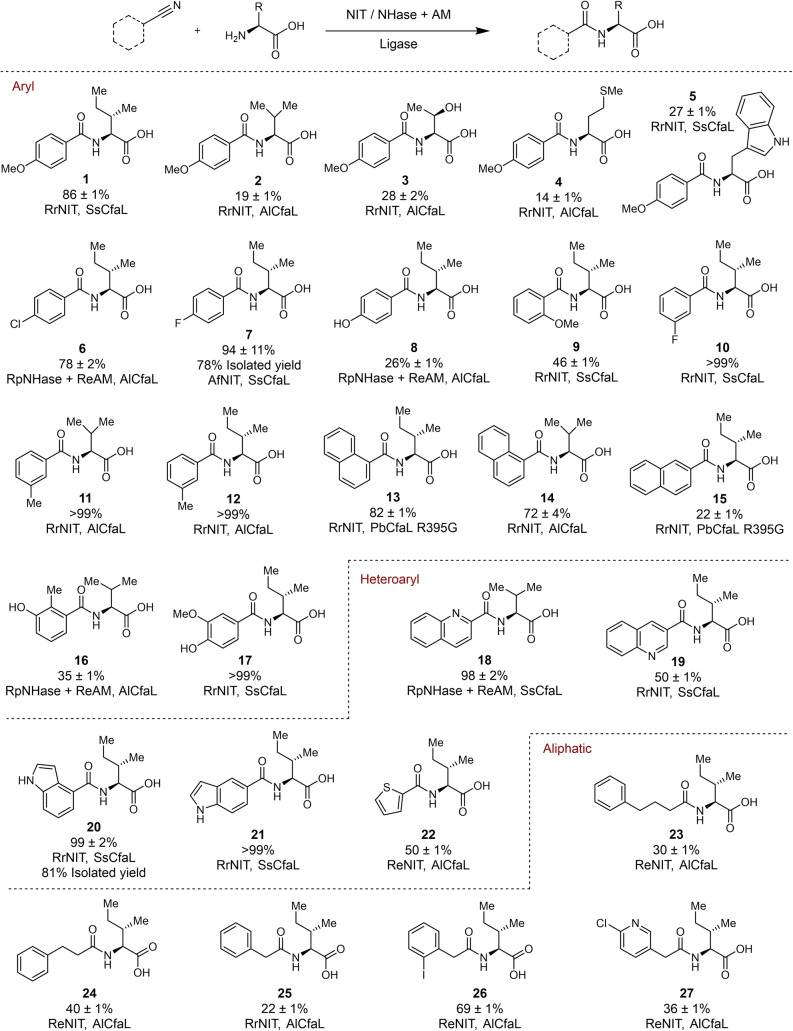
Scope for the integrated biocatalytic cascade for the synthesis of amide from nitriles. Reaction conditions: Nitrile (2 mM), NIT lysate (2 mg/mL) or NHase (whole cells OD600 of 0.5) and AM (whole cells OD600 of 1.3), CfaL (25 μM), l‐amino acid (10 mM), ATP (10 mM), MgCl_2_ (10 mM), HEPES buffer (100 mM, pH=8)/DMSO (5 % v/v), 30 °C, 18 h. Experiments were done in triplicate with yields determined by HPLC with comparison to synthetic standards. Isolated yields were calculated based on the mass of products obtained after purification by column chromatography. Legend: NIT=Nitrilase; NHase=Nitrile hydratase; AM=Amidase.

Our initial objective was to devise an effective transformation of nitriles into amides through the sequential operation of nitrile hydrolysing enzymes^[35]^ and amide bond synthetase enzymes.^[27]^ To test this approach, we selected several nitrilase (NIT) that hydrolyse nitriles to carboxylic acids including *Rhodococcus rhodochrous* J1 (RrNIT),^[36]^ *Fusarium duplospermum* IMI (FdNIT),^[34]^ from *Rhodococcus erythropolis* AJ270 (ReNIT),^[37]^ from *Acidovorax facilis* (AfNIT)^[38]^ (Figure S1). We also explored combining the nitrile hydratase (NHase) from *Rhodopseudomonas palustris* (CGA009, RpNHase)^[33]^ which hydrates nitriles to form primary amides, and an amidase (AM) from *Rhodococcus erythropolis* (*Re*AM),[^39^, ^40^] which catalyse the subsequent hydrolysis of the primary amide to provide carboxylic acid intermediates (Figure S2). Three amide bond synthetase enzymes^[27]^ (AlCfaL from *Azospirillum sp*, SsCfaL from *Streptomyces scabiei*, and PbCfaL R395G from *Pectobacterium brasiliense*) were also produced (Figure S3), which are versatile biocatalysts for coupling a wide range of carboxylic acid (donor) and amino acid (acceptor) substrates to produce valuable amides.^[27]^ Initial screening was carried out with different combinations of NIT‐CfaL in the biocatalytic conversion of a model substrate, 4‐methoxybenzonitrile to the amide **1** via the intermediate carboxylic acids (Figures 2, S4 & S7) RrNIT and SsCfaL proved productive for the synthesis of **1** and further optimisation of this cascade reaction were performed by systematically exploring different enzyme, substrate, and ATP concentrations (Figure S4).

Having optimised conditions for the conversion of 4‐methoxybenzonitrile into amide **1** (86 %) using l‐Ile as the acceptor, we also showed that RrNIT and CfaL enzymes could convert this nitrile to other amide derivatives **2–5** with l‐form Val, Thr, Met and Trp as acceptor substrates in lower yields (14–28 %) (Figures 2 & S8–S11). The scope of NIT‐CfaL or NHase/AM‐CfaL cascades was tested with a range of other nitrile substrates and either l‐Ile or l‐Val which are both preferred acceptor substrates for CfaL (Figures S12–S33). Typically, we began screening the various substrates with the panel of NIT enzymes. When NITs exhibited limited conversion, we then explored the use of the NHase/AM system to establish cascades with superior performance. Most of the benzonitriles with small substituents afforded amides (**6**, **7**, **10–11**) in good‐excellent yields (46–99 %). 1‐Napthonitrile was also transformed to amides **13** and **14** in good yields (82 & 72 %) using RrNIT and either AlCfaL or PbCfaL R395G. Next, we explored the scope of the cascade reaction with heterocyclic nitrile substrates. We found that quinoline, indole and thiophene nitriles were well accepted providing amides (**18–22**). A few aliphatic nitrile substrates were shown to be transformed to amides **23–27** in 21–75 % yield, using ReNIT or RrNIT combined with AlCfaL. Finally, to demonstrate the scalability of the biocatalytic cascade, amides **7** and **20**, were synthesised on a preparative scale (0.1 mmol) with an isolated yield of 78 and 81 % respectively. The various nitrile hydrolysing enzymes (NIT, NHase and AM) are generally highly active and robust, allowing us to use either cell‐free lysates or whole cells, which are more practical for larger‐scale synthesis. We anticipated that further scale‐up of the cascade could also benefit from using established ATP regeneration systems for the ligase step.[^27^, ^29^] The overall scope of the cascade is largely governed by the donor‐acceptor selectivity of the CfaL enzymes, which are more closely related^[27]^ than the more diverse family NIT or NHase/AM deployed in this case. It should therefore be possible to expand the cascades to access a wide range of amide products by replacing CfaL with new and emerging amide bond synthetase enzymes.[^28^, ^41^, ^42^, ^43^]

### Kinetic Resolutions of Racemic Nitrile Donor Substrates

**Figure 3:**
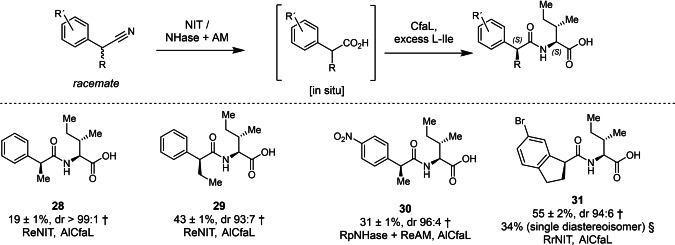
Scope for the integrated biocatalytic cascade for the chiral resolution of racemic nitriles. Reaction conditions: Nitrile (2 mM), NIT lysate (2 mg/mL) or NHase (whole cells OD600 of 0.5) and AM (whole cells OD600 of 1.3), CfaL (25 μM), l‐Ile (10 mM), ATP (10 mM), MgCl_2_ (10 mM), HEPES buffer (100 mM, pH=8)/DMSO (5 % v/v), 30 °C, 18 h. † % conversions and diastereoisomeric ratios (d.r.) was determined in triplicate by HPLC/UV analysis. § Isolated yield of the single diastereoisomer **31** after purification by column chromatography. Legend: NIT=Nitrilase; NHase=Nitrile hydratase; AM=Amidase.

Previously, CfaL enzymes were shown to be very effective for kinetic resolution (KR) of racemic amino acid acceptor substrates, with very high selectivity for l‐amino acids. However, the KR of racemic carboxylic acid donor substrates, resulted in amide products with moderate stereoselectivity.^[27]^ Earlier studies also show NIT provide moderate to good enantioselectivity in the hydrolysis of racemic α‐substituted nitriles such as 2‐phenylpropanenitrile.[^44^, ^45^] RpNHase and ReAM, on the other hand, are both reported to have excellent enantioselectivity and prefer (*S*)‐enantiomer of 2‐phenylpropanenitrile and 2‐phenylpropionamide.[^39^, ^46^] In light of this, we sought to investigate if NIT‐ or NHase/AM‐CfaL cascades could be used for the more stereoselective synthesis of amide products, from readily available racemic nitrile substrates (Figures 3 & S34–S37). First, we explored the synthesis of amide **28** from racemic 2‐phenylpropanenitrile via 2‐phenylpropanoic acid with an excess of l‐Ile as the CfaL acceptor substrate. CfaL is selective for (*S*)‐2‐phenylpropanoic acid, and should be compatible with RpNHase and ReAM which are also *S*‐selective.[^39^, ^40^, ^46^] In addition, we checked the enantioselectivity of RrNIT and ReNIT with 2‐phenylpropanenitrile to establish if these enzymes also exhibit matched stereoselectivity. We found that ReNIT gave very good activity but almost no enantioselectivity, while with RrNIT gave moderate (*S*)‐enantioselectivity, but very poor activity with 2‐phenylpropanenitrile (Figure S5). Although ReNIT offers no enantioselectivity, when we combined with AlCfaL in a cascade reaction using the conditions deployed above (Figure 2), amide **28** was formed in a yield of 19 %, with a high diastereomeric ratio (d.r. >99 : 1), based on the racemic 2‐phenylpropanenitrile substrate. The same enzymes also delivered amide **29** (43 %, 93 : 7 d.r.), whilst the RpNHase, ReAM and AlCfaL three enzyme cascade proved most effective for the synthesis of amide **30** (31 %, 96 : 4 d.r.). The bicyclic amide **31** was also produced in good yield and with high diastereoselectivity using the RrNIT‐AlCfaL cascade. This cascade was also carried out on a preparative scale affording **31** as a single diastereomer in 34 % isolated yield. The excellent diastereoselectivities observed in these cascade reactions underlines the utility of combining enzymes, with matched stereoselectivity, to affect multiple sequential kinetic resolutions. Moreover, the use of nitrile precursors which are prone to racemisation at elevated pH and temperature may afford the opportunity for dynamic kinetic resolution (DKR) to increase the yields of homochiral amide products beyond the 50 % theoretical maximum yield for KR.[^47^, ^48^]

### Extended Strecker Cascades to Non‐Canonical N‐acyl‐l‐Amino Acid Derivates

**Figure 4:**
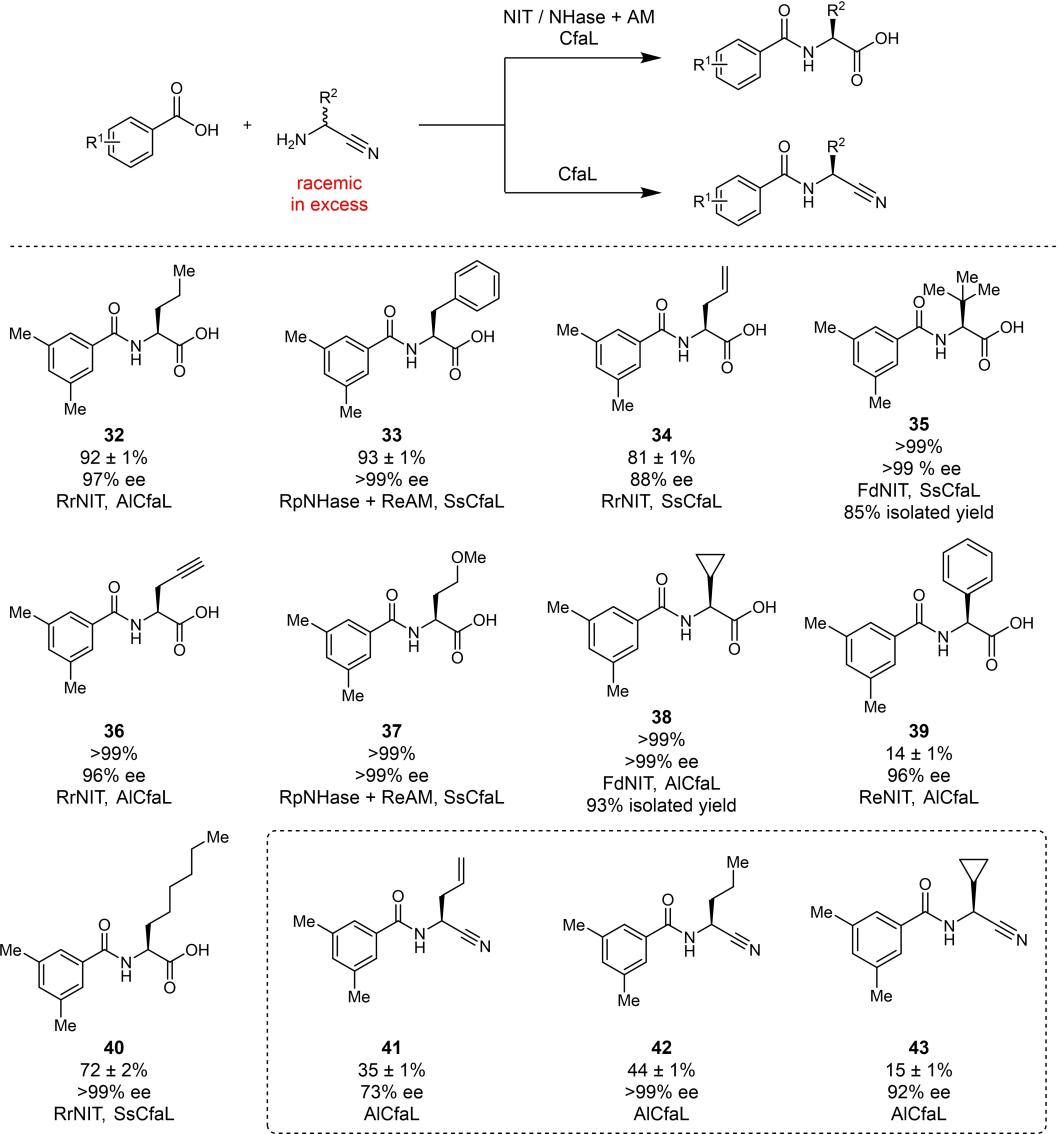
Scope for the integrated biocatalytic cascade for the chiral resolution of α‐amino nitriles in extended Strecker‐type reactions. Reaction conditions: Carboxylic acid (2 mM), NIT lysate (2 mg/mL) or NHase (whole cells OD600 of 0.5) and Amidase (whole cells OD600 of 1.3), CfaL (25 μM), α‐amino nitrile (10 mM), ATP (10 mM), MgCl_2_ (10 mM), HEPES buffer (100 mM, pH=8)/DMSO or MeOH (5 % v/v), 30 °C, 18 h. Conversion was determined in triplicate by HPLC/UV analysis (*n*=3) and presented as mean values±SD. Isolated yields refer to mass of products after purification of column chromatograph. Legend: NIT=Nitrilase; NHase=Nitrile hydratase; AM=Amidase.

We next sought to expand the biocatalytic cascade approach by exploiting the high enantioselectivity of CfaL for l‐ over d‐amino acids^[27]^ (Figure 4). The kinetic resolution of racemic amino acids, although useful, is limited by the fact that many enantiomerically pure amino acids are widely available. On the other hand, the resolution of racemic α‐aminonitrile substrates using NIT or NHase/AM and CfaL to produce N‐acylated derivatives of non‐canonical α‐amino acids, would be more attractive (Figure 4). The widely used Strecker synthesis involves the reaction of aldehydes with ammonia and cyanide, to form α‐aminonitrile intermediates, which are hydrolysed to produce racemic amino acids. Several asymmetric Strecker reactions have been developed but these typically require either the use of chiral auxiliaries which is atom inefficient, or expensive chemical catalysts.^[49]^ NIT have also been used to hydrolyse the α‐aminonitrile to amino acids.[^50^, ^51^] We anticipated that our cascade approach could be used to extend the widely used Strecker synthesis, using NIT or NHase/AM to affect KR of racemic α‐aminonitriles, with CfaL resulting in useful functionalised, or protected, N‐acyl‐l‐amino acid derivatives (Figures 4 & S38–S46). Cascade reactions with an excess of racemic α‐aminonitrile donor and 3,5‐dimethylbenzoic acid as a model acceptor substrate, proved successful, resulting in a wide range of amide products (**32**–**40**) in excellent yields (based on the acyl donor substrate) and high enantioselectivities. To leverage value from the cascades we focused on the synthesis of non‐proteinogenic N‐acyl‐l‐amino acid derivatives, which typically require more extensive chemical synthesis and include common building blocks of pharmaceuticals, particularly peptide therapeutic agents (Figure 1d). Having established the scope of the cascade on an analytical scale, two reactions were performed on a preparative scale to generate amides **35** and **38** in 85 % and 93 % yield with excellent e.e. (>99 %). Although in these examples, we use the same carboxylic acid donor substrate, our earlier studies with CfaL show that a wide range of aromatic and aliphatic carboxylic acid donors would be compatible with this ‘extended Strecker’ cascade (Figure 4).

During the HPLC analysis of some of the cascade reactions (Figure 4), we observed the emergence of a small amount of an intermediate product, which proved to be N‐acyl α‐aminonitrile derivatives. Given our previous studies showed CfaL are highly selective for l‐amino acid donor substrates, we expected that the cascade would proceed with α‐aminonitrile hydrolysis to an α‐amino acid intermediate, which would be N‐acylated by CfaL. We did not anticipate that CfaL could N‐acylate the α‐aminonitrile directly, and so we choose to explore this further by incubating the panel of α‐aminonitriles substrates and 3,5‐dimethylbenzoic acid with CfaL alone. Using AlCfaL α‐aminonitrile substrates with allyl‐, propyl‐ and cyclopropyl side chains resulted in amide products **41** (35 %, 73 % e.e.), **42** (44 %, >99 % e.e.), **43** (15 % yield, 92 % e.e.) (Figures 4 & S47–S49). The scope of CfaL reactions with α‐aminonitriles is more limited than with the native l‐amino acid substrates. Presumably replacing the negatively charged carboxylic acid of the native substrates, with a nitrile substituent is likely to reduce electrostatic interactions with CfaL active site. The absence of a carboxylate group may also account for the lower enantioselectivity observed for production of amides **41** and **43**. N‐acyl α‐aminonitrile products are also more prone to racemisation at higher pH than the corresponding N‐acyl α‐amino acids. Nevertheless, the nitrile group of the products (**41**–**43**) may serve as a versatile synthetic handle, for subsequent transformations (reduction, nucleophilic addition or cycloaddition reactions etc.),[^51^, ^52^] or thiol mediated ligations to generate peptides.[^31^, ^32^]

### Regioselective C−H Amidation using a Photocatalytic‐Enzyme Cascade

**Figure 5:**
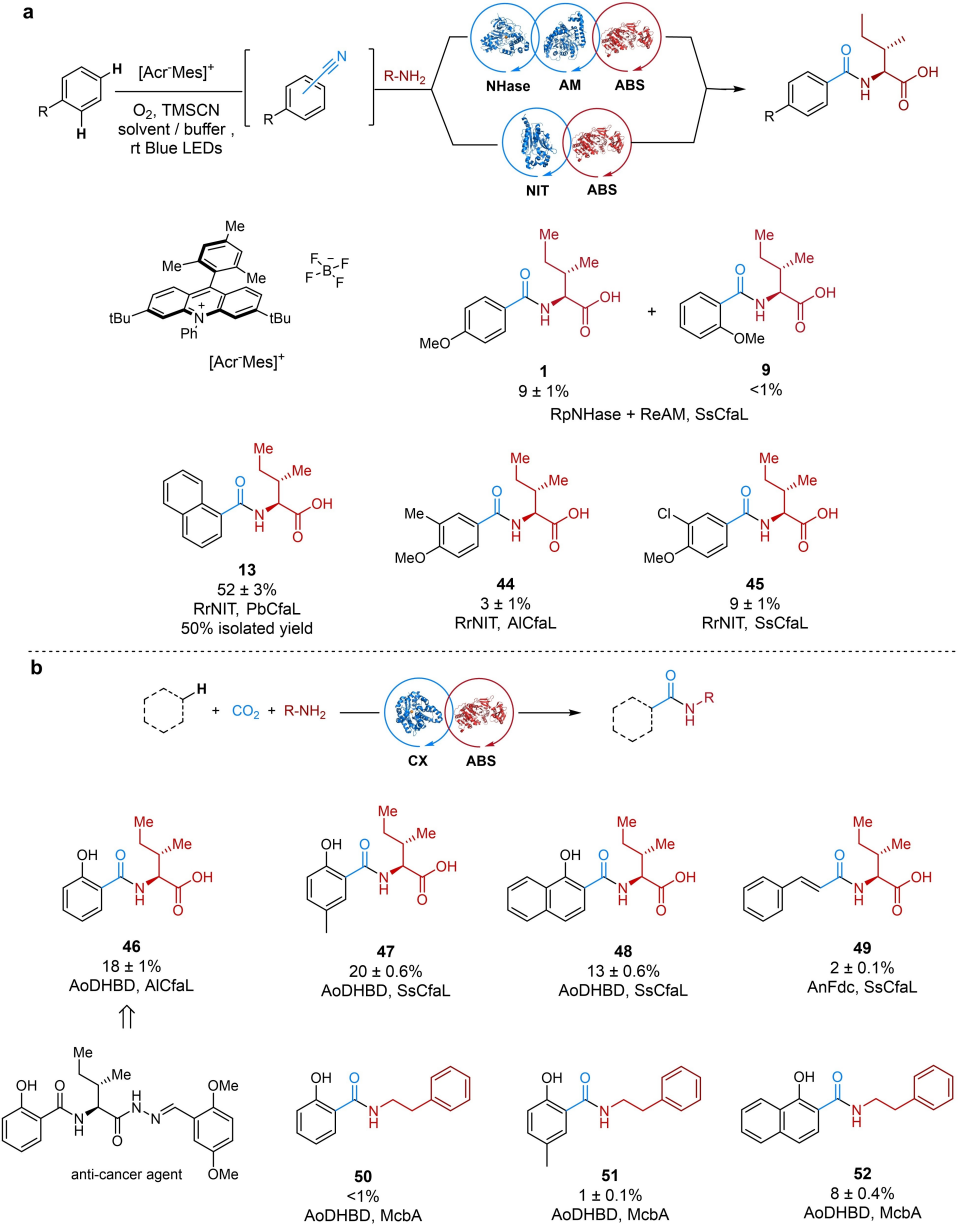
Scope for the regioselective integrated chemo‐ and biocatalytic C−H bond amidation. (a) C−H bond amidation using an integrated photocatalytic‐enzyme cascade. Cyanation reaction conditions: arene (100 mM) in 10: 1 MeCN/KPi buffer (4 M, pH=9), TMSCN (150 mM), blue light for 4–48 h. Amidation reaction condition: NIT lysate (2 mg/mL) or RpNHase (whole cells OD600 of 0.5) and ReAM (whole cells OD600 of 1.23, CfaL (25 μM), l‐Ile (10 mM), ATP (10 mM), MgCl_2_ (10 mM), HEPES buffer (100 mM, pH=8)/DMSO (5 % v/v), 30 °C, 18 h. (b) C−H bond amidation via CO_2_ fixation enzyme cascade. Carboxylation‐ligation reaction condition: arene or styrene (1 mM), l‐Ile or 2‐phenylethylamine (10 mM), ATP (10 mM), MgCl_2_ (10 mM), CfaL or McbA (50 μM), carboxylase (15 or 50 μM), KHCO_3_ (225 or 375 mM), Tris‐HCl buffer (60 mM, pH=8)/DMSO (2 % v/v), 20/30 °C, 1–5 d. The % conversion was determined in triplicate by HPLC/UV analysis (*n*=3) and presented as mean values±SD. Isolated yields refer to mass of products after purification by column chromatography. Legend: NHase=nitrile hydratase; AM=amidase; ABS=amide bond synthetase; NIT=nitrilase; CX=carboxylase.

The direct modification of non‐functionalised starting materials poses a considerable challenge, as non‐directed C−H bond functionalisation methodologies often exhibit suboptimal regioselectivity.^[53]^ We aimed to address this through a chemoenzymatic cascade employing photocatalytic C−H bond cyanation^[54]^ of non‐functionalised arenes to generate nitrile intermediates for further in situ enzymatic derivatisation. Photocatalytic cyanation of simple arenes typically produces a mixture of regioisomers, which can be difficult to separate, thereby limiting the method's utility.^[54]^ We anticipated that enzymatic hydrolysis of the less sterically hindered nitrile may be achievable if the nitrile converting enzymes exhibits adequate regioselectivity. The resulting carboxylic acid regioisomer could then serve as a substrate for CfaL (Figure 5a).

Initially, the C−H bond cyanation procedure^[54]^ was applied to simple naphthalene as a model substrate (Table S1). The established photocatalytic cyanation relies on the use of acetonitrile as solvent,^[54]^ which is unsuitable for the subsequent integration with NIT enzymes due to substrate competition and enzyme deactivation. As a result, other solvents were examined to identify a viable replacement for acetonitrile but all those resulted in a sharp decline in yields of 1‐naphthonitrile (Table S1). Further attempts to identify alternative photocatalysts (Ir, Rh, 4CzIBN) in various solvents did not produce naphthonitrile products. Likewise, adjustments to the stoichiometry of the reaction components failed to improve the yield significantly. A solvent swap (acetonitrile removal under reduced pressure and resuspension in DMSO), before addition of enzymes, was thus unavoidable. Nonetheless, given the relatively small volume of organic solvent used compared to the buffer volume, this approach was still perceived as a feasible strategy for incorporation into the subsequent biocatalytic cascade. Next, efforts were made to realise the full cascade reaction, transforming naphthalene to amide **13**, via 1‐naphthonitrile which we showed was a good substrate for RrNIT and 1‐naphthoic acid which is also accepted by PbCfaL R395G. Following optimisation, a one‐pot C−H bond amidation process was developed affording the amide **13** with a satisfactory 52 % yield from naphthalene (Figures 5a & S5**1**), which was also carried out on a preparative scale (50 % isolated yield). The only required intervention was a solvent exchange following the photocatalytic cyanation, prior to the addition of NITR, CfaL, reagents, and buffer.

Next, we explored the regioselective C−H bond amidation of anisole (Figures 5a & S52–S53). Although, the use of NHase and AM did afford *para*‐isomers **1** in high regioisomeric excess (10 : 1) the overall yield was low (9 %). The significantly improved regioselectivity (10 : 1) compared to photocatalysis alone (ranging from 1 : 1 to 3 : 1)^[54]^ is likely due to the regioselective nature of the enzyme. The C−H bond amidation of 2‐methylanisole and 2‐chloro also afforded amide products (**44** and **45**) in low yields. The less productive photocatalytic‐enzyme cascade to anisole amide derivatives is likely due to the inefficient photoredox catalysis, which affords only modest yields in the direct cyanation of anisoles (in the absence of enzymes).^[54]^ Nevertheless, the promising results with naphthalene, suggest that future development of improved photoredox catalysts, for aryl C−H bond cyanation, could lead to more useful chemobiocatalytic cascades for C−H bond amidation of arene scaffolds.

### Regioselective C−H Bond Amidation via CO_2_ Fixation

As an alternative to C−H bond cyanation we sought to explore if enzymatic carboxylation could be coupled with amide bond formation (Figure 5b). Although enzymatic conversion of abundant CO_2_ into useful chemical products is an attractive approach, there are few synthetically useful CO_2_‐fixing enzymes.^[55]^ To address this, a few (de)carboxylase enzymes have been deployed in the reverse direction, using high concentration of bicarbonate buffers to generate carboxylic acids. Coupling decarboxylase with down‐stream enzymes, that process the carboxylic acid intermediates, can also overcome the unfavourable equilibria associated with the carboxylation process.[^56^, ^57^]

We sought to explore if (de)carboxylase enzymes could be coupled with amide bond synthetase for C−H bond amidation (Figure 5b). The ferulic acid decarboxylase (AnFdc)^[56]^ from *Aspergillus niger* and 2,3‐dihydroxybenzoic acid decarboxylase (AoDHBD)[^58^, ^59^] from *Aspergillus oryzae* were selected based on their potential synthetic utility operating as carboxylases. AnFdc carboxylates styrene generating cinnamic acid, whilst AoDHBD can ortho‐carboxylate several phenolic substrates.[^56^, ^58^, ^59^] The carboxylic acids products of AnFdc and AoDHBD were screened with various CfaL enzymes, as well as with McbA another ABS which prefers phenylethyl amine as an acceptor substrate (Figure S6). Having identified potentially compatible carboxylase‐ABS pairings, conditions were optimised for AoDHBD‐CfaL cascades. Following an extended incubation (5 days) phenol, CO_2_ and l‐Ile were coupled together by AoDHBD‐AlCfaL to give amide product **46** in 18 % yield, with aqueous HCO_3_^−^ serving as the CO_2_ precursor (Figure 5b & S54–S60). Similar phenolic amides (**47** & **48**) were also produced using AoDHBD‐CfaL, with comparable yields of 20 % and 13 % respectively. Finally, cascade reactions with AnFdc‐CfaL or AoDHBD‐McbA were caried out which did produce amides (**49**–**52**), albeit in very low yields.

The limited scope and yields of carboxylase‐ABS cascades are likely due to the inherently low catalytic efficiency of the ligases on the carboxylic acid intermediates, which is insufficient to pull the equilibrium of the thermodynamically unfavourable carboxylation reaction. Expanding the pool of enzymes, including engineered and optimised variants, could provide more synthetically useful processes. For example, the acyl hydrazine derivatives of amide **46** (Figure 5b) was shown to possess anti‐cancer activity.^[60]^ Synthesis of amides (e.g. **46**) from cheap and abundant precursors (phenol, styrene, CO_2_/KHCO_3_, amino acids or amines) in a one‐pot biocatalytic cascade reaction, may therefore offer cleaner routes to pharmaceuticals and other useful products. Traditional methods for the carboxylation of phenols, such as the Kolbe‐Schmitt reaction, relies on high‐temperatures, pressures and acidic conditions that would not be compatible with enzymes.^[61]^ More recent chemocatalytic carboxylation reactions, operate under milder conditions, but use organic solvents, transition metals or other expensive catalysts.[^62^, ^63^, ^64^]

## Conclusion

In summary, we have developed biocatalytic cascades combining nitrilase (NIT), or nitrile hydratase (NHase) and amidase (AM), with amide bond synthetase enzymes (ABS) for the direct (one‐pot) conversion of organic nitriles into amide products. The cooperative activity of nitrile degrading enzymes with ABS provides various amides in high yields and excellent stereoselectivity from widely available racemic nitrile precursors under aqueous conditions using purified enzymes, cell free lysates or whole cell biocatalysts. The transformation of common racemic α‐aminonitriles into homochiral N‐acylated non‐canonical amino acids, via a one pot biocatalytic cascade delivers a useful and previously unexplored extension of the versatile Strecker synthesis. Interestingly, we discovered that CfaL, which acylates amino acids in nature, can also catalyse the unanticipated enantioselective acylation of *α*‐amino nitriles which are useful motifs present in drugs such as nirmatrelivir^[65]^ recently approved to treat COVID (Figure 1d). N‐acyl amino nitriles are also useful intermediates that can be subjected to a wide range of nitrile functional interconversions,[^51^, ^52^] or further extended to peptide products under thiol catalysis or via potentially prebiotic cystine mediated ligation chemistry.[^31^, ^32^] We show that photochemical cyanation of arenes can provide the nitrile precursors for artificial cascades, offering formal C−H bond amidation without isolation of nitrile or carboxylic acids intermediates. Finally, we demonstrate how cascade reaction combining (de)carboxylase and ABS enzymes can be similarly used for C−H bond amidation, coupling inexpensive and abundant precursors (e.g. phenols, CO_2_/KHCO_3_ and amino acids) to deliver amides including potential pharmaceutical precursors.

## Supporting Information

Additional experimental details can be found in the Supporting Information. The authors have cited additional references within the Supporting Information.

## Author Contributions

L.B and J.M. conceptualized the study. D.T., L.B., L.Y., S.A., G.X., S.C.H. performed the experimental investigation. J.M. acquired funding. J.M. and S.S. provided project administration. J.M. and S.S. provided supervision. D. T., L.B. and J.M. wrote the paper.

## Conflict of Interests

The authors declare no conflict of interest

1

## Supporting information

As a service to our authors and readers, this journal provides supporting information supplied by the authors. Such materials are peer reviewed and may be re‐organized for online delivery, but are not copy‐edited or typeset. Technical support issues arising from supporting information (other than missing files) should be addressed to the authors.

Supporting Information

Supporting Information

## Data Availability

The data that support the findings of this study are available from the corresponding author upon reasonable request.
