# Supplementary material for: Enzymatic Cascades for Stereoselective and Regioselective Amide Bond Assembly
Source: Angew Chem Int Ed Engl. 2025 Jan 21;64(13):e202422185. doi: 10.1002/anie.202422185 (PMC11933536; doi:10.1002/anie.202422185)
Supplement: Supplementary file 1 — Supporting Information [file ANIE-64-e202422185-s001.pdf]

Angewandte Chemie
Eine Zeitschrift der Gesellschaft Deutscher Chemiker

Supporting Information

Enzymatic Cascades for Stereoselective and Regioselective Amide Bond Assembly

*D. Torri, L. Bering, L. R. L. Yates, S. M. Angiolini, G. Xu, S. Cuesta-Hoyos, S. A. Shepherd, J. Micklefield**

Supporting Information for

Enzymatic Cascades for Stereoselective and Regioselective Amide Bond Assembly

Daniele Torri,[1†] Luis Bering,[1†] Luke R. L. Yates,[1] Stuart M. Angiolini,[1] Guangcai Xu,[1] Sebastian Cuesta-Hoyos,[1] Sarah A. Shepherd[1] & Jason Micklefield[1,2*]

†These authors contributed equally

Affiliation:

[1] Department of Chemistry and Manchester Institute of Biotechnology, The University of Manchester, 131 Princess Street, Manchester M1 7DN, UK.

[2] Department of Chemistry, Molecular Science Research Hub, Imperial College, London W12 0BZ, UK.

*Corresponding authors Email: Jason.micklefield@manchester.ac.uk or j.micklefield@imperial.ac.uk

Table of Contents

Supplementary methods 3

Materials and general methods 3

Cloning, expression, and protein purification 3

Nitrilase cell-free lysate preparation 4

General methods for enzymatic sascade reactions 5

HPLC methods 7

Supplementary figures and tables 8

Preparative scale enzyme cascades 14

Preparation of starting materials 18

Preparation of amide synthetic standards 19

Experimental data of starting materials 21

Experimental data of amide synthetic standards 24

HPLC traces and quantification of enzymatic cascade reactions 48

NMR spectra of compounds synthesised enzymatically. 102

NMR spectra of chemically synthesised standards and starting material. 109

Supplementary reference 170

Supplementary methods

Materials and general methods

Chemicals were purchased from Sigma-Aldrich, Acros Organics, Fluorochem, Fisher Scientific UK or Alfa Aesar and used without further purification unless otherwise stated. Solvents for chromatography were laboratory grade. Analytical thin-layer chromatography (TLC) was performed on Merck silica gel aluminium plates with F-254 indicator, visualized by irradiation with UV light, $KMnO_4$ or ninhydrin staining solution. Column chromatography was performed using silica gel Merck 60 (particle size 0.040 - 0.063 mm, 60 Å pore size). Chemical yields refer to isolated substances after column chromatography. ^{1}H and ^{13}C NMR spectra were recorded at 298K unless otherwise stated and are reported relative to residual solvent peaks. ^{1}H-NMR and ^{13}C-NMR were recorded on either a Bruker Avance I (400 MHz) or a Bruker Avance (500 MHz) spectrometer in $CDCl_3$ or DMSO-d_6. Unless otherwise stated, spectra are reported relative to residual solvent using iconNMR and TopSpin v3.5pl7. NMR data was processed using MestReNova v11 software. Data are reported in the following order: chemical shift (δ) in ppm; multiplicities are indicated s (singlet), d (doublet), t (triplet), q (quartet), m (multiplet); coupling constants (J) are given in Hertz (Hz). High resolution mass spectra were recorded on Agilent 6560 IM Q-TOF LC-MS and all mass spectrometry data were processed using Mass Hunter Workstation Software Qualitative Analysis B.06.00. HPLC traces were recorded on a Shimadzu NexeraXR UPLC and analysed with Shimadzu LabSolutions Lite v5. Cell suspension OD_{600} values were determined on an Agilent Technologies Cary 60 UV-Vis spectrometer using Cary WinUV Simple Reads Application software v5.0.0.999. Blue LEDs used for C–H bond cyanation were 2x Kessil PR 160 440 nm lamps. All C–H bond cyanation reactions were conducted in clear glass screw cap vials, which were placed approximately 8 cm from blue LEDs. Temperature was maintained using a household fan, mounted on top of the reaction vials.

Cloning, expression, and protein purification

Unless otherwise stated, synthetic genes were supplied by Twist Bioscience or GeneWiz as pET28a (+) constructs with the gene of interest cloned between NdeI and XhoI. Plasmids were used to transform *E. coli* BL21 (DE3) cells following standard procedures.[1] The synthetic DNA and translated protein sequences can be found in Supplementary Data 1. SDS-PAGE

analysis showed that all proteins were obtained in high purity and reasonable yield (Figure S 1-S3). Nitrilases (NIT) were cloned and expressed using the method reported previously and used as cell-free lysates.[2] Nitrile hydratase (NHase) RpNHase and Amidase (AM) ReAM were expressed using the method reported previously and used as wet whole cells.[3] CfaL and McbA ligases were expressed and purified using the method reported previously.[4] AnFdc gene was cloned in pETDuet-1 vector using the BamHI and HindIII site at multiple cloning site (MCS)-1. UbiX gene was cloned into the same vector for prenylated flavin cofactor maturation using NdeI and XhoI site at MCS-2. The expression and purification of AnFdc was performed according to procedure reported previously.[5] AoDHBD was cloned in pET-21a(+) vector using NdeI and XhoI site carrying a C-terminal His-tag. The expression and purification of AoDHBD followed the procedure reported previously.[6]

Nitrilase cell-free lysate preparation

A single colony from freshly transformed *E. coli* BL21 (DE3), or cells from -80 °C glycerol stock, were used to inoculate 10 mL of LB medium containing 50 µg/mL of kanamycin. The seed culture was incubated overnight at 37 °C, with 180 rpm shaking. The next day the seed culture was diluted 1:100 into 400 mL or 800 mL of autoinduction 2YT media supplemented with 50 µg/mL of kanamycin and incubated at 37 °C for 4.5 h, with 180 rpm shaking. The temperature was then reduced to 20 °C and the incubation continued for a further 16 h, with 180 rpm shaking. Following this, the cultures were harvested by centrifugation (3000 x g, 4 °C, 10 min). Cells were washed with PBS buffer (100 mL) and pelleted by centrifugation (3000 x g, 4 °C, 10 min) and stored at -20 °C. The next day, cell pellets were thawed, resuspended in KPi buffer (100 mM, pH = 7.8) and sonicated (10 min, 50% pulse, 50% amplitude). The supernatant was cleared from cell debris by centrifugation (23224 rcf, 4 °C, 30 min), transferred into a new tube and frozen in liquid nitrogen. Frozen pellets were freeze dried until they became a fine pale powder and stored at -20 °C until use. In the enzymatic assay, cell-free lysates were prepared as a stock solution at a concentration of 20 mg/mL and used at a final concentration of 2 mg/mL.

General methods for enzymatic sascade reactions

General method 1: Amide synthesis using NIT or NHase / AM combined with CfaL ligases (Fig. 2 & 3, compounds 1-31). To a solution of NIT cell-free lysate (2 mg/mL) or RpNHase (OD_{600}: 0.5) and ReAM (OD_{600}: 1.3) whole cells, in HEPES buffer (100 mM, pH = 8) / DMSO (5% v/v), were successively added nitrile (2 mM), amino acid (10 mM), CfaL ligase (25 µM), $MgCl_2$ (10 mM) and ATP (10 mM) to a final volume of 100 µL and the reaction was incubated at 30 °C for 24 h with 180 rpm shaking. After that time, the reaction was quenched with MeCN (100 µL), protein aggregates were removed by centrifugation (4000 rpm, 4 °C, 15 Min) and 100 µL of the supernatant was acidified with 20 µL TFA (1 M) before being subjected to HPLC analysis. All experiments on analytical scale were conducted in triplicate.

General method 2: Kinetic resolution of α-aminonitriles to produce *N*-acyl-L-amino acids or *N*-acyl-α-aminonitriles (Fig. 4, compounds 32-43). To a solution of NIT cell-free lysate (2 mg/mL) or RpNHase (OD_{600}: 0.5) and ReAM (OD_{600}: 1.3) whole cells, in HEPES buffer (100 mM, pH = 8) / DMSO (5% v/v), were successively added carboxylic acid (2 mM), α-aminonitriles (10 mM), CfaL ligase (25 µM), $MgCl_2$ (10 mM) and ATP (10 mM) to a final volume of 100 µL and the reaction was incubated at 30 °C for 24 h with 180 rpm shaking. After that time, the reaction was quenched with MeCN (100 µL), protein aggregates were removed by centrifugation (4000 rpm, 4 °C, 15 Min) and 100 µL of the supernatant was acidified with 20 µL TFA (1 M) before being subjected to HPLC analysis on normal and chiral stationary phase. All experiments on analytical scale were conducted in triplicate. The synthesis of *N*-acyl-α-aminonitriles was performed using the same method without the addition of NIT or NHase and AM.

General method 3: Regioselective C–H bond amidation of arenes via integrated photoredox catalysis/biocatalysis (Fig. 5a, compounds 1, 9, 13, 44 & 45). Step 1) To a solution of arene (100 mM) in 10: 1 MeCN / KPi buffer (4 M, pH = 9) was added 9-mesityl-3,6-di-tert-butyl-10-phenylacridinium tetrafluoroborate (5 mM) and the reaction was sparged with oxygen gas for 5 minutes. TMSCN (150 mM) was added, and the reaction was irradiated with blue LEDs for 24-48 h. After that time, the solvent was removed under reduced pressure and the residue containing crude nitrile was taken up in DMSO (40 mM final concentration, based on maximum theoretical conversion). Step 2) To a solution of NIT cell-free lysate (2mg/mL) or the combination of NHase (OD_{600} = 0.5) / amidase (OD_{600} = 1.3) whole cells in

HEPES buffer (100 mM, pH = 8) / DMSO (5% v/v), were successively added the crude nitrile (2 mM), L-Ile (10 mM), CfaL ligase (25 µM), $MgCl_2$ (10 mM) and ATP (10 mM) to a final volume of 100 µL and the reaction was incubated at 30 °C for 24 h with 180 rpm shaking. After that time, the reaction was quenched with MeCN (100 µL), protein aggregates were removed by centrifugation (4000 rpm, 4 °C, 15 Min) and 100 µL of the supernatant was acidified with 20 µL TFA (1 M) before being subjected to HPLC analysis. All experiments on analytical scale were conducted in triplicate.

General method 4: C-H bond amidation via CO_2 fixation (Fig. 5b, compounds 46-52). 100 µL aqueous solutions containing phenolic or styrene substrate (1 mM), L-Ile or 2-phenylethylamine (10 mM), ATP (10 mM), $MgCl_2$ (10 mM), CfaL or McbA (50 µM), (de)carboxylase (15 µM) in $KHCO_3$ (375 mM) with Tris-HCl (60 mM) (a stock solution containing $KHCO_3$ and Tris-HCl was prepared and adjusted to pH 8.0 before being used in the biocatalytic reactions). The reactions were then incubated at 20 °C for 5 days with shaking (700 rpm), prior to being quenched via the addition of one volume of MeCN. The quenched reactions were cleared via centrifugation prior to the supernatant being taken analysis via HPLC method D. Conversions were calculated by comparison to a calibration curve of peak areas from synthetic standards of each product. For compounds **47** & **51**, 50 µM of AoDHBD was used. For compounds **50**-**52**, 225 mM of $KHCO_3$ was used. For compounds **49**-**52**, 2% (v/v) DMSO was used as co-solvent. For compound **49**, the reaction was scaled up to 300 µL (in amber glass vials) and was run at 30 °C for 1 day.

HPLC methods

HPLC method A. Water and acetonitrile were used as mobile phase A and B, respectively, both supplemented with 0.05% TFA. The elution gradient was as follows: 0-6.5 min 5-95% B; 6.5-7.3 min 95% B; 7.3-7.5 min 95-5% B; 7.5-10 min 5% B. The column chamber was set at 40 °C, flow rate was set to 1.5 mL/min and injection volume was 10 µL. The HPLC system was fitted with a Kinetex 2.6 µm XB-C18 (100 Å, 100 x 4.6 mm) column.

HPLC method B. Water and acetonitrile were used as mobile phase A and B, respectively, both supplemented with 0.05% TFA. The elution gradient was as follows: 0-6.5 min 5-95% B; 6.5-7.3 min 95% B; 7.3-7.5 min 95-5% B; 7.5-10 min 5% B. The column chamber was set at 40 °C, flow rate was set to 1.5 mL/min and injection volume was 10 µL. The HPLC system was fitted with a Kinetex 2.6 µm EVO-C18 (100 Å, 50 x 3 mm) column.

HPLC method C. The enantiomeric excess was determined by chiral HPLC on a Shimadzu NexeraXR UPLC and analysed with Shimadzu LabSolutions Lite v5. Water and acetonitrile were used as mobile phase A and B, respectively, both supplemented with 0.1% FA. The elution was in isocratic mode at 40% B unless otherwise stated. The column chamber was set at 40 °C, flow rate was set to 1 mL/min and injection volume was 10 µL. The system was fitted with Lux® 5 µm i-Cellulose-5 LC column 250 x 4.6 mm.

HPLC method D. Water and acetonitrile were used as mobile phase A and B, respectively, both supplemented with 0.05% TFA. The elution gradient was as follows: 0-2 min 5% B; 2-10.5 min 5-95% B; 10.5-13 min 95% B; 13-13.5 min 95-5% B; 13.5-15 min 5% B. The column chamber was set at 40 °C, flow rate was set to 1.5 mL/min and injection volume was 15 µL. The HPLC system was fitted with a Kinetex 5 µm XB-C18 (100 Å, 50 x 4.6 mm) column.

Supplementary figures and tables

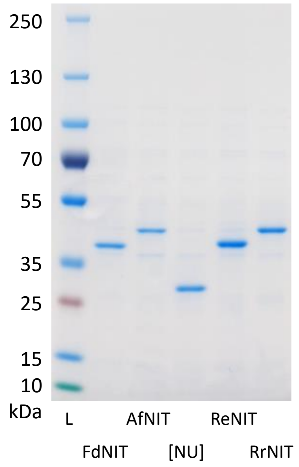

Figure S1. SDS-PAGE analysis of *E. coli* whole cells expressing the nitrilases used in this study. L = Protein ladder. Expected size: FdNIT (*F. duplospermum*): 37.3 kDa; AfNIT (*A. facilis*): 43.1 kDa; ReNIT (*R. erythropolis*): 38.9 kDa; RrNIT (*R. rhodochrous*): 42.0 kDa. [NU] represents another nitrilase that was Not Used in this study.

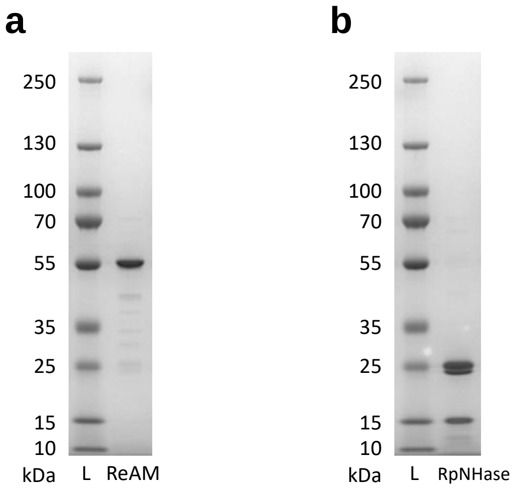

Figure S2. SDS-PAGE analysis of the amidase and nitrile hydratase used in this study. a, *E. coli* cell-free lysate from a culture overexpressing ReAM amidase. **b,** Ni-NTA purified RpNHase nitrile hydratase. L = protein ladder. Expected size: ReAM (*R. erythropolis*): 54.7 kDa; RpNHase (*R. palustris*): α subunit: 23.7 kDa, β subunit: 24.2 kDa, activator protein: 15.5 kDa.

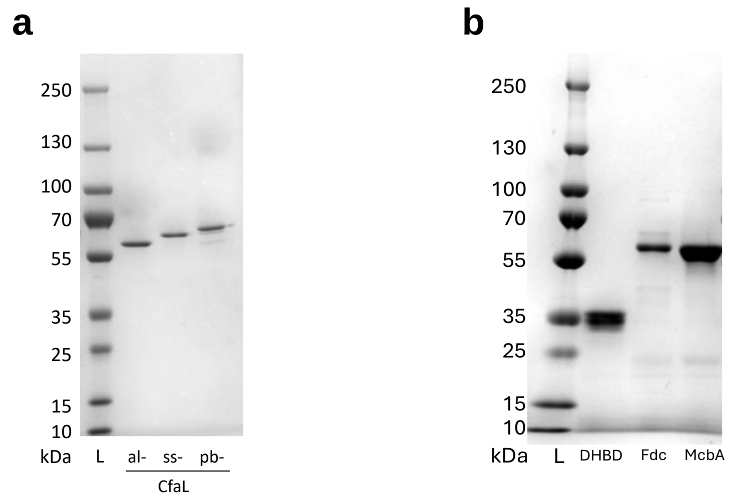

Figure S3. SDS-PAGE analysis of the Ni-NTA purified amide ligases and carboxylases used in this study. a, Purified CfaL enzymes. **b,** Purified carboxylases AoDHBD, AnFdc and an amide ligase McbA. L = protein ladder. Expected size: AlCfaL (*Azospirillum sp.*): 58.4 kDa; SsCfaL (*S. scabiei*): 58.8 kDa; PbCfaL (*P. Brasiliense*): 60.1 kDa; AoDHBD (*A. oryzae*): 39.9 kDa; AnFdc (*A. niger*): 56.8 kDa; McbA (*M. thermotolerans*): 55.0 kDa.

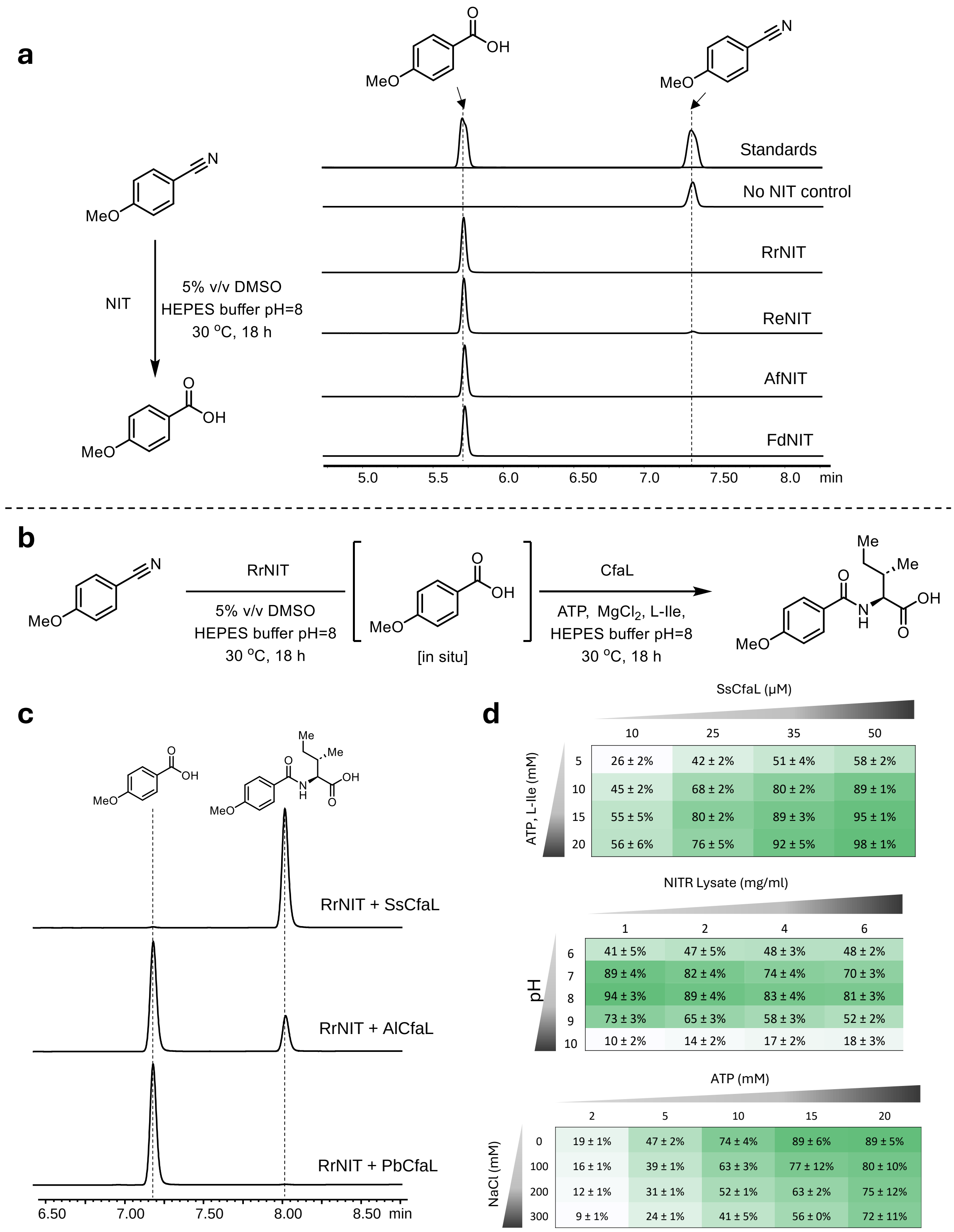

Figure S4. Optimisation of the NIT-CfaL enzyme cascade. a, Testing of the hydrolysis of 4-methoxybenzonitrile with various NITs. Assay conditions: nitrile (2 mM), NIT lysate (6 mg/mL) in HEPES buffer (100 mM, pH = 7) / DMSO (5% v/v), 30 °C, 18 h. **b,** Reaction scheme of the NIT-CfaL cascade on the conversion of 4-methoxybenzonitrile to amide **1** using RrNIT and various CfaL enzymes. **c,** Testing of the NIT-CfaL cascade using RrNIT and various CfaL enzymes. Nitrile was fully converted to the carboxylic acid in all cases. Assay conditions: nitrile (2 mM), RrNIT lysate (6 mg/mL), CfaL (50 μM), L-Ile (5 mM), ATP (5 mM), $MgCl_2$ (10 mM), in HEPES buffer (100 mM, pH = 7) / DMSO (5% v/v), 30 °C, 20 h. **d,** Optimisation of the RrNIT-SsCfaL cascade using the model reaction shown in panel b. After optimisation, the chosen

reaction conditions are: Nitrile (2 mM), NIT lysate (2 mg/mL), CfaL (25 µM), L-Ile (10 mM), ATP (10 mM), $MgCl_2$ (10 mM), HEPES buffer (100 mM, pH = 8) / DMSO (5% v/v), 30 °C, 18 h. Conversion was determined in triplicates by HPLC/UV analysis (n = 3). Conversions are presented as mean values ± Standard Error of the Mean.

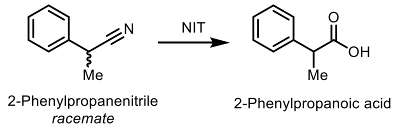

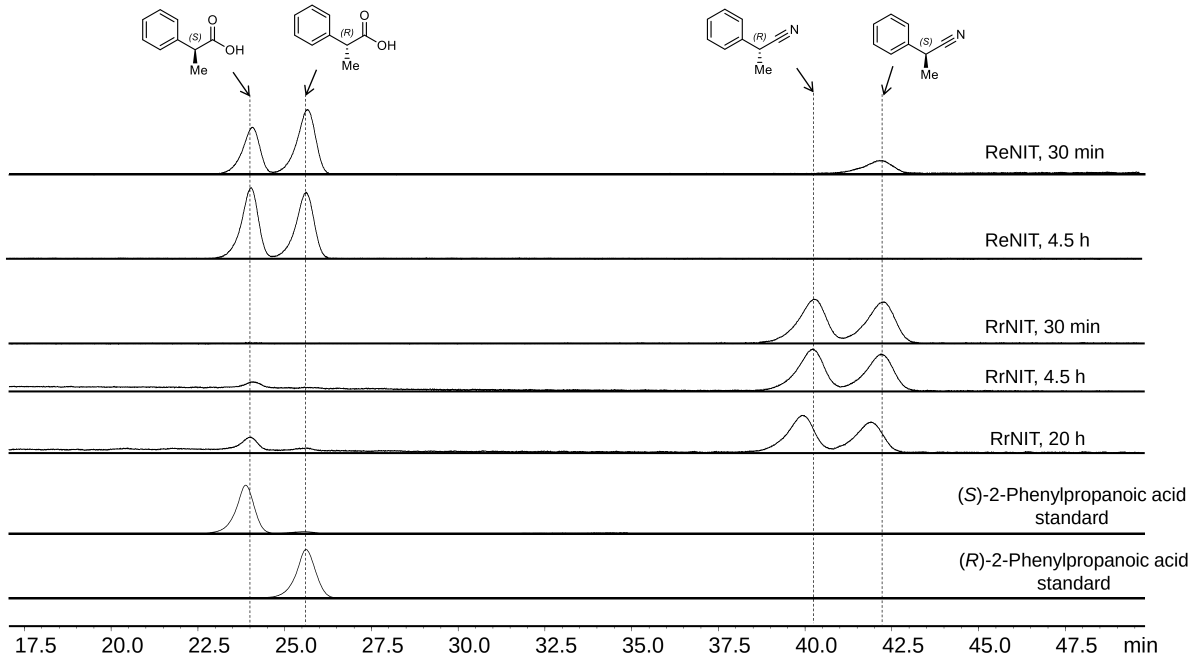

Figure S5. Activity and enantioselectivity test of ReNIT and RrNIT on substrate 2-phenylpropanenitrile. The reaction mixtures (500 µL) containing 2-phenylpropanenitrile (2 mM), NIT cell-free lysate (2 mg/mL), and MeOH (5% v/v) in HEPES buffer (100 mM, pH 8.0) were incubated at 30 °C for 20 h. The reactions were sampled at different time points for chiral HPLC analysis using a Lux Cellulose-3 column (250 × 4.6 mm, phenomenex™), with isocratic mobile phase of 80% H_2O (0.05% TFA) and 20% MeCN (0.05% TFA), flow rate of 1 mL/min, oven temperature of 40 °C, and UV detection of 210 nm.

a

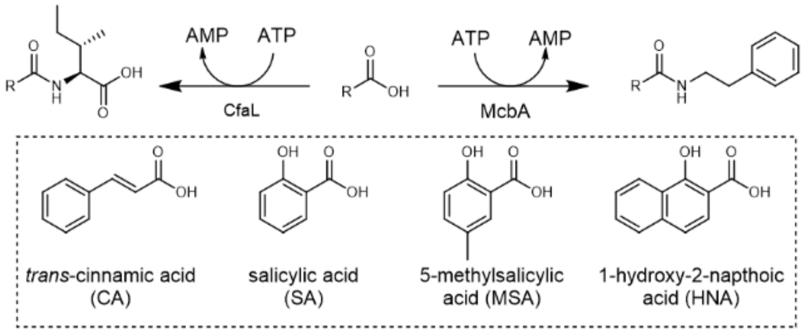

ABS enzyme

acyl donor	*Al*CfaL	*Pb*CfaL R395G	*Ss*CfaL	McbA
CA	66 ± 9%	26 ± 1%	74 ± 2%	74 ± 2%
SA	46 ± 2%	18 ± 0.3%	10 ± 0.3%	3 ± 0.3%
MSA	65 ± 7%	86 ± 1%	79 ± 5%	19 ± 0.1%
HNA	14 ± 1%	< 1%	32 ± 2%	> 99%

b

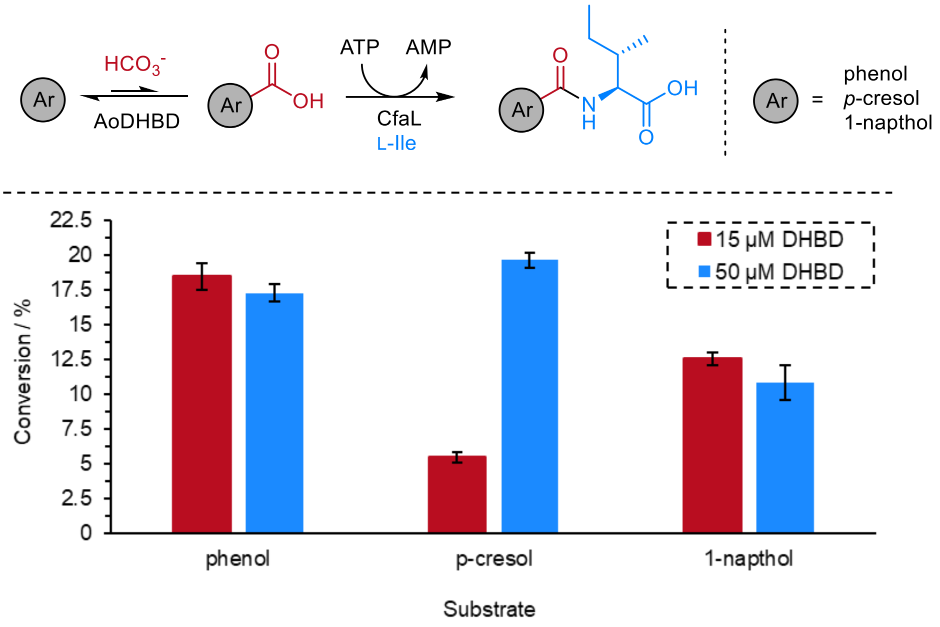

Figure S6. Enzyme activity assays for optimisation of enzymatic C-H amidation cascades. a, Amide ligation activity screening of amide bond synthetase (ABS) enzymes against (de)carboxylase producible carboxylic acids. Percentage conversions (± standard deviation) were determined in triplicate by HPLC using calibration curves with synthetic standards. The reaction mixture containing carboxylic acid (2 mM), L-Ile or phenylethylamine (5 mM), ATP (5 mM), $MgCl_2$ (10 mM) and ABS (25 µM) in Tris-HCl buffer (50 mM, pH 8.0) were incubated for 20 hr at 30 °C with shaking (700 rpm) prior to the reaction being stopped by the addition of 100 µL MeCN. The reaction was cleared by centrifuge and the supernatant was analysed via HPLC method D. The data regarding the CfaL catalysed amidation of trans-cinnamic acid has been reported previously.[4] **b**, Chart displaying the difference in conversion of phenolic substrates phenol, *p*-cresol and 1-napthol to secondary amides respectively, when the cascades were run with AoDHBD (15 or 50 µM). All cascades were incubated at 20 °C for 5 days.

Table S1: Solvent screen for the C-H bond cyanation of naphthalene

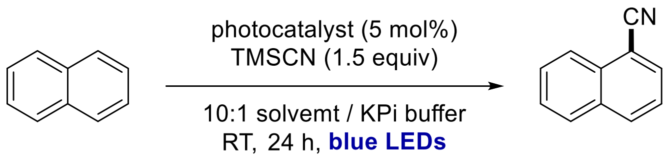

Entry	Solvent	Yield /%[a]
1	MeCN	61 (49)
2	DMSO	-
3	DMF	-
4	DMA	-
5	Ethyl acetate	0
6	EtOH	17
7	*i*PrOH	15
8	1-Butanol	trace
9	2-Ethoxyethanol	-
10	THF	-
11	Acetone	8
12	Butanone	10
13	DCM	31
14	DCE	33
15	Nitroethane	trace

Reaction conditions: Arene (13 mg, 0.1 mmol, 1 equiv), TMSCN (19 µL, 0.15 mmol, 1.5 equiv), 9-Mesityl-3,6-di-*tert*-butyl-10-phenylacridinium tetrafluoroborate (2.9 mg, 0.05 mmol, 5 mol%) in 10:1 solvent / 4 M KPi buffer (1.1 mL) under O_2 atmosphere. [a] Conversion was determined by HPLC/UV using benzophenone as an internal standard. Yield in parentheses refers to isolated product after column chromatography.

Preparative scale enzyme cascades

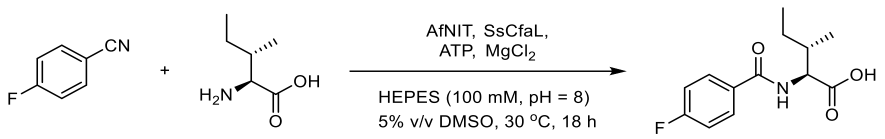

7

A reaction mixture (20 mL) consisting of 4-fluorobenzonitrile (12.1 mg, 5 mM), L-Ile (15 mM), ATP (15 mM), $MgCl_2$ (10 mM), AfNIT cell-free lysate (5 mg/mL), SsCfaL (25 µM), in HEPES buffer (100 mM, pH 8.0) with DMSO (5% (v/v)) was incubated at 30 °C for 18 h with shaking (190 rpm). A 10 µL sample was taken for HPLC analysis, indicating reaction completion before acidifying the reaction with HCl (2 M, to pH 2-3). The reaction was then extracted with EtOAc (15 mL × 3). The organic layers were combined, washed with brine, dried over $MgSO_4$, and concentrated *in vacuo* to obtain crude product. The crude product was purified by silica gel column, giving 19.8 mg of **7** as white solid in 78% isolated yield.

^{1}H NMR (400 MHz, $CDCl_3$) δ 7.86 – 7.76 (m, 2H), 7.16 – 7.06 (m, 2H), 6.72 (d, *J* = 8.3 Hz, 1H), 4.80 (dd, *J* = 8.3, 4.8 Hz, 1H), 2.10 – 2.01 (m, 1H), 1.64 – 1.50 (m, 1H), 1.34 – 1.27 (m, 1H), 1.04 – 0.93 (m, 6H).

^{13}C NMR (101 MHz, $CDCl_3$) δ 166.9, 166.4, 163.9, 130.1, 129.7, 129.6, 116.0, 115.8, 57.2, 38.0, 25.4, 15.6, 11.7.

^{19}F NMR (376 MHz, $CDCl_3$) δ -107.28.

HR-MS (ESI), calc. for $[M+H]^+$: 254.1187, observed: 254.1191.

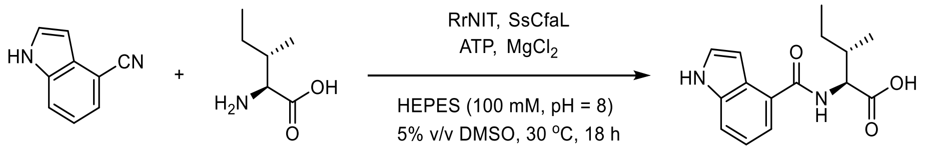

20

A reaction mixture (20 mL) consisting of indole-4-carbonitrile (14.2 mg, 5 mM), L-Ile (15 mM), ATP (15 mM), $MgCl_2$ (10 mM), RrNIT cell-free lysate (5 mg/mL), SsCfaL (25 µM), in HEPES buffer (100 mM, pH 8.0) with DMSO (5% (v/v)) was incubated at 30 °C for 18 h with shaking (190 rpm). A 10 µL sample was taken for HPLC analysis, indicating reaction completion before acidifying the reaction with HCl (2 M, to pH 2-3). The reaction was then extracted with EtOAc (15 mL × 3). The organic layers were combined, washed with brine, dried over $MgSO_4$, and concentrated *in vacuo* to obtain crude product. The crude product was purified by silica gel column, giving 22.2 mg of **20** as white solid in 81% isolated yield.

^{1}H NMR (400 MHz, MeOD) δ 7.57 (d, *J* = 8.1 Hz, 1H), 7.47 (d, *J* = 7.5 Hz, 1H), 7.38 (d, *J* = 3.2 Hz, 1H), 7.19 (t, *J* = 7.8 Hz, 1H), 6.86 (dd, *J* = 3.2, 1.0 Hz, 1H), 4.70 (d, *J* = 5.6 Hz, 1H), 2.15 – 2.02 (m, 1H), 1.72 – 1.60 (m, 1H), 1.41 – 1.31 (m, 1H), 1.06 (d, *J* = 6.8 Hz, 3H), 1.00 (t, *J* = 7.4 Hz, 3H).
^{13}C NMR (101 MHz, MeOD) δ 175.1, 171.7, 138.3, 127.5, 127.2, 127.0, 121.7, 120.4, 115.7, 101.9, 58.5, 38.6, 26.6, 16.1, 11.9.
HR-MS (ESI), calc. for $[M+H]^+$: 275.1390, observed: 275.1385.

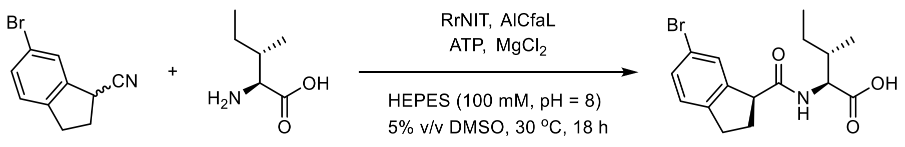

31

A reaction mixture (20 mL) consisting of 6-bromo-2,3-dihydro-1*H*-indene-1-carbonitrile (racemic, 22.2 mg, 5 mM), L-Ile (15 mM), ATP (15 mM), $MgCl_2$ (10 mM), RrNIT cell-free lysate (5 mg/mL), AlCfaL (25 µM), in HEPES buffer (100 mM, pH 8.0) with DMSO (5% (v/v)) was incubated at 30 °C for 18 h with shaking (190 rpm). A 10 µL sample was taken for HPLC analysis, indicating reaction completion before acidifying the reaction with HCl (2 M, to pH 2-3). The reaction was then extracted with EtOAc (15 mL × 3). The organic layers were combined, washed with brine, dried over $MgSO_4$, and concentrated *in vacuo* to obtain crude product. The crude product was purified by silica gel column, giving 12.0 mg of **31** as white solid in 34% isolated yield (single diastereomer).
^{1}H NMR (400 MHz, $CDCl_3$) δ 7.45 (s, 1H), 7.33 (dd, *J* = 8.1, 2.1 Hz, 1H), 7.13 (d, *J* = 8.1 Hz, 1H), 6.06 (d, *J* = 8.5 Hz, 1H), 4.61 (dd, *J* = 8.6, 4.7 Hz, 1H), 3.95 (t, *J* = 7.6 Hz, 1H), 3.08 – 2.96 (m, 1H), 2.92 – 2.79 (m, 1H), 2.50 – 2.27 (m, 2H), 2.02 – 1.91 (m, 1H), 1.50 – 1.38 (m, 1H), 1.19 – 1.06 (m, 1H), 0.97 – 0.86 (m, 6H).
^{13}C NMR (101 MHz, $CDCl_3$) δ 175.8, 173.9, 143.8, 143.5, 131.0, 128.0, 126.6, 120.4, 56.7, 52.3, 37.5, 31.5, 30.7, 25.2, 15.7, 11.7.
HR-MS (ESI): calc. for $[M+H]^+$: 354.0699, observed: 354.0696.

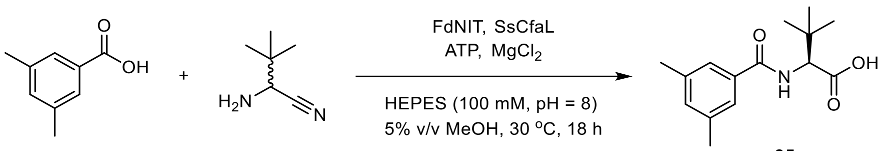

35

A reaction mixture (50 mL) consisting of 3,5-dimethylbenzoic acid (15.0 mg, 2 mM), racemic 2-amino-3,3-dimethylbutanenitrile (10 mM), ATP (5 mM), $MgCl_2$ (10 mM), FdNIT cell-free lysate (2 mg/mL), SsCfaL (15 µM), in HEPES buffer (100 mM, pH 8.0) with MeOH (5% (v/v)) was incubated at 30 °C for 24 h with shaking (200 rpm). A 50 µL sample was taken for HPLC analysis, indicating reaction completion before acidifying the reaction with HCl (2M, to pH 1-2). The reaction was then extracted with EtOAc (25 mL × 3). The organic layers were combined, washed with brine, dried over $MgSO_4$, and concentrated *in vacuo* to obtain crude product. The crude product was purified by reverse phase C18 column using water with formic acid (0.1% (v/v)) and MeCN with formic acid (0.1% (v/v)) as eluent (eluent gradient of MeCN from 10-100%). 22.4 mg of compound **35** was purified as white solid in 85% isolated yield (ee > 99%).

^{1}H NMR (400 MHz, $CDCl_3$) δ 7.38 (s, 2H), 7.15 (s, 1H), 6.62 (d, *J* = 9.2 Hz, 1H), 4.72 (d, *J* = 9.2 Hz, 1H), 2.36 (s, 6H), 1.10 (s, 9H).

^{13}C NMR (101 MHz, $CDCl_3$) δ 175.6, 168.3, 138.6, 134.0, 133.7, 124.9, 60.6, 35.2, 26.8, 21.4.

HR-MS (ESI): calc. for $[M+H]^+$: 264.1594, observed: 264.1596.

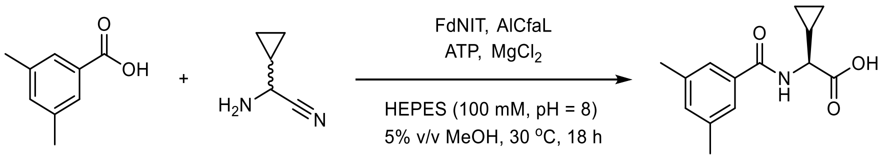

38

A reaction mixture (50 mL) consisting of 3,5-dimethylbenzoic acid (15.0 mg, 2 mM), racemic 2-amino-2-cyclopropylacetic acid (10 mM), ATP (5 mM), $MgCl_2$ (10 mM), FdNIT cell-free lysate (2 mg/mL), AlCfaL (15 µM), in HEPES buffer (100 mM, pH 8.0) with MeOH (5% (v/v))was incubated at 30 °C for 24 h with shaking (200 rpm). A 50 µL sample was taken for HPLC analysis, indicating reaction completion before acidifying the reaction with HCl (2 M, to pH 1-2). The reaction was then extracted with EtOAc (25 mL × 3). The organic layers were combined, washed with brine, dried over $MgSO_4$, and concentrated *in vacuo* to obtain crude product. The crude product was purified by reverse phase C18 column using water with formic

acid (0.1% (v/v)) and MeCN with formic acid (0.1% (v/v)) as eluent (eluent gradient of MeCN from 10-100%). 23.0 mg of compound **38** was purified as white solid in 93% isolated yield (ee > 99%).

^{1}H NMR (400 MHz, MeOD) δ 7.47 (s, 2H), 7.18 (s, 1H), 3.83 (d, *J* = 9.3 Hz, 1H), 2.35 (s, 6H), 1.35 – 1.19 (m, 1H), 0.75 – 0.51 (m, 3H), 0.48 – 0.38 (m, 1H).

^{13}C NMR (101 MHz, MeOD) δ 175.3, 170.6, 139.4, 135.1, 134.2, 126.2, 58.9, 21.3, 13.9, 13.9, 4.3, 4.2.

HR-MS (ESI): calc. for $[M+H]^{+}$: 248.1281, observed: 248.1282.

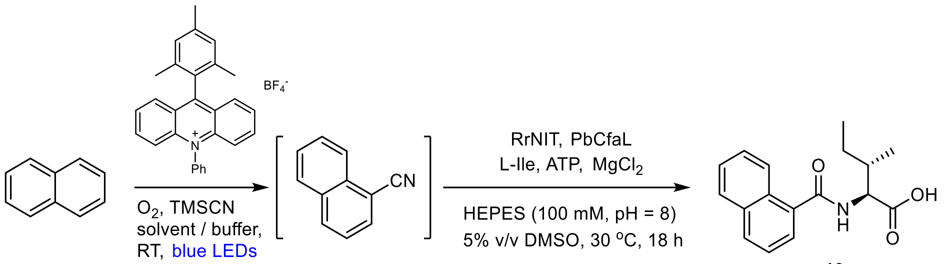

13

To a solution of naphthalene (9.0 mg, 100 mM) in 700 µL 10:1 MeCN / KPi buffer (4 M, pH 9.0) was added 9-mesityl-3,6-di-tert-butyl-10-phenylacridinium tetrafluoroborate (5 mM) and the reaction was sparged with oxygen gas for 5 minutes. TMSCN (150 mM) was added, and the reaction was irradiated with blue LEDs for 24-48 h. After that, the solvent was removed under reduced pressure and the residue containing crude nitrile was taken up in DMSO (700 µL, 100 mM final concentration, based on maximum theoretical conversion). This crude nitrile DMSO solution was used in the biocatalytic reaction mixture (14 mL) consisting of 1-naphthonitrile (crude, 5 mM), L-Ile (15 mM), ATP (15 mM), $MgCl_2$ (10 mM), RrNIT cell-free lysate (5 mg/mL), PbCfaL (25 µM), in HEPES buffer (100 mM, pH 8.0) with DMSO (5% (v/v)). The reaction mixture was incubated at 30 °C for 18 h with shaking (200 rpm). A 10 µL sample was taken for HPLC analysis, indicating reaction completion before acidifying the reaction with HCl (2 M, to pH 2-3). The reaction was then extracted with EtOAc (10 mL × 3). The organic layers were combined, washed with brine, dried over $MgSO_4$, and concentrated *in vacuo* to obtain crude product. The crude product was purified by silica gel column, giving 10.0 mg of white solid **13** in 50% isolated yield.

^{1}H NMR (400 MHz, $CDCl_3$) δ 8.32 (d, *J* = 8.2 Hz, 1H), 7.93 (d, *J* = 8.2 Hz, 1H), 7.87 (d, *J* = 7.8 Hz, 1H), 7.66 (d, *J* = 7.1 Hz, 1H), 7.60 – 7.49 (m, 2H), 7.48 – 7.42 (m, 1H), 6.52 (d, *J* = 8.6 Hz, 1H), 4.95 (dd, *J* = 8.7, 4.6 Hz, 1H), 2.18 – 2.07 (m, 1H), 1.64 – 1.49 (m, 1H), 1.36 – 1.26 (m, 1H), 1.07 (d, *J* = 6.8 Hz, 3H), 0.97 (t, *J* = 7.4 Hz, 3H).

^{13}**C NMR** (101 MHz, $CDCl_3$) δ 176.0, 169.9, 133.8, 133.8, 131.2, 130.2, 128.5, 127.5, 126.7, 125.4, 125.4, 124.8, 57.1, 37.8, 25.3, 15.8, 11.8.
HR-MS (ESI): calc. for $[M+H]^+$: 286.1438, observed: 286.1436.

Preparation of starting materials

General synthesis method A: Preparation of α-aminonitriles

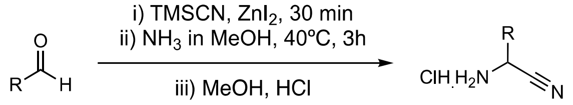

Performed according to a literature procedure.[7] To a mixture of aldehyde (5 mmol, 1 eq.) and TMSCN (750 µL, 12 mmol, 1.2 eq.) was added ZnI_2 (96 mg, 0.5 mmol, 0.1 eq.). After stirring for 30 minutes, 7 N NH_3 in MeOH (10 mL) was added. Then the mixture was warmed to 40 °C and stirred for 3 h. The solvent was removed under reduced pressure, and the residue was taken up with Et_2O, dried over $MgSO_4$ and filtered off. The filtrate was acidified with 4 M HCl in MeOH, the solvent was removed under reduced pressure and ether was added again. The solid formed was collected by filtration.

General synthesis method B: Synthesis of α-aminonitriles

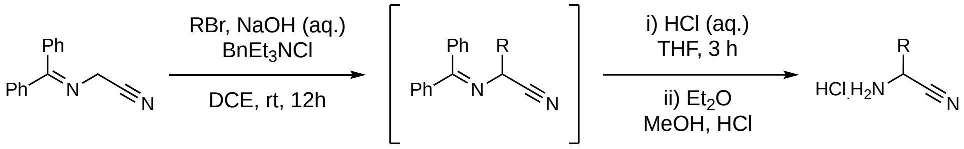

Performed according to a literature procedure.[7] To a solution of 2-[(diphenylmethylene)-amino]acetonitrile (10 mmol, 1 eq.) in DCE (50 mL) was added the alkyl halide (10.5 mmol, 1.05 eq.), 11 M NaOH solution (100 mmol, 10 eq.) and benzyltriethylammonium chloride (1 mmol, 0.1 eq.). The mixture was stirred vigorously at room temperature for 12 h. After that time, the mixture was diluted with water, extracted with DCM (3×) and the combined organic layers were concentrated under reduced pressure. The crude product was dissolved in THF (50 mL) and 1 M HCl solution (15 mL) was added. After stirring 3 h, the reaction mixture was poured into water and washed with Et_2O (3×). The aqueous layer was neutralized by the addition of 10 M NaOH and extracted with DCM (3×). The combined organic layers were dried over Na_2SO_4 and concentrated under reduced pressure. Et_2O was added into the residue and acidified with 4 M HCl in MeOH. The precipitated was collected by filtration.

Preparation of amide synthetic standards

General synthesis method C: EDC-coupling of amino acids and carboxylic acids

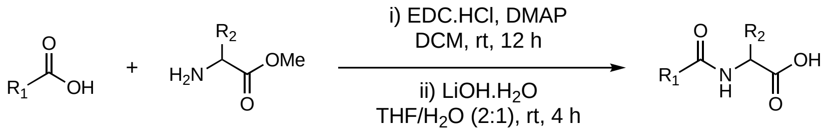

Performed according to a literature procedure.[4] Step 1) To a solution of carboxylic acid (0.4 mmol, 1 eq.) in DCM (8 mL) was added amino acid methyl ester hydrochloride (0.52 mmol, 1.3 eq.), EDC*HCl (100 mg, 0.52 mmol, 1.3 eq.) and DMAP (147 mg, 1.4 mmol, 3 eq.) and the reaction was stirred at room temperature for 12 h. After completion, the reaction was diluted with DCM (20 mL) and washed with sat. $NaHCO_3$ solution (15 mL), 1 M HCl (15 mL) and the organic layer was dried over $MgSO_4$. The solvent was removed under reduced pressure and the ester product isolated by silica gel column chromatography (eluent: n-hexane / ethyl acetate). Step 2) To a solution of the ester product in 2:1 THF / H_2O (4 mL), LiOH*H_2O (67 mg, 1.6 mmol, 4 eq.) was added and the reaction was stirred for 4 h at room temperature. After that time, the reaction was diluted with water (10 mL), washed with Et_2O (15 mL) and acidified to pH 2-3 with 6 M HCl. The aqueous phase was extracted with EtOAc (3 × 20 mL), the combined organic layers were dried over $MgSO_4$ and concentration under reduced pressure to yield the desired amide product.

General synthesis method D: PyBOP-coupling of amino acids and carboxylic acids

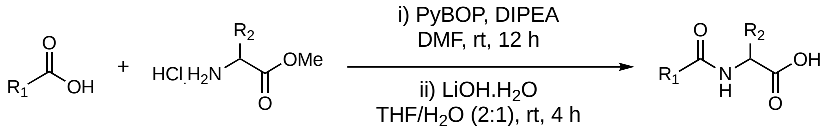

Performed according to a literature procedure.[8] Step 1) To a solution of carboxylic acid (0.4 mmol, 1 eq.) in DMF (2 mL) was added amino acid methyl ester hydrochloride (0.48 mmol, 1.2 eq.), PyBOP (223 mg, 0.44 mmol, 1.1 eq.) and DIPEA (209 mg, 1.4 mmol, 3 eq.) and the reaction was stirred at room temperature for 12 h. After completion, the reaction was diluted with DCM (20 mL) and washed with sat. $NaHCO_3$ solution (15 mL), 1 M HCl (15 mL) and the organic layer was dried over $MgSO_4$. The solvent was removed under reduced pressure and the ester product isolated by silica gel column chromatography (eluent: n-hexane / ethyl acetate). Step 2) To a solution of the ester product in 2:1 THF / H_2O (6 mL), LiOH.H_2O (67 mg, 1.6 mmol, 4 eq.) was added and the reaction was stirred for 4 h at room temperature. After that time, the reaction was diluted with water (10 mL) and acidified to pH 2-3 with 6 M HCl. The

aqueous phase was extracted with EtOAc (3 × 20 mL), the combined organic layers were dried over $MgSO_4$ and concentration under reduced pressure to yield the desired amide product.

General synthesis method E: Acylation of amino acids with carboxylic chlorides

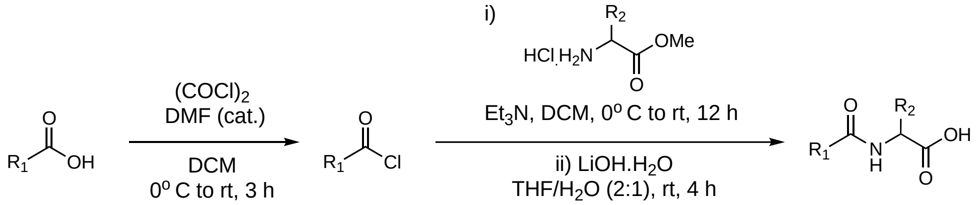

Performed according to a literature procedure.[9] Step 1) To a solution of carboxylic acid (2.5 mmol, 1 eq.) in DCM (2.5 mL) was dropwise added oxalyl chloride (275 µL, 3.25 mmol, 1.3 eq.) and 1-2 drops of DMF sequentially at 0 °C. The reaction was allowed to warm to room temperature and stirred for 3 h. After reaction was completed, the reaction was diluted with DCM (20 mL) and washed with sat. $NaHCO_3$ solution (15 mL). The organic layer was dried over $MgSO_4$ and concentration under reduced pressure. The crude acid chloride was used directly for the next step. Step 2) To a solution of amino acid methyl ester hydrochloride (2.5 mmol, 1.1 eq.) and Et_3N (193 mg, 1.82 mmol, 2 eq.) was added a solution of the acid chloride in DCM (10 mL) was added dropwise at 0 °C. The reaction was allowed to warm to room temperature and stirred for 12 h. After that time, the reaction was partitioned between DCM (20 mL) and water (20 mL), and the organic layer was washed with sat. $NaHCO_3$ solution (2 × 40 mL) and 10% HCl (40 mL). The organic layer was dried over $MgSO_4$ and concentrated *in vacuo*, and the amide methyl ester was isolated via column chromatography. Step 3) To a solution of the ester product in 2:1 THF / H_2O (6 mL), LiOH.H_2O (67 mg, 1.6 mmol, 4 eq.) was added and the reaction was stirred for 4 h at room temperature. After that time, the reaction was diluted with water (10 mL) and acidified to pH 2-3 with 6 M HCl. The aqueous phase was extracted with EtOAc (3 × 20 mL), the combined organic layers were dried over $MgSO_4$ and concentration under reduced pressure to yield the desired amide product.

General synthesis method F: PyBOP-coupling of 2-phenylethylamine and carboxylic acids.

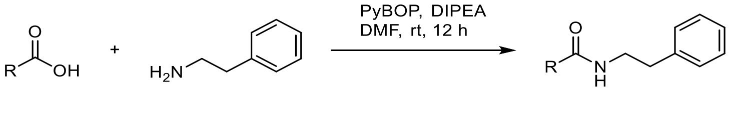

To a solution of carboxylic acid (0.6 mmol, 1 eq.) in DMF (3 mL) was added 2-phenylethylamine (0.72 mmol, 1.2 eq.), PyBOP (335 mg, 0.66 mmol, 1.1 eq.) and DIPEA

(314 mg, 2.1 mmol, 3 eq.) and the reaction was stirred at room temperature for 12 h. After completion, the reaction was diluted with DCM (30 mL) and washed with sat. $NaHCO_3$ solution (23 mL), 1 M HCl (23 mL) and the organic layer was dried over $MgSO_4$. The solvent was removed under reduced pressure and the amide product isolated by silica gel column chromatography.

Experimental data of starting materials

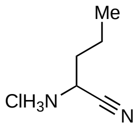

2-Aminopentanenitrile hydrochloride (54) was prepared according to general synthesis method A using butyraldehyde (450 µL, 5 mmol, 1 eq.). The product was obtained as an orange solid (536 mg, 3.98 mmol, 79%).

^{1}H NMR (400 MHz, DMSO) δ 9.32 (s, 3H), 4.54 (dd, J = 8.4, 6.4 Hz, 1H), 1.92 – 1.79 (m, 2H), 1.45 (m, 2H), 0.92 (t, J = 7.4 Hz, 3H).

^{13}C NMR (101 MHz, DMSO) δ 116.9, 40.4, 32.1, 18.0, 13.1.

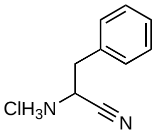

2-Amino-3-phenylpropanenitrile hydrochloride (55) was prepared according to general synthesis method A using phenylacetaldehyde (584 µL, 5 mmol, 1 eq.). The product was obtained as a pale orange solid (513 mg, 3.51 mmol, 70%).

^{1}H NMR (400 MHz, DMSO) δ 9.51 (s, 3H), 7.42 – 7.29 (m, 5H), 4.85 (dd, J = 10.4, 5.2 Hz, 1H), 3.41 – 3.09 (m, 2H).

^{13}C NMR (101 MHz, DMSO) δ 133.8, 129.5, 128.8, 127.8, 116.4, 42.0, 35.9.

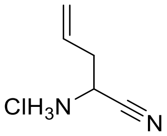

2-Aminopent-4-enenitrile hydrochloride (56) was prepared according to general synthesis method B using 2-((diphenylmethylene)amino) acetonitrile (1.1 g, 5 mmol, 1 eq.) and allyl bromide (0.45 mL, 5.25 mmol, 1.05 eq.). The product was obtained as a white solid (247 mg, 1.87 mmol, 37%).

^{1}H NMR (400 MHz, DMSO) δ 9.29 (br s, 3H), 5.79 (m, 1H), 5.33-5.26 (m, 2H), 4.66 (m, 1H), 2.66 (m, 2H)

^{13}C NMR (101 MHz, DMSO) δ 130.9, 121.3, 116.9, 40.7, 34.8.

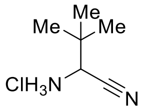

2-Amino-3,3-dimethylbutanenitrile hydrochloride (57) was prepared according to general synthesis method A using trimethylacetaldehyde (1.09 mL, 10 mmol, 1 eq.). The product was obtained as a white solid (661 mg, 4.48 mmol, 44%).

^{1}H NMR (400 MHz, DMSO) δ 9.32 (s, 3H), 4.49 (s, 1H), 1.08 (s, 9H)

^{13}C NMR (101 MHz, DMSO) δ 116.5, 50.5, 34.3, 26.0.

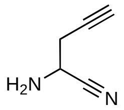

2-Aminopent-4-ynenitrile (58) was prepared according to general synthesis method B using propargyl bromide (0.56 mL of an 80% solution in toluene, 5.25 mmol). The product was obtained as a yellow liquid (103 mg, 1.09 mmol, 21%).

^{1}H NMR (400 MHz, $CDCl_3$) 3.88 (t, *J* = 7.0 Hz, 1H), 3.03 (t, *J* = 2.62 Hz, 1H) 2.55 – 2.54 (m, 1H), 2.53 (t, *J* = 2.62 Hz), 2.40 (br s, 1H)

^{13}C NMR (101 MHz, $CDCl_3$) δ 122.5, 80.1, 74.5, 43.2, 25.6.

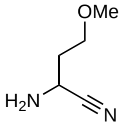

2-Amino-4-methoxybutanenitrile (59) was prepared according to general synthesis method B using 1,2-methoxybromoethane (0.79 mL, 8.4 mmol). The product was obtained as a brown liquid (49 mg, 0.426 mmol, 5%).

^{1}H NMR (400 MHz, $CDCl_3$) 3.72 (t, *J* = 7.51 Hz, 1H) 3.50 – 3.36 (m, 2H), 3.23 (s, 3H), 2.32 (br s, 2H), 1.82 (q, *J* = 6.24 Hz, 2H)

^{13}C NMR (101 MHz, $CDCl_3$) δ 123.5, 68.4, 58.5, 40.7, 35.1.

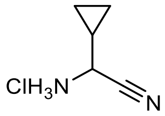

2-Amino-2-cyclopropylacetonitrile hydrochloride (60) was prepared according to general synthesis method A using cyclopropanecarboxaldehyde (0.75 mL, 10 mmol, 1 eq.). The product was obtained as a pale-yellow solid (198 mg, 1.49 mmol, 15%).

^{1}H NMR (400 MHz, DMSO) 9.19 (br s, 3H), 4.17 (d, *J* = 9.13 Hz, 1H), 1.44 – 1.34 (m, 1H), 0.73 – 0.64 (m, 2H), 0.63 – 0.56 (m, 2H).

^{13}C NMR (101 MHz, DMSO) δ 116.3, 44.2, 11.8, 5.1, 3.1.

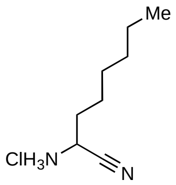

2-Aminooctanenitrile hydrochloride (61) was prepared according to general synthesis method A using heptanal (705 µL, 5 mmol, 1 equiv). The product was obtained as a pale white solid (216 mg, 1.22 mmol, 24%).

^{1}H NMR (400 MHz, DMSO) δ 9.28 (s, 3H), 4.52 (dd, *J* = 8.9, 5.8 Hz, 1H), 1.96 – 1.78 (m, 2H), 1.44 – 1.20 (m, 8H), 0.86 (t, *J* = 6.7 Hz, 3H).

^{13}C NMR (101 MHz, DMSO) δ 116.9, 40.6, 30.8, 30.1, 27.7, 24.5, 21.9, 13.9.

Experimental data of amide synthetic standards

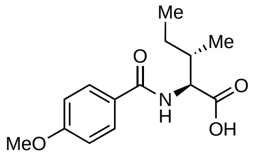

(4-Methoxybenzoyl)-L-isoleucine (1) was prepared according to general synthesis method C using 4-methoxybenzoic acid (61 mg, 0.4 mmol, 1 eq.) and L-isoleucine methyl ester hydrochloride (95 mg, 0.52 mmol, 1.3 eq.). The product was obtained as a white solid (69 mg, 0.26 mmol, 65%).

^{1}H NMR (400 MHz, DMSO) *δ* 12.51 (s, 1H), 8.24 (d, *J* = 8.0 Hz, 1H), 7.88 (d, *J* = 8.8 Hz, 2H), 6.99 (d, *J* = 8.8 Hz, 2H), 4.31 (t, *J* = 7.6 Hz, 1H), 3.81 (s, 3H), 1.98 – 1.89 (m, 1H), 1.53 – 1.24 (m, 2H), 0.92 (d, *J* = 6.8 Hz, 3H), 0.86 (t, *J* = 7.4 Hz, 3H).

^{13}C NMR (101 MHz, DMSO) *δ* 173.4, 166.1, 161.7, 129.5, 126.3, 113.4, 57.2, 55.4, 35.7, 25.2, 15.7, 11.0.

HR-MS: calc. for $[M+Na]^{+}$ $C_{14}H_{19}NO_4$ = 288.1206, observed: 288.1204 (-0.7 ppm)

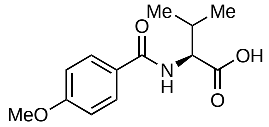

(4-Methoxybenzoyl)-L-Valine (2) was prepared according to general synthesis method D using of 4-methoxybenzoic acid (61 mg, 0.4 mmol, 1 eq.) and L-valine methyl ester hydrochloride (80 mg, 0.48 mmol, 1.2 eq.). The product was obtained as an off-white sticky solid (66 mg, 0.26 mmol, 66%).

^{1}H NMR (400 MHz, DMSO) δ 12.60 (br s, 1H), 8.22 (d, *J* = 8.10 Hz, 1H), 7.90 – 7.85 (m, 2H), 7.02 – 6.96 (m, 2H), 4.26 (dd, *J* = 7.89, 7.12, 1H), 3.81 (s, 3H), 2.17 (m, 1H), 0.96 (t, 6H)

^{13}C NMR (101 MHz, DMSO) δ 173.3, 166.2, 161.7, 129.5, 126.3, 113.4, 58.3, 55.4, 29.5, 19.3, 18.9.

HR-MS: calc. for $[M+Na]^{+}$ = 274.1050, observed: 274.1049 (-0.5 ppm)

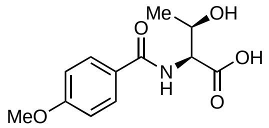

(4-Methoxybenzoyl)-L-threonine (3) was prepared according to the following procedure. To a solution of 4-methoxybenzoic acid (61 mg, 0.4 mmol, 1 eq.) in DMF (2 mL) was added *O*-(*tert*-butyl)- L-threonine *tert*-butyl ester acetate (140 mg, 0.48 mmol, 1.2 eq.), PyBOP (223 mg,

0.44 mmol, 1.1 eq.) and DIPEA (209 µL, 1.4 mmol, 3 eq.) and the reaction was stirred at room temperature for 20 h. After completion, the reaction was diluted with DCM (20 mL) and washed with sat. $NaHCO_3$ solution (15 mL), 1 M HCl (15 mL) and the organic layer was dried over $MgSO_4$. The solvent was removed under reduced pressure and the ester product purified by silica gel column chromatography (n-hexane / ethyl acetate). The procedure for removal of the *tert*-butyl groups was adapted from a literature procedure.[10] To a solution of the ester product in DCM (1 mL) was added aqueous H_3PO_4 (1.3 mL of an 85 wt% solution in H_2O, 10 eq.) and the reaction was stirred at room temperature for 18 h. Upon completion, the reaction mixture was diluted with water (50 mL) and extracted with EtOAc (3 x 50 mL). The combined organic extracts were dried over $MgSO_4$ and concentrated in vacuo to give the desired product. The product was obtained as an off-white solid (73.6 mg, 0.29 mmol, 73%).

^{1}H NMR (400 MHz, DMSO) δ 7.87 (m, 3H), 7.02 (m, 2H), 4.40 (dd, *J* = 8.51, 3.66 Hz, 1H), 4.18 (qd, 6.39, 3.80, 1H), 3.81 (s, 3H), 1.13 (d, *J* = 6.53 Hz, 3H). COOH, OH protons overlapped by residual water from DMSO.

^{13}C NMR (101 MHz, DMSO) δ 172.7, 166.7, 162.3, 129.7, 126.6, 114.0, 67.1, 59.1, 55.9, 21.0.

HR-MS: calc. for $[M+Na]^+$ = 276.0842, observed: 276.0841 (-0.5 ppm)

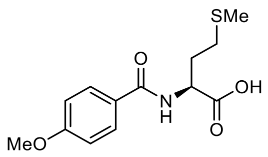

(4-Methoxybenzoyl)-L-methionine (4) was prepared according to general synthesis method D using 4-methoxybenzoic acid (61 mg, 0.4 mmol, 1 eq.) and L-methionine methyl ester hydrochloride (96 mg, 0.48 mmol, 1.2 eq.). The product was obtained as a white solid (39 mg, 0.15 mmol, 38%).

^{1}H NMR (400 MHz, DMSO) δ 12.62 (br s, 1H), 8.46 (d, *J* = 7.96 Hz, 1H), 7.90 – 7.83, (m, 2H), 7.05 – 6.96 (m, 2H), 4.49 (q, *J* = 7.10, 1H), 3.81 (s, 3H), 2.63 – 2.49 (m, 2H), 2.09 – 2.01 (m, 5H)

^{13}C NMR (101 MHz, DMSO) δ 174.1, 166.6, 162.2, 129.8, 126.6, 113.9, 55.8, 52.1, 30.8, 30.6, 15.1.

HR-MS: calc. for $[M+Na]^+$ = 306.0770, observed: 306.0764 (-2 ppm)

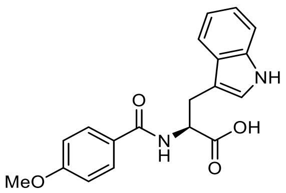

(4-Methoxybenzoyl)tryptophan (5) was prepared according to general synthesis method D using of 4-methoxybenzoic acid (61 mg, 0.4 mmol, 1 eq.) and L-tryptophan methyl ester hydrochloride (122 mg, 0.48 mmol, 1.2 eq). The product was obtained as an off-white sticky solid (61 mg, 0.18 mmol, 45%).

^{1}H NMR (400 MHz, DMSO) δ 12.59 (br s, 1H), 10.80 (s, 1H), 8.47 (d, J = 7.90 Hz, 1H), 7.81 (d, J = 8.66 Hz, 2H), 7.59 (d, J = 7.79 Hz, 1H), 7.32 (d, J = 8.02 Hz), 7.19 (d, J = 1.74 Hz, 1H), 7.05 (t, J = 7.67 Hz, 1H), 7.01 – 6.94 (m, 3H), 4.86 – 4.60 (m, 1H), 3.80 (s, 1H), 3.40 – 3.16 (m, 2H)

^{13}C NMR (101 MHz, DMSO) δ 173.8, 165.8, 161.7, 136.1, 129.3, 127.2, 126.2, 123.6, 120.9, 118.4, 118.2, 113.4, 111.4, 110.5, 55.4, 53.6, 26.7.

HR-MS: calc. for $[M-H]^-$: 337.1193, observed: 337.1191 (-0.8 ppm)

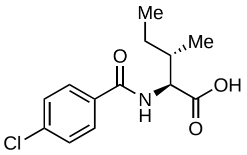

(4-Chlorobenzoyl)-L-isoleucine (6) was prepared according to general synthesis method D using 4-chlorobenzoic acid (63 mg, 0.4 mmol, 1 eq.) and L-isoleucine methyl ester hydrochloride (87 mg, 0.48 mmol, 1.2 eq.). The product was obtained as a white solid (93 mg, 0.34 mmol, 85%).

^{1}H NMR (400 MHz, DMSO) *δ* 12.67 (s, 1H), 8.59 (d, *J* = 8.0 Hz, 1H), 7.96 (d, *J* = 8.5 Hz, 2H), 7.59 (d, *J* = 8.4 Hz, 2H), 4.38 (t, *J* = 7.5 Hz, 1H), 2.11 – 1.88 (m, 1H), 1.64 – 1.23 (m, 2H), 0.98 (d, *J* = 6.8 Hz, 3H), 0.92 (t, *J* = 7.4 Hz, 3H).

^{13}C NMR (101 MHz, DMSO) *δ* 173.1, 165.8, 136.2, 132.8, 129.6, 128.3, 57.3, 35.7, 25.2, 15.7, 11.1.

HR-MS: calc. for $[M+Na]^+$: 292.0711, observed: 292.0715 (-1.37 ppm)

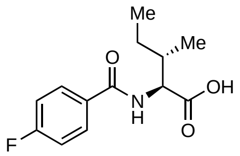

(4-Fluorobenzoyl)-L-isoleucine (7) was prepared according to general synthesis method E using 4-fluorobenzoic acid (140 mg, 1 mmol, 1 eq.) and L-isoleucine (157 mg, 1.2 mmol, 1.2 eq.). The product was obtained as a white solid (170 mg, 0.67 mmol, 67%).

^{1}H NMR (400 MHz, DMSO) δ 12.68 (s, 1H), 8.46 (d, J = 8.0 Hz, 1H), 7.99 – 7.93 (m, 2H), 7.32 – 7.26 (m, 2H), 4.42 – 4.22 (m, 1H), 2.02 – 1.87 (m, 1H), 1.56 – 1.21 (m, 2H), 0.93 (d, J = 6.8 Hz, 3H), 0.86 (t, J = 7.4 Hz, 3H).

^{13}C NMR (101 MHz, DMSO) δ 173.6, 166.2, 166.9 – 162.6 (m), 132.6 (d, J_{CF} = 9.4 Hz), 130.8 (d, J_{CF} = 9.0 Hz), 115.5 (d, J_{CF} = 21.7 Hz), 57.8, 36.2, 25.6, 16.1, 11.5.

HR-MS: calc. for $[M+H]^+$: 254.1187, observed: 254.1193 (-2.36 ppm)

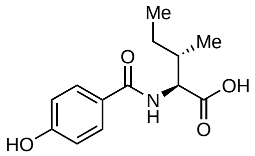

(4-Hydroxybenzoyl)-L-isoleucine (8) was prepared according to general synthesis method D using 4-hydroxybenzoic acid (55 mg, 0.4 mmol, 1 eq.) and L-isoleucine methyl ester hydrochloride (87 mg, 0.48 mmol, 1.2 eq.). The product was obtained as a white solid (60 mg, 0.24 mmol, 60%).

^{1}H NMR (400 MHz, DMSO) δ 12.37 (s, 1H), 9.97 (s, 1H), 8.10 (d, J = 8.1 Hz, 1H), 7.76 (d, J = 8.7 Hz, 2H), 6.79 (d, J = 8.6 Hz, 2H), 4.29 (t, J = 7.6 Hz, 1H), 1.93 (dd, J = 7.8, 3.5 Hz, 1H), 1.56 – 1.21 (m, 3H), 0.91 (d, J = 6.8 Hz, 3H), 0.86 (t, J = 7.4 Hz, 3H).

^{13}C NMR (101 MHz, DMSO) δ 173.4, 166.3, 160.2, 129.6, 124.8, 114.7, 57.1, 35.7, 25.2, 15.7, 11.0.

HR-MS: calc. for $[M-H]^-$: 250.1084, observed: 250.1084 (-0.3 ppm)

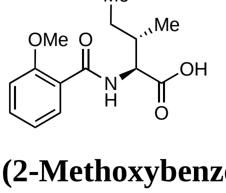

(2-Methoxybenzoyl)-L-isoleucine (9) was prepared according to general synthesis method C using 2-methoxybenzoic acid (61 mg, 0.4 mmol, 1 eq.) and L-Isoleucine methyl ester

hydrochloride (95 mg, 0.52 mmol, 1.3 eq.). The product was obtained as a white solid (64 mg, 0.24 mmol, 60%).

^{1}H NMR (400 MHz, DMSO) *δ* 8.39 (d, *J* = 8.1 Hz, 1H), 7.85 (dd, *J* = 7.7, 1.8 Hz, 1H), 7.52 (ddd, *J* = 8.3, 7.3, 1.9 Hz, 1H), 7.19 (dd, *J* = 8.5, 1.0 Hz, 1H), 7.07 (td, *J* = 7.5, 1.0 Hz, 1H), 4.46 (dd, *J* = 8.1, 5.0 Hz, 1H), 3.94 (s, 3H), 1.56 – 1.20 (m, 2H), 0.92 (m, 6H).

^{13}C NMR (101 MHz, DMSO) *δ* 172.8, 172.0, 164.3, 157.3, 132.9, 130.8, 121.7, 120.8, 112.4, 56.5, 56.3, 36.7, 24.9, 21.1, 15.7, 11.5.

HR-MS: calc. for $[M+Na]^+$ = 288.1206, observed: 288.1202 (-1.3 ppm)

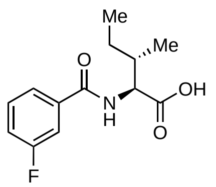

(3-Fluorobenzoyl)-L-isoleucine (10) was prepared according to general synthesis method D using 3-fluorobenzoic acid (56 mg, 0.4 mmol, 1 eq.) and L-isoleucine methyl ester hydrochloride (87 mg, 0.48 mmol, 1.2 eq.). The product was obtained as a white solid (64 mg, 0.094 mmol, 63%).

^{1}H NMR (400 MHz, DMSO) δ 12.62 (br s, 1H), 8.53 (d, *J* = 8.13 Hz, 1H), 7.76 – 7.68 (m, 2H), 7.77 – 7.50 (m, 1H), 7.43 – 7.35 (m, 1H), 4.35 – 4.30 (m, 1H), 1.98 – 1.46 (m, 2H), 1.31 – 1.22 (m, 1H), 0.96 – 0.82 (m, 6H).

^{13}C NMR (101 MHz, DMSO) δ 173.5, 165.9, 165.9, 163.6, 161.1, 136.9, 136.8, 130.9, 130.8, 124.3, 124.3, 118.8, 118.6, 115.0, 114.7, 57.8, 36.1, 25.6, 16.1, 11.5.

HR-MS: calc. for $[M+H]^+$: 254.1187, observed: 254.1191 (-1.57 ppm)

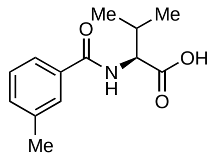

(3-Methylbenzoyl)-L-valine (11) was prepared according to general synthesis method D using of 3-toluic acid (69 mg, 0.4 mmol, 1 eq.) and L-isoleucine methyl ester hydrochloride (87 mg, 0.48 mmol, 1.2 eq.). The product was obtained as a white solid (90 mg, 0.32 mmol, 79%).

^{1}H NMR (400 MHz, DMSO) δ 12.53 (br s, 1H), 8.33 (d, *J* = 8.10 Hz), 7.71 (s, 1H), 7.68 – 7.64 (m, 1H), 7.37 - 7.31 (m, 2H), 4.28 (dd, *J* = 8.06, 7.10 Hz 1H), 2.36 (s, 3H), 2.23 – 2.13 (m, 1H), 0.96 (t, *J* = 6.84 Hz, 6H)

^{13}C NMR (101 MHz, DMSO) δ 173.6, 167.4, 137.9, 134.6, 132.3, 128.5, 125.3, 58.8, 30.0, 21.4, 19.8, 19.3.

HR-MS: calc. for $[M+Na]^+$ = 258.1101, observed: 258.1106 (1.9 ppm)

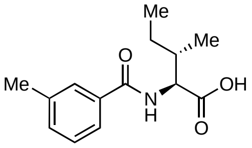

(3-Methylbenzoyl)-L-isoleucine (12) was prepared according to general synthesis method E using 3-toluic acid (136 mg, 1 mmol, 1 eq.) and L-isoleucine (157 mg, 1.2 mmol, 1.2 eq.). The product was obtained as a white solid (173 mg, 0.69 mmol, 69%).

^{1}H NMR (400 MHz, DMSO) δ ^{1}H NMR (400 MHz, DMSO) δ 12.59 (s, 1H), 8.34 (d, *J* = 8.1 Hz, 1H), 7.70 (s, 1H), 7.66 (m, 1H), 7.39 – 7.30 (m, 2H), 4.33 (dd, *J* = 8.1, 7.1 Hz, 1H), 2.36 (s, 3H), 1.94 (m, 1H), 1.55 – 1.23 (m, 2H), 0.93 (d, *J* = 6.8 Hz, 3H), 0.87 (t, *J* = 7.4 Hz, 3H).

^{13}C NMR (101 MHz, DMSO) δ 173.2, 166.8, 137.4, 134.1, 131.8, 128.1 (2C), 124.8, 57.2, 35.7, 25.1, 20.9, 15.7, 11.0.

HR-MS: calc. for $[M+H]^+$: 254.1187, observed: 254.1193 (-2.36 ppm)

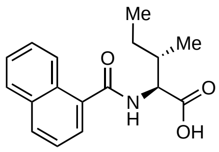

(1-Naphthoyl)-L-isoleucine (13) was prepared according to the general synthesis method C using 1-napthoic acid (69 mg, 0.4 mmol) and L-isoleucine methyl ester hydrochloride (87 mg, 0.48 mmol, 1.2 eq.). The product was obtained as a white solid (157 mg, 0.55 mmol, 63%).

^{1}H NMR (400 MHz, DMSO) *δ* 12.56 (s, 1H), 8.71 (d, *J* = 7.9 Hz, 1H), 8.22 – 8.14 (m, 1H), 8.04 – 7.94 (m, 2H), 7.61 – 7.52 (m, 4H), 4.48 – 4.38 (m, 1H), 1.97 – 1.91 (m, 1H), 1.57 – 1.27 (m, 2H), 0.98 (d, *J* = 6.8 Hz, 3H), 0.89 (t, *J* = 7.4 Hz, 3H).

^{13}C NMR (101 MHz, DMSO) *δ* 173.1, 169.0, 134.6, 133.0, 129.9, 129.6, 128.1, 126.6, 126.2, 125.4, 125.3, 125.0, 57.2, 35.8, 25.0, 15.7, 11.2.

HR-MS: calc. for $[M+Na]^+$ = 308.1257, observed: 308.1256 (-0.3 ppm).

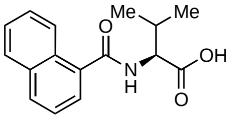

(1-Naphthoyl)-L-valine (14) was prepared according to general synthesis method D using 1-napthoic acid (69 mg, 0.4 mmol, 1 eq.) and L-isoleucine methyl ester hydrochloride (87 mg, 0.48 mmol, 1.2 eq.). The product was obtained as an off-white solid (75 mg, 0.28 mmol, 69%).

^{1}H NMR (400 MHz, DMSO) δ 12.60 (br s, 1H), 8.69 (d, *J* = 8.19 Hz), 8.21 – 8.14 (m, 1H), 8.04 – 7.94 (m, 2H), 7.61 – 7.52 (m, 4H), 4.40 (dd, *J* = 8.15, 6.33 Hz, 1H), 2.25 – 2.14 (m, 1H), 1.00 (dd, *J* = 10.24, 6.99 Hz, 6H).

^{13}C NMR (101 MHz, DMSO) δ 173.6, 169.6, 135.1, 133.5, 130.3, 130.1, 128.6, 127.1, 126.6, 125.9, 125.8, 125.4, 58.6, 29.9, 19.8, 18.9.

HR-MS: calc. for $[M+Na]^+$ = 294.1101, observed: 294.1098 (-0.9 ppm)

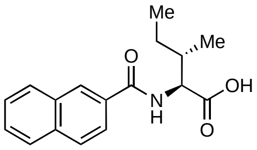

(2-Naphthoyl)-L-isoleucine (15)

Prepared according to general synthesis method D using 2-napthoic acid (69 mg, 0.4 mmol, 1 eq.) and L-Isoleucine methyl ester hydrochloride (87 mg, 0.48 mmol, 1.2 eq.).

Yield: The product was obtained as a white solid (90 mg, 0.32 mmol, 79%).

^{1}H NMR (400 MHz, DMSO) δ 12.63 (br s, 1H), 8.58 (d, *J* = 8.13 Hz), 8.51 (s, 1H), 8.05 - 8.03 (m, 1H), 8.00 - 7.94 (m, 3H), 7.64 – 7.58 (m, 2H), 4.41 (t, *J* = 4.41 Hz 1H), 2.01 (m, 1H), 1.60 - 1.29 (m, 2H), 0.98 - 0.88 (m, 6H).

^{13}C NMR (101 MHz, DMSO) δ 173.2, 166.8, 134.2, 132.1, 131.4, 128.9, 127.8, 127.7, 127.6, 126.7, 124.5, 57.3, 35.8, 25.2, 15.7, 11.1.

HR-MS: calc. for $[M+Na]^+$: 308.1257, observed: 308.1261 (-1.30 ppm)

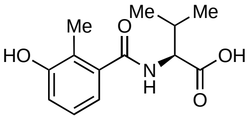

(3-Hydroxy-2-methylbenzoyl)-L-valine (16) was prepared according to general procedure D on a 0.4 mmol scale using 3-hydroxy-2-methylbenzoic acid acid (61 mg, 0.4 mmol, 1 eq.)

and L-valine methyl ester hydrochloride (80 mg, 0.48 mmol, 1.2 eq.). The product was obtained as an off-white solid (58 mg, 0.23 mmol, 58%)

^{1}H NMR (400 MHz, DMSO) δ 12.56 (br s, 1H), 9.44 (s, 1H) 8.28 (d, *J* = 8.22 Hz, 1H), 7.02 (t, *J* = 7.93 Hz, 1H) 6.84 (dd, *J* = 8.13, 0.80 Hz, 1H), 6.72 (dd, *J* = 7.62, 0.85 Hz, 1H), 4.25 (dd, *J* = 8.16, 6.61 Hz, 1H), 2.12 (hept, 6.75 Hz, 1H), 2.11 (s, 3H) 0.94 (t, *J* = 6.56 Hz, 6H).

^{13}C NMR (101 MHz, DMSO) δ 173.1, 169.8, 155.5, 138.9, 125.9, 121.5, 117.8, 115.3, 57.9, 29.4, 19.3, 18.4, 12.6.

HR-MS: calc. for $[M+Na]^{+}$: 274.105, observed: 274.1054 (-1.46 ppm)

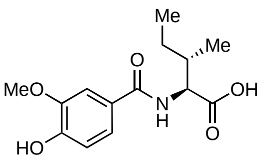

(4-Hydroxy-3-methoxybenzoyl)-L-isoleucine (17) was prepared according to general synthesis method D using vanillic acid (67 mg, 0.4 mmol, 1 eq.) in and L-isoleucine methyl ester hydrochloride (87 mg, 0.48 mmol, 1.2 eq.). The product was obtained as a white solid (43 mg, 0.15 mmol, 38%).

^{1}H NMR (400 MHz, DMSO) *δ* 12.47 (s, 1H), 9.55 (s, 1H), 8.16 (d, *J* = 8.1 Hz, 1H), 7.44 (s, 1H), 7.41 (d, *J* = 8.2 Hz, 1H), 6.81 (dd, *J* = 8.2, 1.4 Hz, 1H), 4.31 (t, *J* = 7.6 Hz, 1H), 3.82 (d, *J* = 1.3 Hz, 3H), 1.99 – 1.91 (m, 1H), 1.54 – 1.23 (m, 2H), 0.99 – 0.89 (m, 3H), 0.86 (t, *J* = 7.4 Hz, 3H).

^{13}C NMR (101 MHz, DMSO) *δ* 173.4, 166.3, 149.6, 147.1, 125.0, 121.2, 114.7, 111.7, 57.1, 55.7, 35.7, 25.2, 15.7, 11.0.

HR-MS: calc. for $[M+Na]^{+}$: 304.1155, observed: 304.1157 (-0.66 ppm)

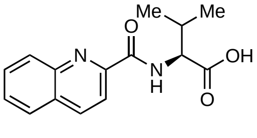

(Quinoline-2-carbonyl)-L-valine (18) was prepared according to general synthesis method E using quinoline-2-carboxylic acid (433 mg, 2.5 mmol, 1 eq.) and L-valine methyl ester hydrochloride (419 mg, 2.5 mmol, 1 eq.). The product was obtained as an off-white solid (355 mg, 1.3 mmol, 33%).

^{1}H NMR (400 MHz, DMSO) δ 13.07 (br s, 1H), 8.67 (d, *J* = 8.90 Hz, 1H), 8.62 – 8.58 (m, 1H), 8.10 (dd, *J* = 8.31, 1.02 Hz, 1H), 7.89 (ddd, *J* = 8.40, 6.88, 1.57, 1H), 7.73 (ddd, *J* = 8.18, 6.97, 1.19, 1H), 4.48 (dd, *J* = 8.86, 5.48, 1H), 2.29 (m, 1H), 0.98 (dd, *J* = 6.88, 2.60, 6H).

^{13}C NMR (101 MHz, DMSO) δ 173.1, 164.1, 149.7, 146.4, 138.7, 131.2, 129.8, 129.5, 128.8, 128.6, 119.0, 57.7, 30.9, 19.6.

HR-MS: calc. for $[M-H]^-$: 271.1088, observed: 271.1086 (-0.9 ppm)

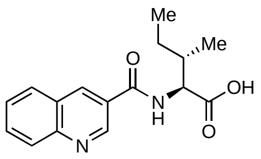

(Quinoline-3-carbonyl)-L-isoleucine (19) was prepared according to general synthesis method D using quinoline-3-carboxylic acid (69 mg, 0.4 mmol, 1 eq.) and L-isoleucine methyl ester hydrochloride (87 mg, 0.48 mmol, 1.2 eq.). The product was obtained as a white solid (26.8 mg, 0.094 mmol, 23%).

^{1}H NMR (400 MHz, DMSO) δ 12.69 (br s, 1H), 9.27 (d, *J* = 2.19 Hz, 1H), 8.89 (d, *J* = 1.88 Hz, 1H), 8.83 (d, *J* = 1.88 Hz, 1H), 8.11 (t, *J* = 9.31 Hz, 2H), 7.90 – 7.85 (m, 1H), 7.70 (t, *J* = 7.60 Hz, 1H), 4.43 (t, *J* = 7.48 Hz, 1H), 2.02 – 1.96 (m, 1H), 1.61 – 1.30 (m, 2H), 0.87 – 1.00 (m, 6H).

^{13}C NMR (101 MHz, DMSO) δ 173.4, 166.0, 149.6, 148.9, 136.3, 131.7, 129.6, 129.2, 127.9, 127.2, 126.9, 57.8, 36.4, 25.6, 16.2, 11.6.

HR-MS: calc. for $[M+H]^+$ = 287.1390, observed: 287.1391 (0.2 ppm)

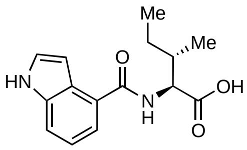

(1*H*-Indole-4-carbonyl)-L-isoleucine (20) was prepared according to general synthesis method D using indole 4-carboxylic acid (65 mg, 0.4 mmol, 1 eq.) and L-isoleucine methyl ester hydrochloride (87 mg, 0.48 mmol, 1.2 eq.). The product was obtained as a white solid (93 mg, 0.335 mmol, 84%).

^{1}H NMR (400 MHz, DMSO) *δ* 12.59 (s, 1H), 11.31 (s, 1H), 8.04 (d, *J* = 8.1 Hz, 1H), 7.55 (d, *J* = 8.0 Hz, 1H), 7.50 – 7.40 (m, 2H), 7.15 (t, *J* = 7.7 Hz, 1H), 6.81 (s, 1H), 4.42 (t, *J* = 7.3 Hz, 1H), 1.94 (d, *J* = 4.4 Hz, 1H), 1.58 – 1.23 (m, 2H), 0.96 (d, *J* = 6.9 Hz, 3H), 0.89 (t, *J* = 7.4 Hz, 3H).

^{13}C NMR (101 MHz, DMSO) *δ* 173.4, 168.0, 136.5, 126.6, 126.2, 125.8, 120.1, 119.0, 114.3, 101.4, 56.9, 36.0, 25.2, 15.7, 11.2.

HR-MS: calc. for $[M+Na]^+$: 297.121, observed: 297.1215 (-1.68 ppm)

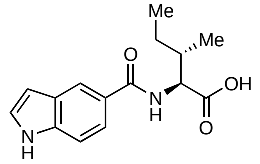

(1*H*-Indole-5-carbonyl)-L-isoleucine (21) was prepared according to general synthesis method D using indole-5-carboxylic acid (65 mg, 0.4 mmol, 1 eq.) and added L-Isoleucine methyl ester hydrochloride (87 mg, 0.48 mmol, 1.2 eq.). The product was obtained as a pale brown solid (90 mg, 0.33 mmol, 82%).

^{1}H NMR (400 MHz, DMSO) δ 12.51 (s, 1H), 11.31 (s, 1H), 8.18 (d, *J* = 7.5 Hz, 2H), 7.66 (d, *J* = 8.5 Hz, 1H), 7.48 – 7.36 (m, 2H), 6.54 (s, 1H), 4.34 (d, *J* = 7.6 Hz, 1H), 1.95 (dd, *J* = 7.4, 4.1 Hz, 1H), 1.59 – 1.23 (m, 2H), 0.95 (d, *J* = 6.8 Hz, 3H), 0.88 (t, *J* = 7.4 Hz, 3H).

^{13}C NMR (101 MHz, DMSO) δ 173.5, 167.7, 137.5, 126.9, 126.6, 125.1, 120.9, 120.3, 110.8, 102.1, 57.2, 35.8, 25.2, 15.7, 11.1.

HR-MS: calc. for $[M-H]^-$: 273.1244, observed: 273.124 (-1.6 ppm)

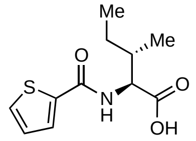

(Thiophene-2-carbonyl)-L-isoleucine (22) was prepared according to general synthesis method D using thiophene-2-carboxylic acid (52 mg, 0.4 mmol, 1 eq.) and L-isoleucine methyl ester hydrochloride (87 mg, 0.48 mmol, 1.2 eq.). The product was obtained as a colourless sticky solid (80 mg, 0.33 mmol, 83%).

^{1}H NMR (400 MHz, CD_3OD) δ 7.88 – 7.74 (m, 1H), 7.65 (dd, *J* = 4.9, 1.1 Hz, 1H), 7.12 (dd, *J* = 5.0, 3.8 Hz, 1H), 4.52 (d, *J* = 6.5 Hz, 1H), 2.06 – 1.97 (m, 1H), 1.66 – 1.28 (m, 2H), 1.01 (d, *J* = 6.8 Hz, 3H), 0.96 (t, *J* = 7.4 Hz, 3H).

^{13}C NMR (101 MHz, CD_3OD) δ 174.9, 164.4, 139.7, 132.0, 130.1, 128.8, 58.7, 38.1, 26.6, 16.1, 11.6.

HR-MS: calc. for $[M+Na]^+$: 398.0224, observed: 398.0227 (-0.75 ppm)

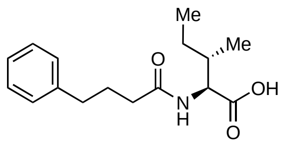

(4-Phenylbutanoyl)-L-isoleucine (23) was prepared according to general synthesis method D using 4-phenylbutanoic acid (66 mg, 0.4 mmol, 1 eq.) and L-isoleucine methyl ester hydrochloride (87 mg, 0.48 mmol, 1.2 eq.). The product was obtained as a white solid (81 mg, 0.29 mmol, 73%).

^{1}H NMR (400 MHz, DMSO) δ 12.49 (s, 1H), 7.96 (d, J = 8.4 Hz, 1H), 7.27 (t, J = 7.5 Hz, 2H), 7.18 (d, J = 7.2 Hz, 3H), 4.18 (dd, J = 8.4, 6.1 Hz, 1H), 2.55 (t, J = 7.7 Hz, 2H), 2.24 – 2.09 (m, 2H), 1.86 – 1.68 (m, 3H), 1.43 – 1.14 (m, 2H), 0.84 (m, 6H).

^{13}C NMR (101 MHz, DMSO) δ 173.2, 172.2, 141.9, 128.3, 128.3, 125.7, 56.2, 36.2, 34.6, 34.5, 27.3, 24.7, 15.6, 11.2.

HR-MS: calc. for $[M+Na]^+$: 300.157, observed: 300.1577 (-2.33 ppm)

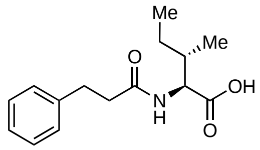

(3-Phenylpropanoyl)-L-isoleucine (24) was prepared according to general synthesis method D using of 3-phenylpropanoic acid (60 mg, 0.4 mmol, 1 eq.) and L-isoleucine methyl ester hydrochloride (87 mg, 0.48 mmol, 1.2 eq.). The product was obtained as a white solid (91 mg, 0.345 mmol, 86%).

^{1}H NMR (400 MHz, DMSO) δ 12.52 (s, 1H), 7.98 (d, J = 8.5 Hz, 1H), 7.33 – 7.06 (m, 5H), 4.25 – 4.10 (m, 1H), 2.80 (t, J = 7.7 Hz, 2H), 2.43 (m, 2H), 1.70 (m, 1H), 1.38 – 1.07 (m, 2H), 0.81 (t, J = 6.7 Hz, 6H).

^{13}C NMR (101 MHz, DMSO) δ 173.2, 171.5, 141.3, 128.2, 128.2, 125.8, 56.1, 36.5, 36.3, 31.1, 24.6, 15.5, 11.2.

HR-MS: calc. for $[M+Na]^+$: 286.1414, observed: 286.142 (-2.10 ppm)

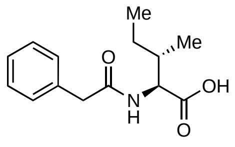

(2-Phenylacetyl)-L-isoleucine (25) was prepared according to general synthesis method C using phenylacetic acid (136 mg, 1 mmol, 1 eq.) and L-isoleucine methyl ester hydrochloride

(236 mg, 1.3 mmol, 1.3 eq.). The product was obtained as a white solid (185 mg, 0.74 mmol, 74%).

^{1}H NMR (400 MHz, DMSO) δ 7.45 – 7.07 (m, 5H), 4.38 (d, *J* = 5.6 Hz, 1H), 3.59 (d, *J* = 2.8 Hz, 2H), 1.97 – 1.81 (m, 1H), 1.58 – 1.14 (m, 2H), 0.91 (m, 6H).

^{13}C NMR (101 MHz, DMSO) δ 174.7, 174.1, 137.0, 130.2, 129.5, 127.9, 58.2, 43.4, 38.4, 26.2, 16.0, 11.8.

HR-MS: calc. for $[M+Na]^+$: 272.1257, observed: 272.1252 (1.84 ppm)

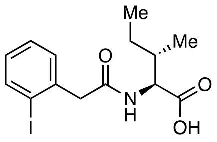

(2-(2-Iodophenyl)acetyl)-L-isoleucine (26) was prepared according to general synthesis method D using 2-(2-iodophenyl)acetic acid (105 mg, 0.4 mmol, 1 eq.) and L-isoleucine methyl ester hydrochloride (87 mg, 0.48 mmol, 1.2 eq.). The product was obtained as a white solid (118 mg, 0.31 mmol, 79%).

^{1}H NMR (400 MHz, DMSO) δ 12.59 (s, 1H), 8.21 (d, *J* = 8.4 Hz, 1H), 7.92 – 7.73 (m, 1H), 7.32 (m, 2H), 6.98 (m, 1H), 4.22 (dd, *J* = 8.4, 5.9 Hz, 1H), 3.76 – 3.58 (m, 2H), 1.87 – 1.73 (m, 1H), 1.47 – 1.18 (m, 2H), 0.94 – 0.78 (m, 6H).

^{13}C NMR (101 MHz, DMSO) δ 173.0, 169.1, 139.6, 138.8, 130.7, 128.5, 128.2, 101.6, 56.3, 46.5, 36.4, 24.7, 15.7, 11.3.

HR-MS: calc. for $[M+Na]^+$: 398.0224, observed: 398.0227 (-0.75 ppm)

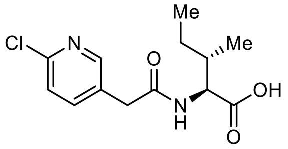

(2-(6-Chloropyridin-3-yl)acetyl)-L-isoleucine (27) was prepared according to general synthesis method D using 2-(6-chloropyridin-3-yl)acetic acid (69 mg, 0.4 mmol, 1 eq.) and L-isoleucine methyl ester hydrochloride (87 mg, 0.48 mmol, 1.2 eq.). The product was obtained as an off-white solid (41 mg, 0.145 mmol, 36%).

^{1}H NMR (400 MHz, DMSO) δ 12.60 (br s, 1H), 8.36 (d, *J* = 8.73 Hz, 1H), 8.28 (d, *J* = 2.32 Hz, 1H), 7.73 (dd, *J* = 8.39, 2.35 Hz, 1H), 7.45 (d, *J* = 8.39 Hz, 1H), 4.18 (t, *J* = 7.11 Hz, 1H), 3.57 (m, 2H),1.83 – 1.72 (m, 1H), 1.45 – 1.12 (m, 2H), 0.83 (m, 6H)

^{13}C NMR (101 MHz, DMSO) δ 169.9, 150.5, 148.8, 140.7, 132.3, 124.3, 38.4, 36.9, 25.2, 16.1, 11.7.

HR-MS: calc. for $[M+Na]^+$: 307.082, observed: 307.0825 (-1.63 ppm)

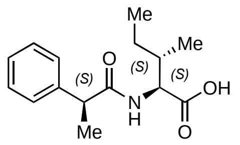

((*S*)-2-Phenylpropanoyl)-L-isoleucine (28) was prepared according to general synthesis method C using (*S*)-2-phenyl-propionic acid (60 mg, 0.4 mmol, 1 eq.) and L-isoleucine methyl ester hydrochloride (94 mg, 0.42 mmol, 1.3 eq.). The product was obtained as a white solid (95 mg, 0.33 mmol, 83%).

^{1}H NMR (400 MHz, $CDCl_3$) *δ* 7.38 – 7.28 (m, 5H), 5.90 (d, *J* = 8.3 Hz, 1H), 4.52 (dd, *J* = 8.3, 4.9 Hz, 1H), 3.65 (q, *J* = 7.2 Hz, 1H), 1.94 – 1.81 (m, 1H), 1.54 (d, *J* = 7.2 Hz, 3H), 1.36 – 0.96 (m, 2H), 0.86 (m, 6H).

^{13}C NMR (101 MHz, $CDCl_3$) *δ* 175.7, 174.9, 140.6, 129.1, 127.8, 127.6, 56.7, 47.2, 37.5, 25.1, 18.4, 15.5, 11.6.

HR-MS: calc. for $[M+Na]^+$ = 286.1414, observed: 286.1412 (-0.6 ppm)

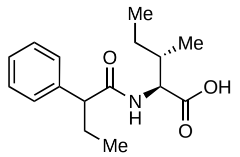

(2-Phenylbutanoyl)-L-isoleucine (29) was prepared according to general synthesis method D using 2-phenylbutyric acid (137 mg, 0.8 mmol, 1 eq.) and L-isoleucine methyl ester hydrochloride (194 mg, 0.88, 1.2 eq.). The product was isolated as a mixture of diastereoisomers. The product was obtained as a white solid (138 mg, 0.49 mmol, 62%).

^{1}H NMR (400 MHz, DMSO) δ 12.49 (s, 1H), 8.18, 8.14* (d, *J* = 8.66 Hz, 1H), 7.34 – 7.30 (m, 2H), 7.27 (td, *J* = 7.27, 1.25 Hz, 2H), 7.21 – 7.16 (m, 1H) 4.22, 4.12* (dd, *J* = 8.36, 6.27 Hz, 1H), 3.59 - 3.53 (m, 1H), 1.97 – 1.88 (m, 1H) 1.82 – 1.75, 1.74 – 1.66* (m, 1H), 1.65 – 1.54 (m, 1H), 1.45 – 1.37, 1.11 - 0.99* (m, 1H), 1.32 – 1.15 (m, 1H), 0.88 – 0.84 (m, 3H), 0.81 (t, *J* = 7.01 Hz, 3H), 0.75 – 0.68 (m, 3H) (*diastereoisomer peak, approx. 1:1 ratio of isomers)

^{13}C NMR (101 MHz, DMSO) δ 173.7, 173.5, 173.1, 141.6, 141.4*, 128.5, 128.2*, 128.0, 126.9, 56.6, 56.5*, 52.8, 52.7, 36.9, 36.7*, 27.1, 26.4, 25.1, 25.0*, 16.1, 15.9*, 12.7, 12.6*, 11.6. (*diastereoisomer peak, approx. 1:1 ratio of isomers)

HR-MS: calc. for $[M+Na]^+$ = 300.1570, observed: 300.1568 (-0.7 ppm)

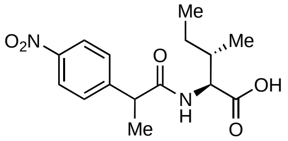

(2-(4-Nitrophenyl)propanoyl)-L-isoleucine (30) was prepared according to general synthesis method D using 2-(4-nitrophenylpropionic) acid (78 mg, 0.4 mmol, 1 eq.) and L-isoleucine methyl ester hydrochloride (87 mg, 0.48 mmol, 1.2 eq.). The product was isolated as a mixture of diastereoisomers. The product was obtained as a sticky pale-yellow solid (87 mg, 0.28 mmol, 71%).

^{1}H NMR (400 MHz, DMSO) 12.60 (s, 1H), 8.31 (d, *J* = 8.14, 1H), 8.20 – 8.15 (m, 2H), 7.65 – 7.57 (m, 2H), 4.21 – 4.13 (m, 1H), 4.04 - 3.96 (m, 1H), 1.83 – 1.66 (m, 1H), 1.40 – 1.31 (m, 3H), 1.31 – 1.14 (m, 2H), 0.90 – 0.82 (m, 2H), 0.77 – 0.66 (m, 4H)

^{13}C NMR (101 MHz, DMSO) δ 173.5, 173.3, 172.8, 150.6, 150.3, 146.7, 129.2, 129.1, 123.8, 123.8, 56.8, 56.6, 44.8, 36.9, 25.2, 25.0, 19.2, 18.5, 16.0, 16.0, 11.7, 11.6.

HR-MS: calc. for $[M+Na]^+$ = 331.1265, observed: 331.1261 (-1.1 ppm)

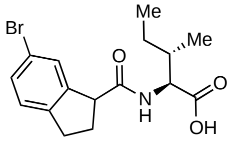

(6-Bromo-2,3-dihydro-1H-indene-1-carbonyl)-L-isoleucine (31) was prepared according to general synthesis method D using 6-bromo-2,3-dihydro-1*H*-indene-1-carboxylic acid (97 mg, 0.4 mmol, 1 equiv) L-isoleucine methyl ester hydrochloride (87 mg, 0.48, 1.2 eq.). The product was isolated as a mixture of diastereoisomers. The product was obtained as a white solid (69 mg, 0.195 mmol, 49%).

^{1}H NMR (400 MHz, DMSO) δ 12.66 (br s, 1H), 8.42#, 8.38* (d, *J* = 8.51 Hz, 1H), 7.52#, 7.40* (s, 1H), 7.35 – 7.33#, 7.33 – 7.30* (m, 1H), 7.21 – 7.18*, 7.18 – 7.16# (d, *J* = 2.73 Hz, 1H), 4.25#, 4.21* (dd, *J* = 8.39#, 8.48*,6.02#, 6.34* Hz, 1H), 4.11 – 4.04 (m, 1H), 3.01 – 2.87 (m, 1H), 2.84 – 2.73 (m, 1H), 2.33 – 2.21 (m, 1H), 2.21 – 2.11 (m, 1H), 1.89 – 1.78 (m, 1H), 1.52 – 1.39 (m, 1H), 1.31 – 1.20 (m, 1H), 0.92 – 0.83 (m, 6H) (#minor diastereoisomer, *major diastereoisomer)

^{13}C NMR (101 MHz, DMSO) δ 173.84#, 173.6*, 173.0*, 172.9#, 146.5#, 146.6*, 144.0*, 143.8#, 130.1*, 130.0#, 127.6#, 127.5*, 126.9*, 126.7#, 119.4#, 119.3*, 56.9#, 56.8*, 50.6*, 50.4#, 36.9*, 36.7#, 31.8*, 31.6#, 28.9#, 28.7*, 25.3#, 25.2*, 16.2#, 16.1*, 11.8#, 11.7*. (#minor diastereoisomer, *major diastereoisomer)

HR-MS: calc. for $[M-H]^-$: 352.0554, observed: 352.0558 (1.1 ppm)

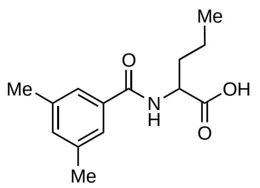

2-(3,5-dimethylbenzamido)pentanoic acid (32) was prepared according to general synthesis method E using 3,5-dimethylbenzoic acid (75 mg, 0.5 mmol, 1 eq.) and DL-norvaline (64 mg, 0.55 mmol, 1.1 eq.). The product was obtained as a white solid (54 mg, 0.22 mmol, 43%).

^{1}H NMR (400 MHz, DMSO) δ 8.41 (d, *J* = 7.7 Hz, 1H), 7.49 (s, 2H), 7.16 (s, 1H), 4.36 (q, *J* = 7.5 Hz, 1H), 2.32 (s, 6H), 1.76 (q, *J* = 7.5 Hz, 2H), 1.47 – 1.29 (m, 2H), 0.89 (t, *J* = 7.3 Hz, 3H).

^{13}C NMR (101 MHz, DMSO) δ 174.0, 166.7, 137.3, 134.1, 132.5, 125.2, 52.3, 32.7, 20.8, 19.0, 13.5.

HR-MS: calc. for $[M+Na]^+$ = 272.1257, observed: 272.1259 (0.8 ppm)

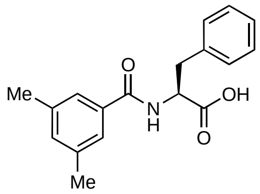

(3,5-Dimethylbenzoyl)-L-phenylalanine (33) was prepared according to general synthesis method E using 3,5-dimethylbenzoic acid (150 mg, 1 mmol, 1 eq.) and L-phenylalanine (198 mg, 1.2 mmol, 1.2 eq.). The product was obtained as a white solid (163 mg, 0.55 mmol, 55%).

^{1}H NMR (400 MHz, DMSO) *δ* 12.72 (s, 1H), 8.56 (d, *J* = 8.2 Hz, 1H), 7.40 (s, 2H), 7.32 – 7.23 (m, 4H), 7.16 (d, *J* = 14.3 Hz, 2H), 4.61 (m, 1H), 3.21 – 3.02 (m, 2H), 2.30 (s, 6H).

^{13}C NMR (101 MHz, DMSO) *δ* 173.2, 166.6, 138.2, 137.3, 134.0, 132.6, 129.0, 128.2, 126.3, 125.1, 54.1, 36.3, 20.8.

HR-MS: calc. for $[M+Na]^+$: 320.1257, observed: 320.1265 (-2.50 ppm)

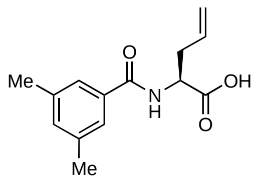

(*S*)-2-(3,5-Dimethylbenzamido)pent-4-enoic acid (34) was prepared based on the following procedure. To a solution of **43** (114 mg, 0.5 mmol, 1 eq.) was added 6 M HCl (2 mL) and the suspension was stirred at 100 °C for 18 h. After that time, the reaction was carefully basified with 10 M NaOH, the aqueous phase was washed with Et_2O (15 mL) and re-acidified with 6 M HCl. The aqueous layer was extracted with EtOAc (3x30 mL), the combined organic layers were dried over $MgSO_4$ and concentrated under reduced pressure. Silica gel column chromatography (elute: DCM / MeOH) afforded the desired product. The product was obtained as a white solid (63 mg, 0.25 mmol, 50%).

^{1}H NMR (400 MHz, DMSO) δ 8.44 (d, *J* = 7.9 Hz, 1H), 7.47 (s, 2H), 7.16 (s, 1H), 5.82 (m, 1H), 5.19 – 5.07 (m, 1H), 5.03 (dd, *J* = 10.2, 2.1 Hz, 1H), 4.48 – 4.36 (m, 1H), 2.64 – 2.51 (m, 2H), 2.31 (s, 6H).

^{13}C NMR (101 MHz, DMSO) δ 173.2, 166.5, 137.4, 134.7, 134.1, 132.6, 125.1, 117.3, 52.4, 35.1, 20.8.

HR-MS: calc. for $[M+Na]^+$: 270.1101, observed: 270.1103 (-0.74 ppm)

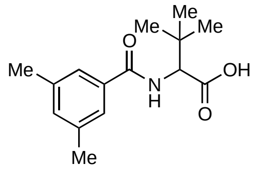

2-(3,5-Dimethylbenzamido)-3,3-dimethylbutanoic acid (35) was prepared according to general synthesis method D using 3,5-dimethylbenzoic acid (150 mg, 1 mmol, 1.2 eq.) and *tert*-leucine methyl ester hydrochloride (134 mg, 0.833 mmol, 1 eq.). The product was obtained as a pale-yellow solid (167 mg, 0.53 mmol, 53%).

^{1}H NMR (400 MHz, DMSO) 12.64 (s, 1H), 8.01 (d, *J* = 8.42, 1H), 7.47 (s, 2H) 7.16 (s, 1H), 4.37 (dd, *J* = 9.13, 1.40 Hz, 1H), 2.32 (s, 6H), 1.04 (2, 9H)

^{13}C NMR (101 MHz, DMSO) δ 173.0, 167.6, 137.7, 134.7, 133.0, 125.9, 61.2, 34.1, 27.4, 21.3.

HR-MS: calc. for $[M+Na]^+$ = 286.1414, observed: 286.1411 (-1.1 ppm)

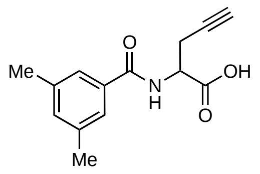

2-(3,5-dimethylbenzamido)pent-4-ynoic acid (36) was prepared according to the following procedure. To a solution of DL-propargylglycine (113 mg, 1 mmol) in dry MeOH (30 mL) at 0 °C was dropwise added $SOCl_2$ (0.175 mL, 2.4 mmol, 2.4 eq.). The reaction was stirred at 0 °C for 30 min before heating to 60 °C for 12 h. Next, the solvent was removed in vacuo, the residue was dissolved in DCM and dried over $MgSO_4$. The ester product was concentrated under reduced pressure and used without further purification in the synthesis of **36** according to general synthesis method D along with 3,4-dimethylbenzoic acid (125 mg, 0.833 mmol, 1 eq.). The product was obtained as a white solid (107 mg, 0.438 mmol, 53%).

^{1}H NMR (400 MHz, DMSO) δ 12.85 (s, 1H), 8.64 (d, *J* = 7.75 Hz, 1H), 7.49 (s, 2H), 7.18 (s, 1H), 4.53 (q, *J* = 7.75 Hz, 1H), 2.87 – 2.85 (m, 1H), 2.79 – 2.65 (m, 2H), 2.32 (s, 6H)

^{13}C NMR (101 MHz, DMSO) δ 172.4, 166.9, 137.9, 134.3, 133.2, 125.6, 81.4, 73.3, 52.1, 21.3, 21.2.

HR-MS: calc. for $[M+Na]^+$ = 268.0944, observed: 268.0945 (0.3 ppm)

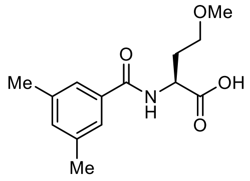

***N*-(3,5-Dimethylbenzoyl)-*O*-methyl-L-homoserine (37).** Methyl *O*-methyl-L-homoserinate hydrochloride was prepared according to a literature procedure from *N*-(*tert*-butyloxycarbonyl)- L-homoserine (1.1 g, 5 mmol, 1 eq.).[11] This was then used in general synthesis method D for the preparation of **37**. The product was obtained as an orange oil (148 mg, 0.559 mmol, 56%).

^{1}H NMR (500 MHz, $CDCl_3$) 10.28 (s, 1H), 7.64 (d, *J* = 6.40 Hz, 1H), 7.39 (s, 1H), 7.13 (s, 1H), 4.79 (q, *J* = 6.02 Hz, 1H), 3.69 – 3.57 (m, 2H), 3.38 (s, 3H), 2.33 (s, 6H), 2.29 – 2.16 (m, 2H)

^{13}C NMR (101 MHz, $CDCl_3$) δ 174.6, 168.5, 138.3, 133.6, 133.3, 125.0, 77.4, 77.1, 76.8, 70.0, 59.0, 52.2, 30.8, 21.2.

HR-MS: calc. for $[M-H]^-$: 264.1241, observed: 264.1237 (-1.6 ppm)

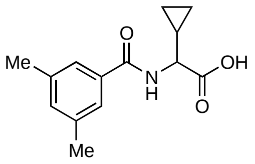

2-Cyclopropyl-2-(3,5-dimethylbenzamido)acetic acid (38) was prepared according to general synthesis method D using 3,5-dimethylbenzoic acid (60 mg, 0.4 mmol, 1 eq.) and methyl 2-amino-2-cyclopropylacetate (96 mg, 0.48 mmol, 1.2 eq.). The product was obtained as an oily off-white solid (64.2 mg, 0.26 mmol, 65%).

^{1}H NMR (400 MHz, DMSO) δ 12.36z (br s, 1H), 8.65 (d, *J* = 7.16 Hz, 1H), 7.51 (s, 2H), 7.16 (s, 1H), 3.69 (dd, *J* = 8.95, 7.32 Hz, 1H), 2.32 (s, 6H), 1.28 – 1.19 (m, 1H), 0.62 – 0.54 (m, 1H), 0.52 – 0.41 (m, 2H), 0.37 – 0.29 (m, 1H).

^{13}C NMR (101 MHz, DMSO) δ 173.7, 166.9, 137.8, 134.3, 133.0, 125.7, 57.4, 21.3, 13.2, 4.0, 4.0.

HR-MS: calc. for $[M-H]^-$: 246.1135, observed: 246.1128 (-3 ppm)

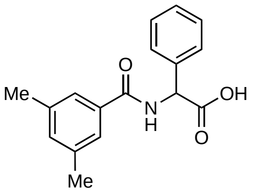

2-(3,5-Dimethylbenzamido)-2-phenylacetic acid (39) was prepared according to general synthesis method D using DL-phenylglycine methyl ester hydrochloride (97 mg, 0.48 mmol, 1.2 eq.) and 3,5-dimethylbenzoic acid (60 mg, 0.4 mmol, 1 eq.). The product was obtained as a white solid (50 mg, 0.175 mmol, 44%).

^{1}H NMR (400 MHz, $CDCl_3$) δ 12.89 (br s, 1H), 8.89 (d, *J* = 7.56 Hz, 1H), 7.54 (s, 2H), 7.51 – 7.47 (m, 2H), 7.41 – 7.29 (m, 3H), 7.16 (s, 1H), 5.59 (d, *J* = 7.52 Hz, 1H), 2.31 (s, 6H).

^{13}C NMR (101 MHz, $CDCl_3$) δ 172.4, 166.9, 137.8, 137.7, 134.2, 133.2, 128.9, 128.6, 128.3, 125.9, 57.3, 21.3.

HR-MS: calc. for $[M-H]^-$: 282.1135, observed: 282.1136 (0.2 ppm)

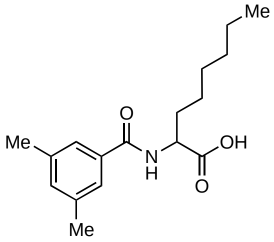

2-(3,5-Dimethylbenzamido)octanoic acid (40) was prepared according to general synthesis method D using 3,5-dimethylbenzoic acid (89 mg, 0.59 mmol, 1 eq.) and methyl 2-aminooctanoate (142 mg, 0.82 mmol, 1.4 eq.). The product was obtained as a white solid (155 mg, 0.53 mmol, 90%)

^{1}H NMR (400 MHz, DMSO) δ 12.50 (br s, 1H), 8.43 (d, *J* = 7.83 Hz, 1H)z, 7.49 (s, 1H), 7.16 (s, 1H), 4.34 (dt, *J* = 11.31, 5.38 Hz 1H), 2.32 (s, 6H), 1.77 (m, 2H), 1.33 – 1.24 (m, 8H), 0.85 (t, *J* = 6.62 Hz, 3H).

^{13}C NMR (101 MHz, DMSO) δ 174.4, 167.2, 137.8, 134.5, 133.0, 125.7, 53.0, 31.6, 31.1, 28.7, 26.2, 22.5, 21.3, 14.4.

HR-MS: calc. for $[M+Na]^+$: 314.1727, observed: 314.1739 (-3.82 ppm)

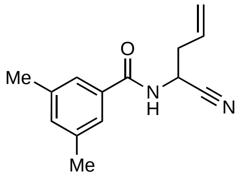

N-(1-cyanobut-3-en-1-yl)-3,5-dimethylbenzamide (41) was prepared according to general synthesis method D using 3,5-dimethylbenzoic acid (173 mg, 1.15 mmol, 1 eq.) and 2-aminopent-4-enenitrile hydrochloride (183 mg, 1.4 mmol, 1.2 eq.). The product was obtained as a white solid (235 mg, 1.03 mmol, 90%).

^{1}H NMR (400 MHz, DMSO) *δ* 9.07 (d, *J* = 7.8 Hz, 1H), 7.47 (s, 2H), 7.21 (s, 1H), 5.89 – 5.73 (m, 1H), 5.24 (dd, *J* = 17.2, 1.8 Hz, 1H), 5.18 (dd, *J* = 10.5, 1.8 Hz, 1H), 5.02 (m, 1H), 2.65 (m, 2H), 2.32 (s, 6H).

^{13}C NMR (101 MHz, DMSO) *δ* 166.4, 137.7, 133.2, 133.0, 132.4, 125.2, 119.4, 119.2, 40.3, 35.9, 20.8.

HR-MS: calc. for $[M+H]^+$: 229.1335, observed: 229.1341 (-2.62 ppm)

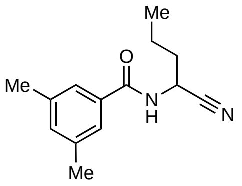

N-(1-Cyanobutyl)-3,5-dimethylbenzamide (42) was prepared according to general synthesis method E using 3,5-dimethylbenzoic acid (75 mg, 0.5 mmol, 1 eq.) and 2-aminopentanenitrile hydrochloride (81 mg, 0.6 mmol, 1.2 eq.). The product was obtained as a pale white solid (86 mg, 0.37 mmol, 74%).

^{1}H NMR (400 MHz, DMSO) δ 9.01 (d, *J* = 7.7 Hz, 1H), 7.48 (s, 2H), 7.21 (s, 1H), 4.92 (q, *J* = 7.7 Hz, 1H), 2.33 (s, 6H), 1.91 – 1.79 (m, 2H), 1.51 – 1.35 (m, 2H), 0.92 (t, *J* = 7.4 Hz, 3H).

^{13}C NMR (101 MHz, DMSO) δ 166.5, 137.6, 133.2, 133.0, 125.2, 119.7, 33.6, 20.8, 18.4, 13.1.

HR-MS: calc. for $[M-H]^-$: 229.1346, observed: 229.1347 (0.3 ppm)

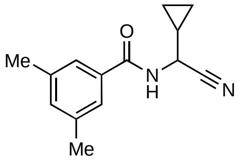

N-(cyano(cyclopropyl)methyl)-3,5-dimethylbenzamide (43) was prepared according to general synthesis method D using 3,5-dimethylbenzoic acid (90.1 mg, 0.6 mmol, 1 eq.) and 2-amino-2-cyclopropylacetonitrile hydrochloride (80 mg, 0.6 mmol, 1 eq.). The product was obtained as a white solid (77 mg, 0.337 mmol, 56%).

^{1}H NMR (400 MHz, DMSO) δ 9.22 (d, *J* = 7.65 Hz, 1H), 7.50 (s, 2H), 7.21 (s, 1H), 4.46 (dd, *J* = 8.82, 7.68 Hz, 1H), 2.33 (s, 6H), 1.51 – 1.41 (m, 1H), 0.69 – 0.62 (m, 1H), 0.62 – 0.53 (m, 2H), 0.45 – 0.36 (m, 1H).

^{13}C NMR (101 MHz, DMSO) δ 166.8, 138.1, 133.7, 133.4, 125.7, 119.5, 45.0, 21.3, 13.9, 4.7, 3.5.

HR-MS: calc. for $[M-H]^-$: 227.1189, observed: 227.1186 (-1.5 ppm)

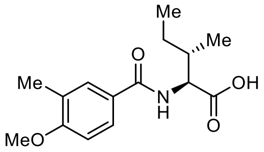

(4-Methoxy-3-methylbenzoyl)-L-isoleucine (44) was prepared according to general synthesis method D using of 4-methoxy-3-methylbenzoic acid (66 mg, 0.4 mmol, 1 eq.) and

L-isoleucine methyl ester hydrochloride (87 mg, 0.48 mmol, 1.2 eq.). The product was obtained as a white solid (56 mg, 0.21 mmol, 53%).

^{1}H NMR (400 MHz, DMSO) δ 12.53 (br s, 1H), 8.16 (d, *J* = 8.16 Hz, 1H), 7.77 - 7.73 (m, 2H), 6.99 (d, *J* = 6.99 Hz, 1H), 4.31 (t, *J* = 4.32 Hz, 1H), 3.84 (s, 2H), 2.18 (s, 3H), 1.97 - 1.90 (m, 1H), 1.54 - 1.23 (m, 2H) 0.93 - 0.84 (m, 6H)

^{13}C NMR (101 MHz, DMSO) δ 173.9, 166.7, 160.2, 130.3, 127.7, 126.2, 125.5, 110.0, 57.6, 56.0, 36.2, 25.6, 16.5, 16.1, 11.5.

HR-MS: calc. for $[M+Na]^+$: 302.1363, observed: 302.1368 (-1.65 ppm)

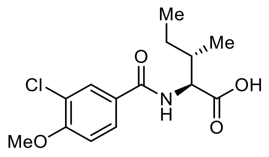

(3-Chloro-4-methoxybenzoyl)-L-isoleucine (45) was prepared according to general synthesis method D using 3-chloro-4-methoxybenzoic acid (75 mg, 0.4 mmol, 1 eq.) and L-isoleucine methyl ester hydrochloride (87 mg, 0.48 mmol, 1.2 eq.). The product was obtained as a white solid (84 mg, 0.28 mmol, 70%).

^{1}H NMR (400 MHz, DMSO) *δ* 12.57 (s, 1H), 8.42 (d, *J* = 8.0 Hz, 1H), 8.03 (d, *J* = 2.2 Hz, 1H), 7.89 (dd, *J* = 8.6, 2.3 Hz, 1H), 7.23 (d, *J* = 8.7 Hz, 1H), 4.31 (t, *J* = 7.5 Hz, 1H), 3.92 (s, 3H), 1.98 – 1.87 (m, 1H), 1.54 – 1.24 (m, 2H), 0.92 (d, *J* = 6.8 Hz, 3H), 0.86 (t, *J* = 7.4 Hz, 3H).

^{13}C NMR (101 MHz, DMSO) *δ* 173.2, 165.0, 156.8, 129.1, 128.4, 127.0, 120.7, 112.2, 57.3, 56.4, 35.7, 25.2, 15.7, 11.0.

HR-MS: calc. for $[M+H]^+$: 300.0997, observed: 300.1006 (-3.00 ppm)

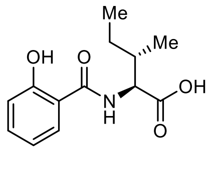

(2-Hydroxybenzoyl)-L-isoleucine (46) was prepared according to general synthesis method C using salicylic acid (81 mg, 0.585 mmol, 1 eq.) and L-isoleucine methyl ester hydrochloride (110 mg, 0.76 mmol, 1.3 eq.). The product was obtained as a colourless gum (58.95 mg, 0.235 mmol, 40%).

¹H NMR (400 MHz, $CDCl_3$) δ 11.94 (s, 1H), 8.82 (s, 1H), 7.45-7.39 (m, 2H), 6.99 (d, *J* = 7.6 Hz, 1H), 6.90-6.83 (m, 2H), 4.82 (s, 1H), 2.13-2.04 (m, 1H), 1.65-1.54 (m, 1H), 1.37-1.29 (m, 1H), 1.07-0.93.
¹³C NMR (101 MHz, $CDCl_3$) δ 176.9, 169.9, 161.6, 134.8, 125.8, 119.1, 118.8, 114.0, 56.5, 38.0, 25.4, 15.6, 11.7.
HR-MS: calc. for $[M+H]^+$: 252.1230, observed: 252.1231.

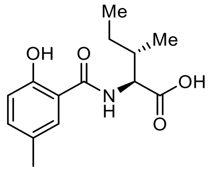

(2-Hydroxy-5-methylbenzoyl)-L-isoleucine (47) was prepared according to general synthesis method C using 5-methylsalicylic acid (88.95 mg, 0.585 mmol, 1 eq.) and L-isoleucine methyl ester hydrochloride (110 mg, 0.76 mmol, 1.3 eq.). The product was obtained as a colourless gum (36.81 mg, 0.139 mmol, 24%)
¹H NMR (400 MHz, $CDCl_3$) δ 11.72 (s, 1H), 7.24-7.19 (m, 2H), 6.89 (d, *J* = 8.3 Hz, 1H), 6.81 (d, *J* = 8.3 Hz, 1H), 4.84-4.80 (m, 1H), 2.30 (s, 3H), 2.14-2.04 (m, 1H), 1.65-1.55 (m, 1H), 1.38-1.27 (m, 1H), 1.04-0.98 (m, 6H).
¹³C NMR (101 MHz, $CDCl_3$): δ 177.0, 169.9, 159.4, 135.7, 128.2, 125.6, 118.6, 113.6, 56.5, 38.0, 25.4, 20.7, 15.6, 11.7.
HR-MS: calc. for $[M+H]^+$: 266.1387, observed: 266.1381.

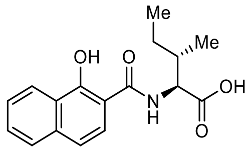

(1-Hydroxy-2-naphthoyl)-L-isoleucine (48) was prepared according to general synthesis method C using 1-hydroxy-2-napthoic acid (110 mg, 0.585 mmol, 1 eq.) and L-isoleucine methyl ester hydrochloride (110 mg, 0.76 mmol, 1.3 eq.). The product was obtained as a colourless oil (21.15 mg, 0.07 mmol, 12%).
¹H NMR (400 MHz, $CDCl_3$) δ 13.44 (s, 1H), 8.42 (d, *J* = 8.2 Hz, 1H), 7.76 (d, *J* = 8.0 Hz, 1H), 7.61-7.50 (m, 2H), 7.39-7.27 (m, 2H), 6.81 (d, *J* = 8.2, 1H), 4.89 (q, *J* = 4.3 Hz, 1H), 2.13 (m, 1H), 1.69-1.59 (m, 1H), 1.40-1.30 (m, 1H), 1.07-1.00 (m, 6H).
¹³C NMR (101 MHz, $CDCl_3$) δ 177.1, 170.1, 161.0, 136.7, 129.3, 127.5, 126.1, 125.7, 124.1, 120.9, 118.6, 106.4, 56.5, 38.1, 25.5, 15.6, 11.8.

HR-MS: calc. for $[M+H]^+$: 302.1387, observed: 302.1383.

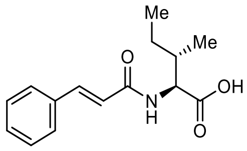

Cinnamoyl-L-isoleucine (49) was prepared according to general synthesis method C using *trans*-cinnamic acid (121 mg, 0.817 mmol, 1 eq.) and L-isoleucine methyl ester hydrochloride (129 mg, 1.06 mmol, 1.3 eq.). The product was obtained as a white solid (27 mg, 0.124 mmol, 15%).

^{1}H NMR (400 MHz, MeOD) δ 7.61-7.48 (m, 3H), 7.42-7.29 (m, 3H), 6.79 (d, J = 15.8 Hz, 1H), 4.53 (d, J = 5.6 Hz, 1H), 2.00-1.91 (m, 1H), 1.62-1.25 (m, 2H), 1.05-0.87 (m, 6H).

^{13}C NMR (101 MHz, MeOD) δ 174.8, 168.6, 142.3, 136.3, 130.9, 129.9, 128.9, 121.5, 58.3, 38.5, 26.3, 16.1, 11.9.

HR-MS: calc. for $[M+H]^+$: 262.1438, observed: 262.1436.

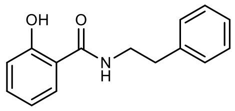

2-hydroxy-N-phenethylbenzamide (50) was prepared according to general synthesis method F using salicylic acid (83 mg, 0.6 mmol, 1 eq.) and 2-phenylethylamine (87 mg, 0.72 mmol, 1.2 eq.). The product was obtained as a white solid (52 mg, 0.2 mmol, 33%)

^{1}H NMR (400 MHz, $CDCl_3$) δ 12.27 (s, 1H), 7.32-7.13 (m, 6H), 7.11 (d, *J* = 8 Hz, 1H), 6.89 (d, *J* = 9.6 Hz, 1H), 6.71 (t, *J* = 8.3 Hz, 1H), 6.25 (s, 1H), 3.62 (q, *J* = 6.6 Hz, 2H), 2.85 (t, *J* = 6.9 Hz, 2H).

^{13}C NMR (101 MHz, $CDCl_3$) δ 170.1, 161.7, 138.6, 134.3, 129.0, 128.9, 126.9, 125.3, 118.8, 114.4, 40.9, 35.7.

HR-MS: calc. for $[M+H]^+$: 242.1176, observed: 242.1188.

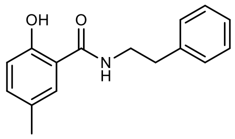

2-hydroxy-5-methyl-N-phenethylbenzamide (51) was prepared according to general synthesis method F using 5-methylsalicylic acid (91 mg, 0.6 mmol, 1 eq.) and 2-phenylethylamine (87 mg, 0.72 mmol, 1.2 eq.). The product was obtained as a white solid (71 mg, 0.272 mmol, 45%).

^{1}**H NMR** (400 MHz, $CDCl_3$) δ 12.09 (s, 1H), 7.37-7.18 (m, 6H), 6.89 (d, *J* = 8.5 Hz, 1H), 6.67 (s, 1H), 6.30 (s, 1H), 3.70 (q, *J* = 6.6 Hz, 2H), 2.94 (t, *J* = 7.0 Hz, 2H), 2.25 (s, 3H).
^{13}**C NMR** (101 MHz, $CDCl_3$) δ 170.1, 159.5, 138.7, 135.2, 129.0, 127.8, 126.9, 125.3, 118.5, 114.0, 40.9, 35.7, 20.7.
HR-MS: calc. for $[M+H]^+$: 256.1332, observed: 256.1330.

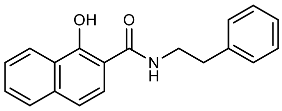

1-hydroxy-N-phenethyl-2-naphthamide (52) was prepared according to general synthesis method F using 1-hydroxy-2-napthoic acid (113 mg, 0.6 mmol, 1 eq.) and 2-phenylethylamine (87 mg, 0.72 mmol, 1.2 eq.). The product was obtained as a white solid (56 mg, 0.192 mmol, 32%).
^{1}**H NMR** (400 MHz, $CDCl_3$) δ 13.83 (s, 1H), 8.44 (d, *J* = 8.2 Hz, 1H), 7.74 (d, *J* = 8.9 Hz, 1H), 7.60-7.51 (m, 2H), 7.36 (t, *J* = 7.2, 2H), 7.30-7.21 (m, 4H), 7.14 (d, *J* = 8.8 Hz, 1H), 6.34 (s, 1H), 3.77 (q, *J* = 6.6 Hz, 2H), 2.98 (t, *J* = 6.9 Hz, 2H).
^{13}**C NMR** (101 MHz, $CDCl_3$) δ 170.7, 160.8, 138.7, 136.4, 129.0, 129.0, 129.0, 127.4, 126.9, 126.0, 125.8, 124.0, 120.7, 118.3, 106.7, 40.9, 35.8.
HR-MS: calc. for $[M+H]^+$: 292.1332, observed: 292.1337.

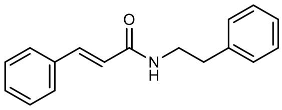

N-phenethylcinnamamide (53) was prepared according to general synthesis method F using cinnamic acid (89 mg, 0.6 mmol, 1 eq.) and 2-phenylethylamine (87 mg, 0.72 mmol, 1.2 eq.). The product was obtained as a white solid (84 mg, 0.334 mmol, 56%).
^{1}**H NMR** (400 MHz, $CDCl_3$) δ 7.54 (d, *J* = 15.5 Hz, 1H), 7.43-7.38 (m, 2H), 7.33-7.09 (m, 7H), 6.25 (d, J = 15.6 Hz, 1H), 5.62 (s, 1H), 3.59 (q, *J* = 6.6 Hz, 2H), 2.82 (t, *J* = 6.9 Hz, 2H).
^{13}**C NMR** (101 MHz, $CDCl_3$) δ 166.0, 141.2, 139.0, 134.9, 129.8, 129.0, 128.9, 128.3, 127.9, 126.7, 120.7, 40.9, 35.8.
HR-MS: calc. for $[M+H]^+$: 252.1383, observed: 252.1379.

HPLC traces and quantification of enzymatic cascade reactions

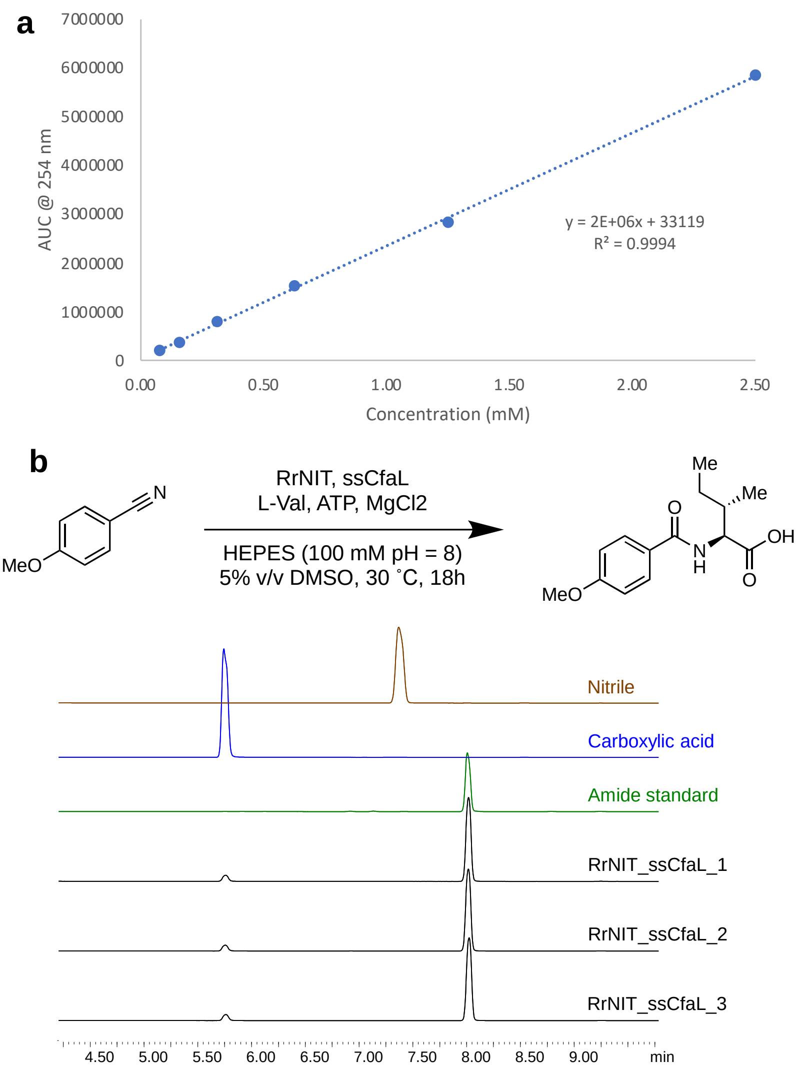

Figure S7. Enzymatic cascade reaction for the synthesis of 1. a, HPLC calibration using amide synthetic standard. **b,** Reaction scheme and HPLC chromatogram (HPLC method A). Traces recorded at 254 nm. Prepared according to the general method 1 using 4-methoxybenzonitrile and L-isoleucine employing RrNIT and SsCfaL. HPLC yield: run1 = 87%, run2 = 85%, run3 = 86%. Average: 86 ± 1% (triplicate).

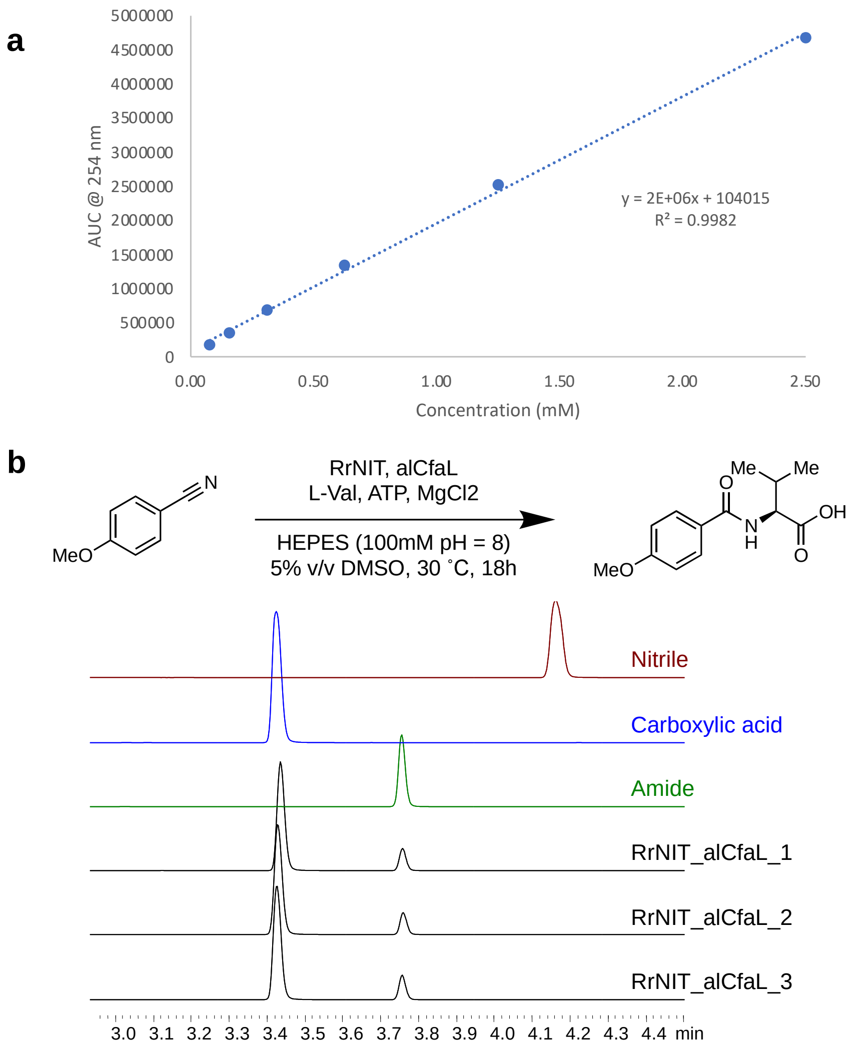

Figure S8. Enzymatic cascade reaction for the synthesis of 2. a, HPLC calibration using amide synthetic standard. **b,** Reaction scheme and HPLC chromatogram (HPLC method A). Traces recorded at 254 nm. Prepared according to the general method 1 using 4-methoxybenzonitrile and L-valine employing RrNIT and AlCfaL. HPLC yield: run1 = 18%, run2 = 18%, run3 = 20%. Average: 19 ± 1% (triplicate).

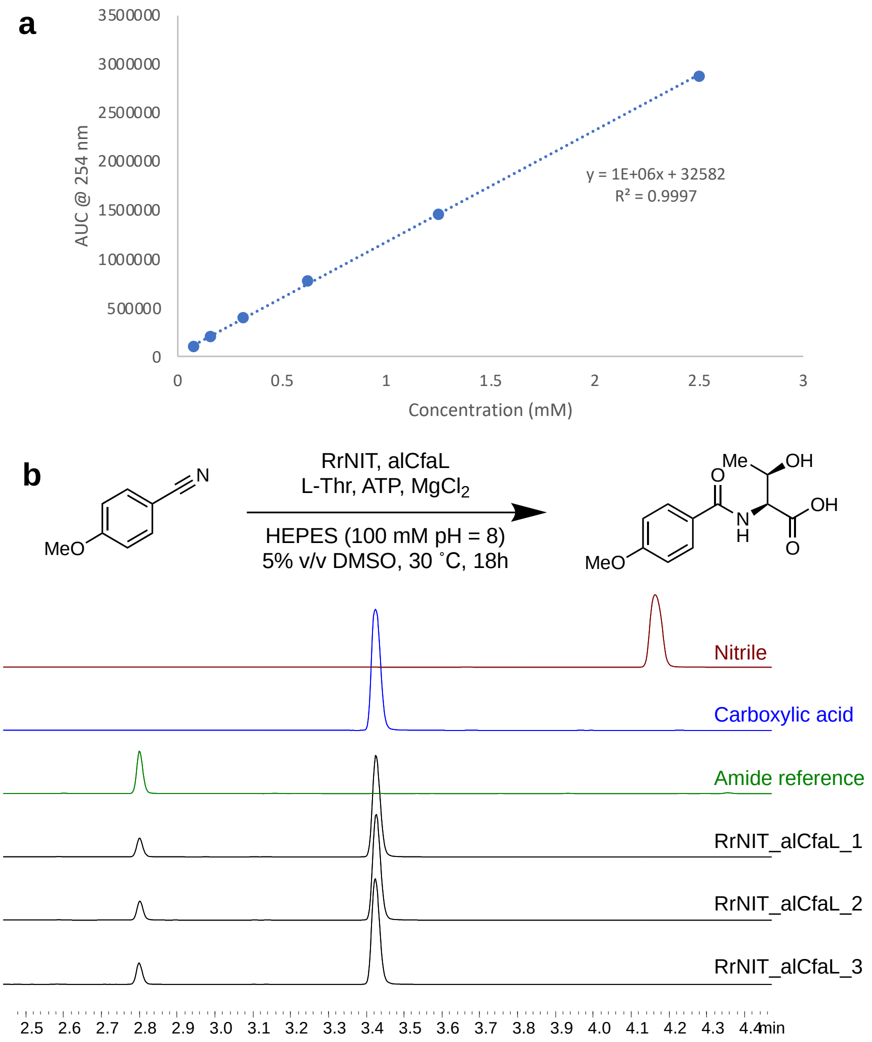

Figure S9. Enzymatic cascade reaction for the synthesis of 3. a, HPLC calibration using amide synthetic standard. **b,** Reaction scheme and HPLC chromatogram (HPLC method A). Traces recorded at 254 nm. Prepared according to the general method 1 using 4-methoxybenzonitrile and L-threonine employing RrNIT and AlCfaL. HPLC yield: run1 = 27%, run2 = 27%, run3 = 31%. Average: 28 ± 2% (triplicate).

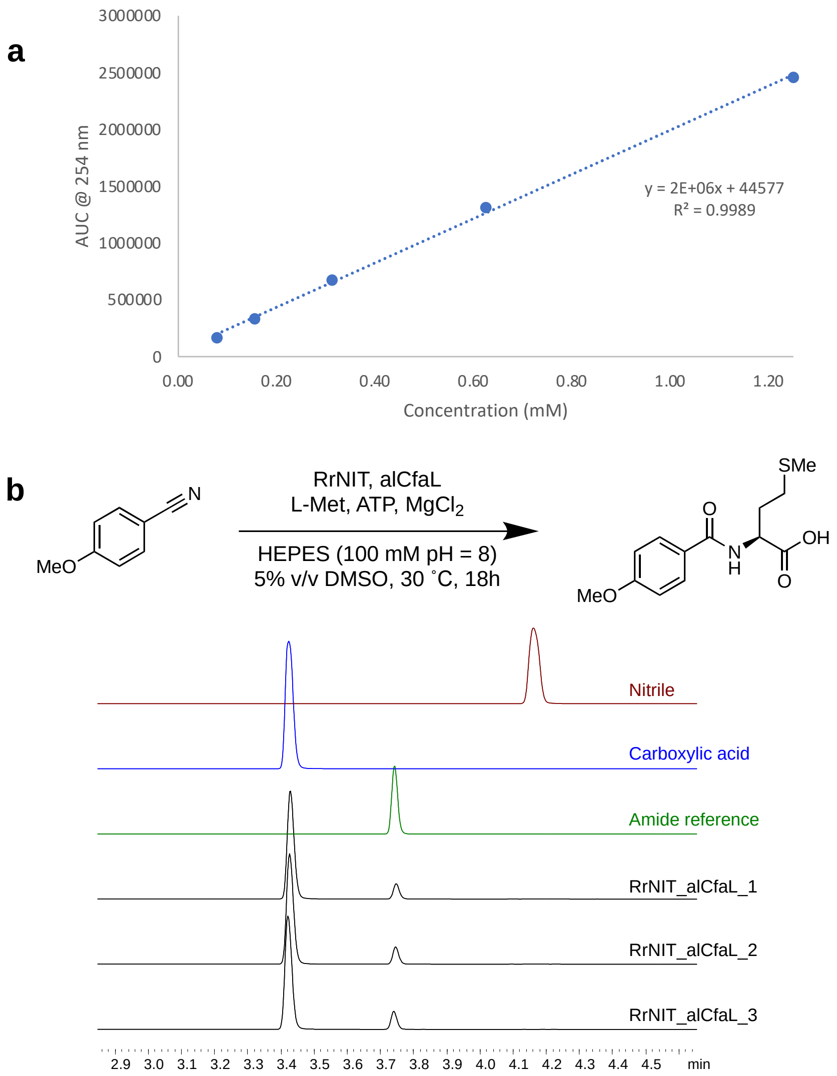

Figure S10. Enzymatic cascade reaction for the synthesis of 4. a, HPLC calibration using amide synthetic standard. **b,** Reaction scheme and HPLC chromatogram (HPLC method A). Traces recorded at 254 nm. Prepared according to the general method 1 using 4-methoxybenzonitrile and L-methionine employing RrNIT and AlCfaL. HPLC yield: run1 = 13%, run2 = 15%, run3 = 15%. Average: 14 ± 1% (triplicate).

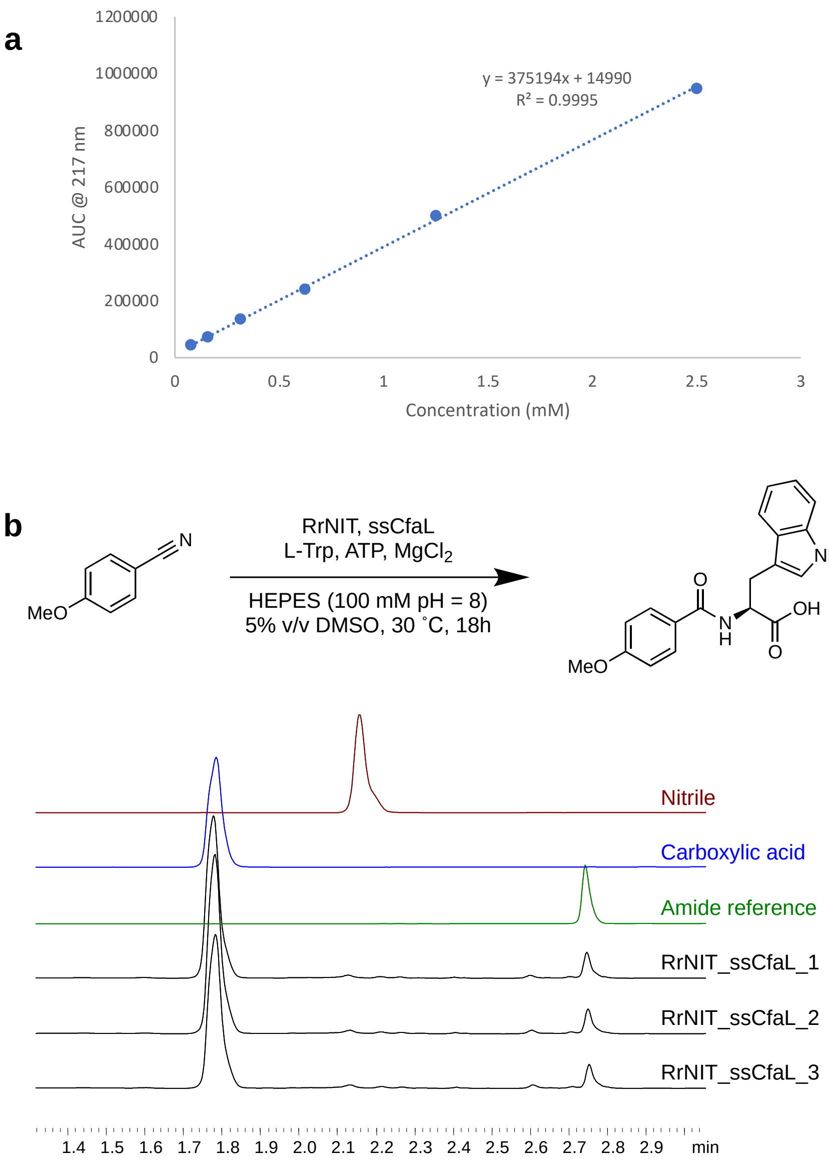

Figure S11. Enzymatic cascade reaction for the synthesis of 5. a, HPLC calibration curve using amide synthetic standard. **b,** Reaction scheme and HPLC chromatogram (HPLC method B). Traces recorded at 217 nm. Prepared according to the general method 1 using 4-methylbenzonitrile and L-tryptophan employing RrNIT and SsCfaL. HPLC yield: run1 = 28%, run2 = 26%, run3 = 26%. Average: 27 ± 1% (triplicate).

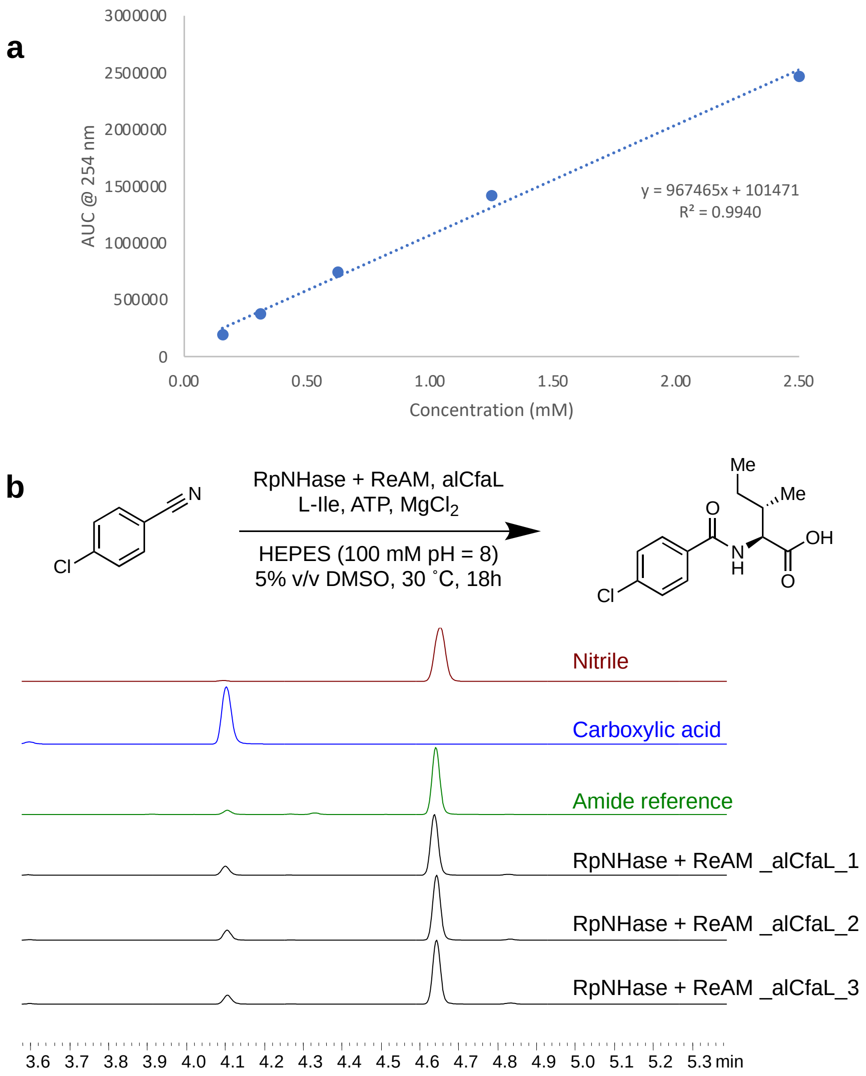

Figure S12. Enzymatic cascade reaction for the synthesis of 6. a, HPLC calibration curve using amide synthetic standard. **b,** Reaction scheme and HPLC chromatogram (HPLC method A). Traces recorded at 254 nm. Prepared according to the general method 1 using 4-chlorobenzonitrile and L-isoleucine employing RpNHase, ReAM and AlCfaL. HPLC yield: run1 = 75%, run2 = 81%, run3 = 78%. Average: 78 ± 2% (triplicate).

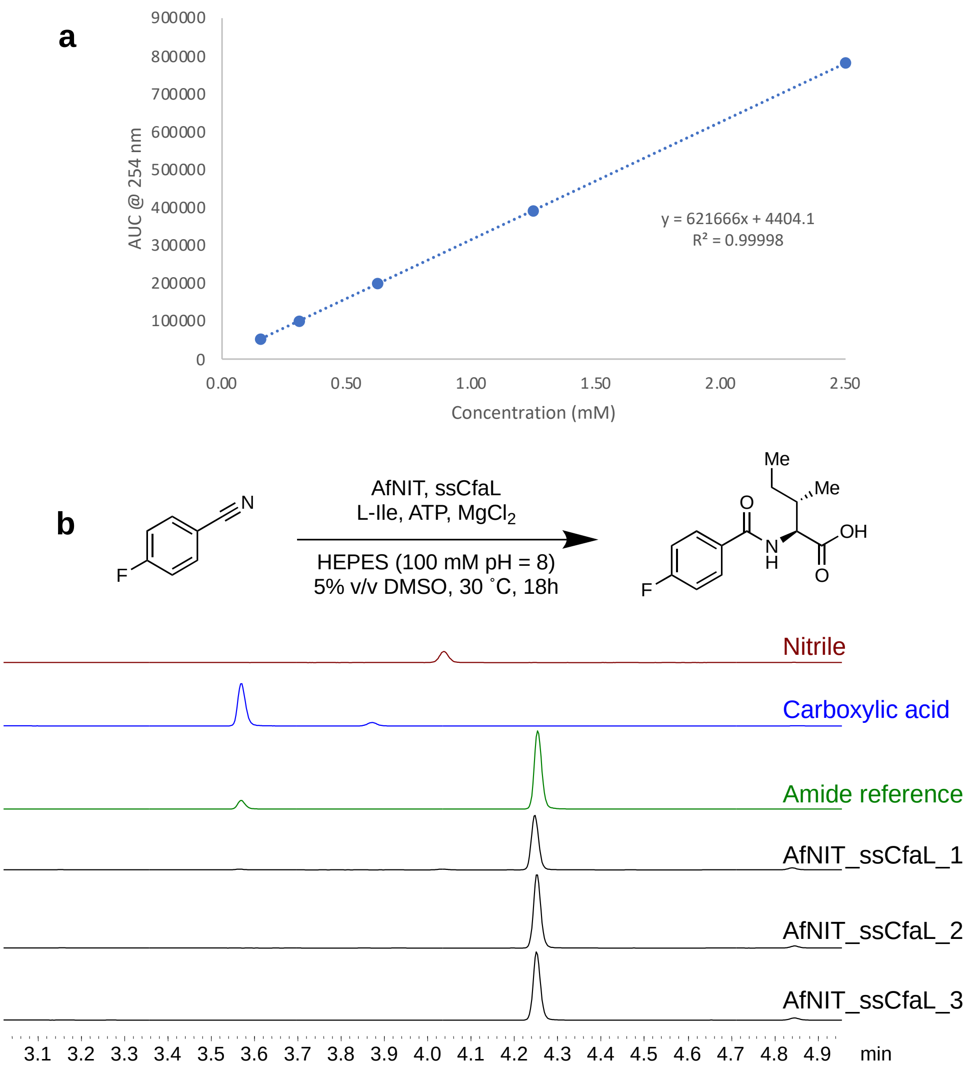

Figure S13. Enzymatic cascade reaction for the synthesis of 7. a, HPLC calibration using amide synthetic standard. **b,** Reaction scheme and HPLC chromatogram (HPLC method A). Traces recorded at 254 nm. Prepared according to the general method 1 using 4-fluorobenzonitrile and L-isoleucine employing AfNIT and AlCfaL. HPLC yield: run1 = 79%, run2 = 105%, run3 = 97%. Average: 94 ± 11% (triplicate).

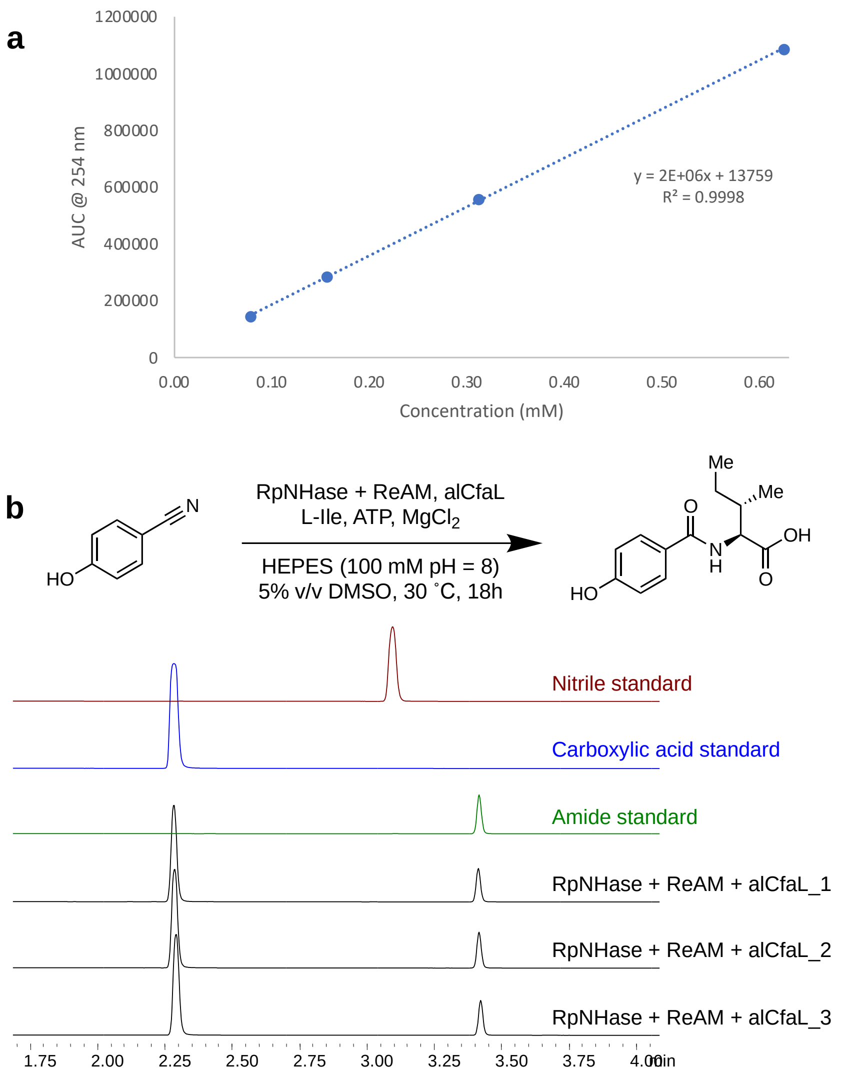

Figure S14. Enzymatic cascade reaction for the synthesis of 8. a, HPLC calibration using amide synthetic standard. b, Reaction scheme and HPLC chromatogram (HPLC method A). Traces recorded at 254 nm. Prepared according to the general method 1 using 4-hydroxybenzonitrile and L-isoleucine employing RpNHase, ReAM and AlCfaL. HPLC yield: run1 = 25%, run2 = 27%, run3 = 25%. Average: 26 ± 1% (triplicate).

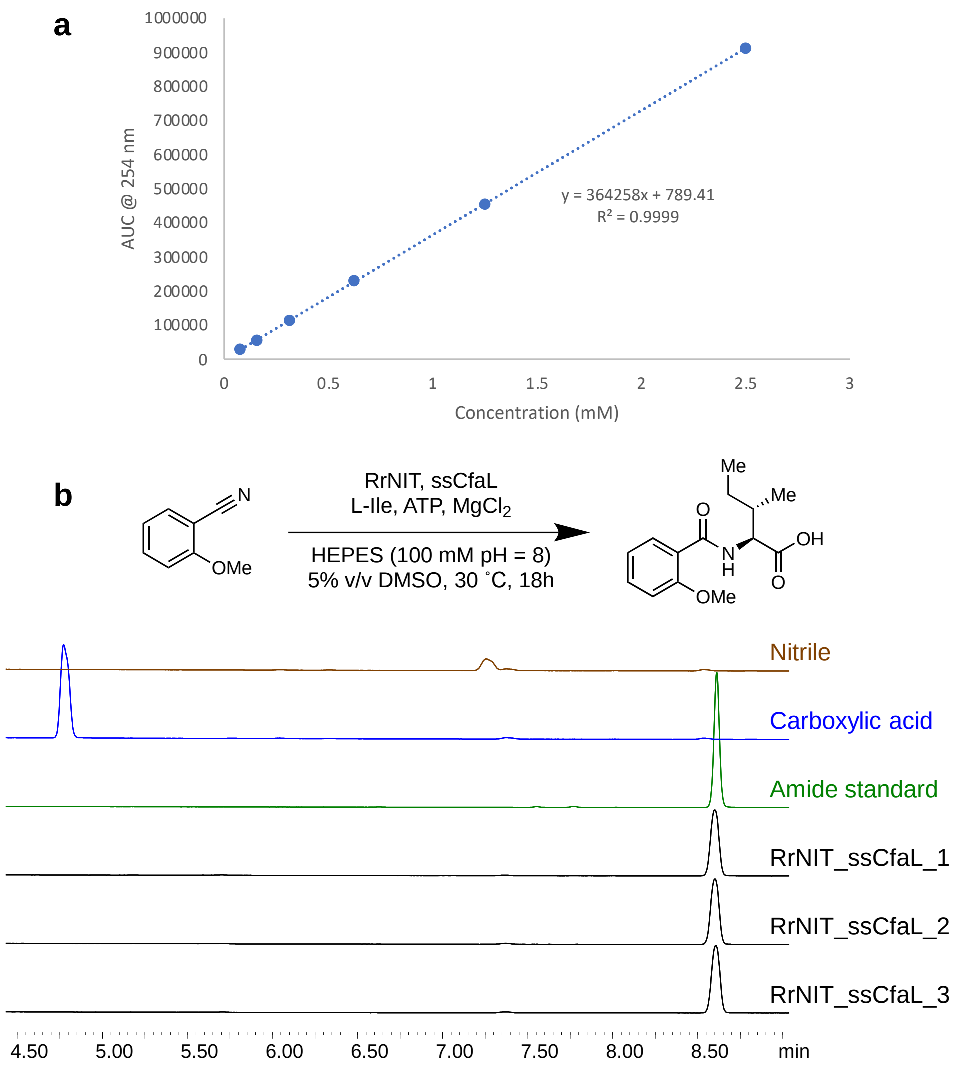

Figure S15. Enzymatic cascade reaction for the synthesis of 9. a, HPLC calibration using amide synthetic standard**. b**, Reaction scheme and HPLC chromatogram (HPLC method A). Traces recorded at 254 nm. Prepared according to the general method 1 using 2-methoxylbenzonitrile and L-isoleucine employing RrNIT and SsCfaL. HPLC yield: run1 = 46%, run2 = 45%, run3 = 47%. Average: 46 ± 1% (triplicate).

a

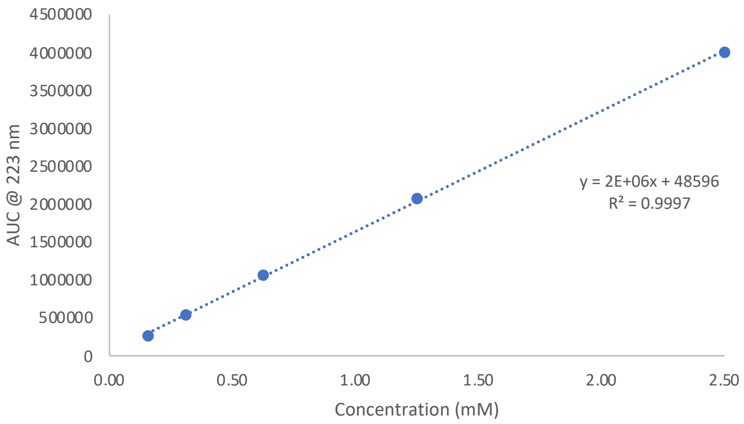

b

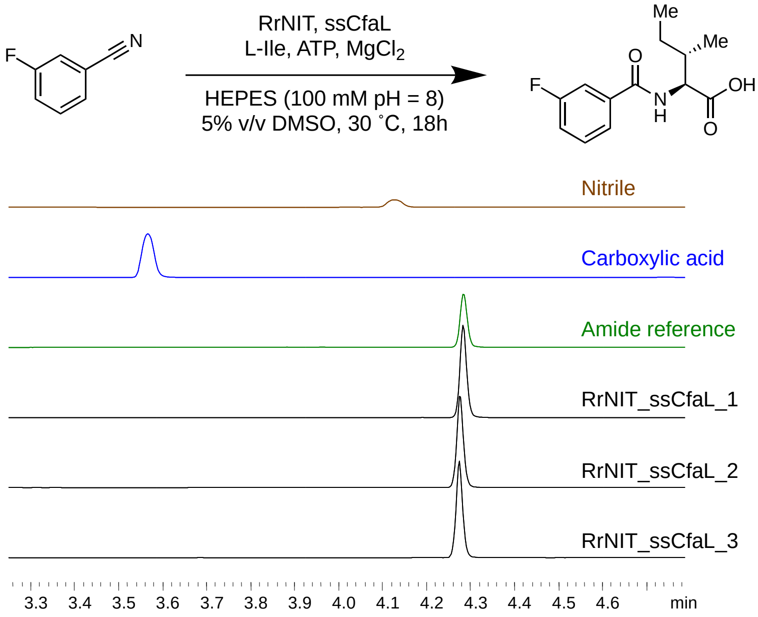

Figure S16. Enzymatic cascade reaction for the synthesis of 10. a, HPLC calibration using amide synthetic standard. **b,** Reaction scheme and HPLC chromatogram (HPLC method A). Traces recorded at 223 nm. Prepared according to the general method 1 using 3-fluorobenzonitrile and L-isoleucine employing RrNIT and SsCfaL. HPLC yield: run1 > 99%, run2 > 99%, run3 > 99%. Average: > 99% (triplicate).

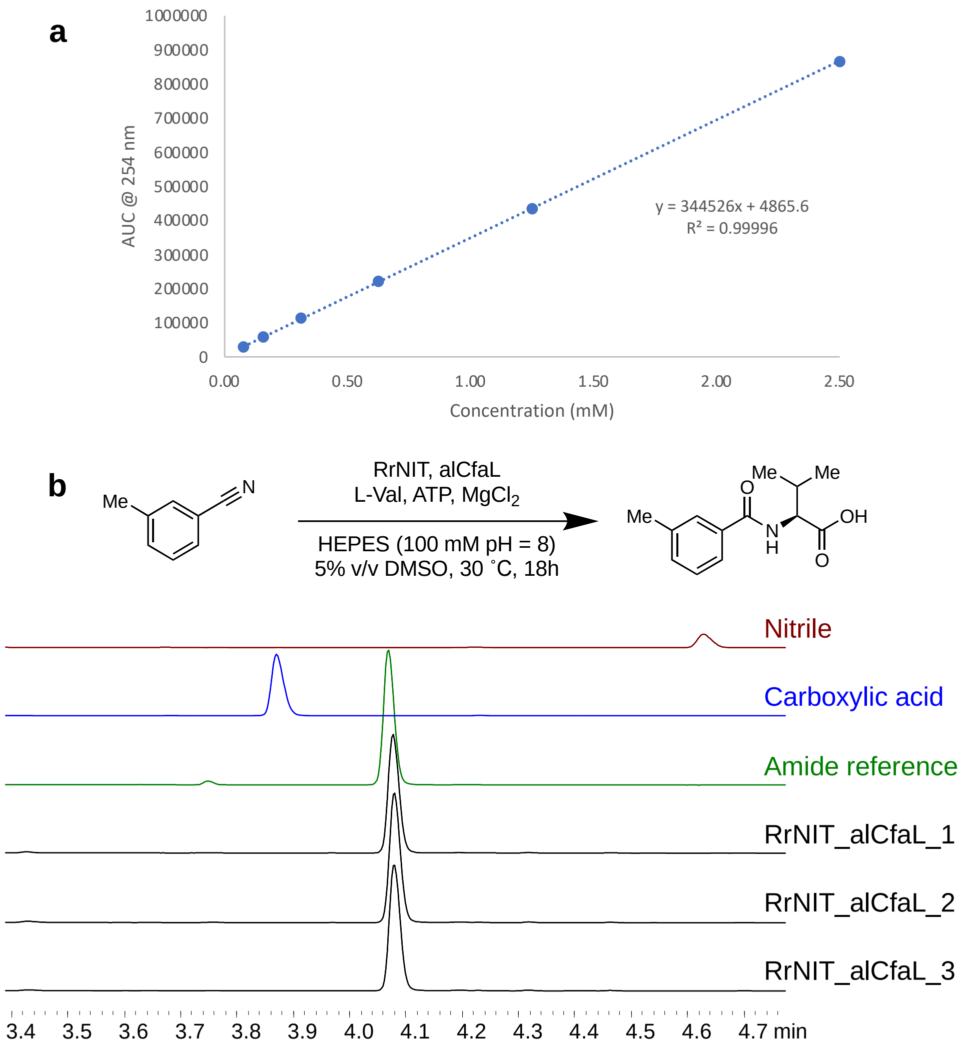

Figure S17. Enzymatic cascade reaction for the synthesis of 11. a, HPLC calibration using amide synthetic standard. **b,** Reaction scheme and HPLC chromatogram (HPLC method A). Traces recorded at 254 nm. Prepared according to the general method 1 using 3-methylbenzonitrile and L-valine employing RrNIT and AlCfaL. HPLC yield: run1 > 99%, run2 > 99%, run3 > 99%. Average: > 99% (triplicate).

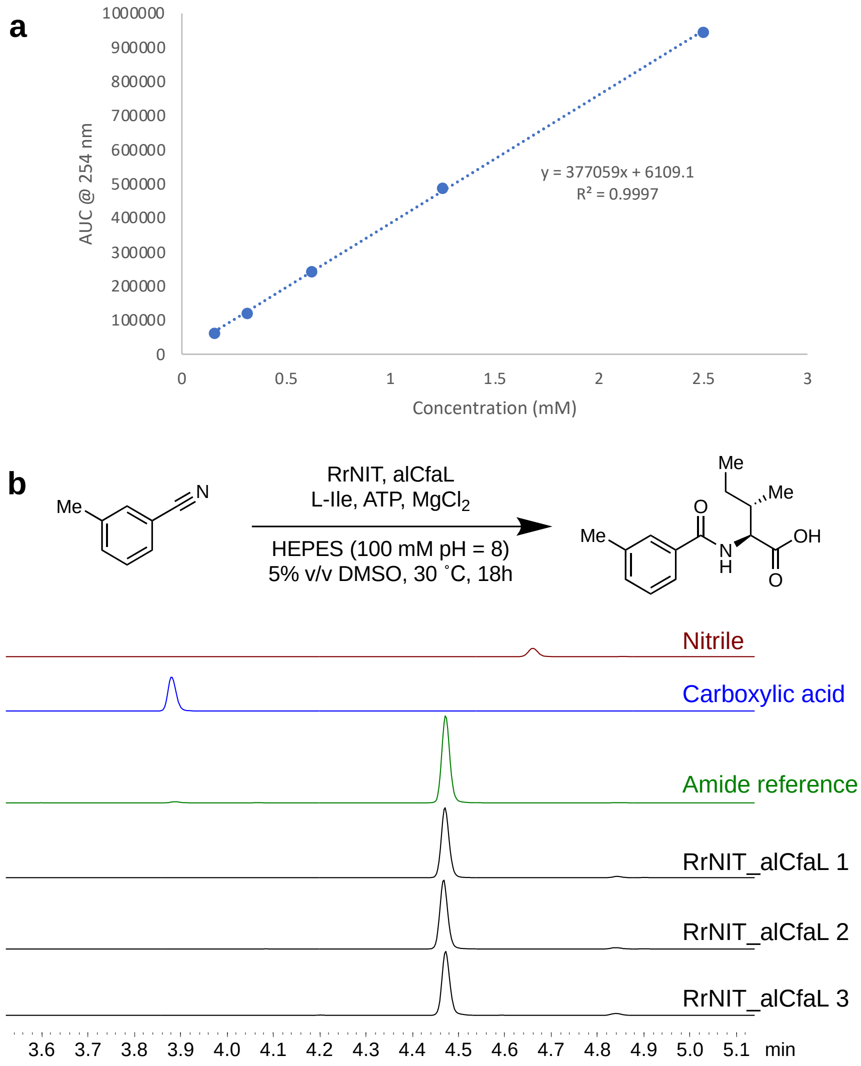

Figure S18. Enzymatic cascade reaction for the synthesis of 12. a, HPLC calibration using amide synthetic standard. **b,** Reaction scheme and HPLC chromatogram (HPLC method A). Traces recorded at 254 nm. Prepared according to the general method 1 using 3-methylbenzonitrile and L-isoleucine employing RrNIT and AlCfaL. HPLC yield: run1 > 99%, run2 > 99%, run3 > 99%. Average: > 99% (triplicate).

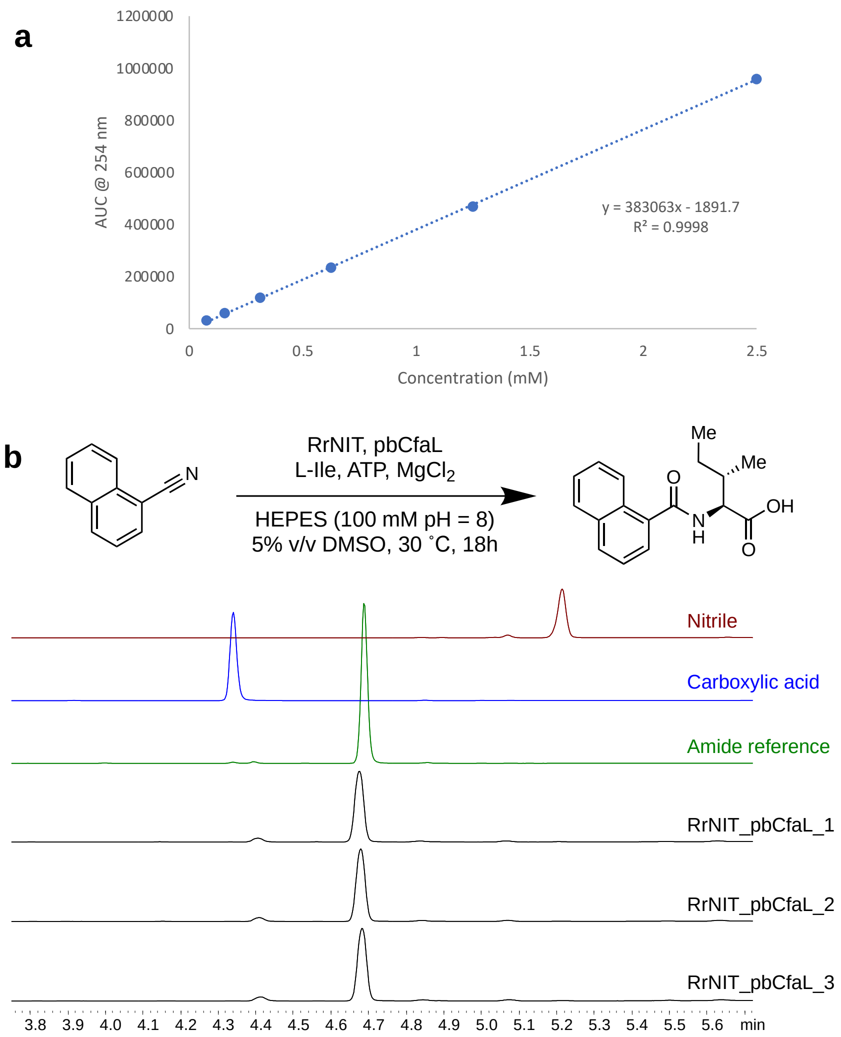

Figure S19. Enzymatic cascade reaction for the synthesis of 13. a, HPLC calibration using amide synthetic standard. **b,** Reaction scheme and HPLC chromatogram of the analytical scale cascade reaction (HPLC method A). Traces recorded at 254 nm. Prepared according to the general method 1 using 1-naphtonitrile and L-isoleucine, using RrNIT and PbCfaL. HPLC yield: run1 = 80%, run2 = 83%, run3 = 83%. Average: 82 ± 1% (triplicate).

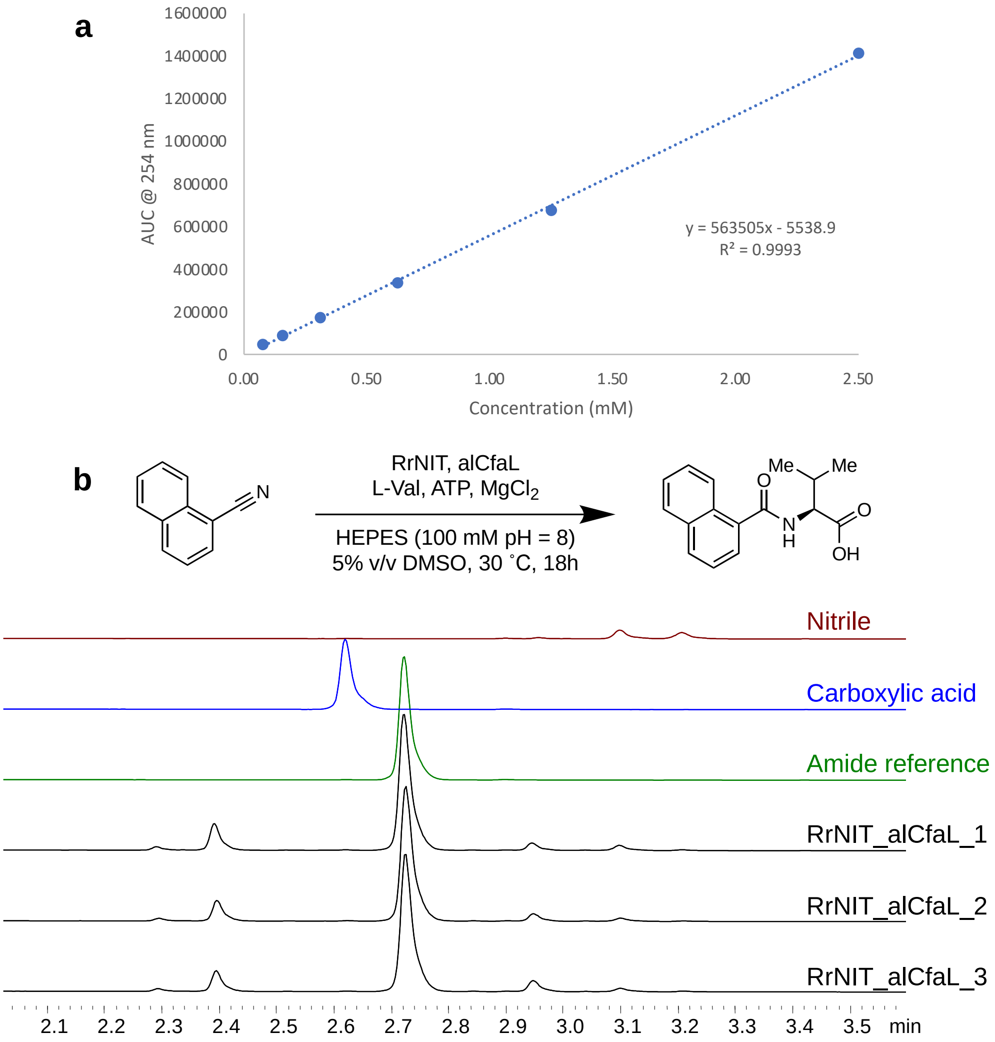

Figure S20. Enzymatic cascade reaction for the synthesis of 14. a, HPLC calibration using amide synthetic standard. **b,** Reaction scheme and HPLC chromatogram (HPLC method B). Traces recorded at 254 nm. Prepared according to the general method 1 using 1-naphtonitrile and L-valine employing RrNIT and AlCfaL. HPLC yield: run1 = 68%, run2 = 73%, run3 = 76%. Average: 72 ± 4% (triplicate).

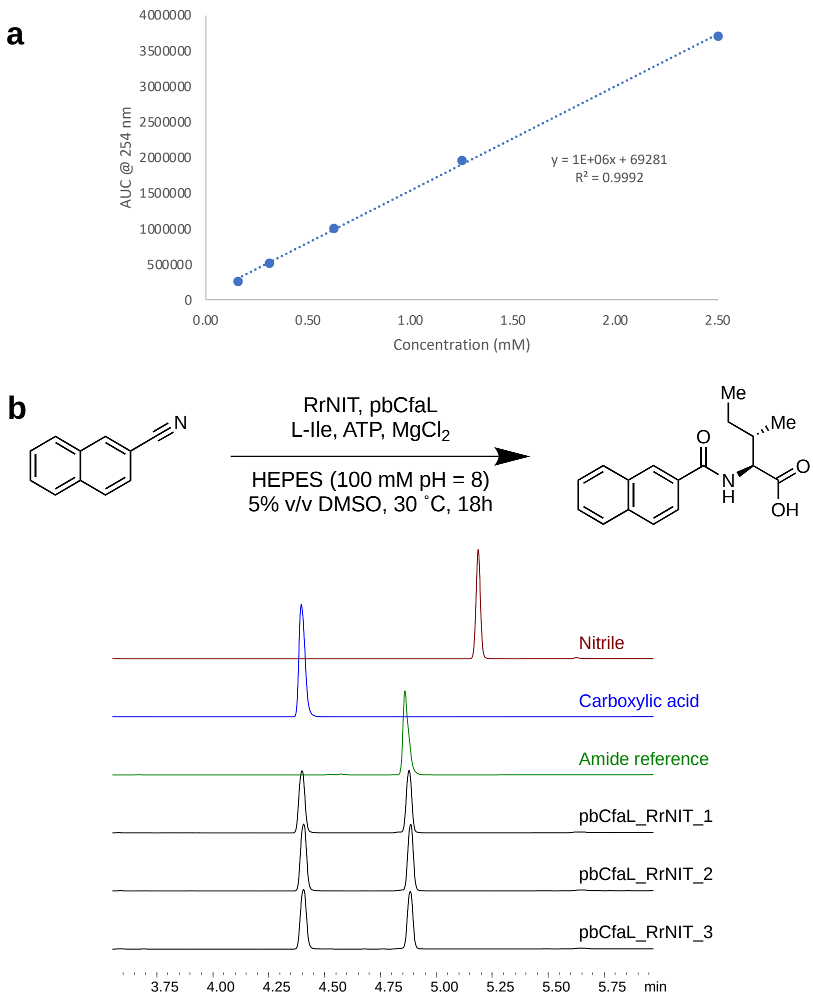

Figure S21. Enzymatic cascade reaction for the synthesis of 15. a, HPLC calibration using amide synthetic standard. **b,** Reaction scheme and HPLC chromatogram (HPLC method A). Traces recorded at 254 nm. Prepared according to the general method 1 using 2-naphtonitrile and L-isoleucine employing RrNIT and PbCfaL. HPLC yield: run1 = 22%, run2 = 23%, run3 = 20%. Average: 22 ± 1% (triplicate).

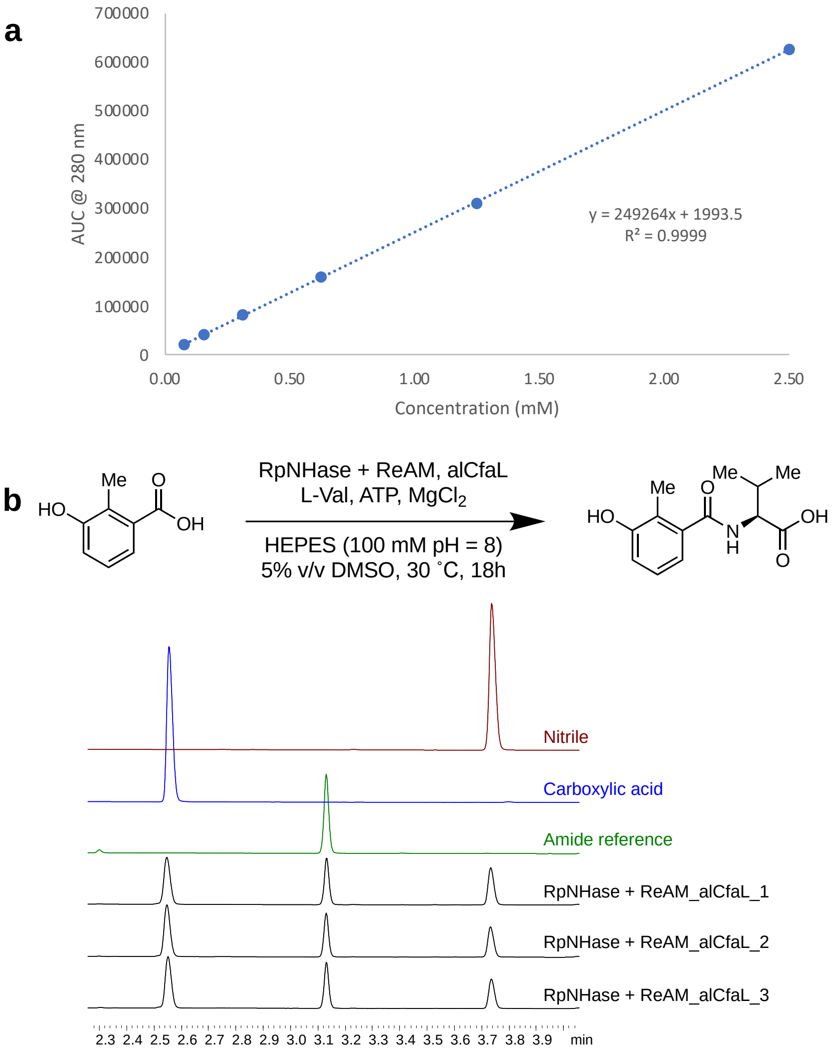

Figure S22. Enzymatic cascade reaction for the synthesis of 16. a, HPLC calibration using amide synthetic standard. **b,** Reaction scheme and HPLC chromatogram (HPLC method A). Traces recorded at 280 nm. Prepared according to the general method 1 using 3-hydroxy-2-methylbenzonitrile and L-valine employing RpNHase, ReAM and AlCfaL. HPLC yield: run1 = 36%, run2 = 33%, run3 = 35%. Average: 35 ± 1% (triplicate).

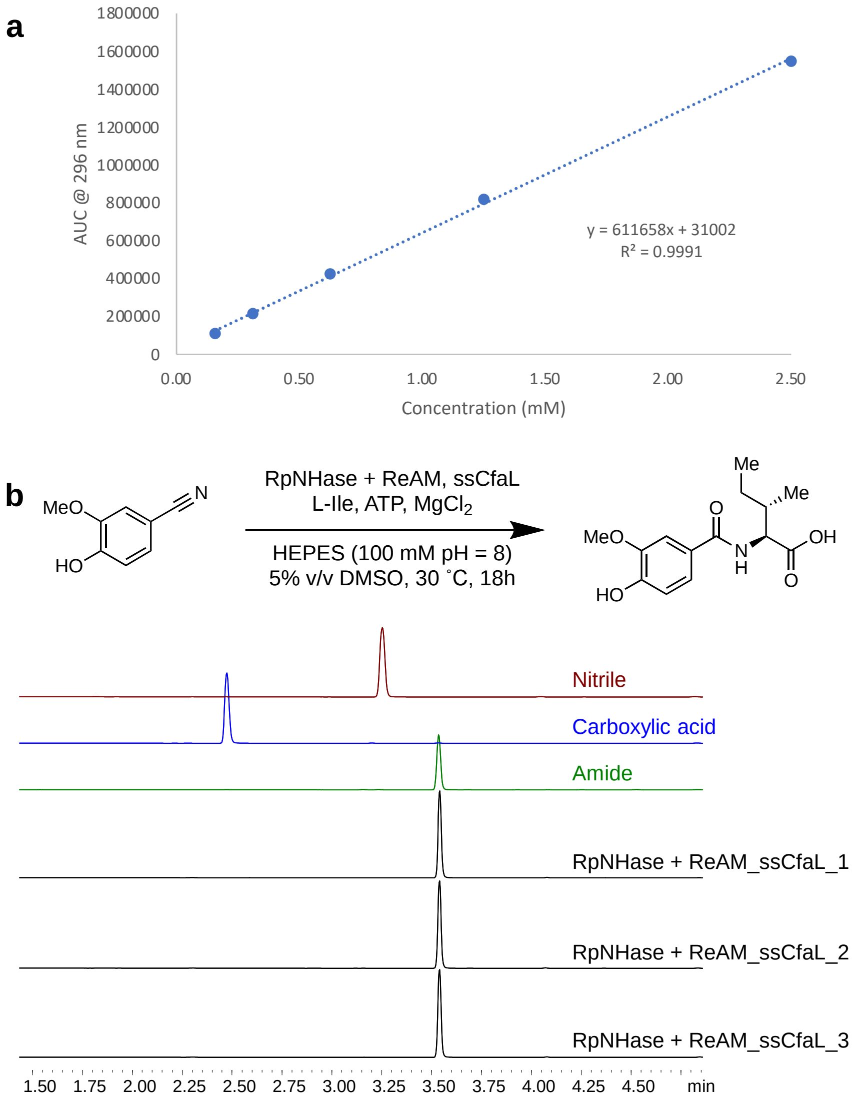

Figure S23. Enzymatic cascade reaction for the synthesis of 17. a, HPLC calibration using amide synthetic standard. **b,** Reaction scheme and HPLC chromatogram (HPLC method A). Traces recorded at 296 nm. Prepared according to the general method 1 using 4-hydroxy-3-methoxybenzonitrile and L-isoleucine employing RpNHase, ReAM and SsCfaL. HPLC yield: run1 > 99%, run2 > 99%, run3 > 99%. Average: > 99% (triplicate).

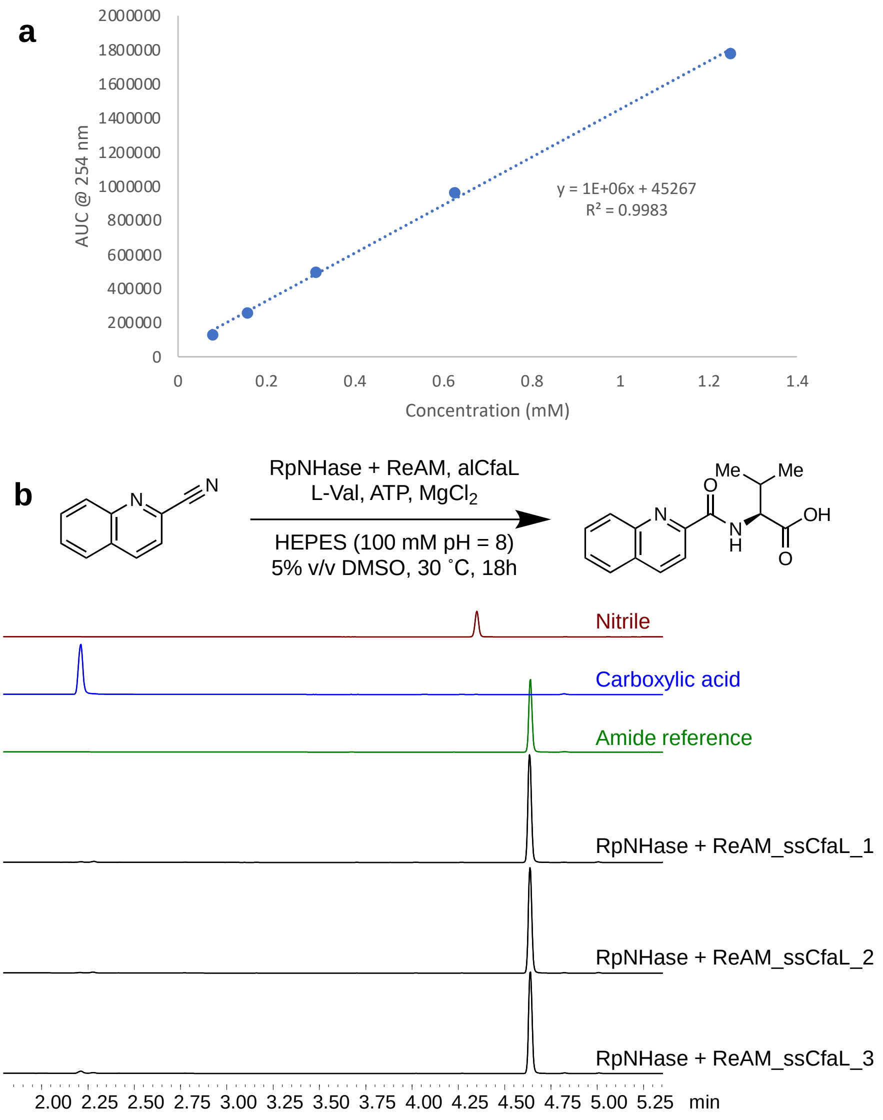

Figure S24. Enzymatic cascade reaction for the synthesis of 18. a, HPLC calibration using amide synthetic standard. **b,** Reaction scheme and HPLC chromatogram (HPLC method A). Traces recorded at 254 nm. Prepared according to the general method 1 using quinoline-2-carbonitrile and L-valine employing RpNHase, ReAM and SsCfaL. HPLC yield: run1 = 95%, run2 = 98%, run3 > 99%. Average: 98 ± 2% (triplicate).

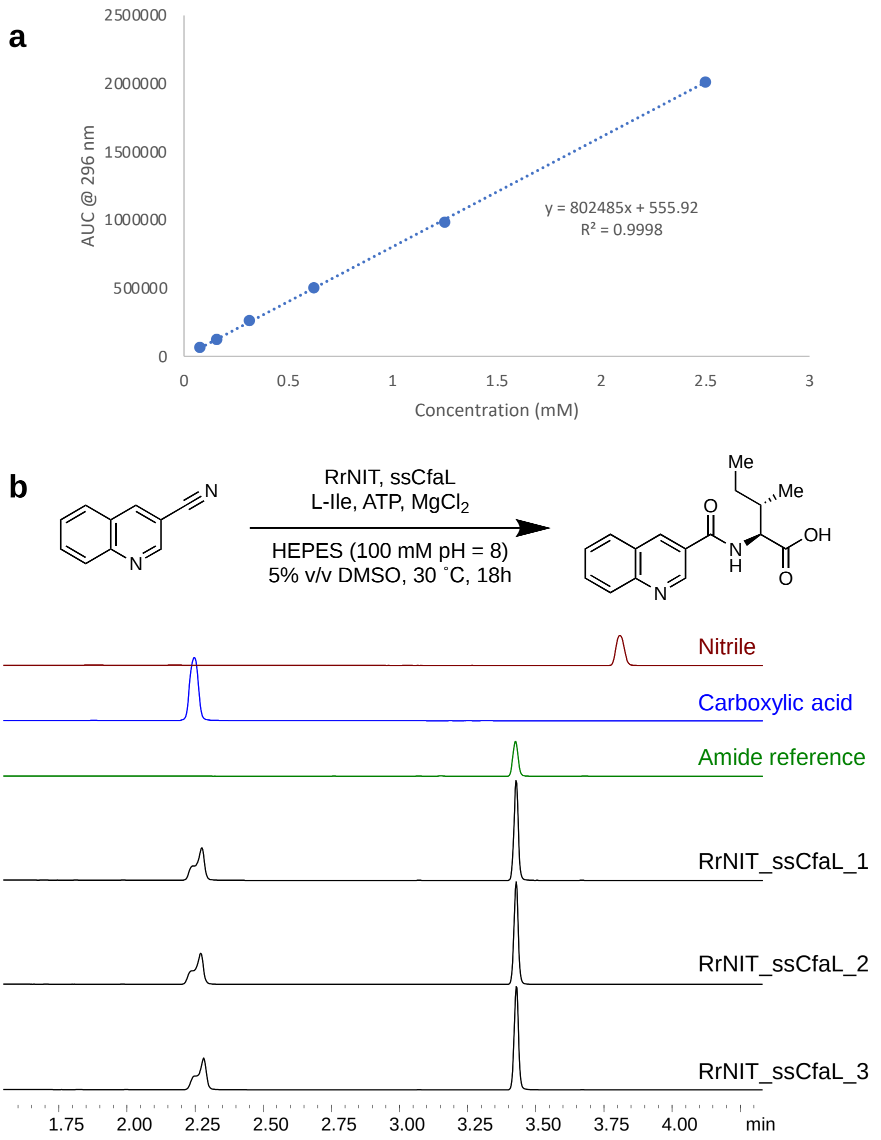

Figure S25. Enzymatic cascade reaction for the synthesis of 19. a, HPLC calibration using amide synthetic standard. **b,** Reaction scheme and HPLC chromatogram HPLC (method A). Traces recorded at 296 nm. Prepared according to the general method 1 using quinoline-3-carbonitrile and L-isoleucine employing RrNIT and SsCfaL. HPLC yield: run1 = 48%, run2 = 49%, run3 = 52%. Average: 50 ± 1% (triplicate).

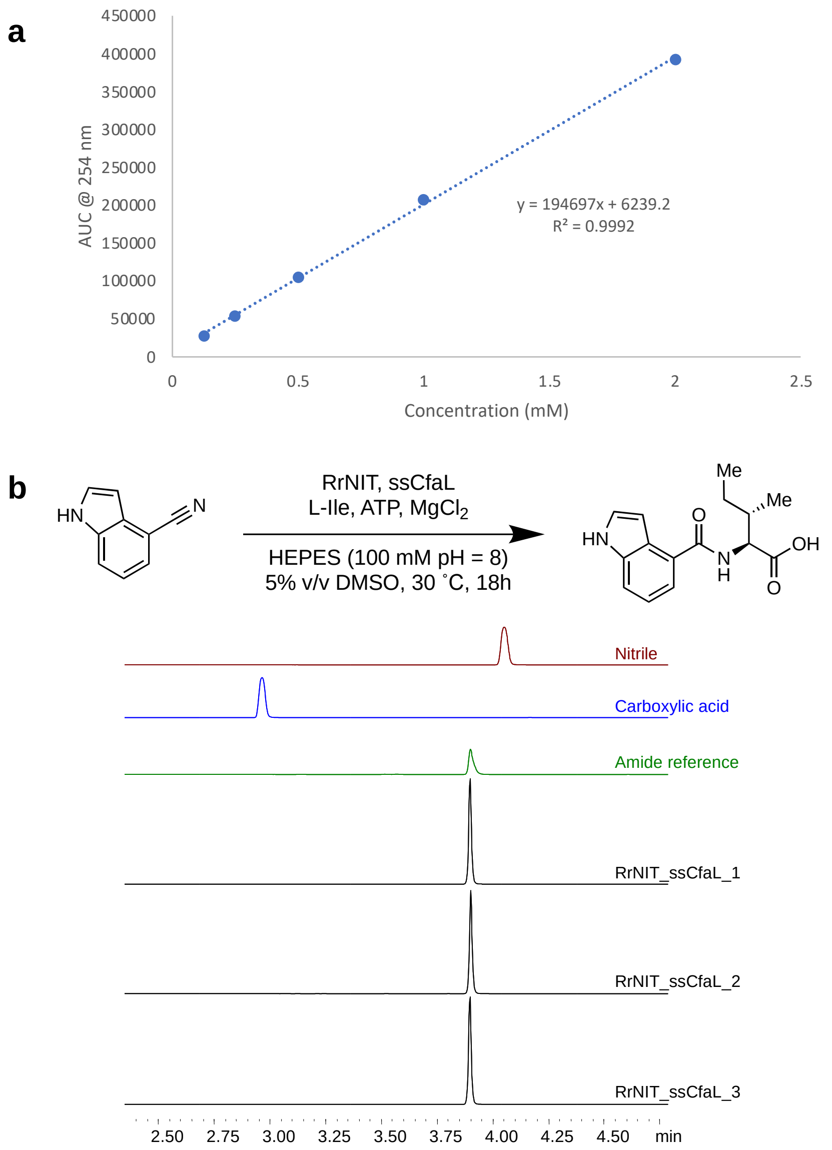

Figure S26. Enzymatic cascade reaction for the synthesis of 20. a, HPLC calibration using amide synthetic standard. **b,** Reaction scheme and HPLC chromatogram (HPLC method A). Traces recorded at 254 nm. Prepared according to the general method 1 using 1H-indole-4-carbonitrile and L-isoleucine employing RrNIT and SsCfaL. HPLC yield: run1 = 99%, run2 = 97%, run3 =101%. Average: 99 ± 2% (triplicate).

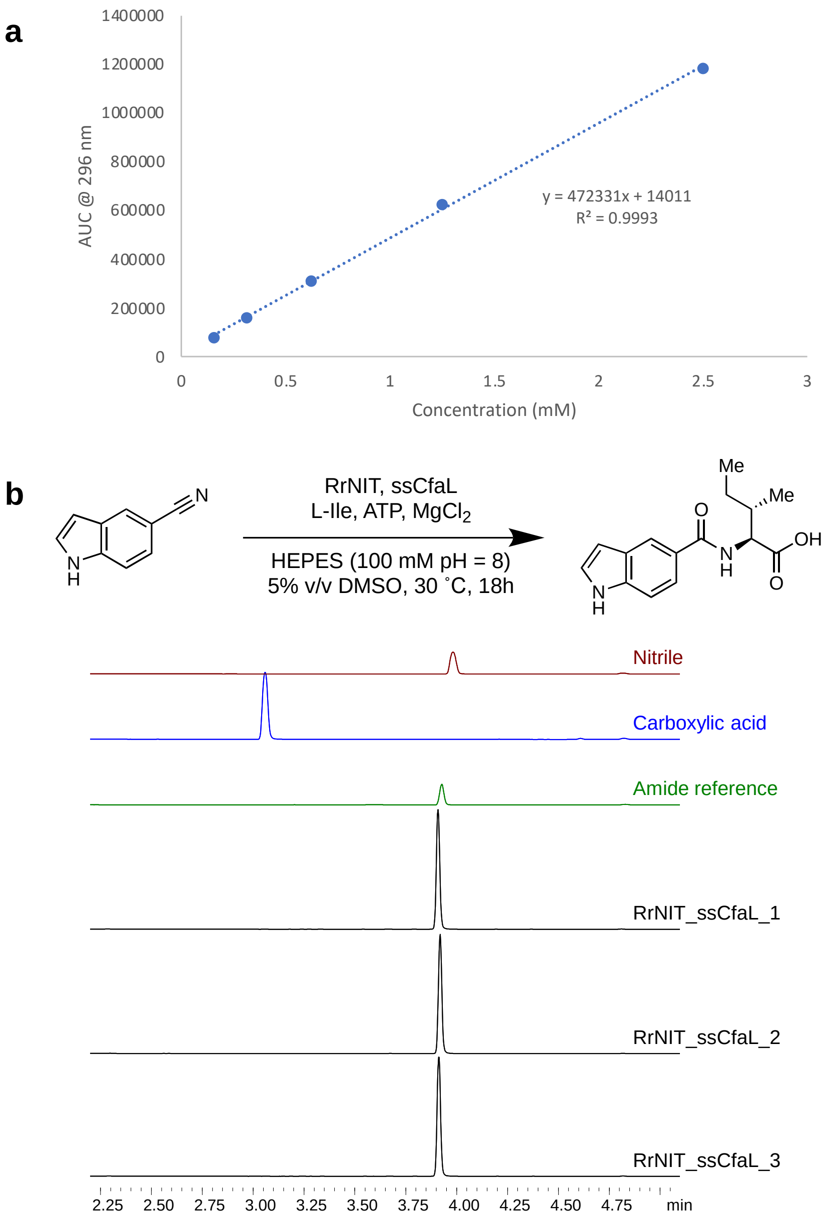

Figure S27. Enzymatic cascade reaction for the synthesis of 21. a, HPLC calibration using amide synthetic standard. **b,** Reaction scheme and HPLC chromatogram (HPLC method A). Traces recorded at 296 nm. Prepared according to the general method 1 using 1H-indole-5-carbonitrile and L-isoleucine employing RrNIT and SsCfaL. HPLC yield: run1 > 99%, run2 > 99%, run3 > 99%. Average: > 99% (triplicate).

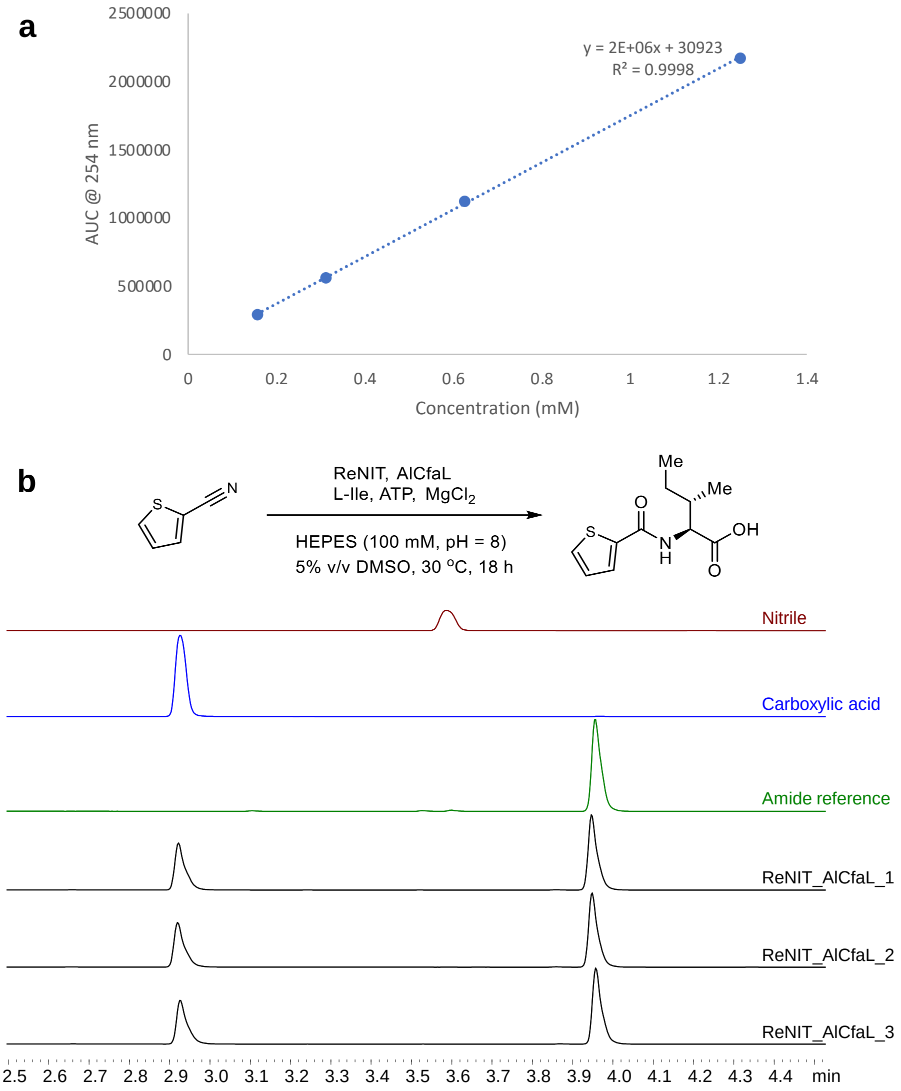

Figure S28. Enzymatic cascade reaction for the synthesis of 22. a, HPLC calibration using amide synthetic standard. **b,** Reaction scheme and HPLC chromatogram (HPLC method A). Traces recorded at 254 nm. Prepared according to the general method 1 using thiophene-2-carbonitrile and L-isoleucine employing ReNIT and AlCfaL. HPLC yield: run1 = 50%, run2 = 49%, run3 = 51%. Average: 50 ± 1% (triplicate).

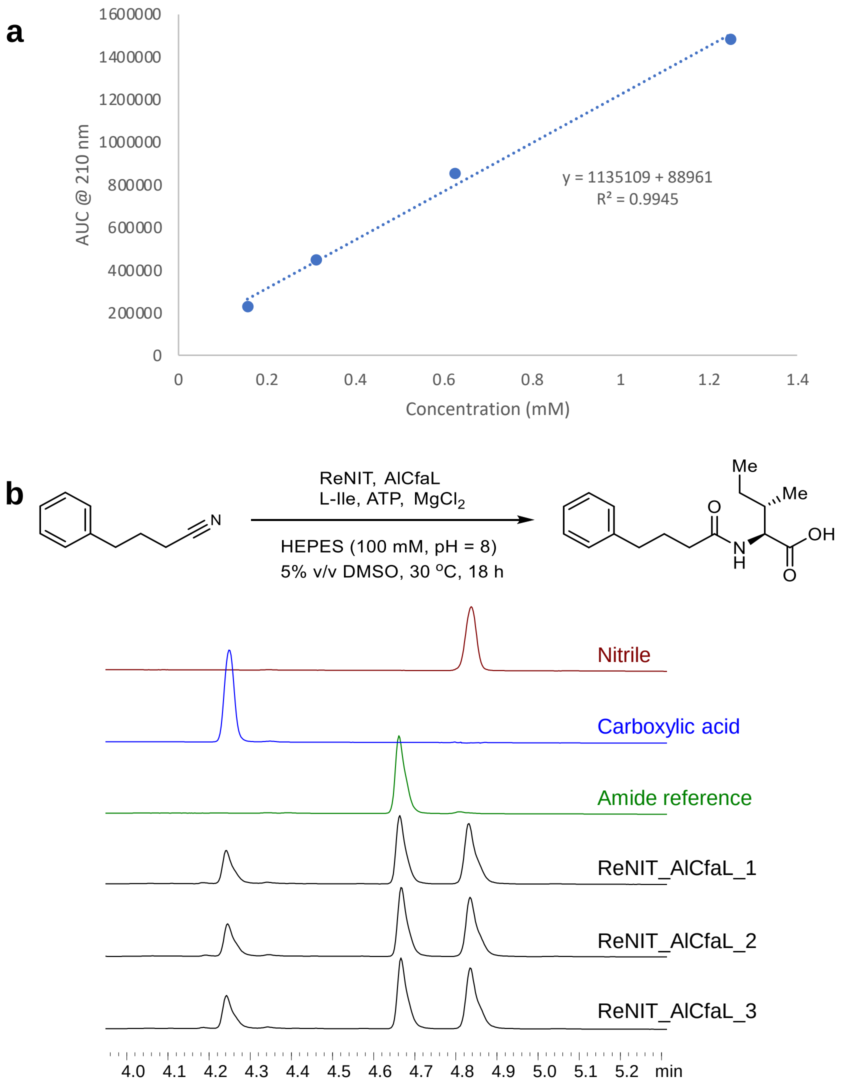

Figure S29. Enzymatic cascade reaction for the synthesis of 23. a, HPLC calibration using amide synthetic standard. **b,** Reaction scheme and HPLC chromatogram (method A). Traces recorded at 210 nm. Prepared according to the general method 1 using 4-phenylbutanenitrile and L-isoleucine employing ReNIT and AlCfaL. HPLC yield: run1 = 29%, run2 = 29%, run3 = 31%. Average: 30 ± 1% (triplicate).

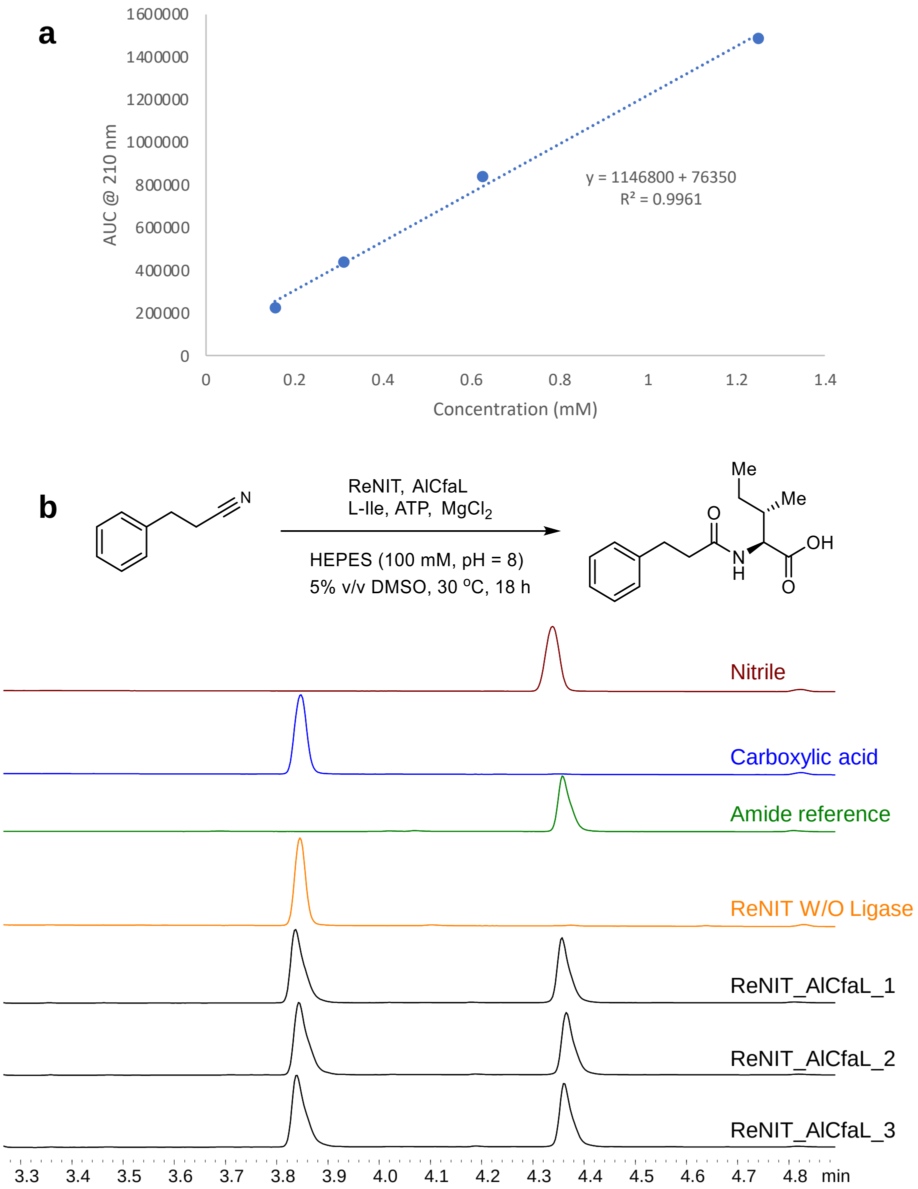

Figure S30. Enzymatic cascade reaction for the synthesis of 24. a, HPLC calibration using amide synthetic standard. **b,** Reaction scheme and HPLC chromatogram (HPLC method A). Traces recorded at 210 nm. Prepared according to the general method 1 using 3-phenylpropanenitrile and L-isoleucine employing ReNIT and AlCfaL. HPLC yield: run1 = 41%, run2 = 39%, run3 = 41%. Average: 40 ± 1% (triplicate).

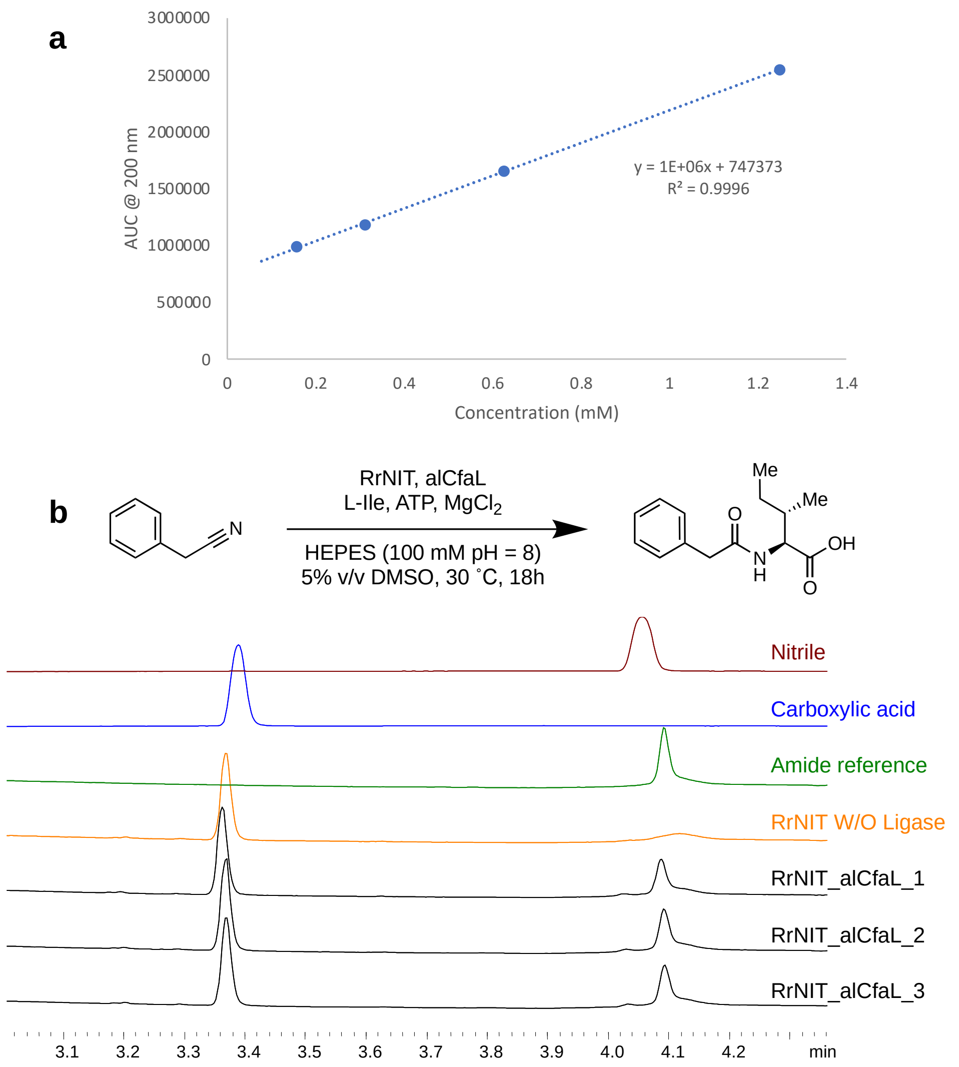

Figure S31. Enzymatic cascade reaction for the synthesis of 25. a, HPLC calibration using amide synthetic standard**. b,** Reaction scheme and HPLC chromatogram (HPLC method A). Traces recorded at 200 nm. Prepared according to the general method 1 using 2-phenylacetonitrile and L-isoleucine employing RrNIT and AlCfaL. HPLC yield: run1 = 21%, run2 = 23%, run3 = 24%. Average: 22 ± 1% (triplicate).

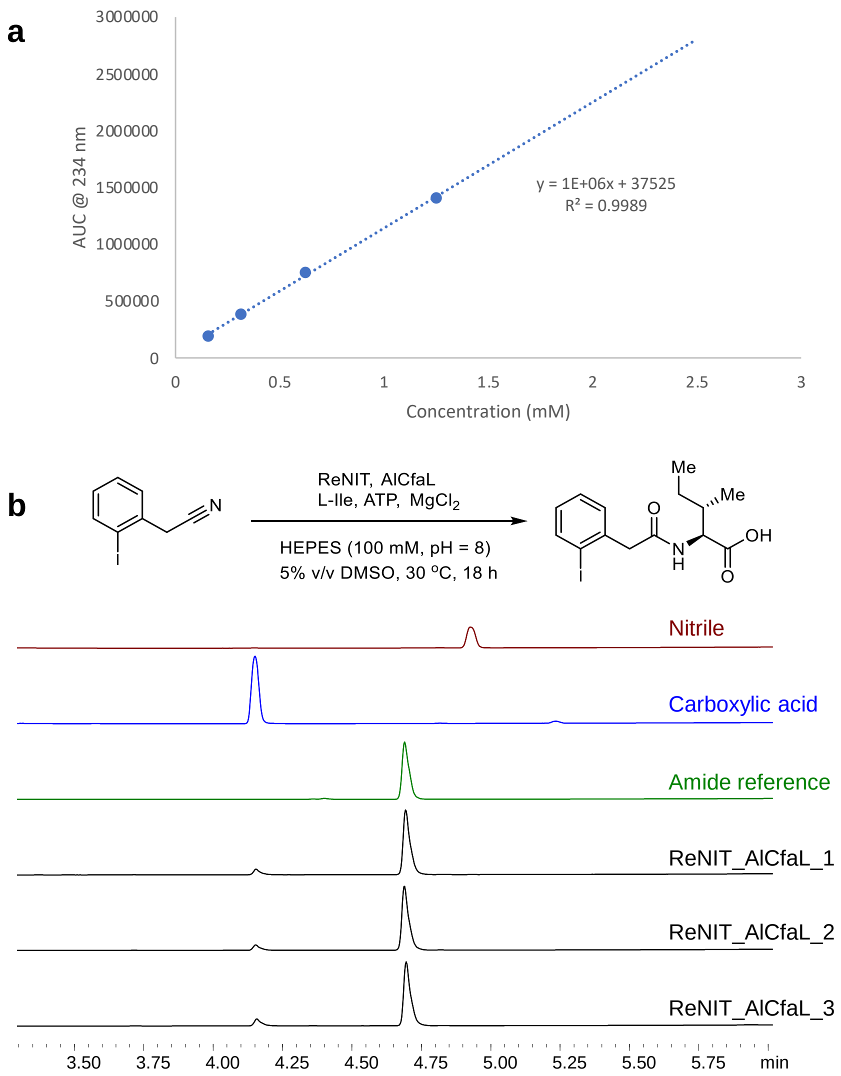

Figure S32. Enzymatic cascade reaction for the synthesis of 26. a, HPLC calibration using amide synthetic standard. **b,** Reaction scheme and HPLC chromatogram (HPLC method A). Traces recorded at 234 nm. Prepared according to the general method 1 using 2-(2-iodophenyl)acetonitrile and L-isoleucine employing ReNIT and AlCfaL. HPLC yield: run1 = 70%, run2 = 69%, run3 = 69%. Average: 69 ± 1% (triplicate).

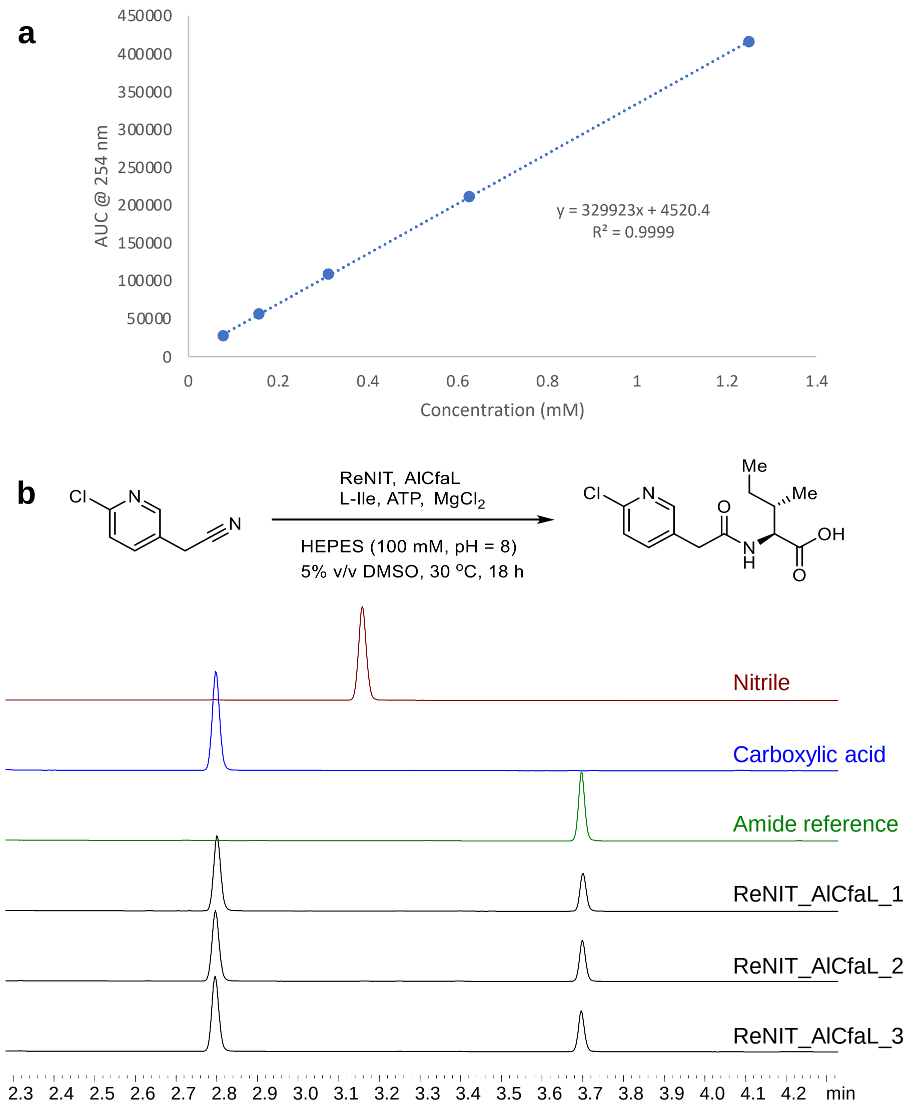

Figure S33. Enzymatic cascade reaction for the synthesis of 27. a, HPLC calibration using amide synthetic standard. **b,** Reaction scheme and HPLC chromatogram (HPLC method A). Traces recorded at 254 nm. Prepared according to the general method 1 using 2-(6-chloropyridin-3-yl)acetonitrile and L-isoleucine employing ReNIT and AlCfaL. HPLC yield: run1 = 34%, run2 = 36%, run3 = 36%. Average: 36 ± 1% (triplicate).

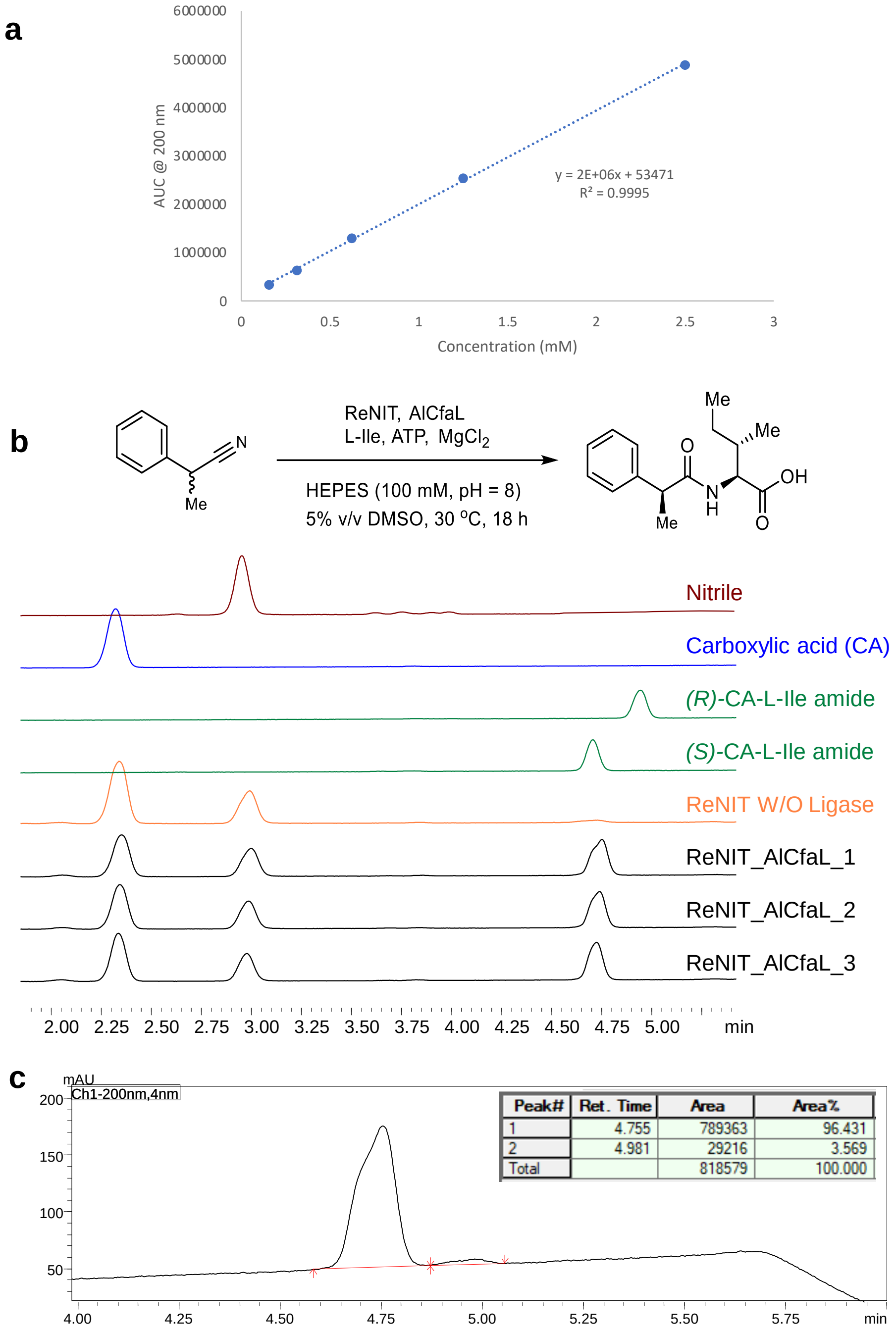

Peak#	Ret. Time	Area	Area%
1	4.755	789363	96.431
2	4.981	29216	3.569
Total		818579	100.000

Figure S34. Enzymatic cascade reaction for the synthesis of 28. a, HPLC calibration using amide synthetic standard. **b,** Reaction scheme and HPLC chromatogram (HPLC method B). **c,** Zoomed-in HPLC chromatogram showing the diastereomeric ratio of the product. Traces recorded at 200 nm. Prepared according to the general method 1 using 2-phenylpropanenitrile and L-isoleucine employing ReNIT and AlCfaL. HPLC yield: run1 = 18%, run2 = 19%, run3 = 19%. Average: 19 ± 1%.

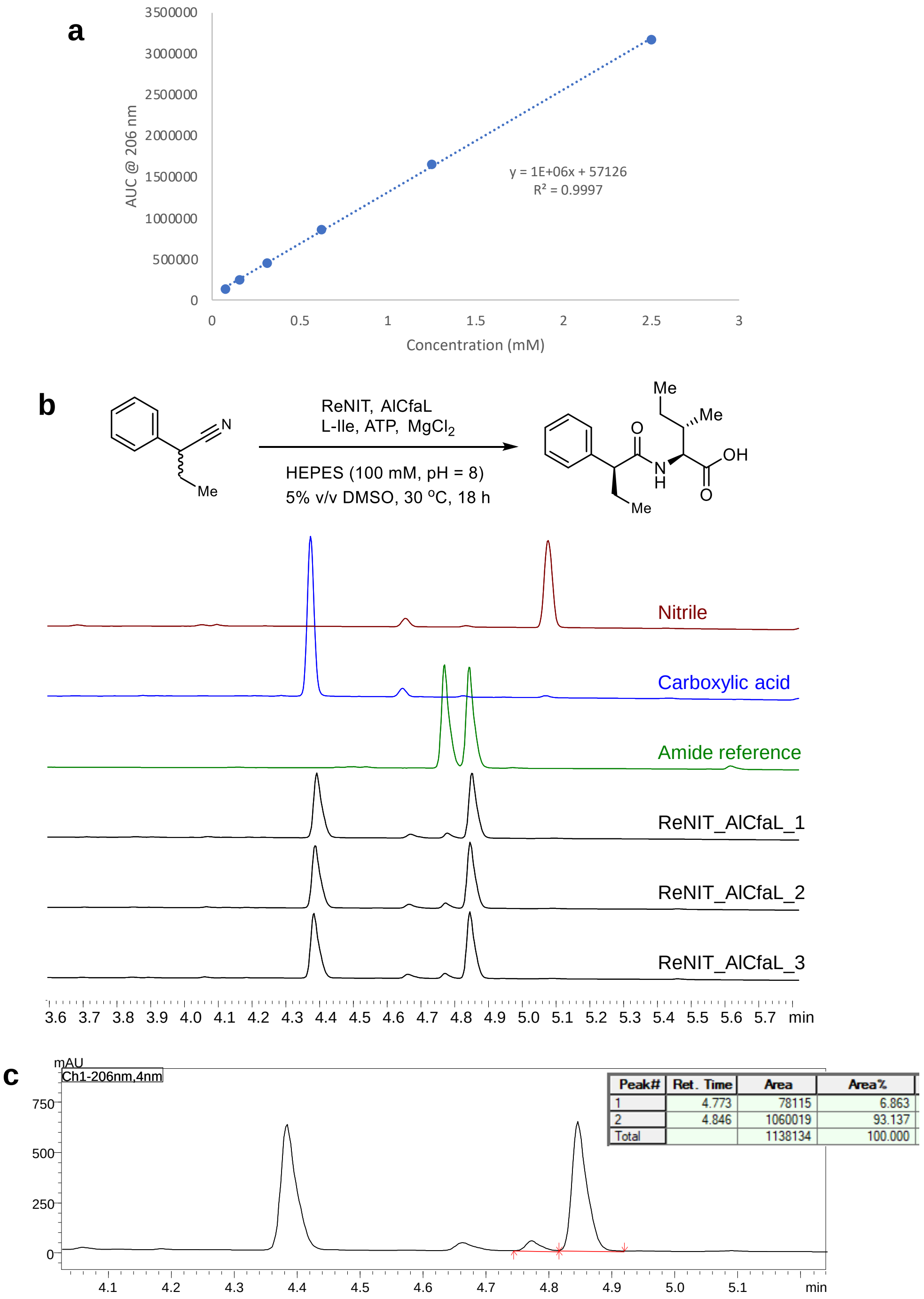

Figure S35. Enzymatic cascade reaction for the synthesis of 29. a, HPLC calibration using amide synthetic standard. **b,** Reaction scheme and HPLC chromatogram (HPLC method A). **c,** Zoomed-in HPLC chromatogram showing the diastereomeric ratio of the product. Traces recorded at 206 nm. Prepared according to the general method 1 using 2-phenylbutanenitrile and L-isoleucine employing ReNIT and AlCfaL. HPLC yield: run1 = 42%, run2 = 43%, run3 = 43%. Average: 43 ± 1%.

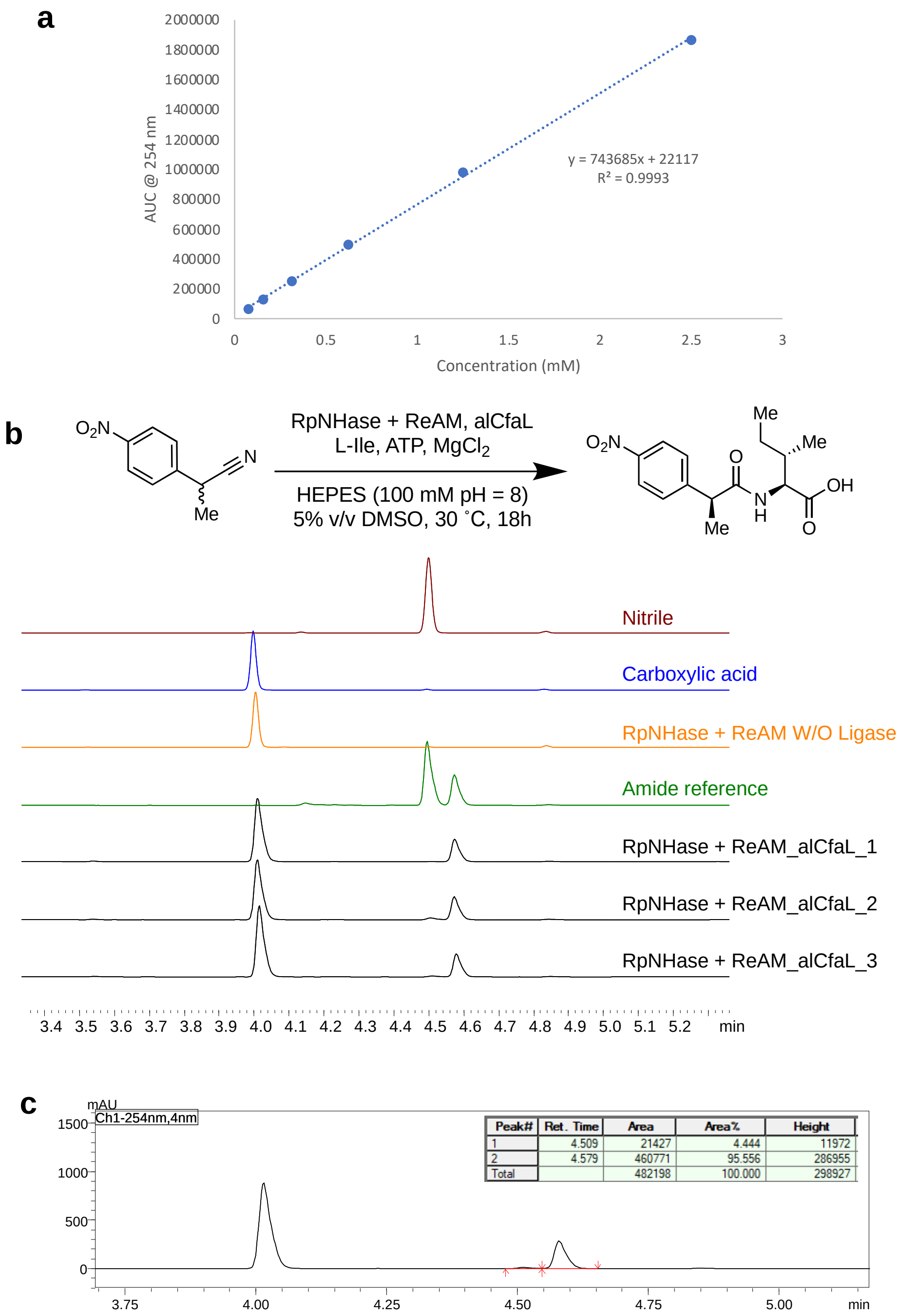

Peak#	Ret. Time	Area	Area%	Height
1	4.509	21427	4.444	11972
2	4.579	460771	95.556	286955
Total		482198	100.000	298927

Figure S36. Enzymatic cascade reaction for the synthesis of 30. a, HPLC calibration using amide synthetic standard. **b,** Reaction scheme and HPLC chromatogram (HPLC method A). **c,** Zoomed-in HPLC chromatogram showing the diastereomeric ratio of the product. Traces recorded at 254 nm. Prepared according to the general method 1 using 2-(4-nitrophenyl)propanenitrile and L-isoleucine employing RpNHase, ReAM and AlCfaL. HPLC yield: run1 = 30%, run2 = 30%, run3 = 33%. Average: 31 ± 1%.

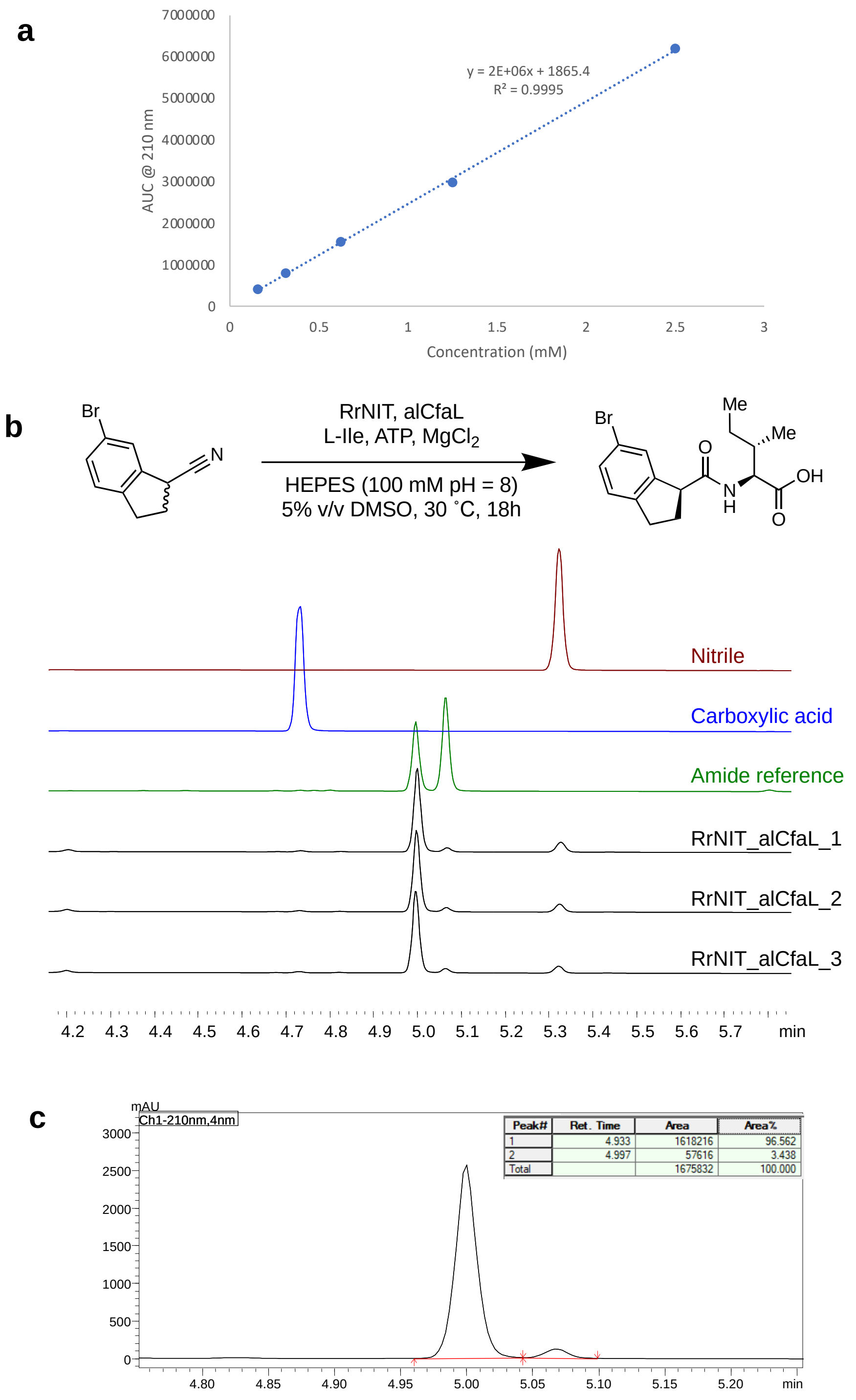

Figure S37. Enzymatic cascade reaction for the synthesis of 31. a, HPLC calibration using amide synthetic standard. **b,** Reaction scheme and HPLC chromatogram (HPLC method A). **c,** Zoomed-in HPLC chromatogram showing the diastereomeric ratio of the product. Traces recorded at 210 nm. Prepared according to the general method 1 using 6-bromo-2,3-dihydro-1H-indene-1-carbonitrile and L-isoleucine employing RrNIT and AlCfaL. HPLC yield: run1 = 54%, run2 = 54%, run3 = 57%. Average: 55 ± 2%.

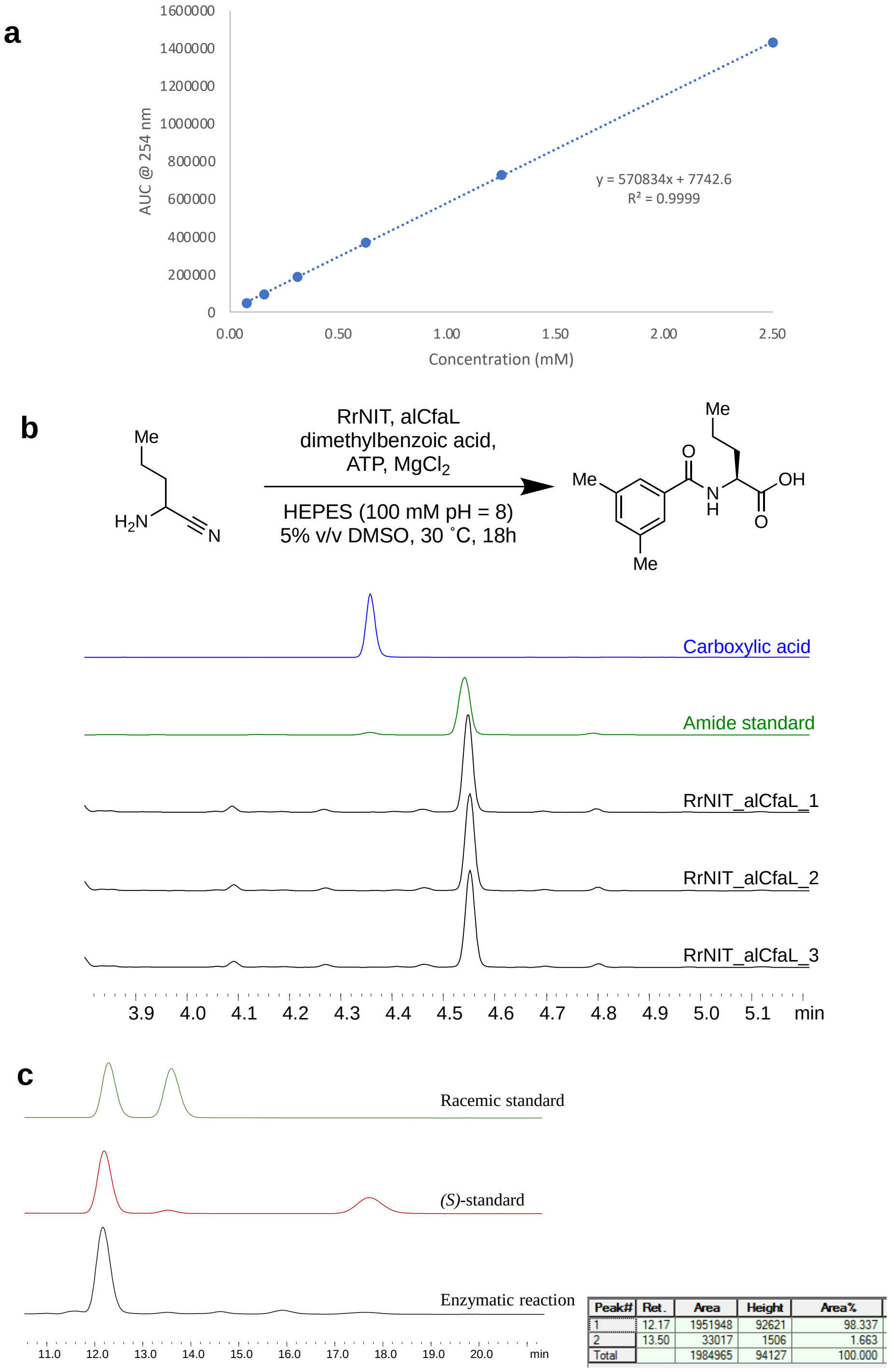

Peak#	Ret.	Area	Height	Area%
1	12.17	1951948	92621	98.337
2	13.50	33017	1506	1.663
Total		1984965	94127	100.000

Figure S38. Enzymatic cascade reaction for the synthesis of 32. a, HPLC calibration using amide synthetic standard. **b,** Reaction scheme and HPLC chromatogram (HPLC method A). **c,** Chiral HPLC analysis of the amide product (HPLC method C, 30% B). Traces recorded at 254 nm. Prepared according to the general method 2 using dimethyl benzoic acid and **54** employing RrNIT and AlCfaL. HPLC yield: run1 = 93%, run2 = 92%, run3 = 92%. Average: 92 ± 1%.

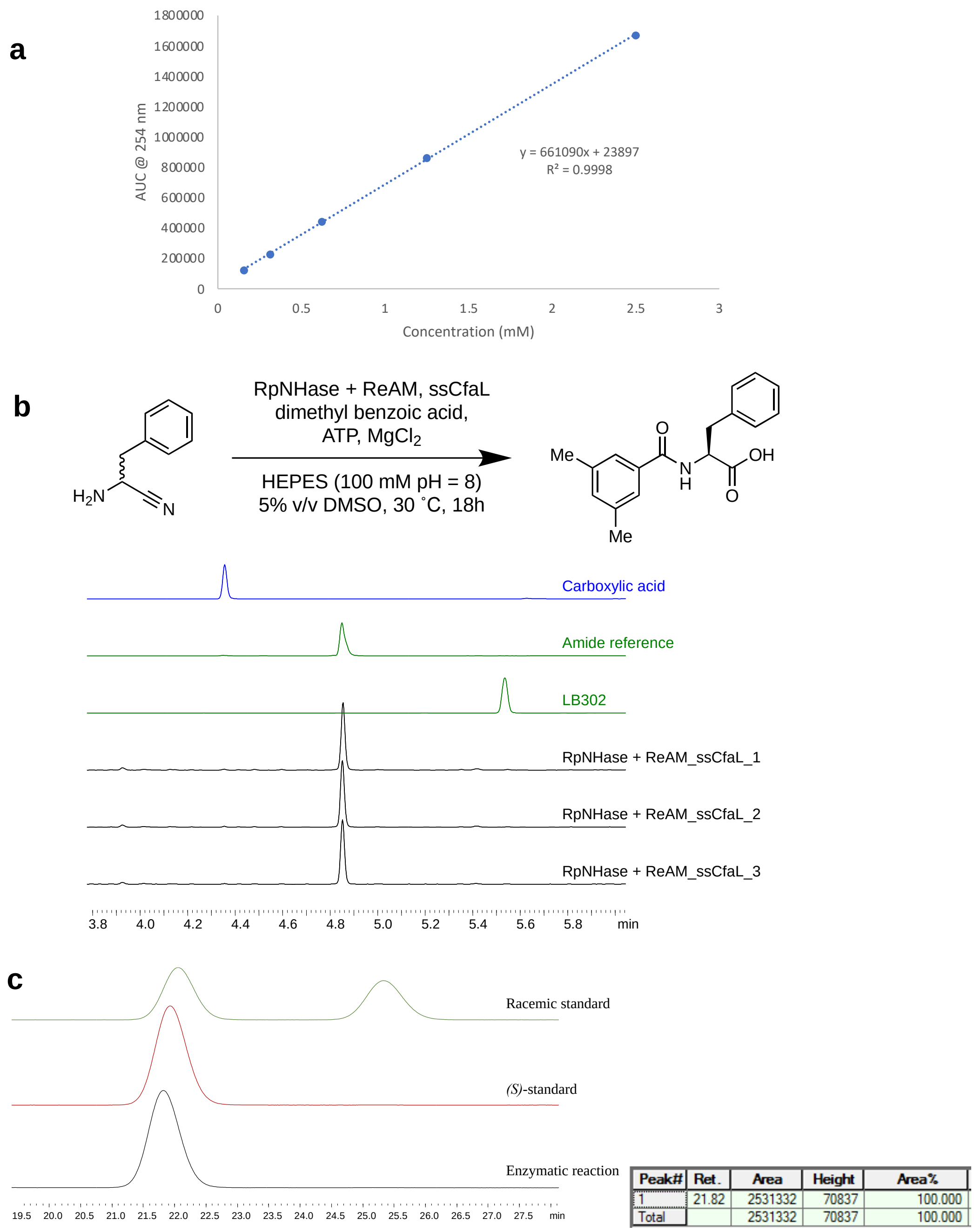

Peak#	Ret.	Area	Height	Area%
1	21.82	2531332	70837	100.000
Total		2531332	70837	100.000

Figure S39. Enzymatic cascade reaction for the synthesis of 33. a, HPLC calibration using amide synthetic standard. **b,** Reaction scheme and HPLC chromatogram (HPLC method A). **c,** Chiral HPLC analysis of the amide product (HPLC method C, 30% B). Traces recorded at 254 nm. Prepared according to the general method 2 using dimethyl benzoic acid and **55** employing RpNHase, ReAM and SsCfaL. HPLC yield: run1 = 94%, run2 = 94%, run3 = 91%. Average: 93 ± 1%.

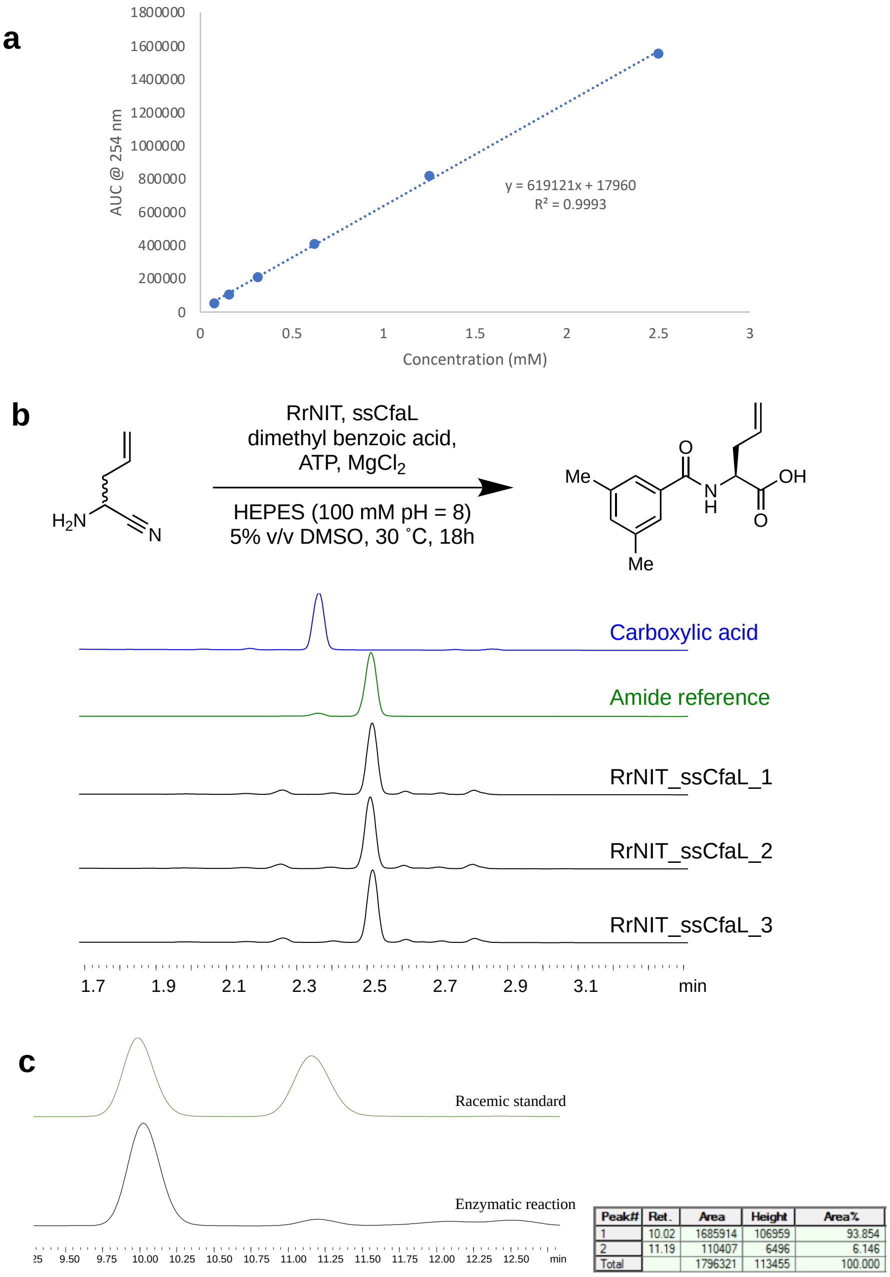

Peak#	Ret.	Area	Height	Area%
1	10.02	1685914	106959	93.854
2	11.19	110407	6496	6.146
Total		1796321	113455	100.000

Figure S40. Enzymatic cascade reaction for the synthesis of 34. a, HPLC calibration using amide synthetic standard. **b,** Reaction scheme and HPLC chromatogram (HPLC method A). **c,** Chiral HPLC analysis of the amide product (HPLC method C, 30% B). Traces recorded at 254 nm. Prepared according to the general method 2 using dimethyl benzoic acid and **56** employing RrNIT and SsCfaL. HPLC yield: run1 = 80%, run2 = 82%, run3 = 82%. Average: 81 ± 1%.

a

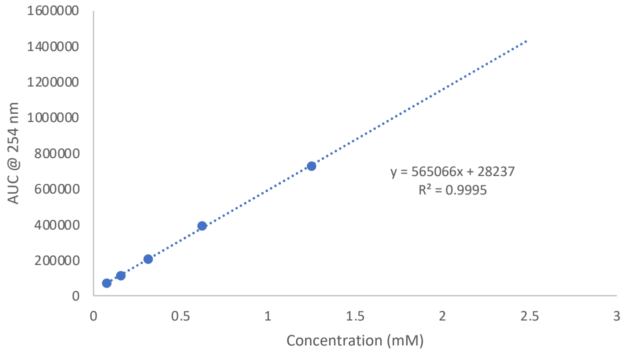

b

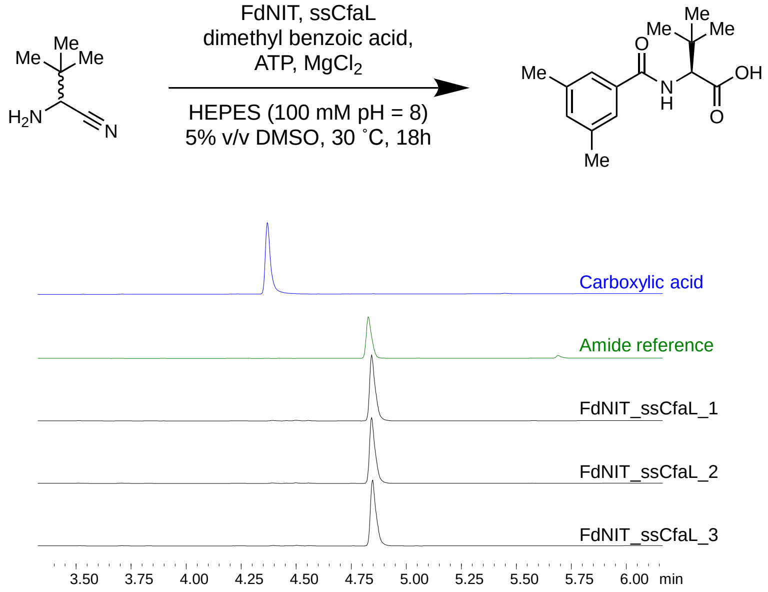

c

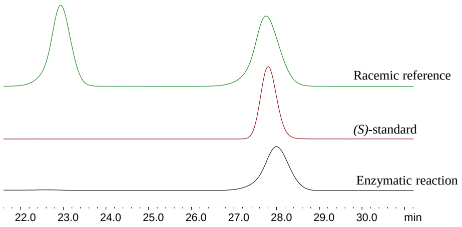

Peak#	Ret. Time	Area	Area%
1	16.528	4147224	99.525
2	18.361	19813	0.475
Total		4167037	100.000

Figure S41. Enzymatic cascade for the synthesis of 35. a, HPLC calibration using amide synthetic standard. **b,** Reaction scheme and HPLC chromatogram (HPLC method A). **c,** Chiral HPLC analysis of the amide product (HPLC method C, 30% B). Traces recorded at 254 nm. Prepared according to the general method 2 using dimethyl benzoic acid and **57** employing FdNIT and SsCfaL. HPLC yield: run1 > 99%, run2 > 99%, run3 > 99%. Average: > 99%.

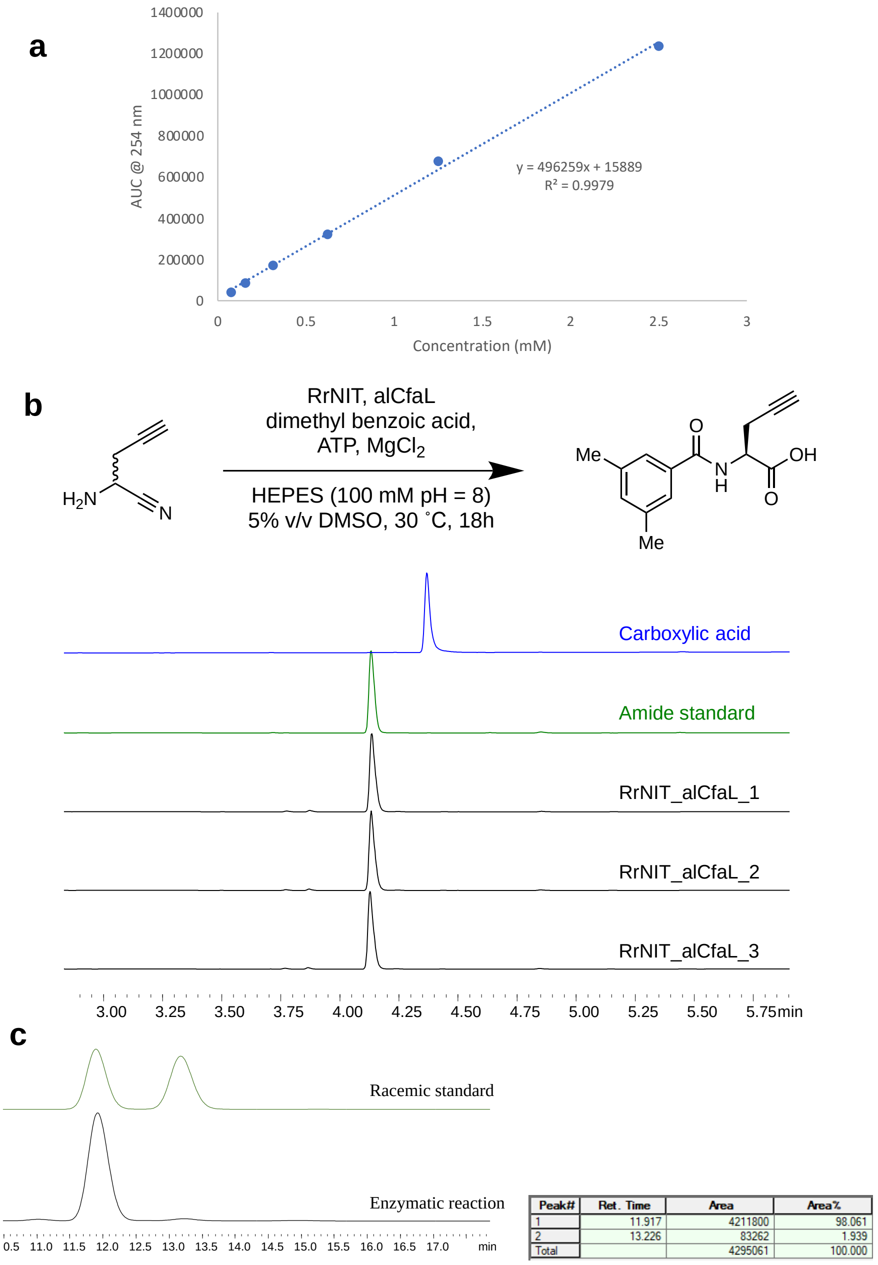

Peak#	Ret. Time	Area	Area%
1	11.917	4211800	98.061
2	13.226	83262	1.939
Total		4295061	100.000

Figure S42. Enzymatic cascade reaction for the synthesis of 36. a, HPLC calibration using amide synthetic standard. **b,** Reaction scheme and HPLC chromatogram (HPLC method A). **c,** Chiral HPLC analysis of the amide product (HPLC method C, 25% B). Traces recorded at 254 nm. Prepared according to the general method 2 using dimethyl benzoic acid and **58** employing RrNIT and AlCfaL. HPLC yield: run1 > 99%, run2 > 99%, run3 > 99%. Average: > 99%.

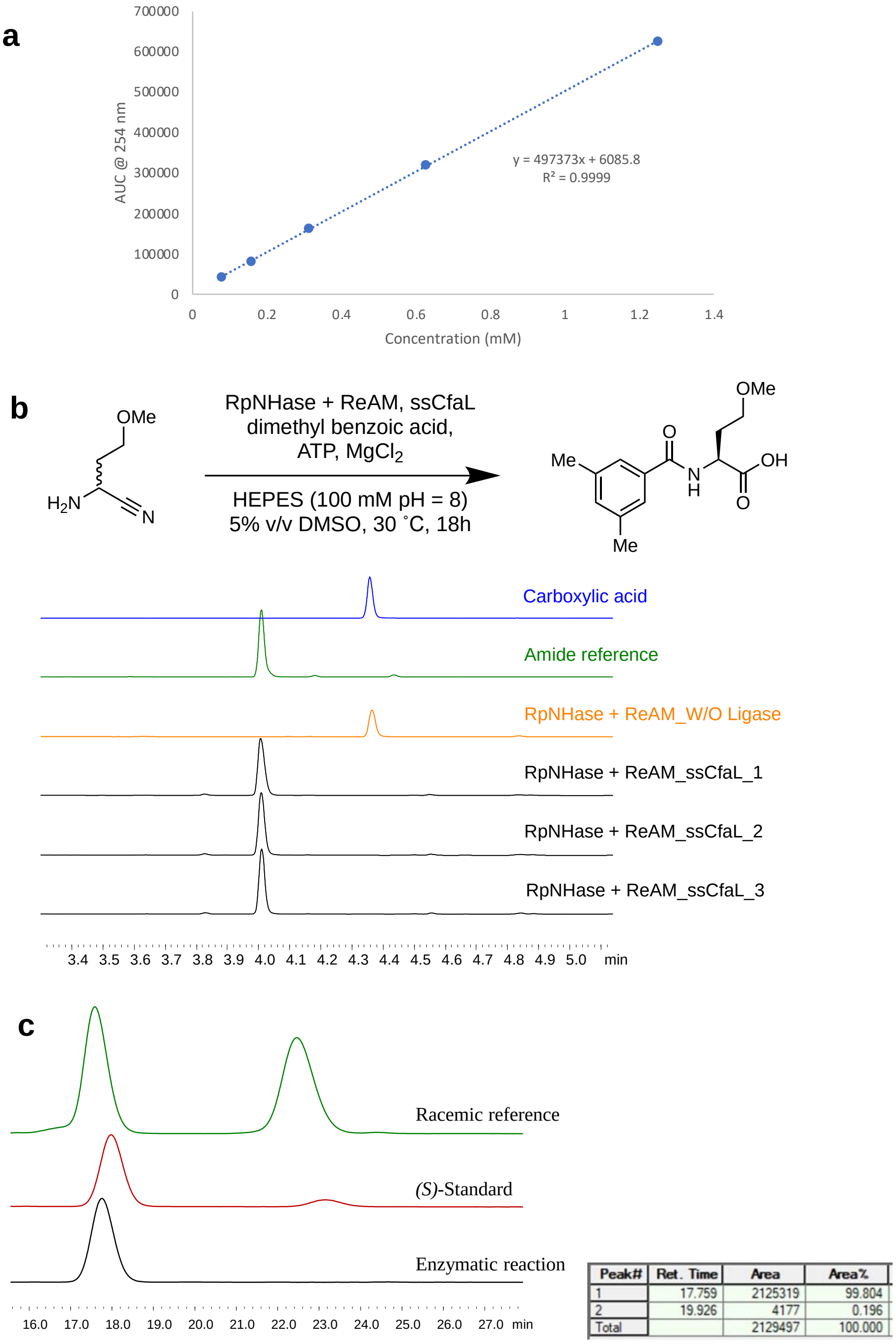

Peak#	Ret. Time	Area	Area%
1	17.759	2125319	99.804
2	19.926	4177	0.196
Total		2129497	100.000

Figure S43. Enzymatic cascade reaction for the synthesis of 37. a, HPLC calibration using amide synthetic standard. **b,** Reaction scheme and HPLC chromatogram (HPLC method A). **c,** Chiral HPLC analysis of the amide product (HPLC method C, 20% B). Traces recorded at 254 nm. Prepared according to the general method 2 using dimethyl benzoic acid and **59** employing RpNHase, ReAM and SsCfaL. HPLC yield: run1 > 99%, run2 > 99%, run3 > 99%. Average: > 99%.

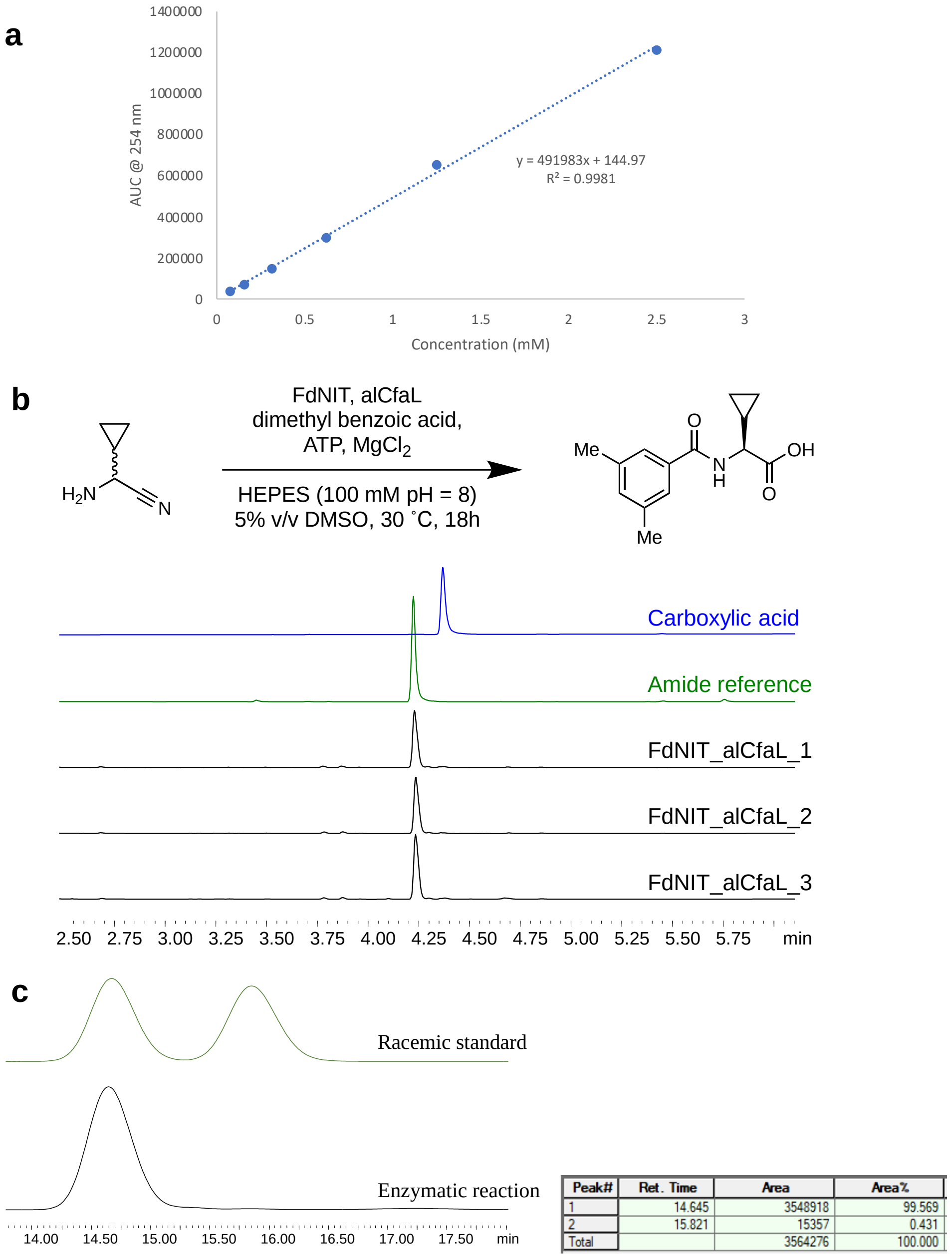

Peak#	Ret. Time	Area	Area%
1	14.645	3548918	99.569
2	15.821	15357	0.431
Total		3564276	100.000

Figure S44. Enzymatic cascade reaction for the synthesis of 38. a, HPLC calibration using amide synthetic standard. **b,** Reaction scheme and HPLC chromatogram (HPLC method A). **c,** Chiral HPLC analysis of the amide product (HPLC method C, 25% B). Traces recorded at 254 nm. Prepared according to the general method 2 using dimethyl benzoic acid and **60** employing FdNIT and AlCfaL. HPLC yield: run1 > 99%, run2 > 99%, run3 > 99%. Average: > 99%.

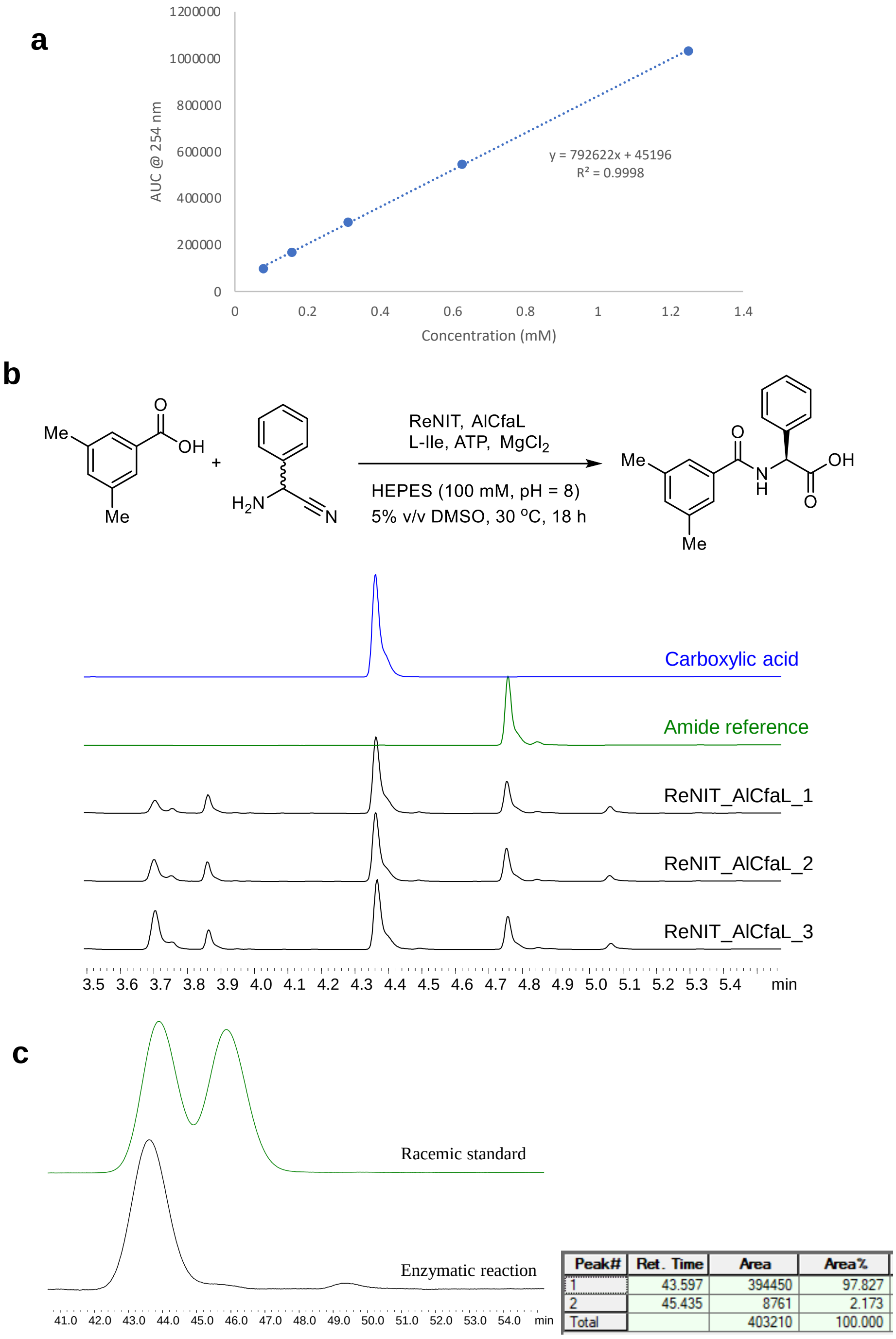

Peak#	Ret. Time	Area	Area%
1	43.597	394450	97.827
2	45.435	8761	2.173
Total		403210	100.000

Figure S45. Enzymatic cascade reaction for the synthesis of 39. a, HPLC calibration using amide synthetic standard. **b,** Reaction scheme and HPLC chromatogram (HPLC method A). **c,** Chiral HPLC analysis of the amide product (HPLC method C, 25% B). Traces recorded at 254 nm. Prepared according to the general method 2 using dimethyl benzoic acid and 2-amino-2-phenylacetonitrile employing ReNIT and AlCfaL. HPLC yield: run1 = 14%, run2 = 14%, run3 = 14%. Average: 14 ± 1%.

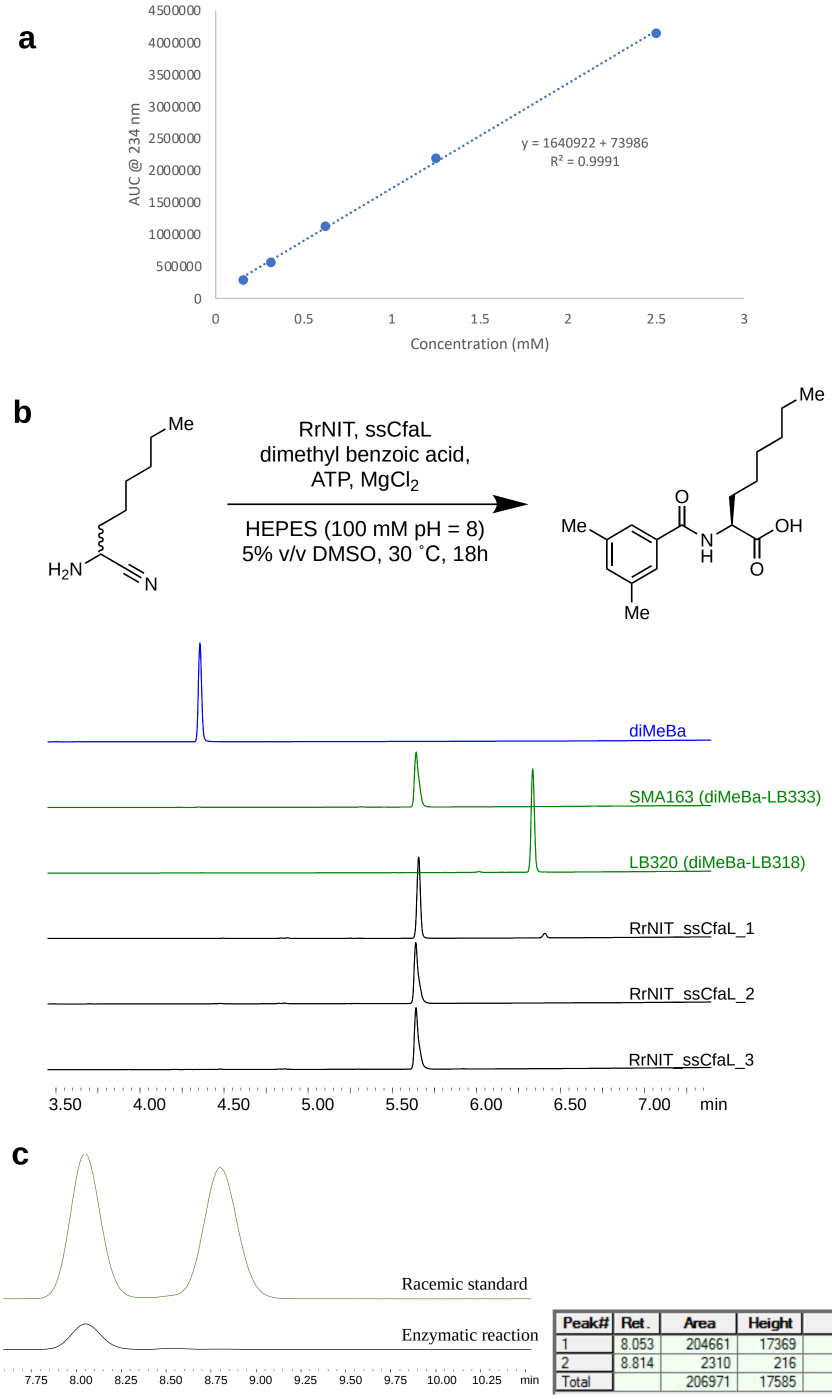

Peak#	Ret.	Area	Height	Area%
1	8.053	204661	17369	98.884
2	8.814	2310	216	1.116
Total		206971	17585	100.000

Figure S46. Enzymatic cascade reaction for the synthesis of 40. a, HPLC calibration using amide synthetic standard. **b,** Reaction scheme and HPLC chromatogram (HPLC method A). **c,** Chiral HPLC analysis of the amide product (HPLC method C, 50% B). Traces recorded at 234 nm. Prepared according to the general method 2 using dimethyl benzoic acid and **61** employing RrNIT and SsCfaL. HPLC yield: run1 = 75%, run2 = 71%, run3 = 71%. Average: 72 ± 2%.

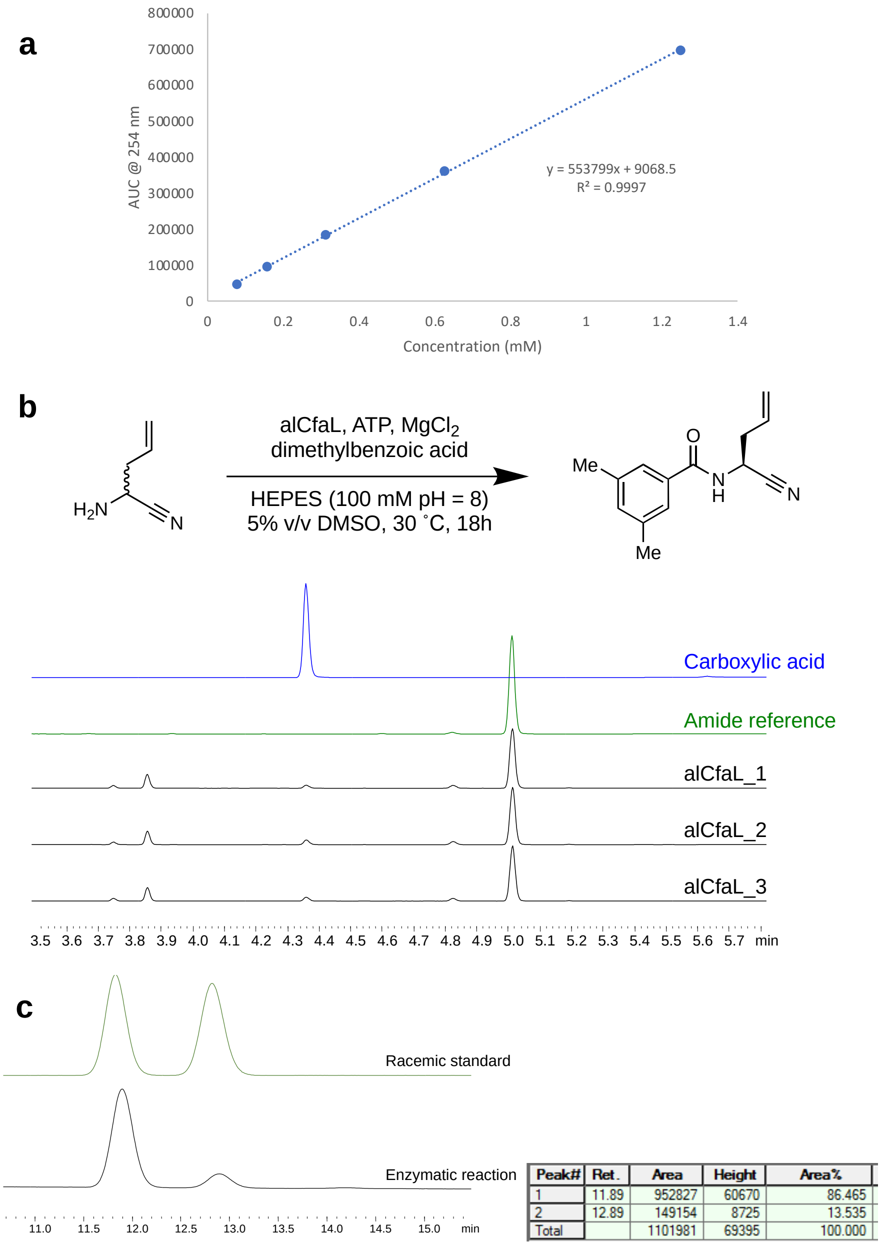

Peak#	Ret.	Area	Height	Area%
1	11.89	952827	60670	86.465
2	12.89	149154	8725	13.535
Total		1101981	69395	100.000

Figure S47. Enzymatic synthesis of 41. a, HPLC calibration using amide synthetic standard. **b,** Reaction scheme and HPLC chromatogram (HPLC method A). **c,** Chiral HPLC analysis of the amide product (HPLC method C). Traces recorded at 254 nm. Prepared according to the general method 2 using dimethyl benzoic acid and **56** employing AlCfaL. HPLC yield: run1 = 37%, run2 = 35%, run3 = 34%. Average: 35 ± 1%.

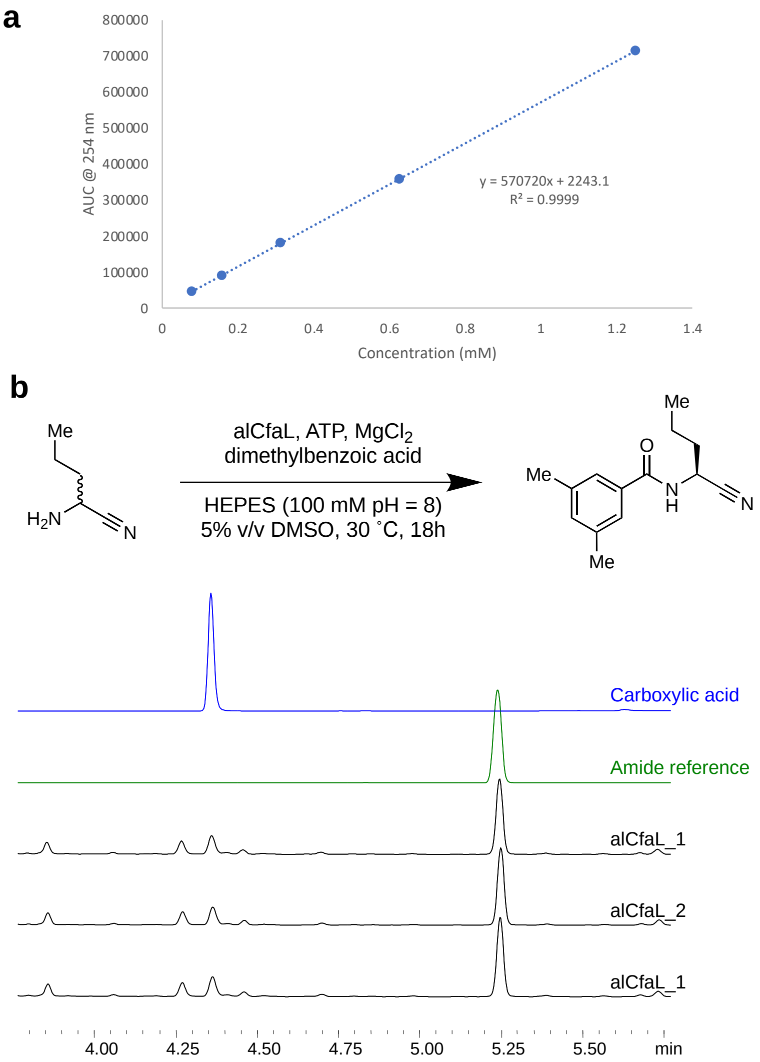

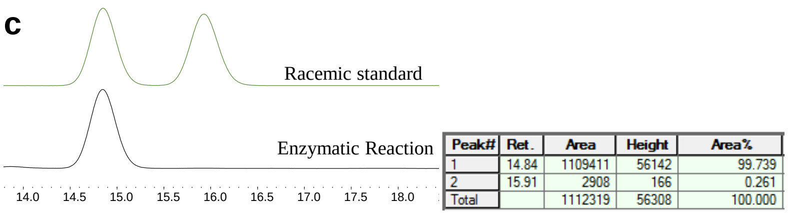

Peak#	Ret.	Area	Height	Area%
1	14.84	1109411	56142	99.739
2	15.91	2908	166	0.261
Total		1112319	56308	100.000

Figure S48. Enzymatic synthesis of 42. a, HPLC calibration using amide synthetic standard. **b,** Reaction scheme for and HPLC chromatogram (HPLC method A). **c,** Chiral HPLC analysis of the amide product (HPLC method C). Traces recorded at 254 nm. Prepared according to the general method 2 using dimethyl benzoic acid and **54** employing AlCfaL. HPLC yield: run1 = 43%, run2 = 45%, run3 = 43%. Average: 44 ± 1%.

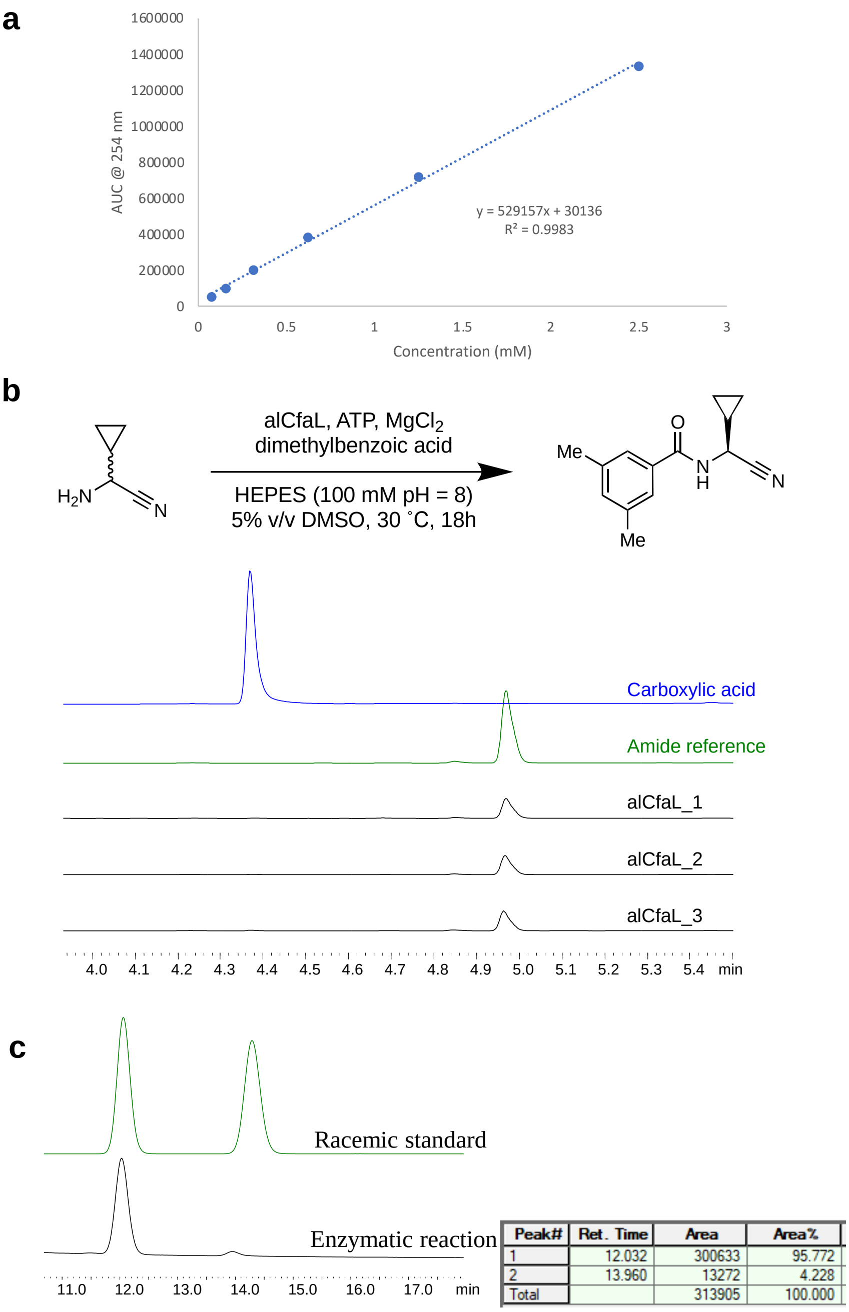

Peak#	Ret. Time	Area	Area%
1	12.032	300633	95.772
2	13.960	13272	4.228
Total		313905	100.000

Figure S49. Enzymatic synthesis of 43. a, HPLC calibration using amide synthetic standard. **b,** Reaction scheme and HPLC chromatogram (HPLC method A). **c,** Chiral HPLC analysis of the amide product (HPLC method C). Traces recorded at 254 nm. Prepared according to the general method 2 using dimethyl benzoic acid and **60** employing AlCfaL. HPLC yield: run1 = 15%, run2 = 15%, run3 = 15%. Average: 15 ± 1%.

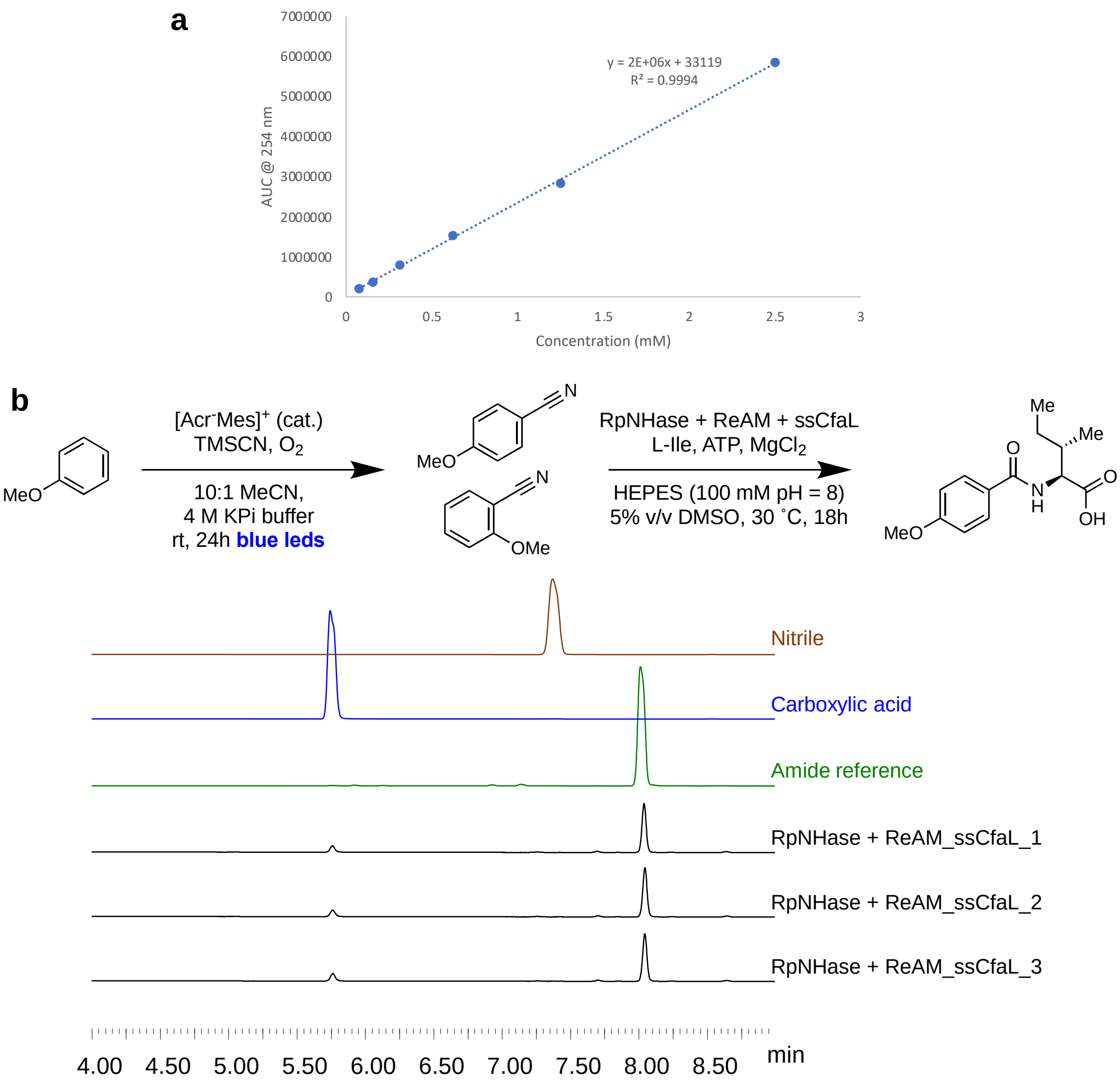

Figure S50. Chemoenzymatic cascade reaction for the synthesis of 1. a, HPLC calibration using amide synthetic standard. **b,** Reaction scheme and HPLC chromatogram (HPLC method A). Traces recorded at 254 nm. Prepared according to the general method 3 using anisole and L-isoleucine employing RpNHase, ReAM and SsCfaL. HPLC yield: run1 = 9%, run2 = 9%, run3 = 9%. Average: 9 ± 1%. Regioisomeric ratio > 9:1 (compound **1**: compound **9**).

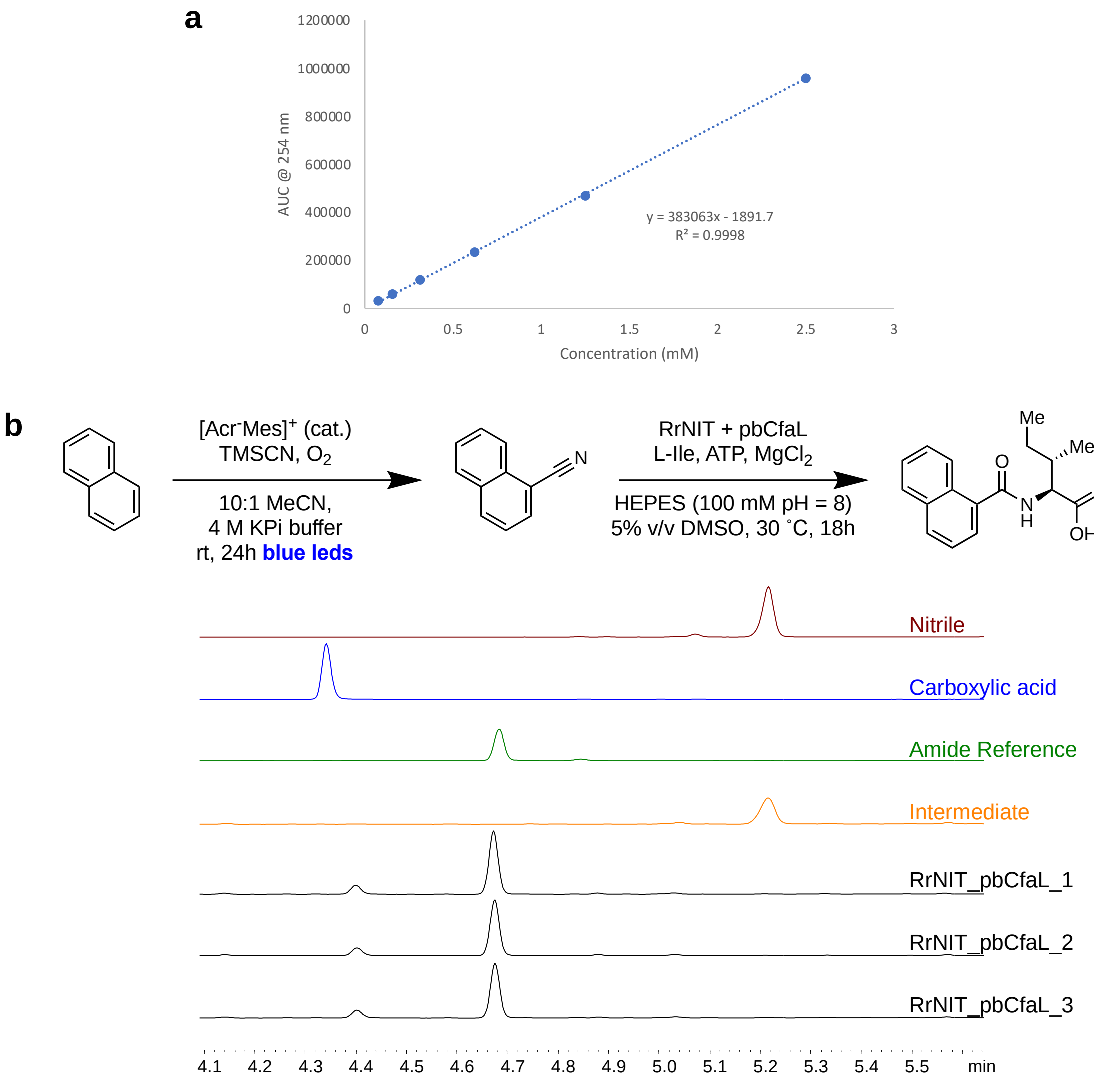

Figure S51. Chemoenzymatic cascade reaction for the synthesis of 13. a, HPLC calibration using amide synthetic standard. **b,** Reaction scheme and HPLC chromatogram (HPLC method A). Traces recorded at 254 nm. Prepared according to the general method 3 employing RrNIT and pbCfaL. HPLC yield: run1 = 56%, run2 = 50%, run3 = 49%. Average: 52 ± 3%.

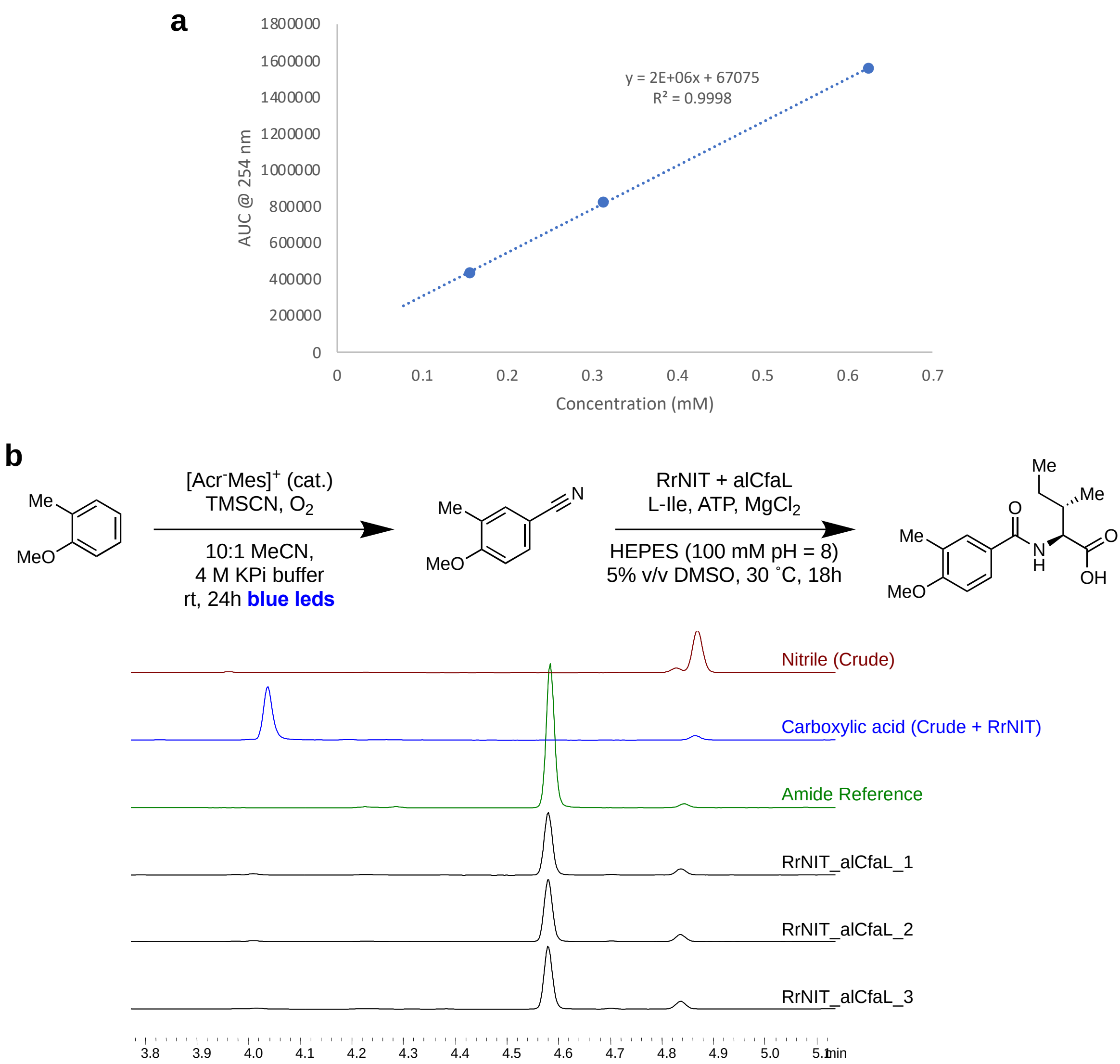

Figure S52. Chemoenzymatic cascade reaction for the synthesis of 44. a, HPLC calibration using amide synthetic standard. **b,** Reaction scheme and HPLC chromatogram (HPLC method A). Traces recorded at 254 nm. Prepared according to the general method 3 using 1-methoxy-2-methylbenzene and L-isoleucine employing RrNIT and AlCfaL. HPLC yield: run1 = 3%, run2 = 3%, run3 = 3%. Average: 3 ± 1%.

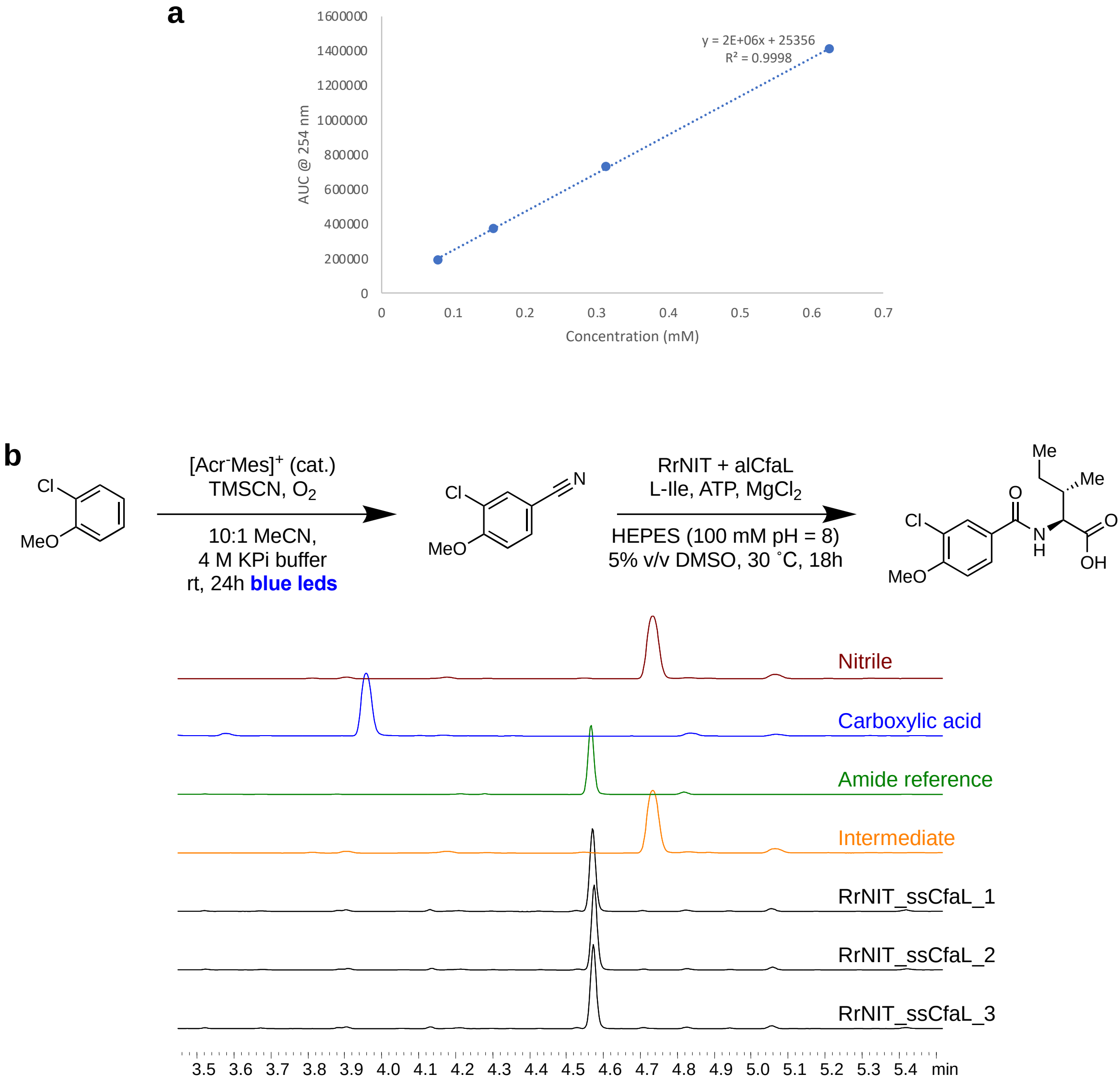

Figure S53. Chemoenzymatic cascade reaction for the synthesis of 45. a, HPLC calibration using amide synthetic standard. **b,** Reaction scheme and HPLC chromatogram (HPLC method A). Traces recorded at 254 nm. Prepared according to the general method 3 using 3-chloro-4-methoxybenzonitrile and L-isoleucine employing RrNIT and SsCfaL. HPLC yield: run1 = 9%, run2 = 9%, run3 = 9%. Average: 9 ± 1%.

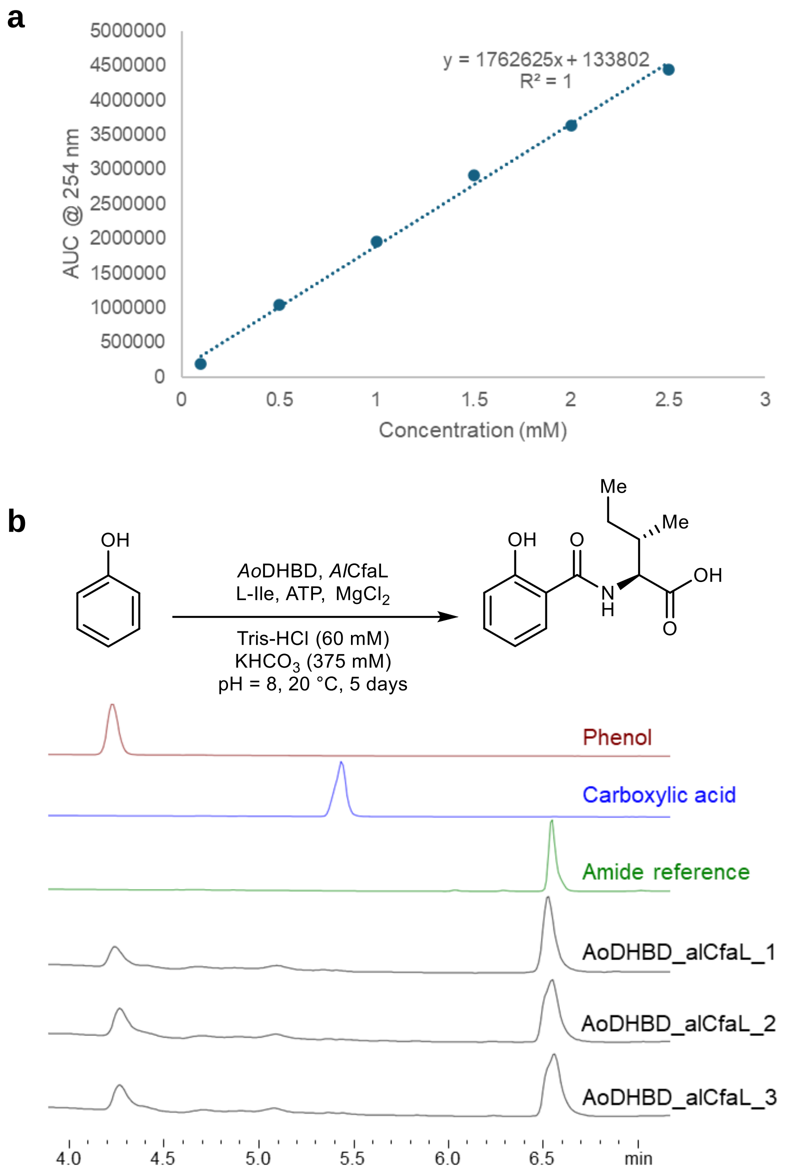

Figure S54. Enzymatic C-H amidation cascade reaction for the synthesis of 46. a, HPLC calibration using synthetic standard. **b,** Reaction scheme and HPLC chromatogram (HPLC method C). Traces recorded at 254 nm. Prepared according to general method 4 using phenol and L-isoleucine employing AoDHBD and AlCfaL. HPLC yield: run1 = 19%, run2 = 19%, run3 = 17%. Average = 18 ± 1% (triplicate).

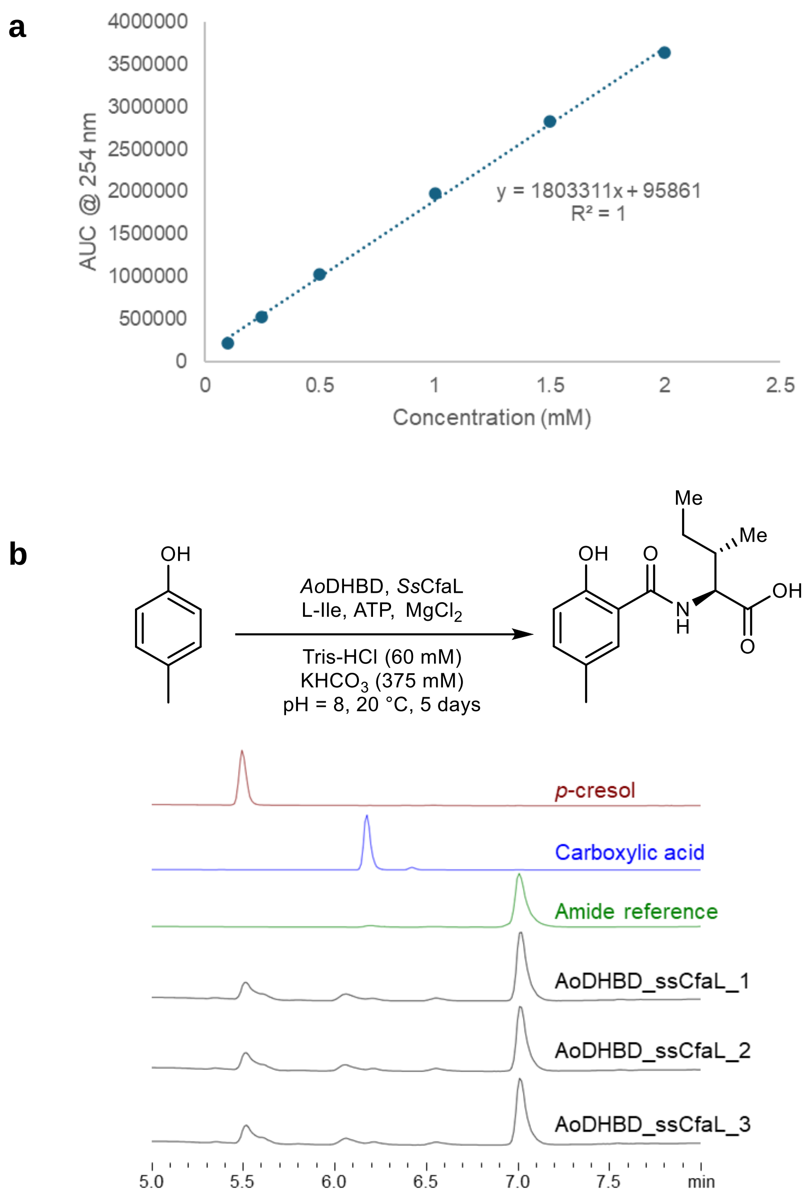

Figure S55. Enzymatic C-H amidation cascade reaction for the synthesis of 47. a, HPLC calibration using amide synthetic standard. **b,** Reaction scheme and HPLC chromatogram (HPLC method C). Traces recorded at 254 nm. Prepared according to general method 4 using *p*-cresol and L-isoleucine employing AoDHBD and AlCfaL. HPLC yield: run1 = 20%, run2 = 19%, run3 = 20%. Average = 20 ± 0.6% (triplicate).

a

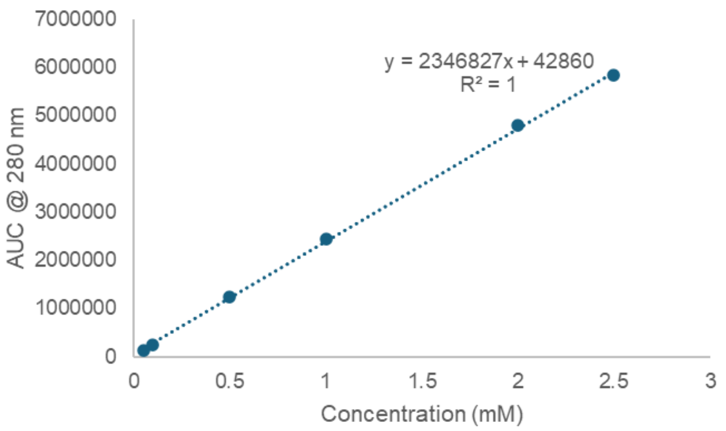

b

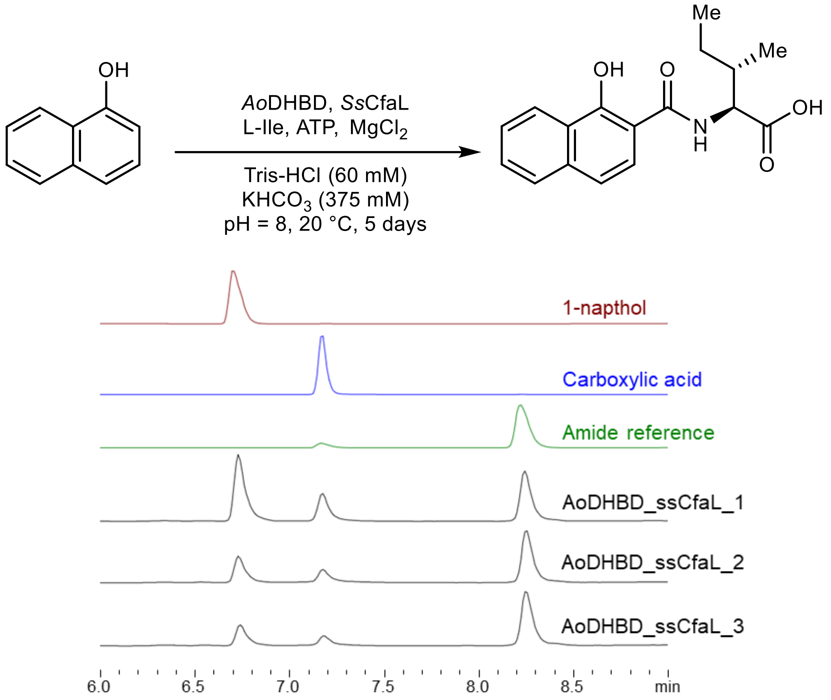

Figure S56. Enzymatic C-H amidation cascade reaction for the synthesis of 48. a, HPLC calibration using synthetic standard. **b,** Reaction scheme and HPLC chromatogram (HPLC method C). Traces recorded at 280 nm. Prepared according to general method 4 using 1-napthol and L-isoleucine employing AoDHBD and SsCfaL. HPLC yield: run1 = 14%, run2 = 14%, run3 = 13%. Average = 13 ± 0.6% (triplicate).

a

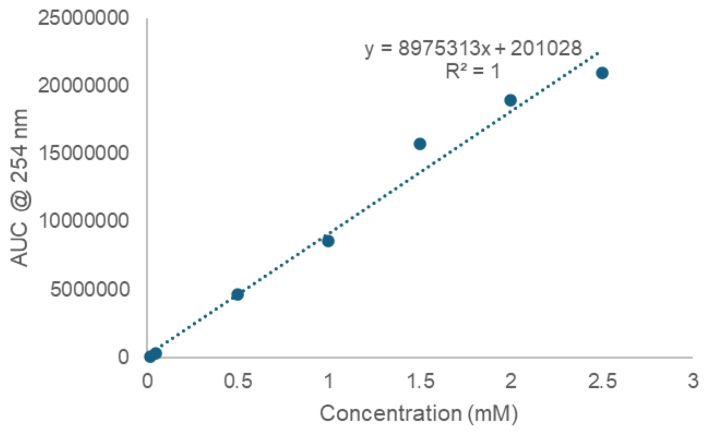

b

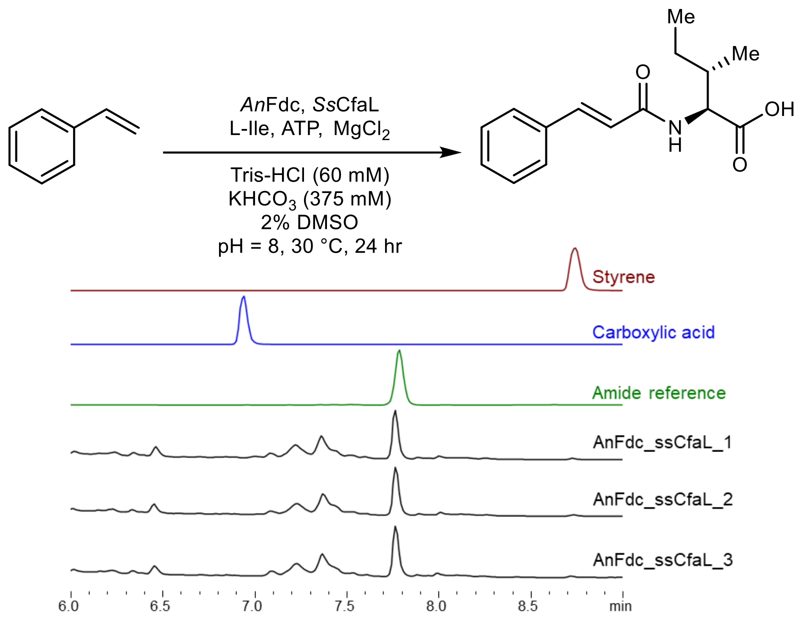

Figure S57. Enzymatic C-H amidation cascade for the synthesis of 49. a, HPLC calibration using amide synthetic standard. **b,** Reaction scheme and HPLC chromatogram (HPLC method C). Traces recorded at 254 nm. Prepared according to general method 4 using styrene and L-isoleucine employing AnFdc and SsCfaL. HPLC yield: run1 = 2%, run2 = 2%, run3 = 2%. Average = 2 ± 0.09% (triplicate).

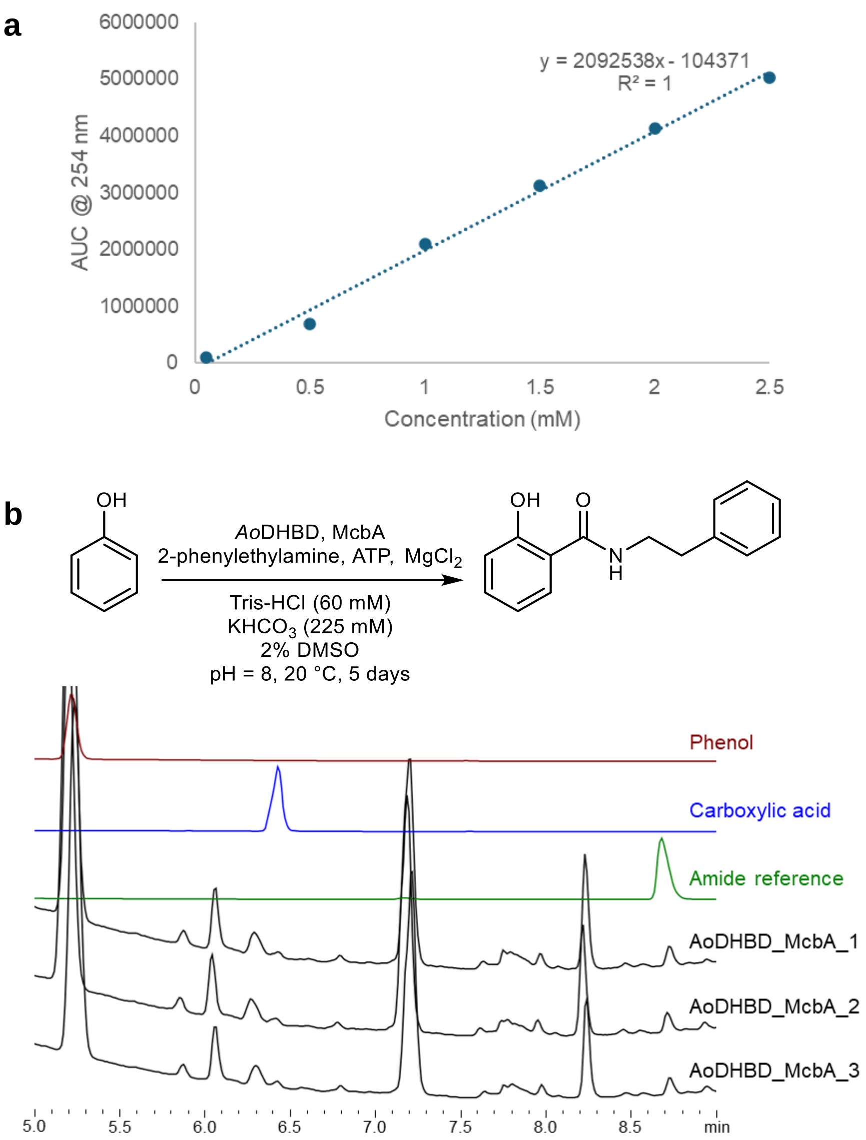

Figure S58. Enzymatic C-H amidation cascade reaction for the synthesis of 50. a, HPLC calibration using amide synthetic standard. **b,** Reaction scheme and HPLC chromatogram (HPLC method C). recorded at 254 nm. Prepared according to general method 4 using phenol and 2-phenylethylamine employing AoDHBD and McbA. HPLC yield: run1 = < 1%, run2 = < 1%, run3 = < 1%. Average = < 1% (triplicate).

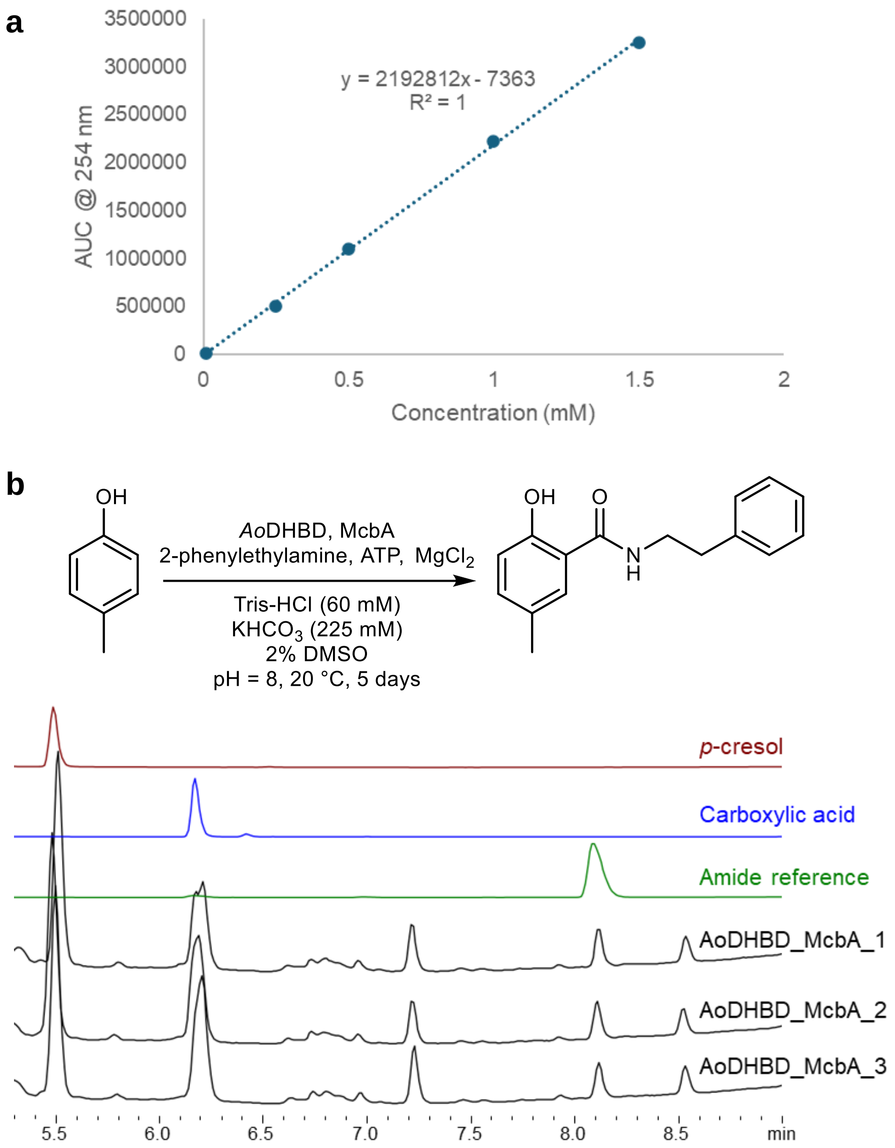

Figure S59. Enzymatic C-H amidation cascade for the synthesis of 51. a, HPLC calibration using amide synthetic standard. **b,** Reaction scheme and HPLC chromatogram (HPLC method C). Traces recorded at 254 nm. Prepared according to general method 4 using *p*-cresol and 2-phenylethylamine employing AoDHBD and McbA. HPLC yield: run1 = 1%, run2 = 1%, run3 = 1%. Average = 1 ± 0.06% (triplicate).

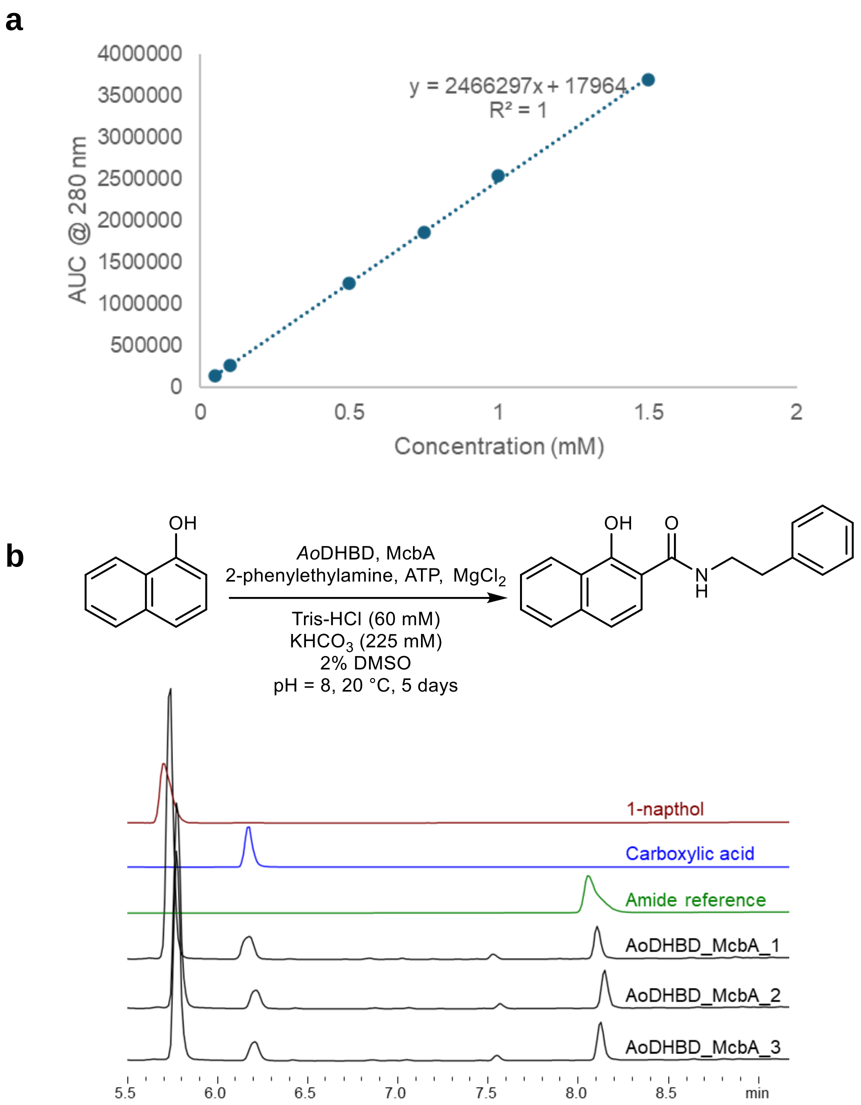

Figure S60. Enzymatic C-H amidation reaction for the synthesis of 52. a, HPLC calibration using amide synthetic standard. **b,** Reaction scheme and HPLC chromatogram (HPLC method A). Traces recorded at 280 nm. Prepared according to general method 4 using 1-napthol and 2-phenylethylamine employing AoDHBD and McbA. HPLC yield: run1 = 7%, run2 = 8%, run3 = 8%. Average = 8 ± 0.4% (triplicate).

NMR spectra of compounds synthesised enzymatically.

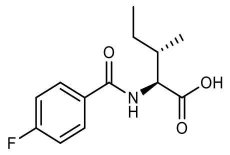

^{1}H NMR spectrum of **7** (synthesised using AfNIT - SsCfaL cascade)

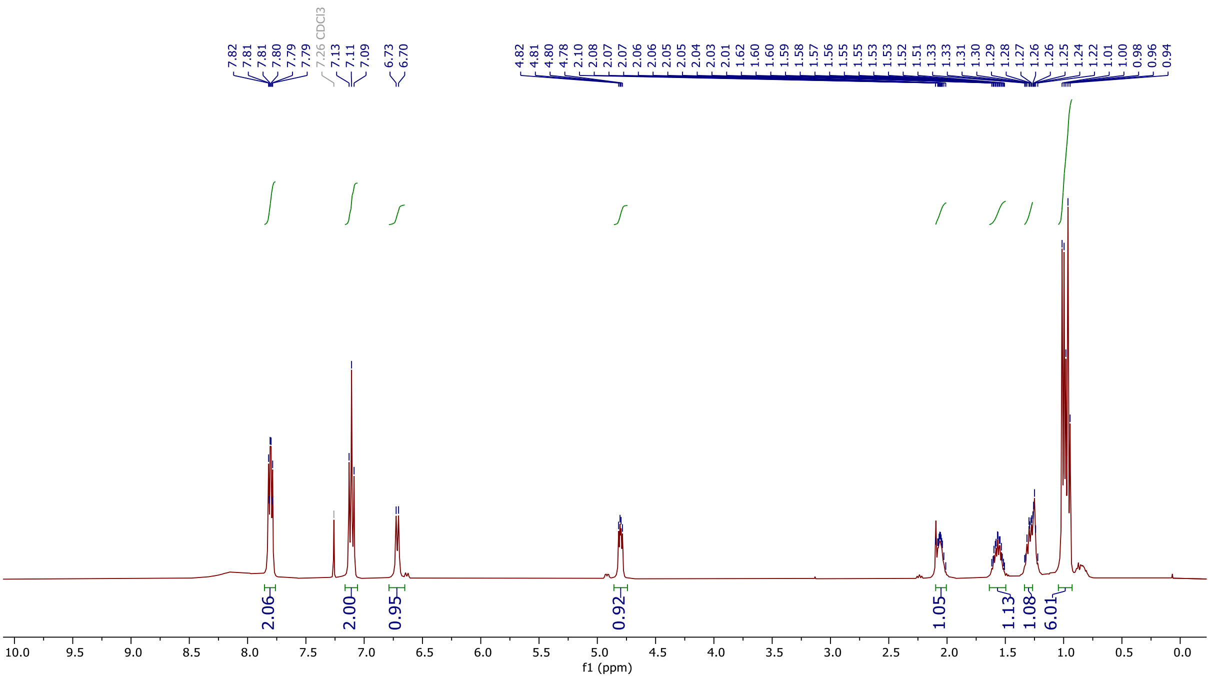

^{13}C NMR spectrum of **7** (synthesised using AfNIT - SsCfaL cascade)

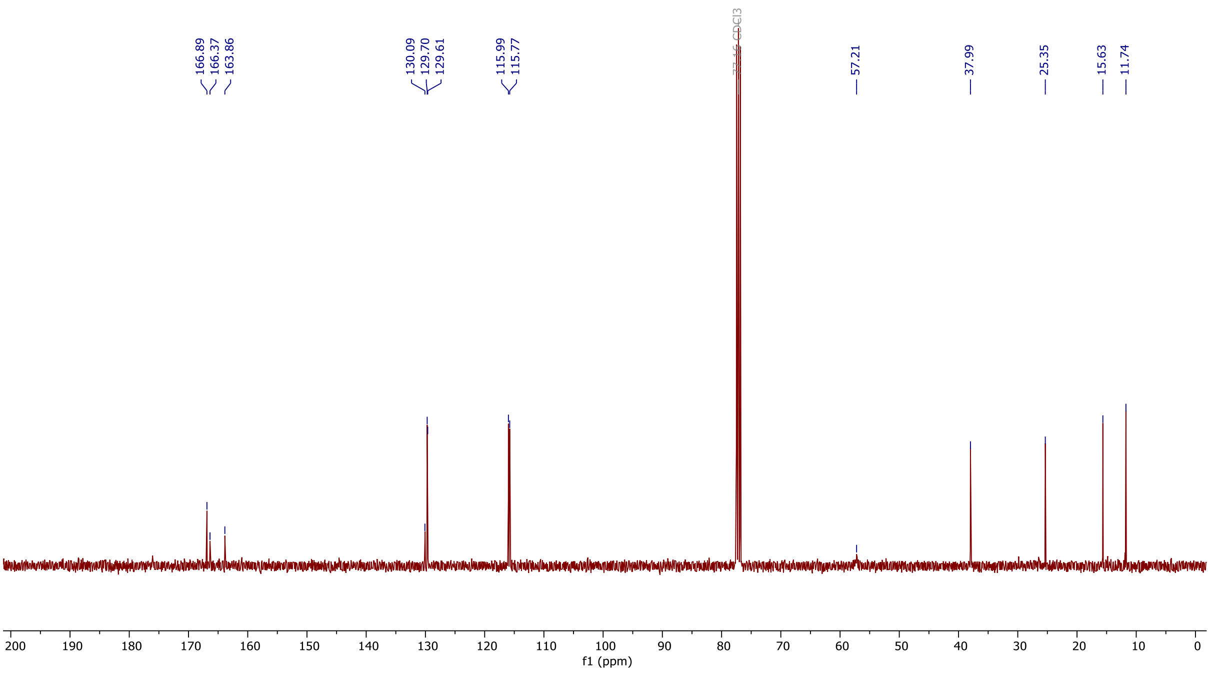

^{19}F NMR spectrum of **7** (synthesised using AfNIT - SsCfaL cascade)

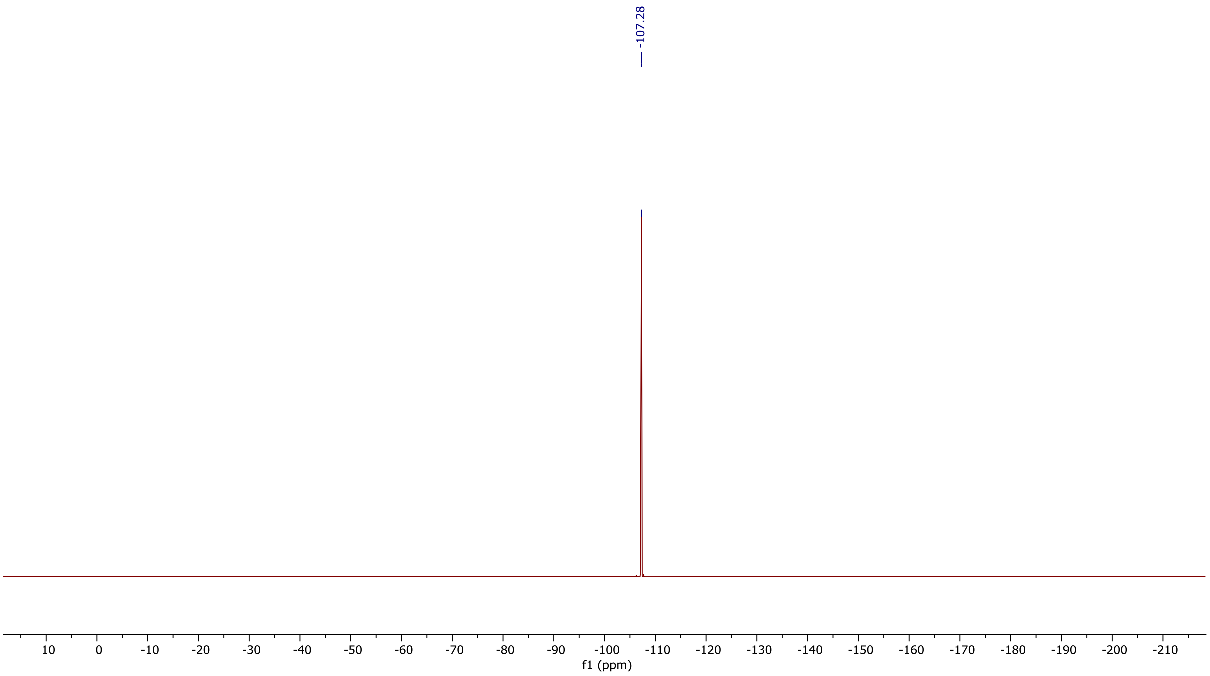

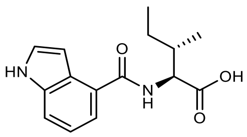

^{1}H NMR spectrum of **20** (synthesised using RrNIT - SsCfaL cascade)

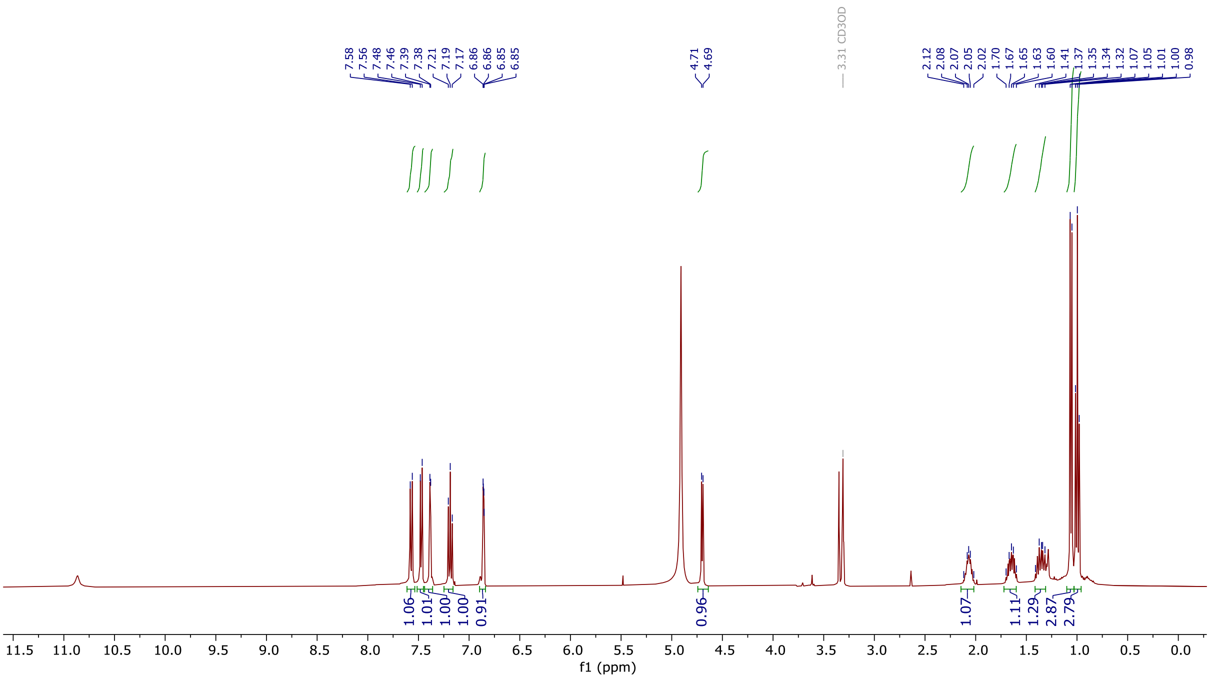

^{13}C NMR spectrum of **20** (synthesised using RrNIT - SsCfaL cascade)

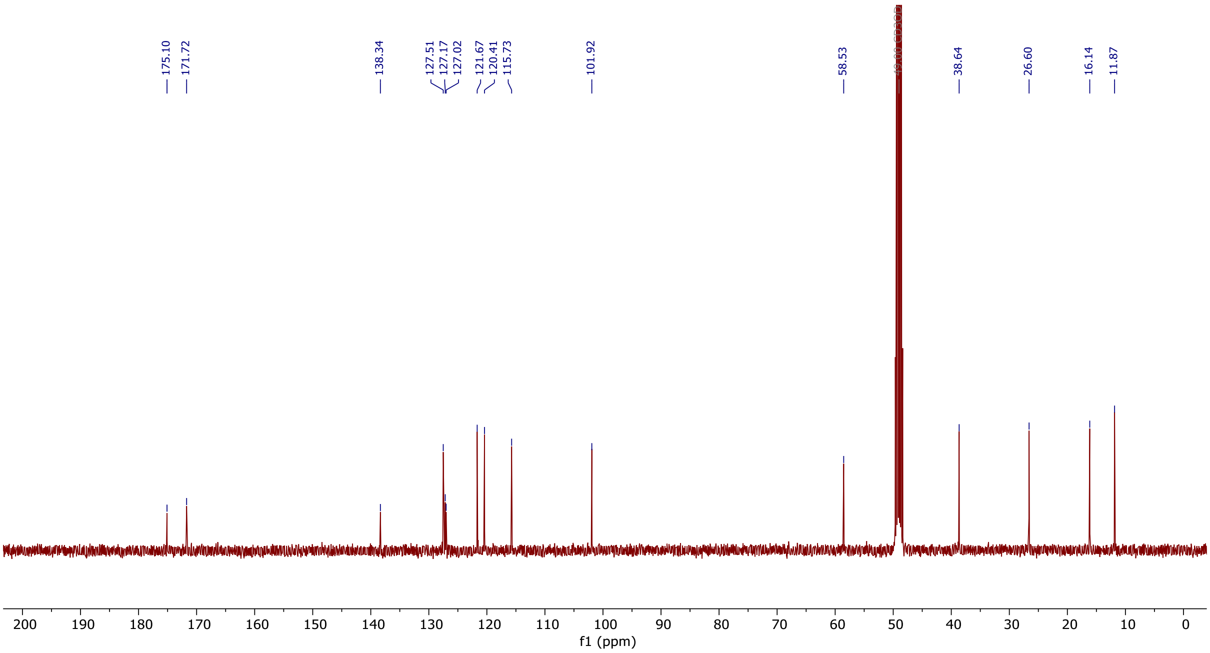

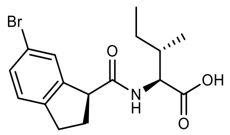

^{1}H NMR spectrum of **31** (synthesised using RrNIT - AlCfaL cascade)

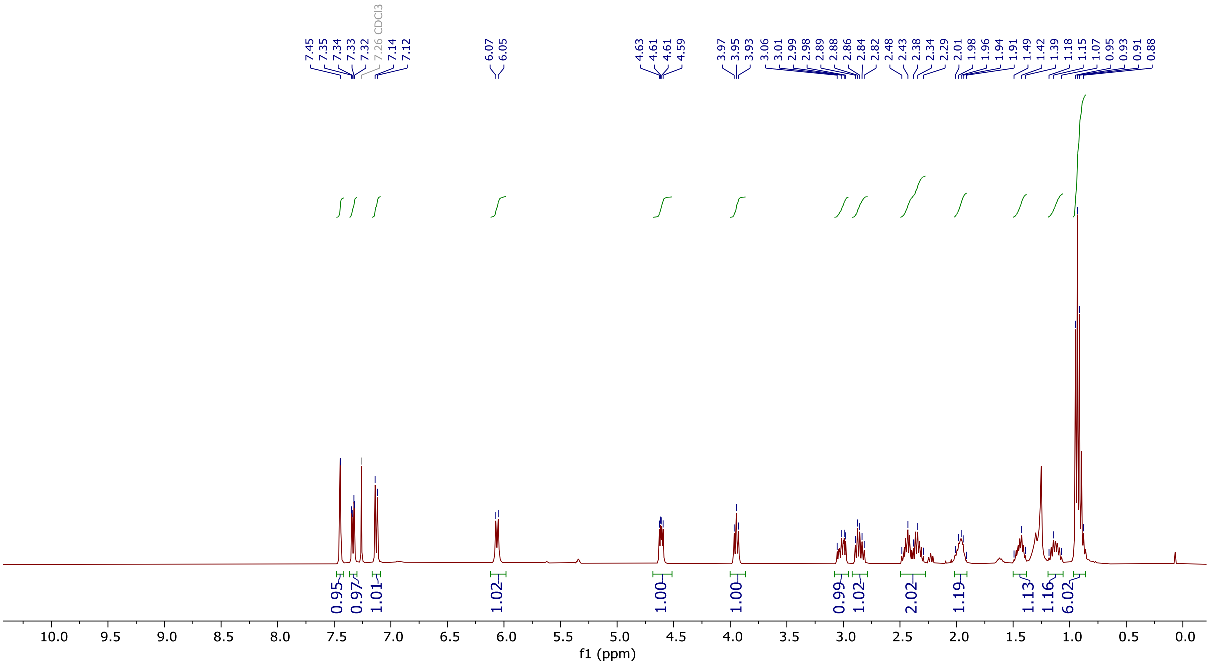

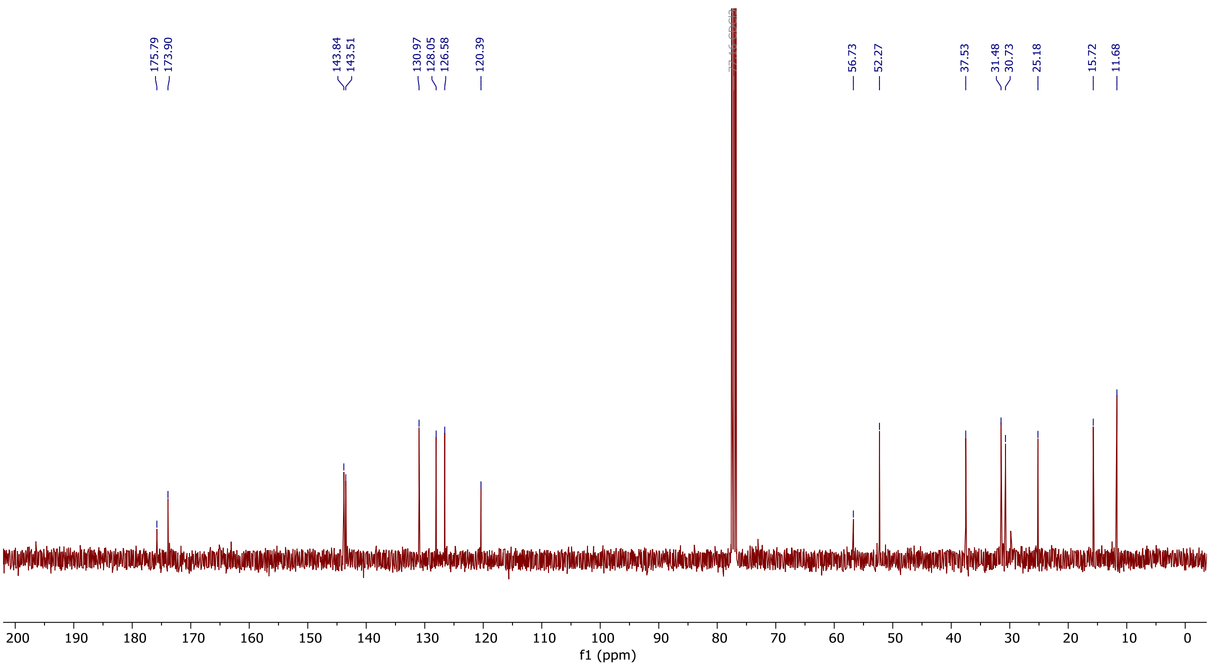

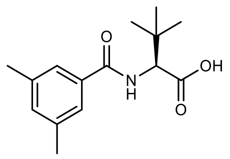

^{1}H NMR spectrum of **35** (synthesised using FdNIT - SsCfaL cascade)

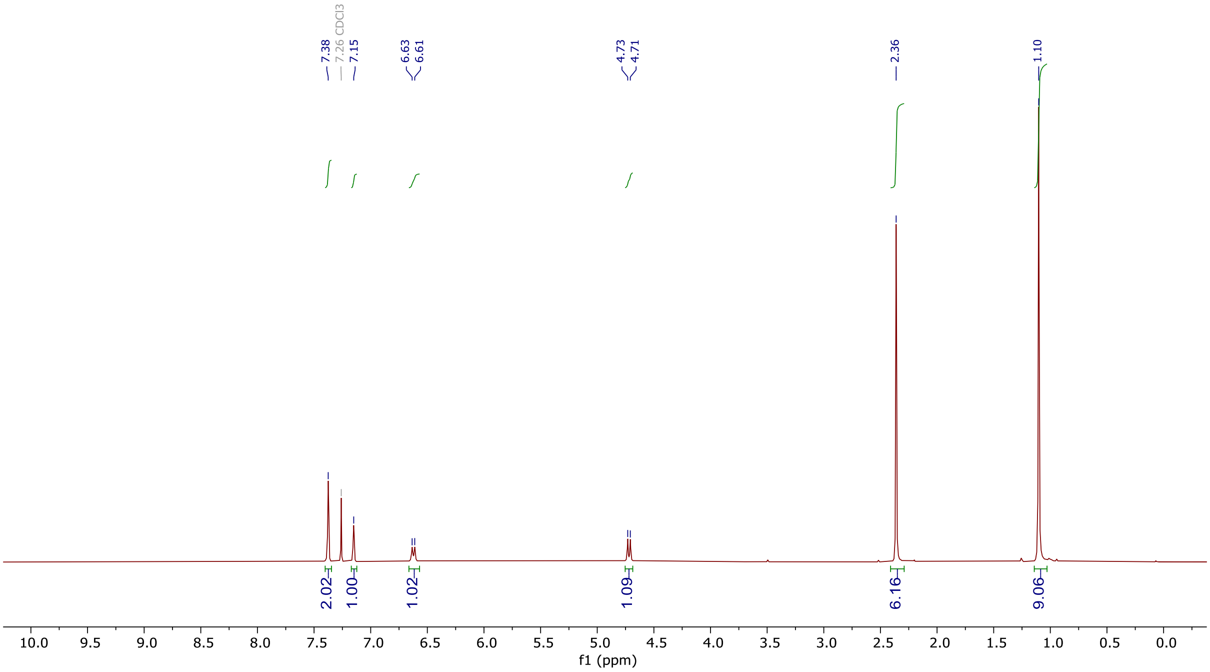

^{13}C NMR spectrum of **35** (synthesised using FdNIT - SsCfaL cascade)

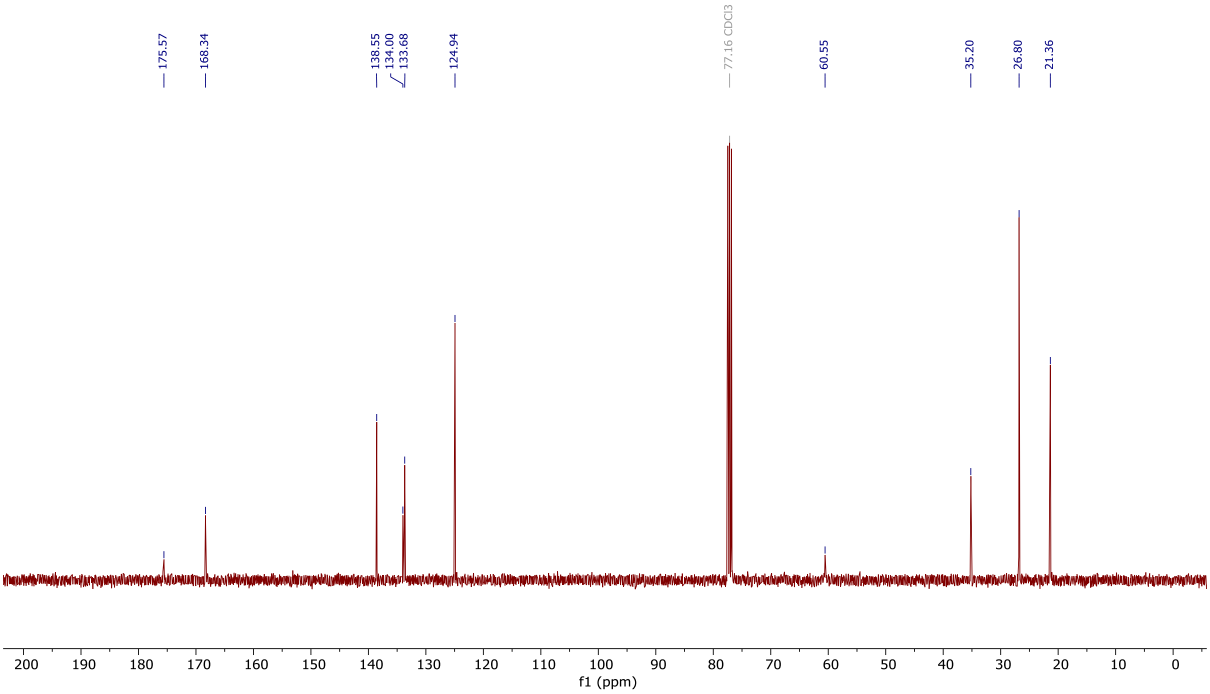

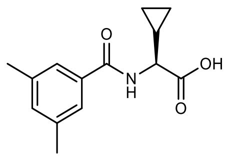

^{1}H NMR spectrum of **38** (synthesised using FdNIT - AlCfaL cascade)

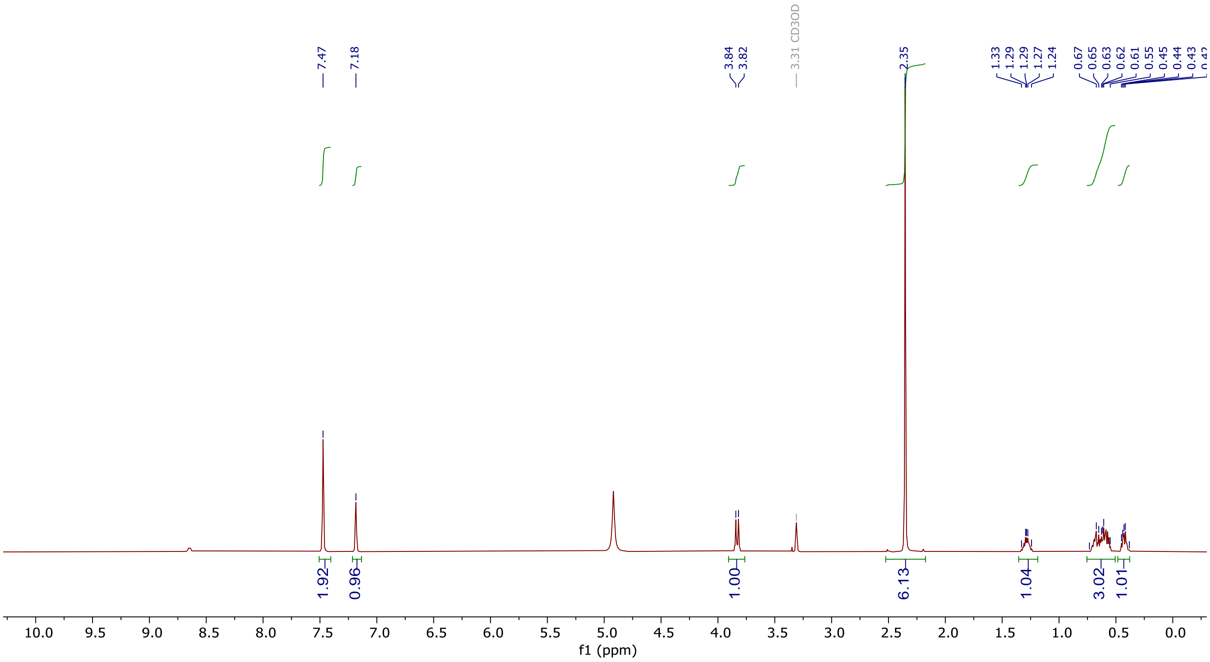

^{13}C NMR spectrum of **38** (synthesised using FdNIT - AlCfaL cascade)

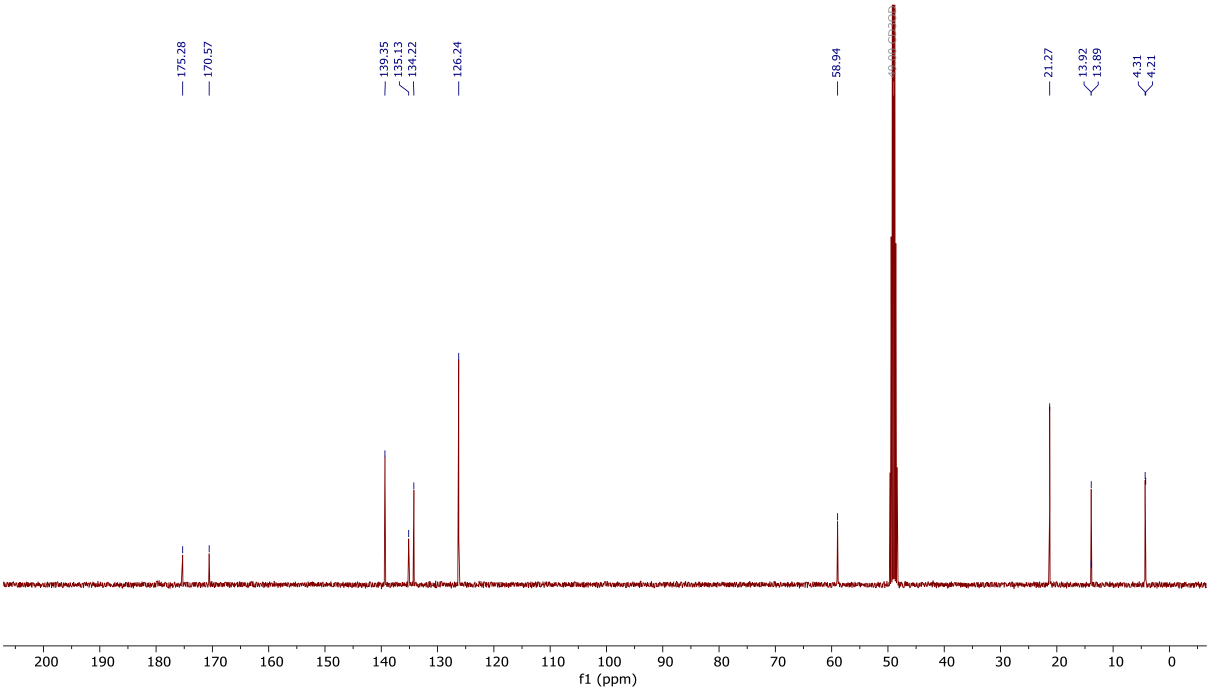

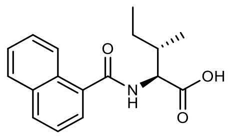

^{1}H NMR spectrum of **13** (synthesised using RrNIT - PbCfaL cascade)

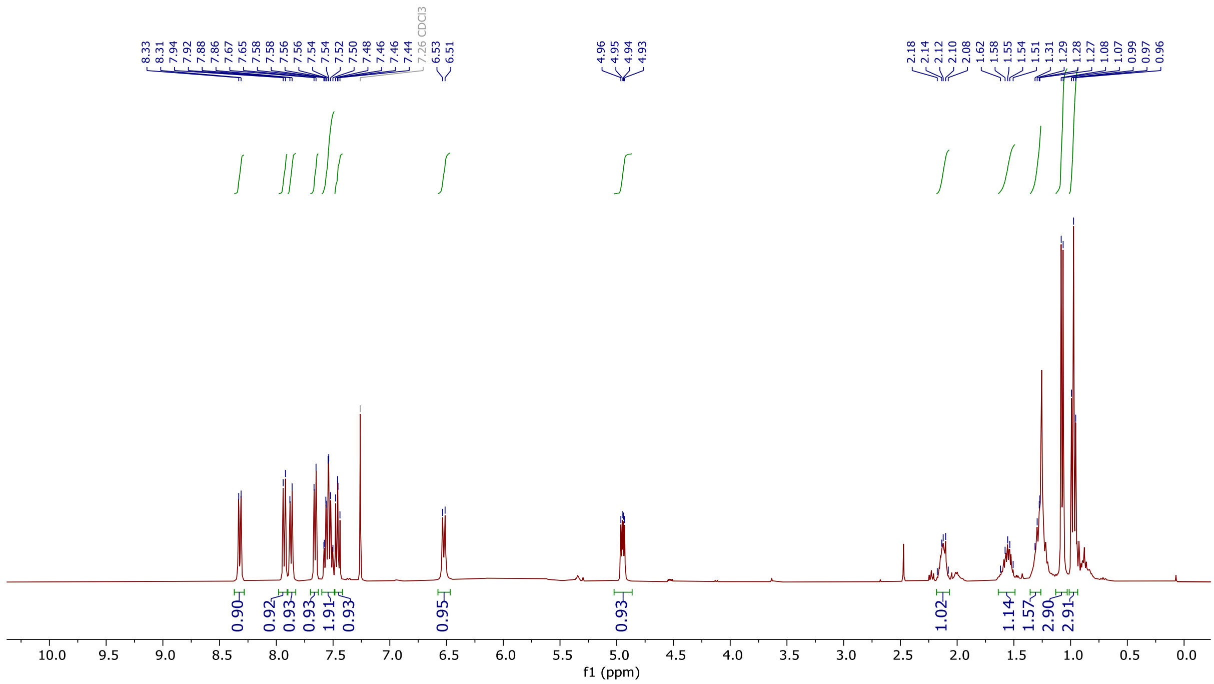

^{13}C NMR spectrum of **13** (synthesised using RrNIT - PbCfaL cascade)

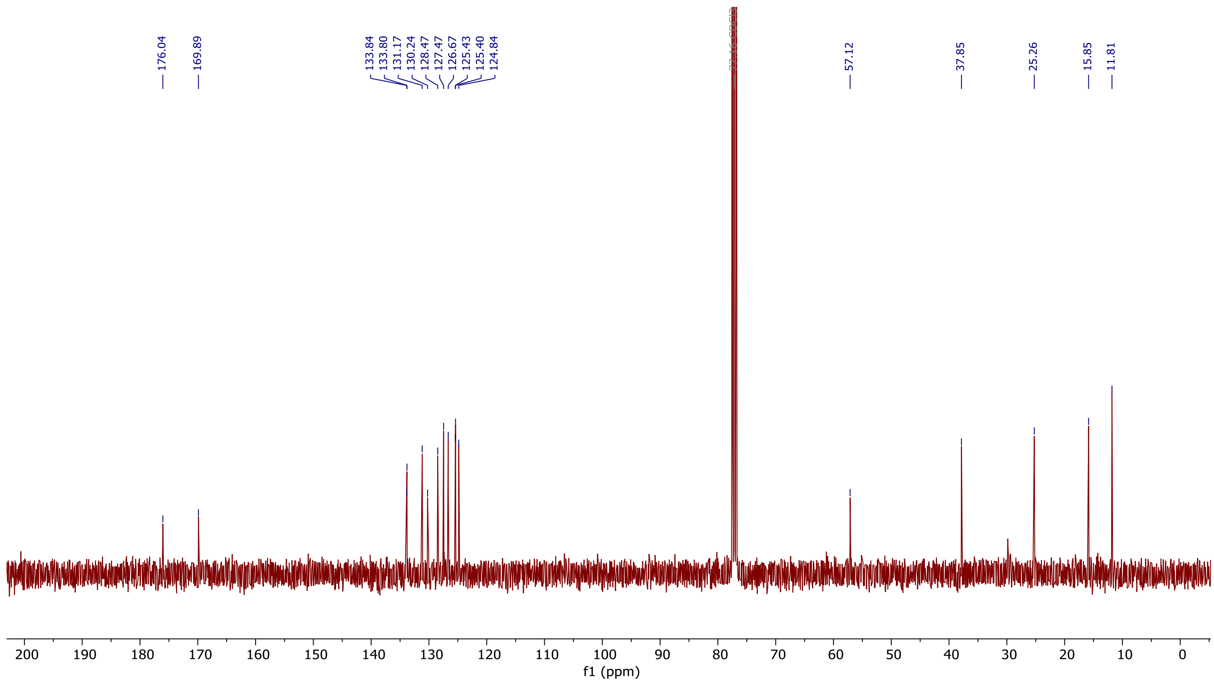

NMR spectra of chemically synthesised standards and starting material.

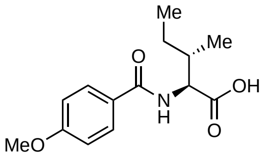

^{1}H NMR spectrum of **1**.

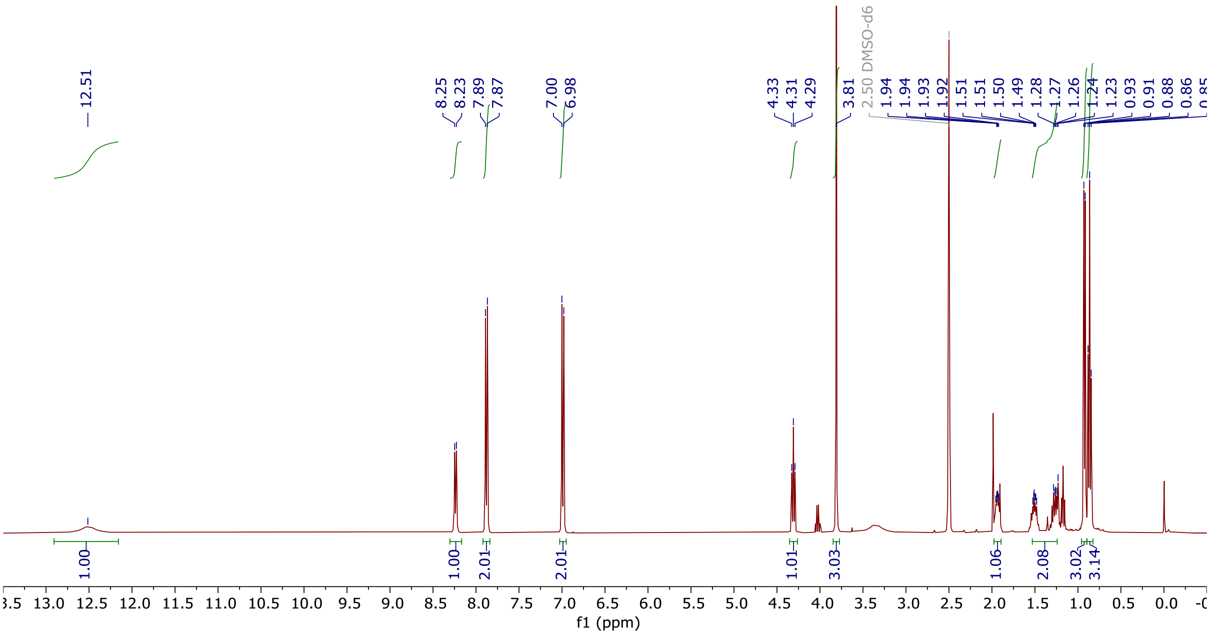

^{13}C NMR spectrum of **1**.

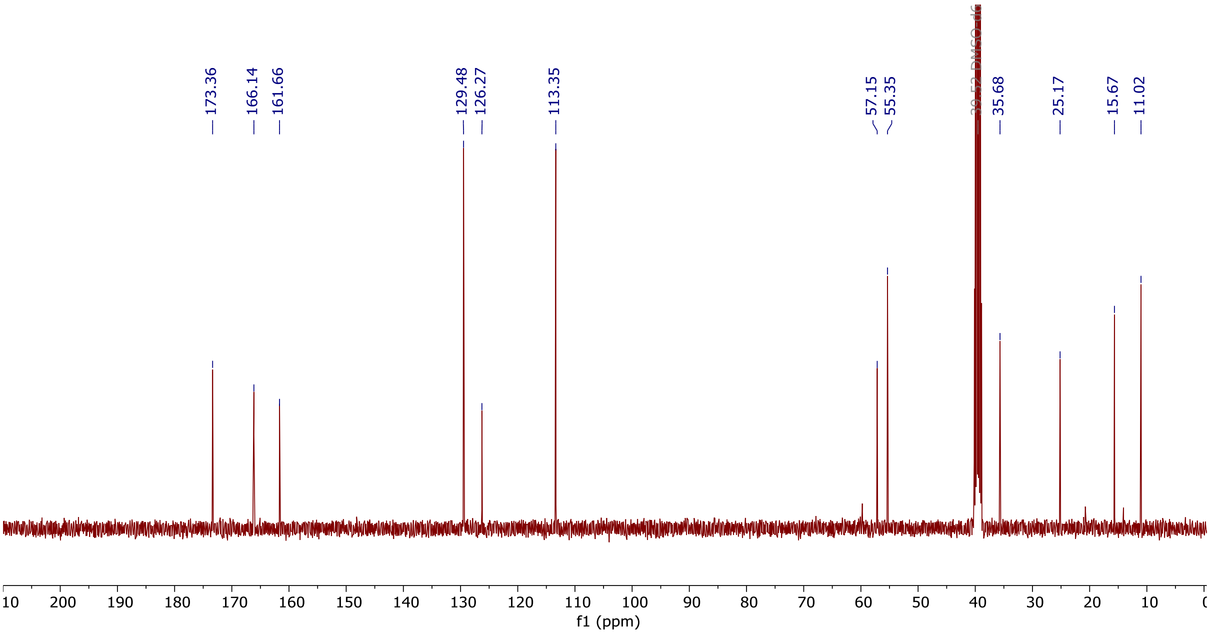

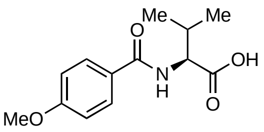

^{1}H NMR spectrum of **2**.

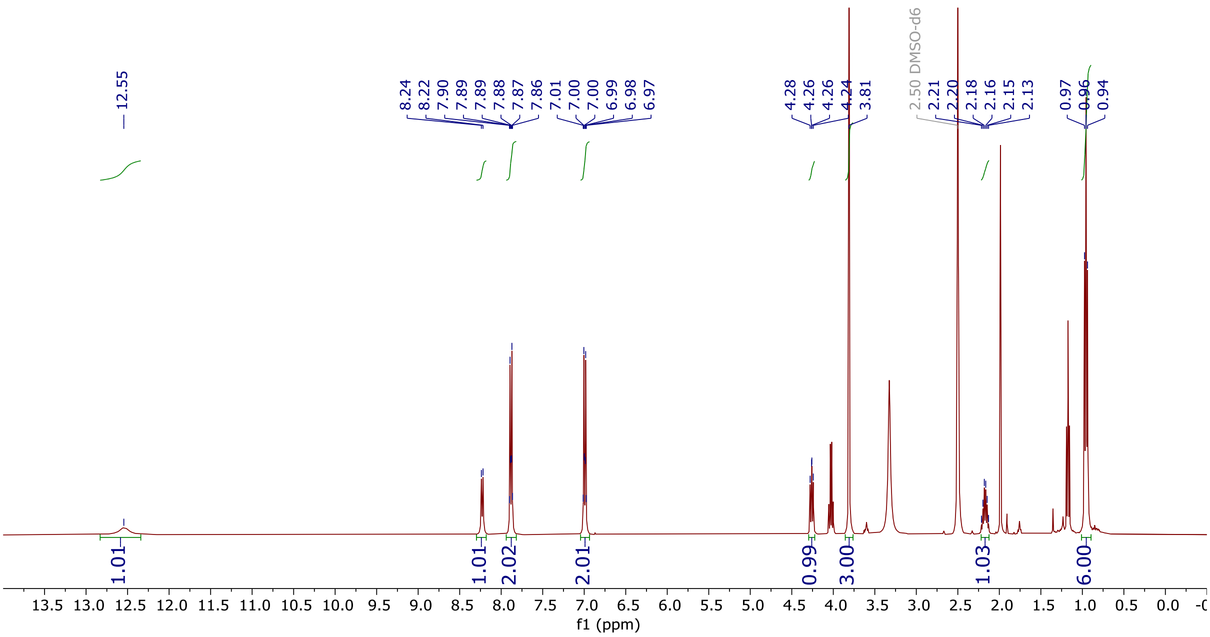

^{13}C NMR spectrum of **2**.

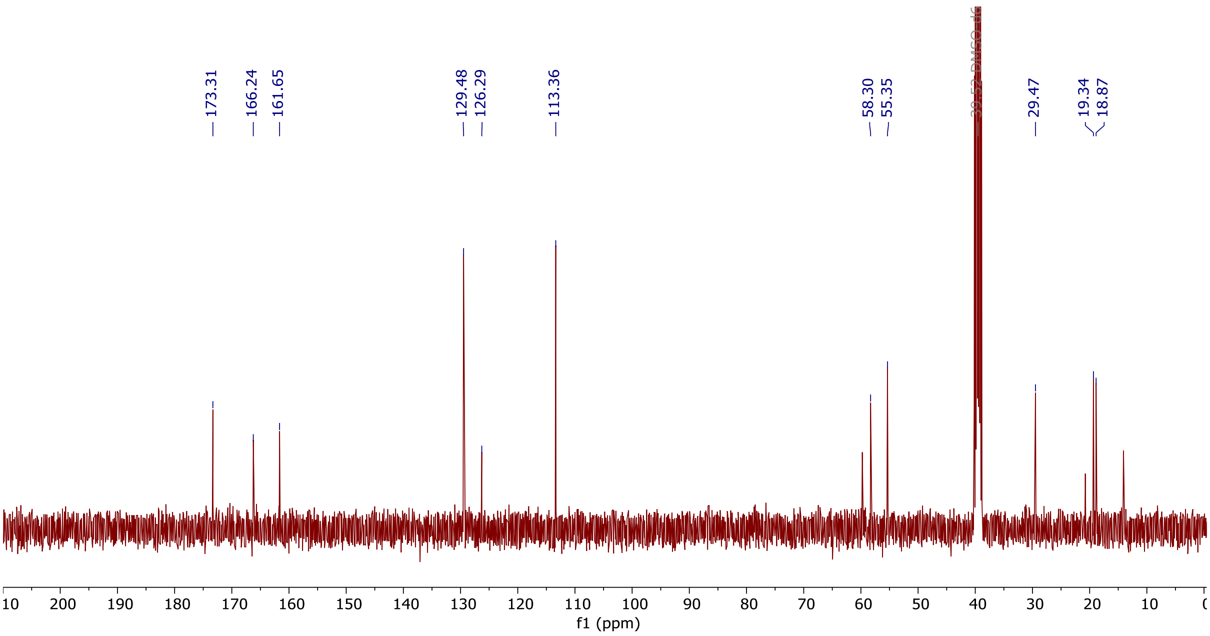

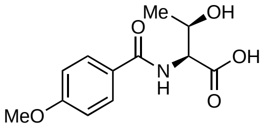

^{1}H NMR spectrum of **3**.

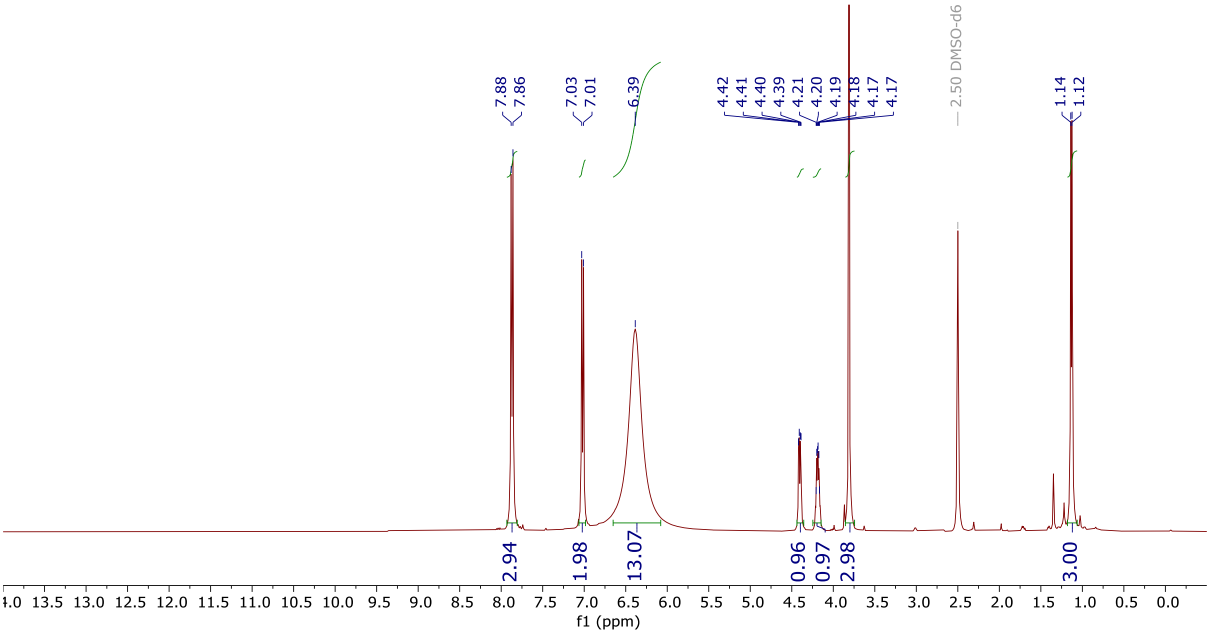

^{13}C NMR spectrum of **3**.

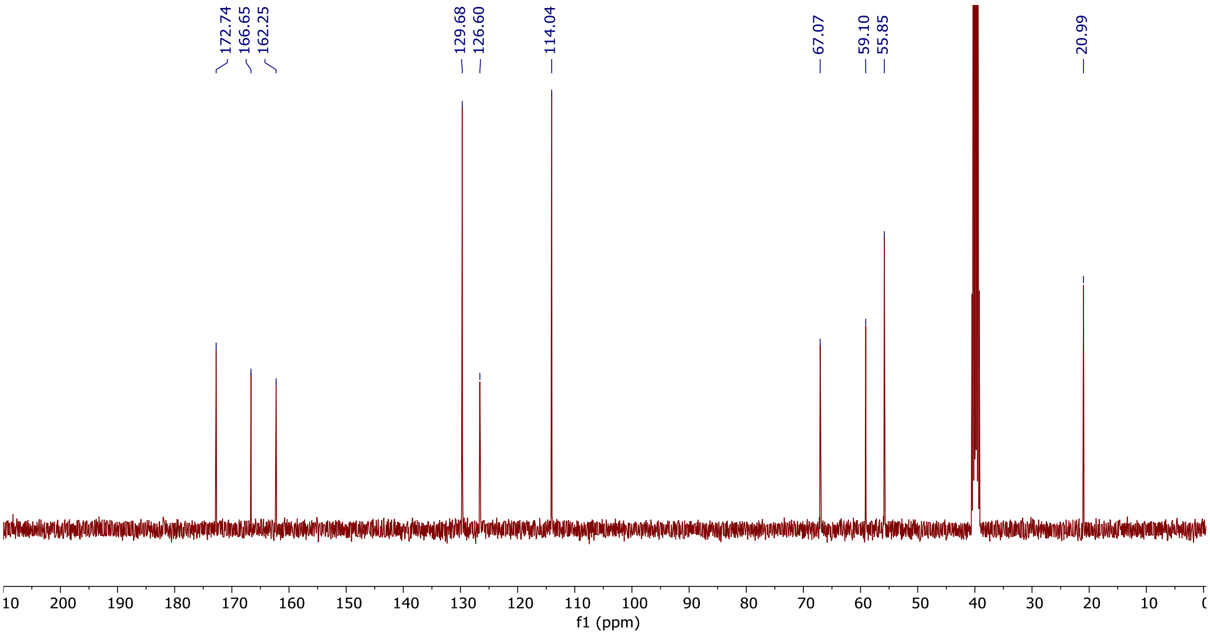

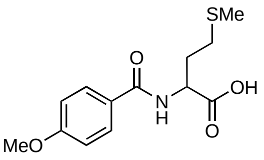

^{1}H NMR spectrum of **4**.

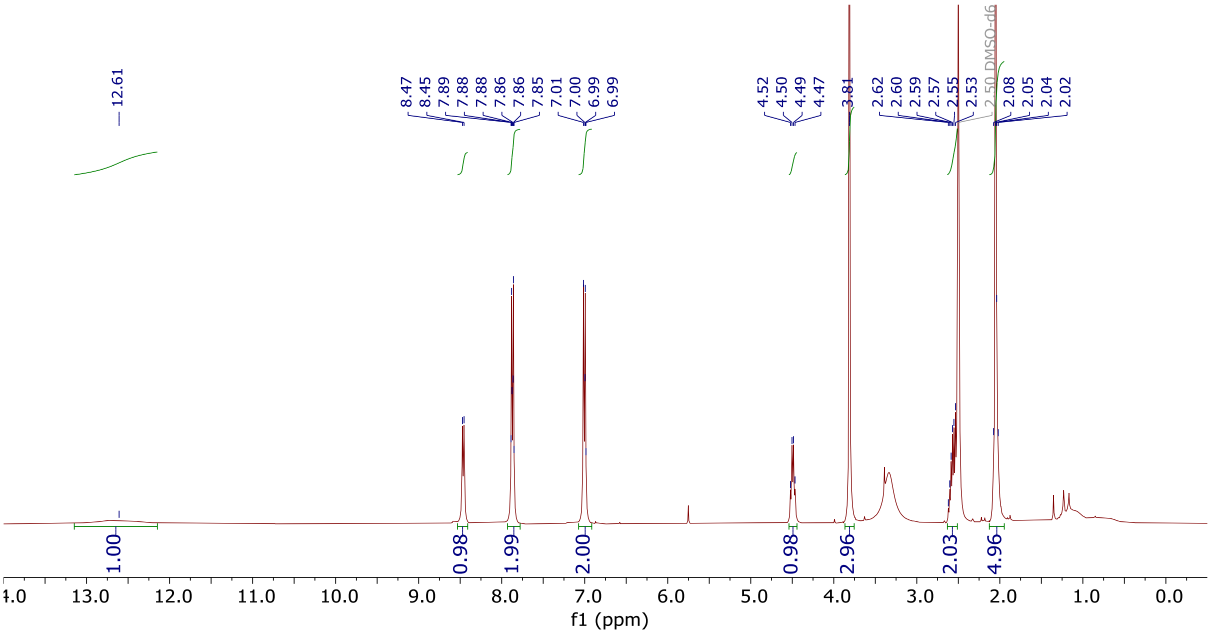

^{13}C NMR spectrum of **4**.

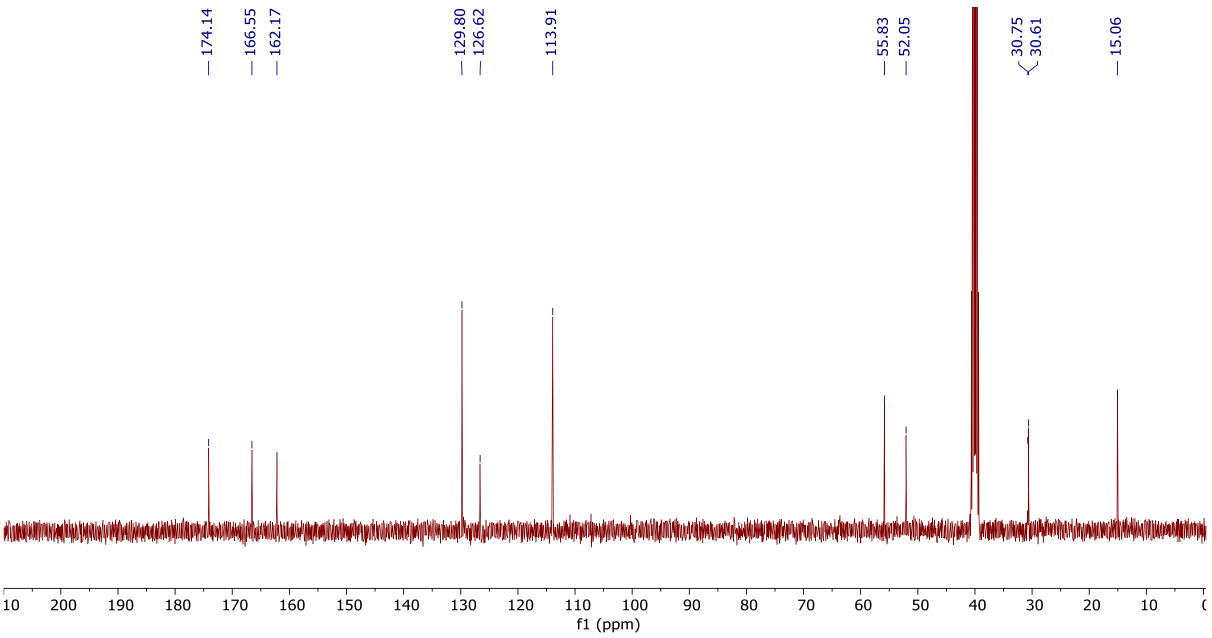

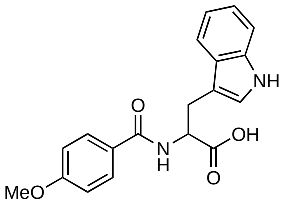

^{1}H NMR spectrum of **5**

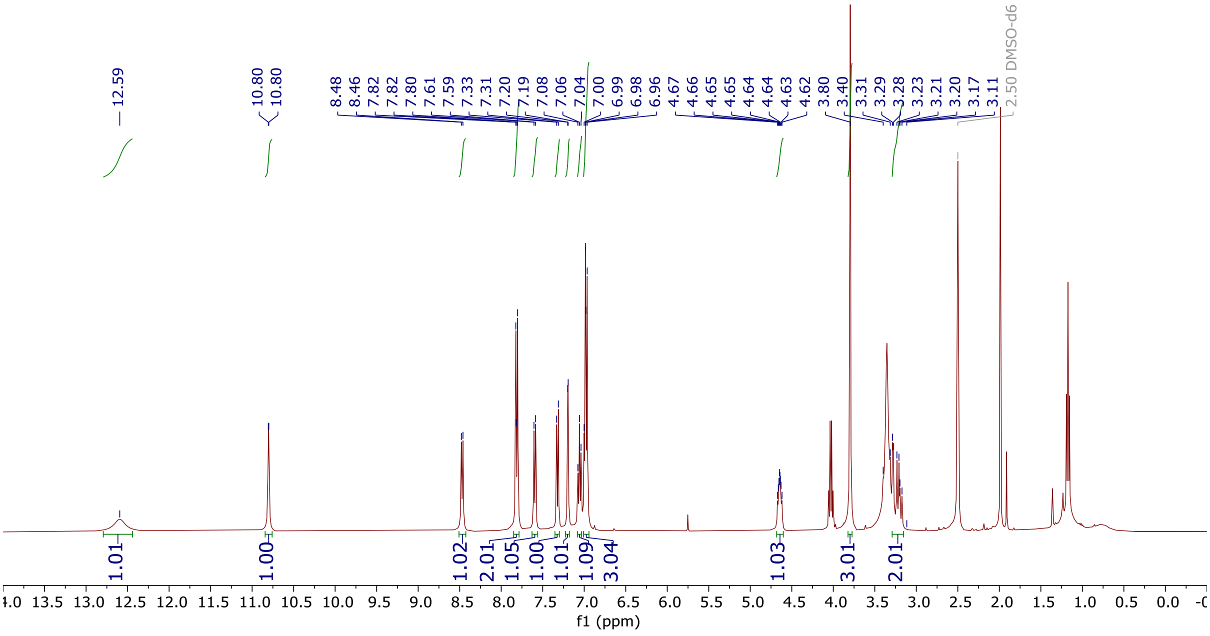

^{13}C NMR spectrum of **5**

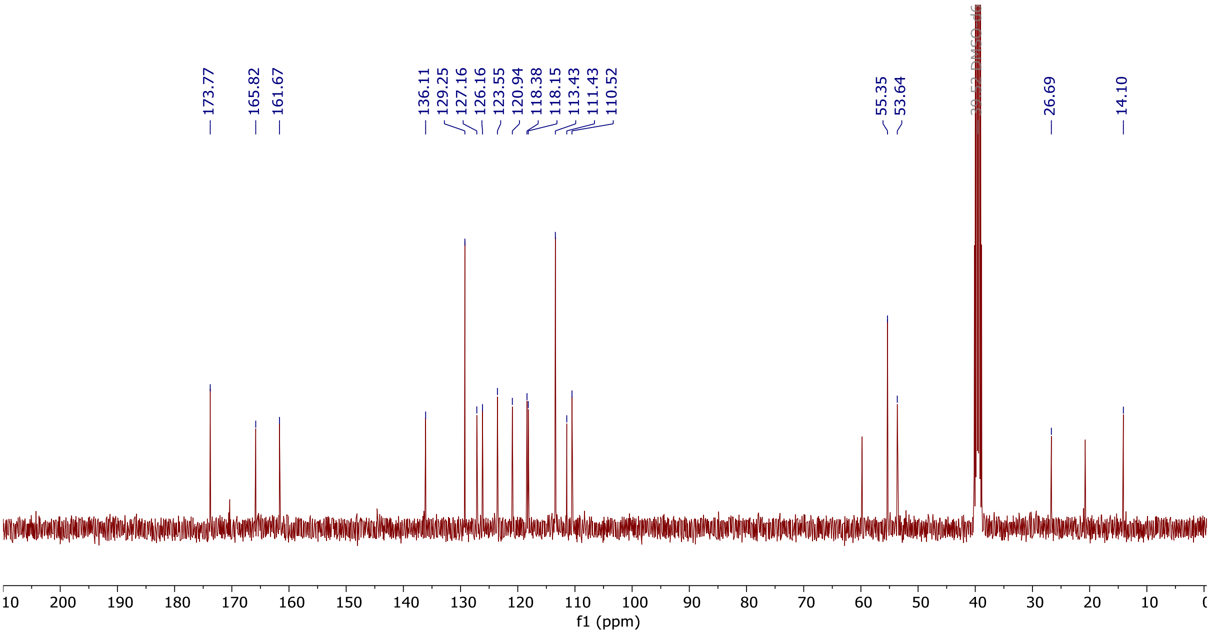

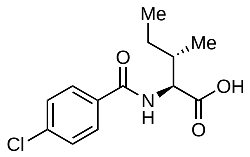

^{1}H NMR spectrum of **6**.

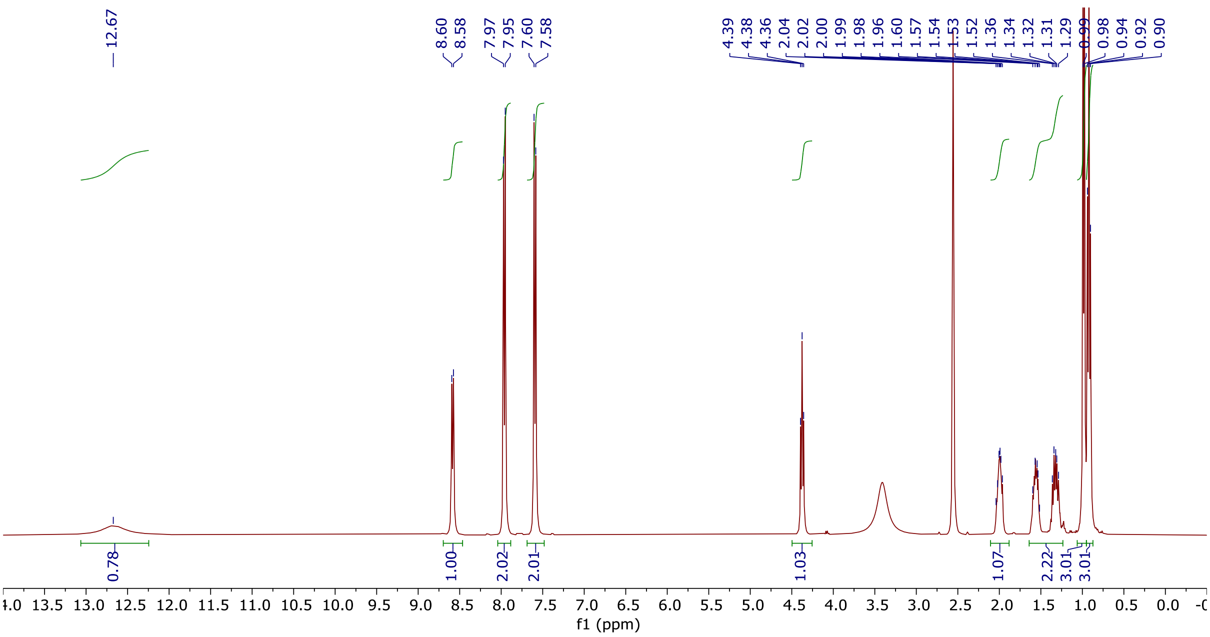

^{13}C NMR spectrum of **6**.

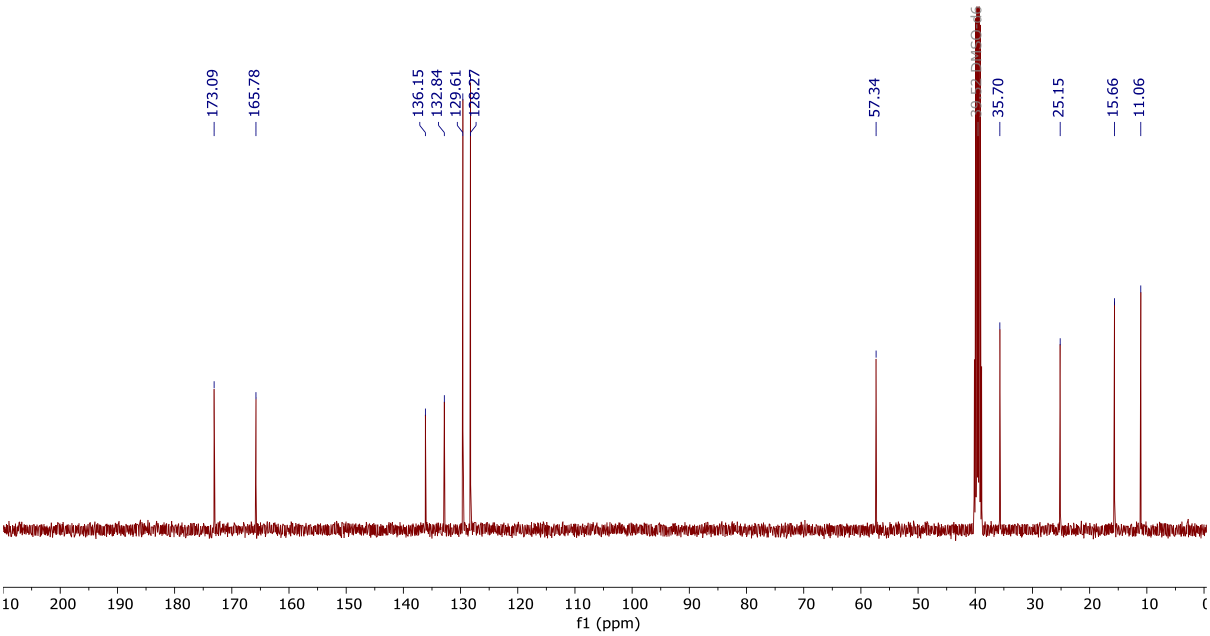

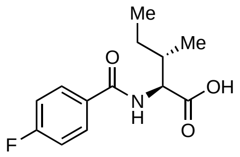

^{1}H NMR spectrum of **7**.

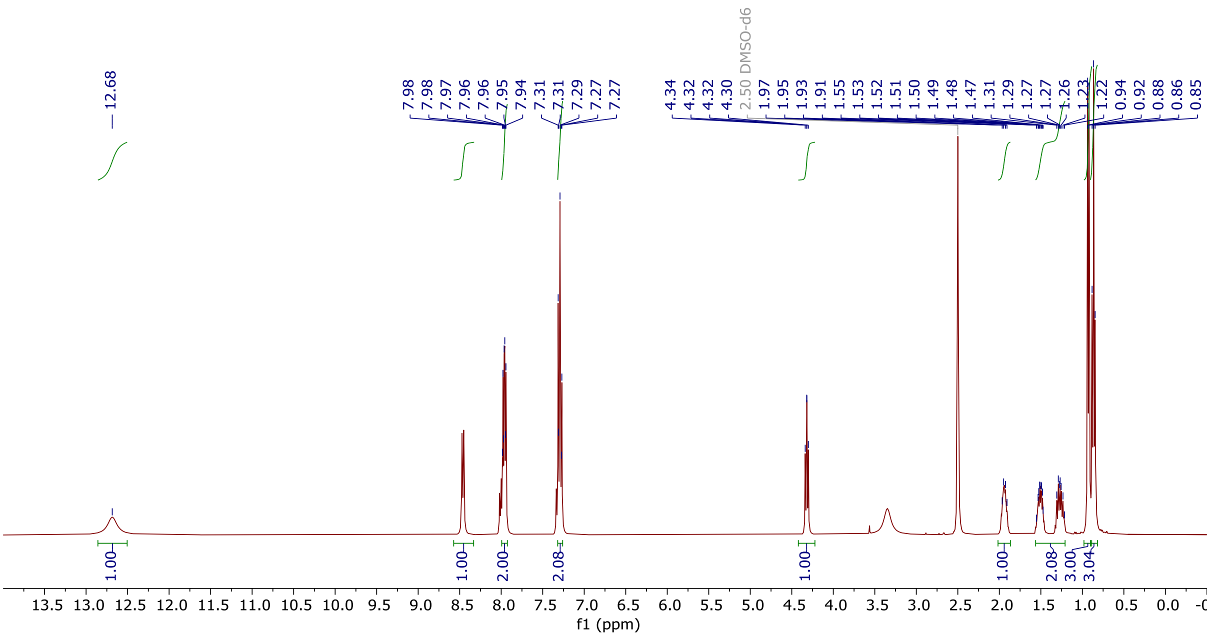

^{13}C NMR spectrum of **7**.

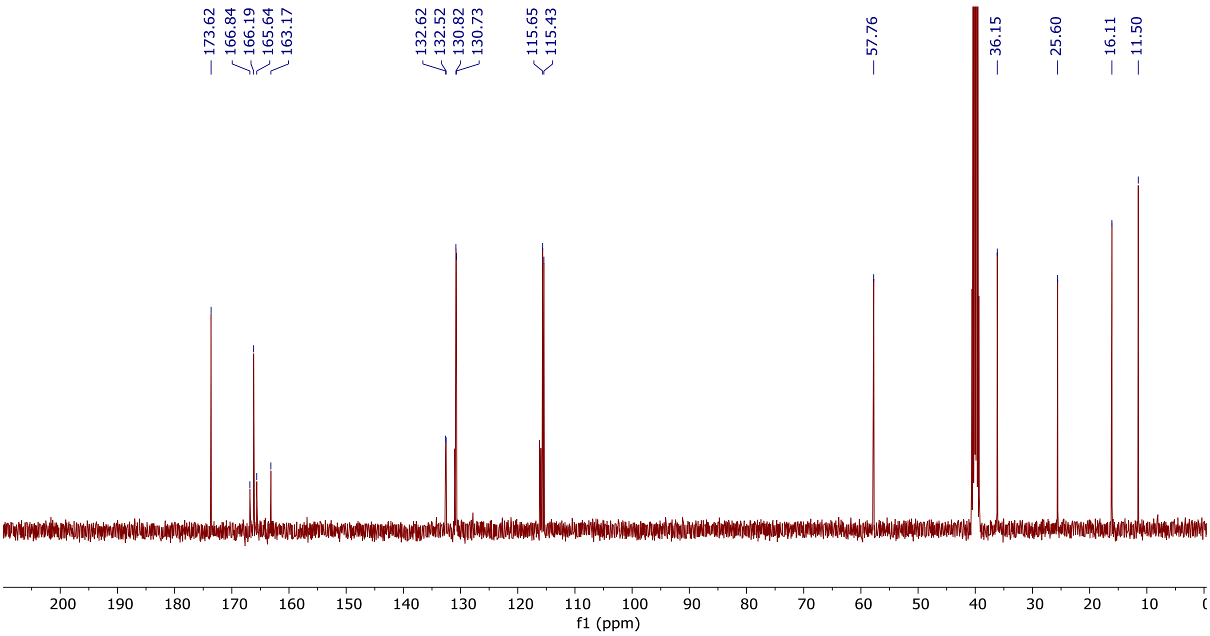

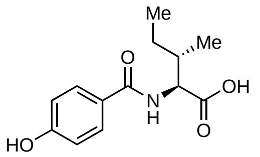

^{1}H NMR spectrum of **8**.

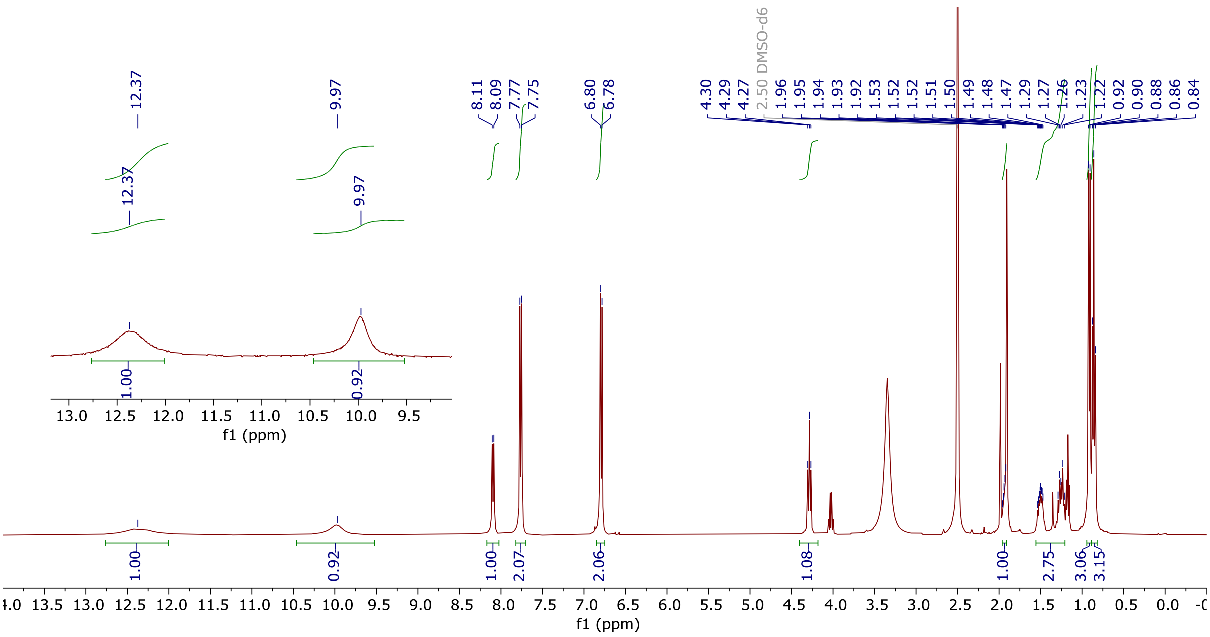

^{13}C NMR spectrum of **8**.

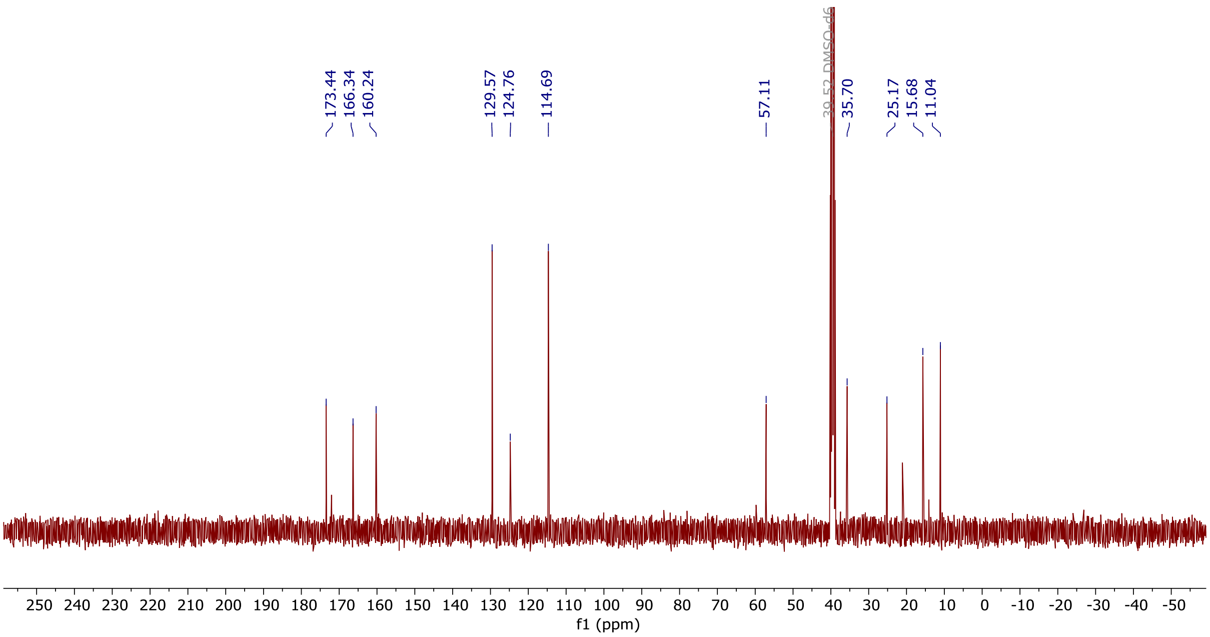

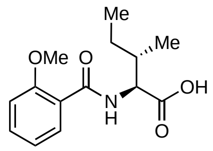

^{1}H NMR spectrum of **9**.

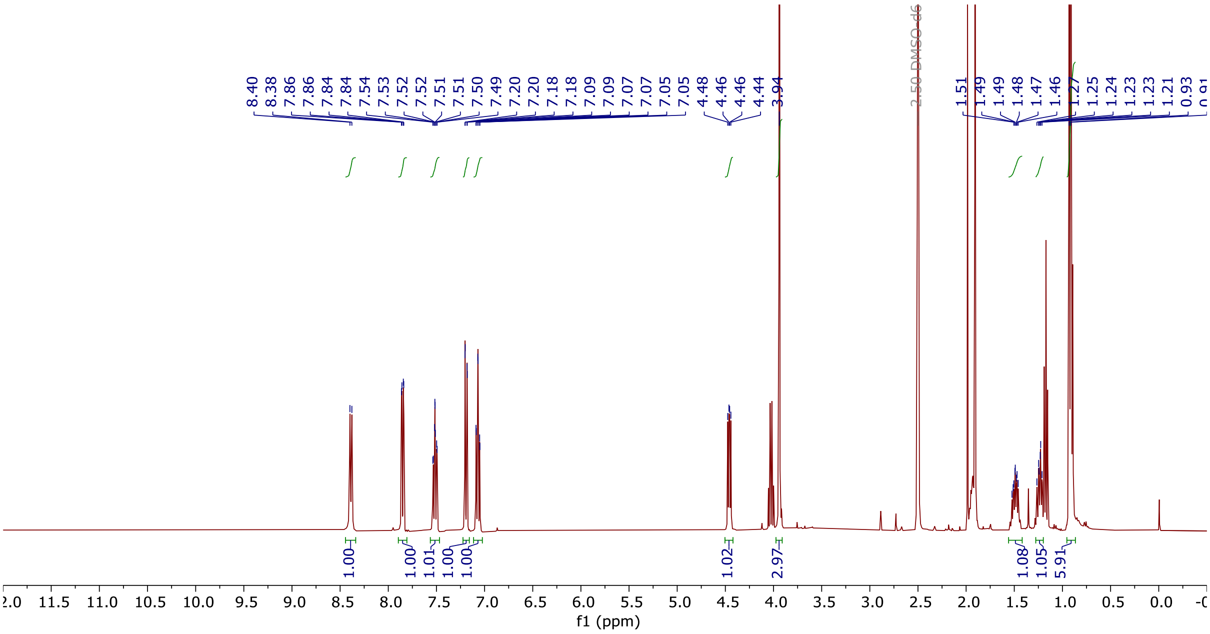

^{13}C NMR spectrum of **9**.

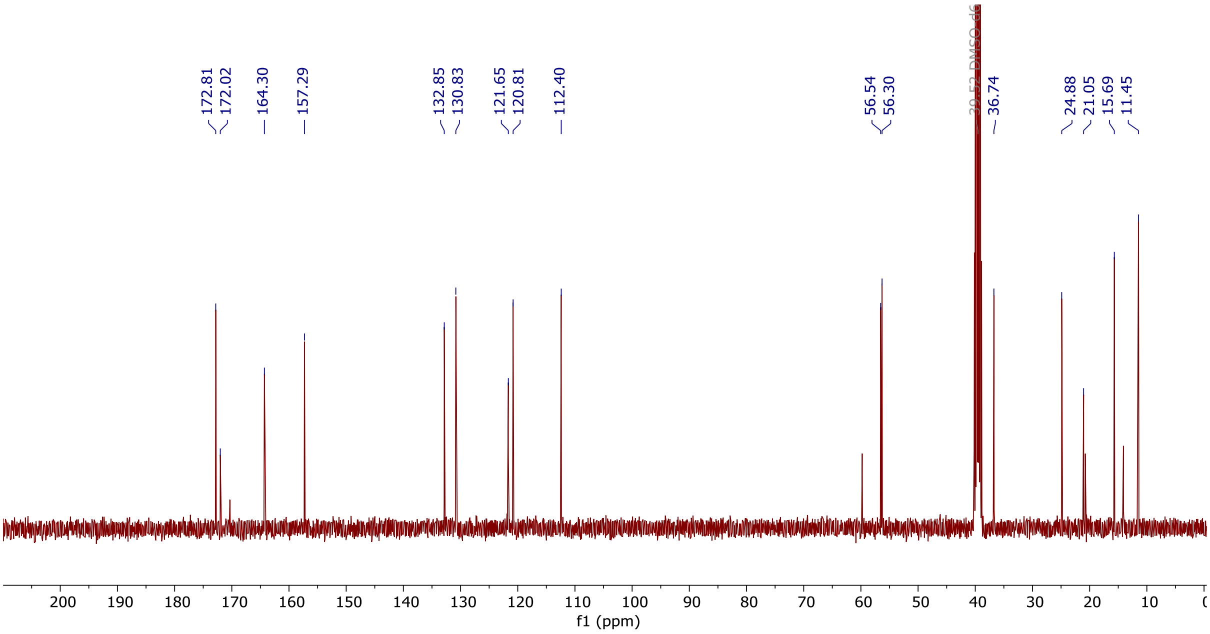

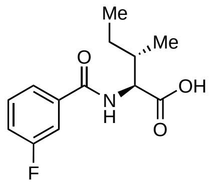

^{1}H NMR spectrum of **10**.

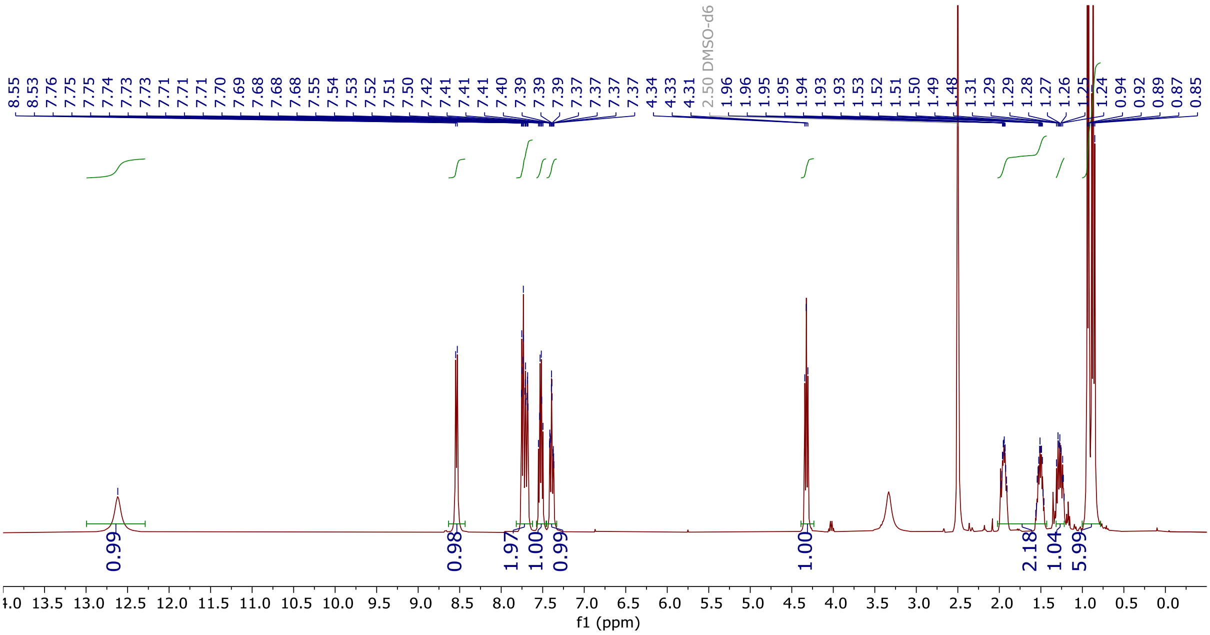

^{13}C NMR spectrum of **10**.

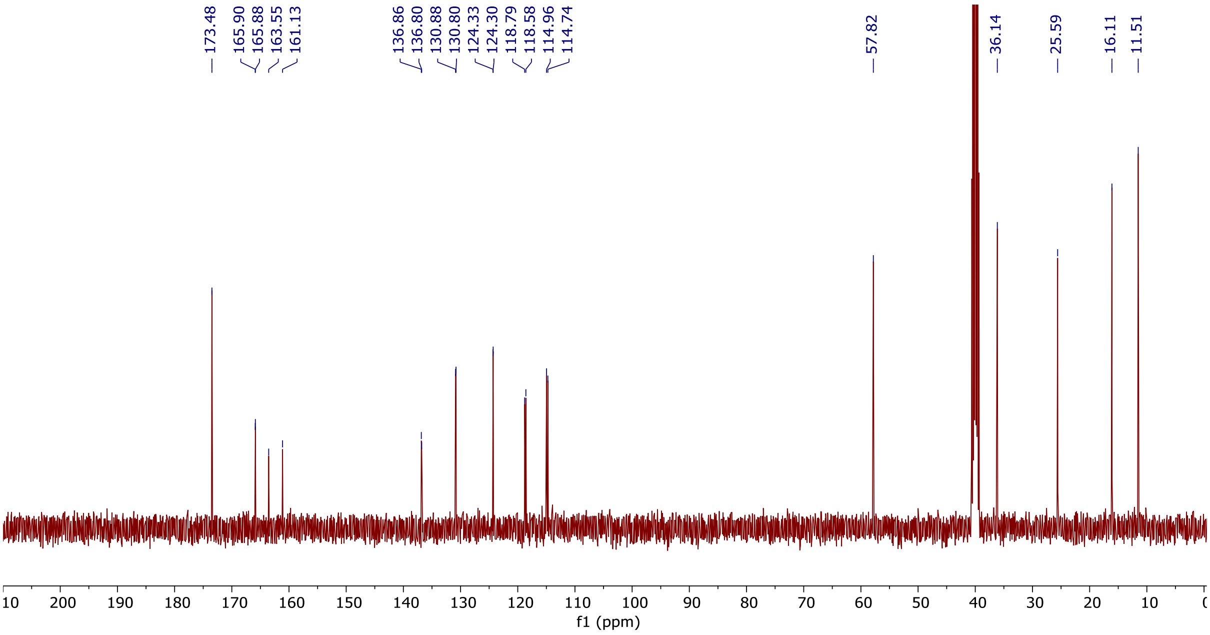

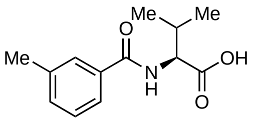

^{1}H NMR spectrum of **11**.

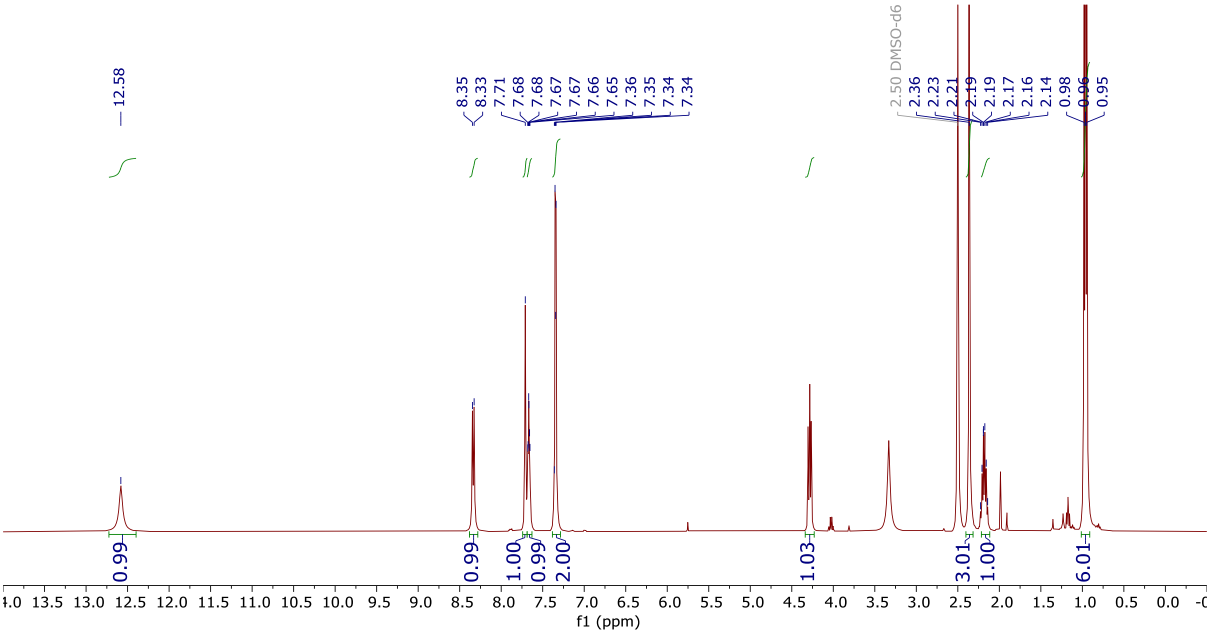

^{13}C NMR spectrum of **11**.

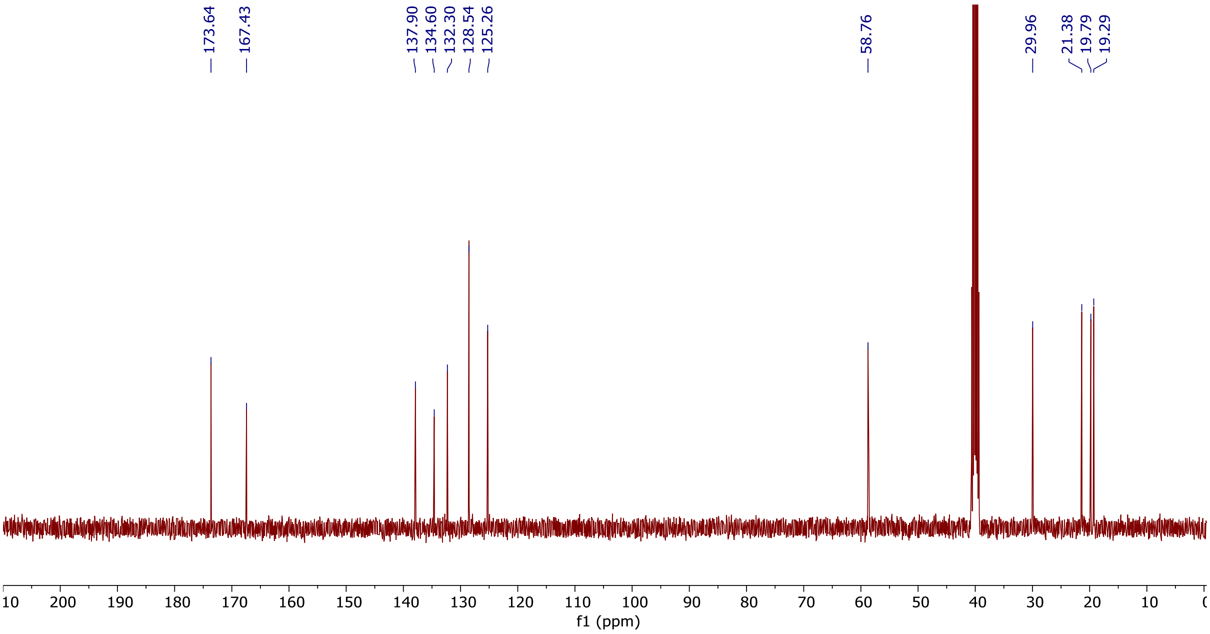

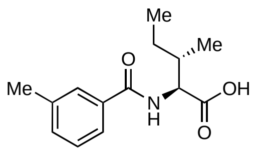

^{1}H NMR spectrum of **12**.

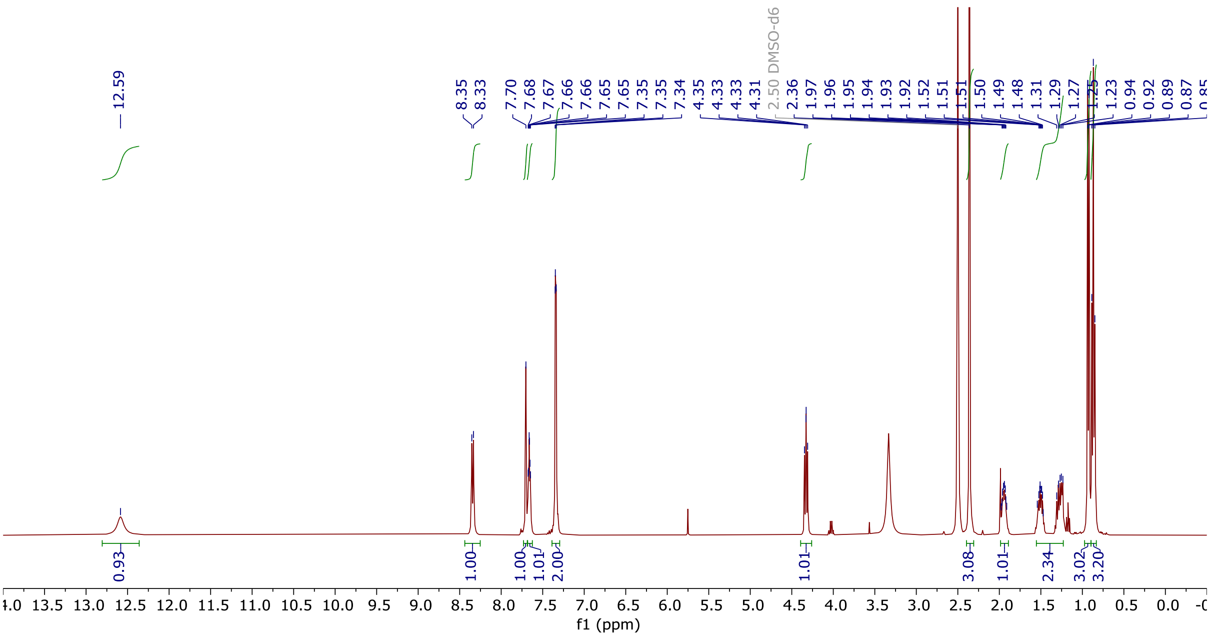

^{13}C NMR spectrum of **12**.

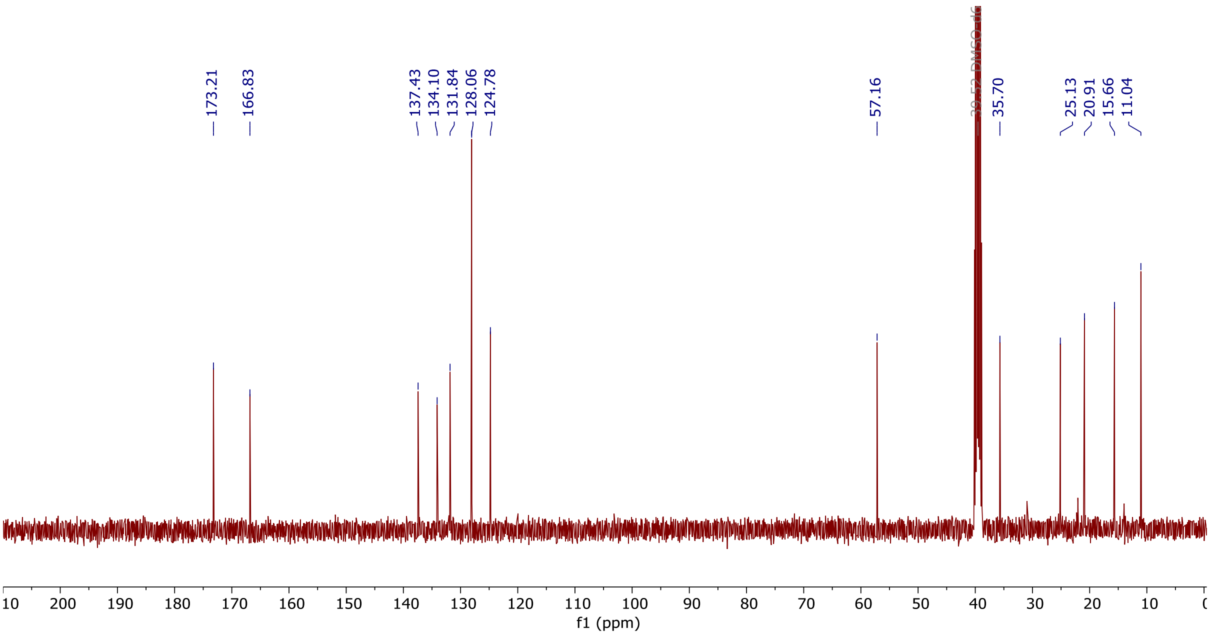

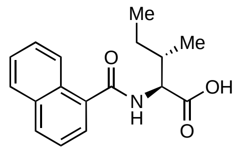

^{1}H NMR spectrum of **13**.

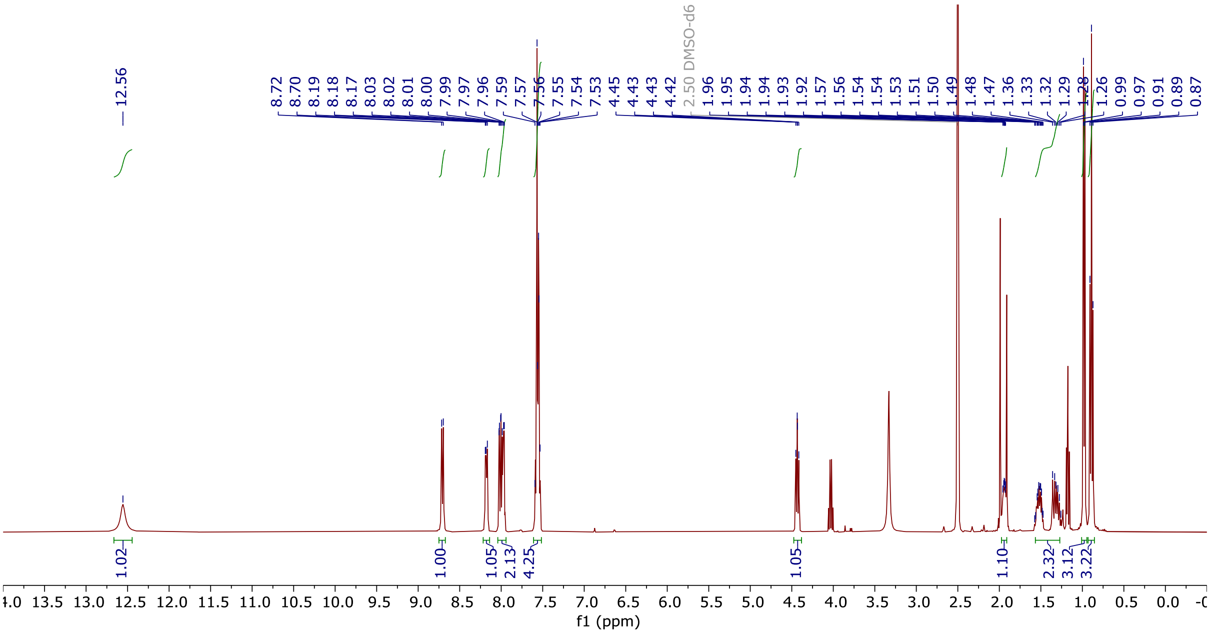

^{13}C NMR spectrum of **13**.

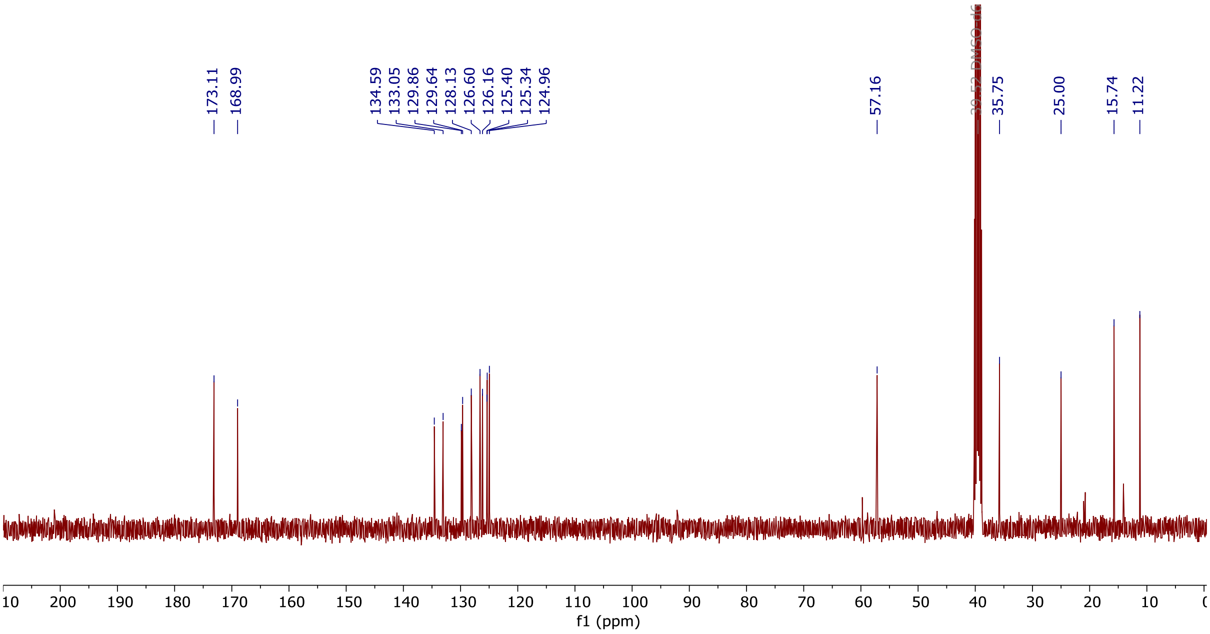

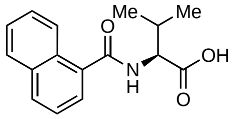

^{1}H NMR spectrum of **14**.

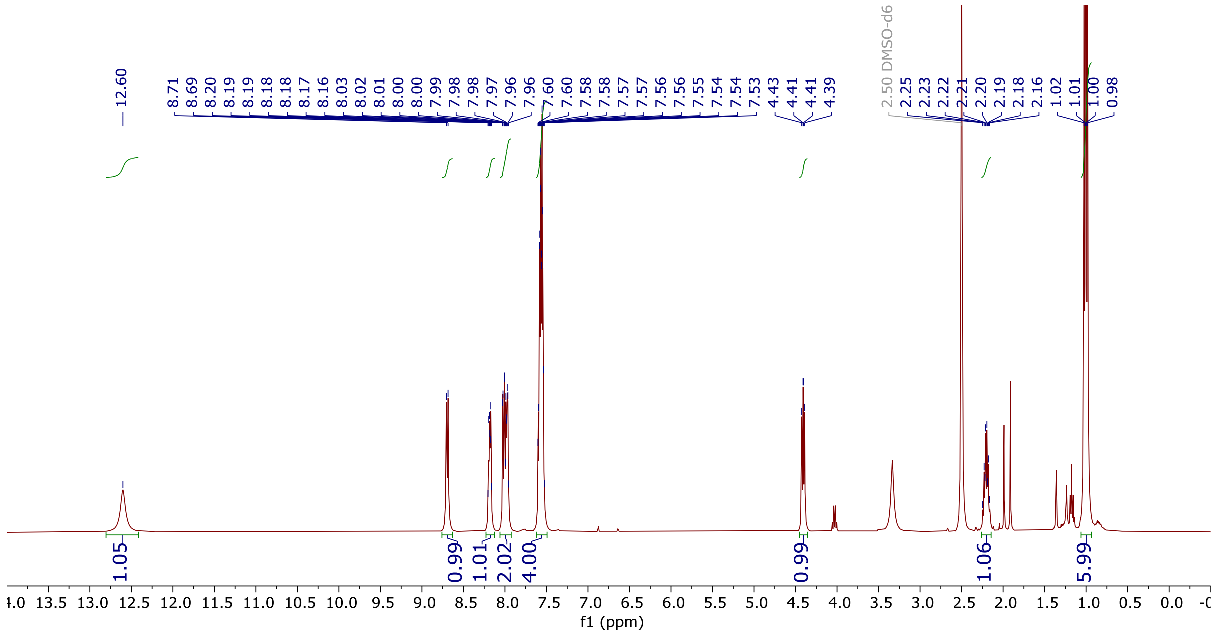

^{13}C NMR spectrum of **14**.

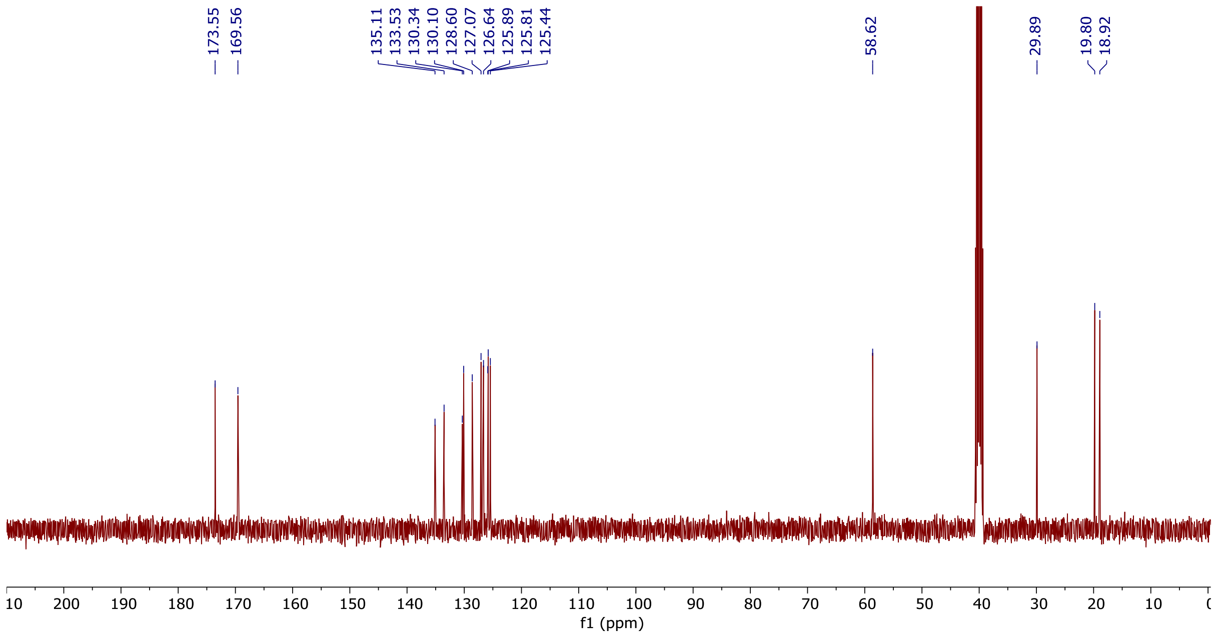

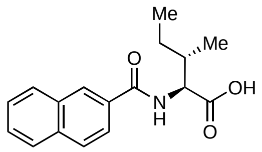

^{1}H NMR spectrum of **15**.

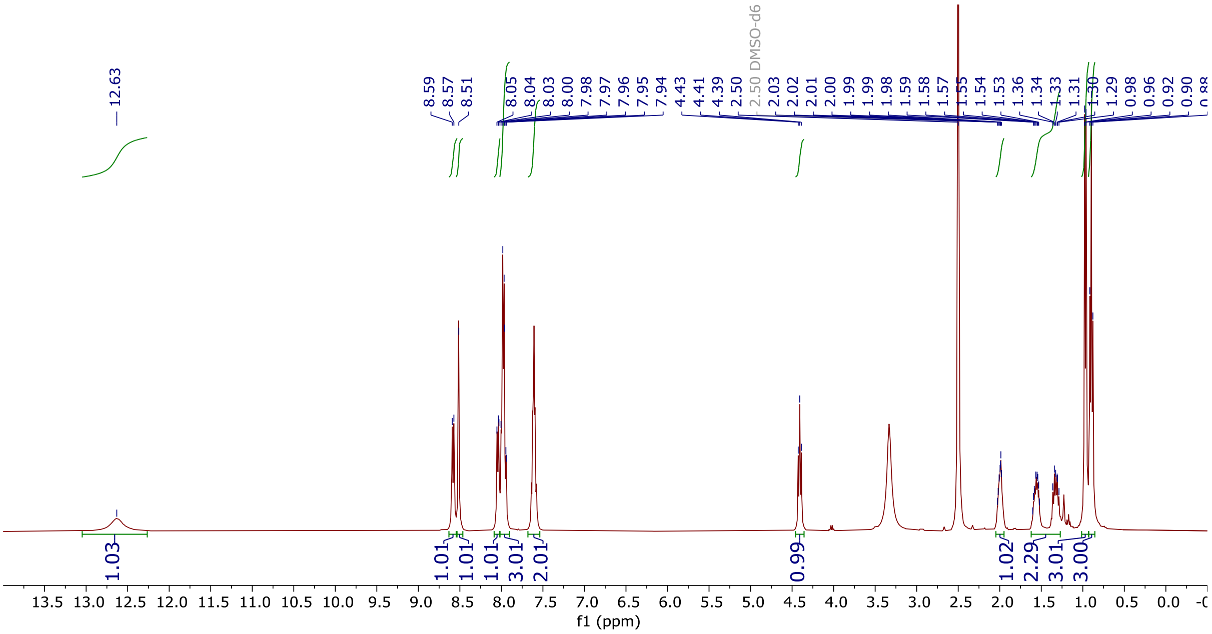

^{13}C NMR spectrum of **15**.

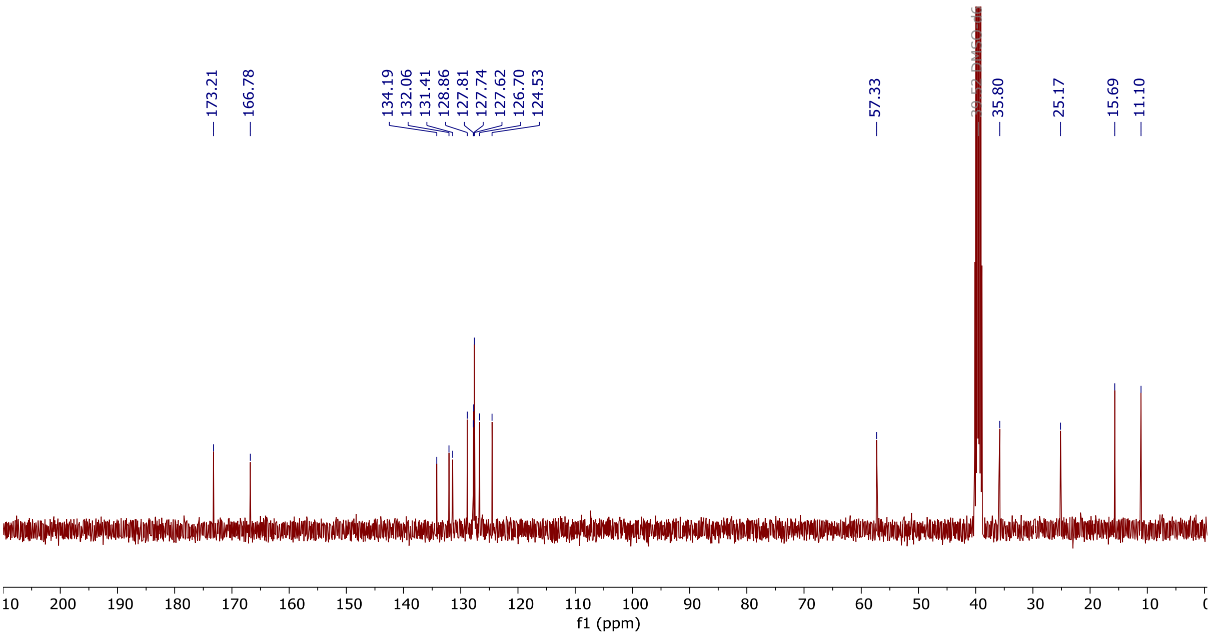

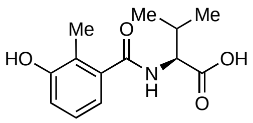

^{1}H NMR spectrum of **16**.

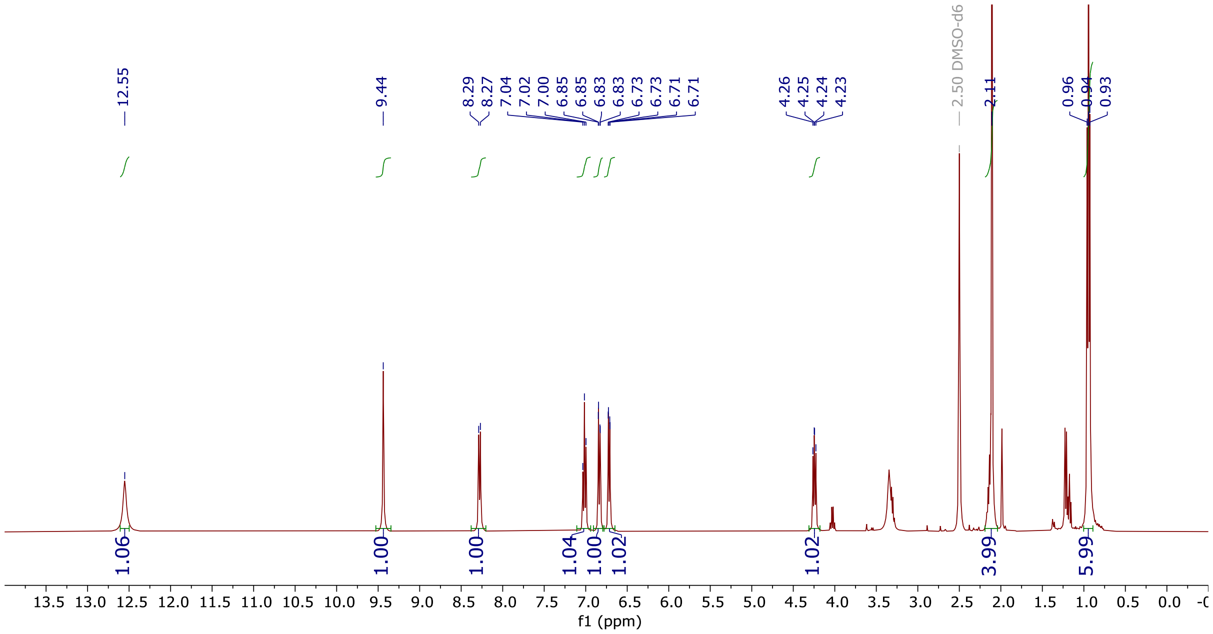

^{13}C NMR spectrum of **16**.

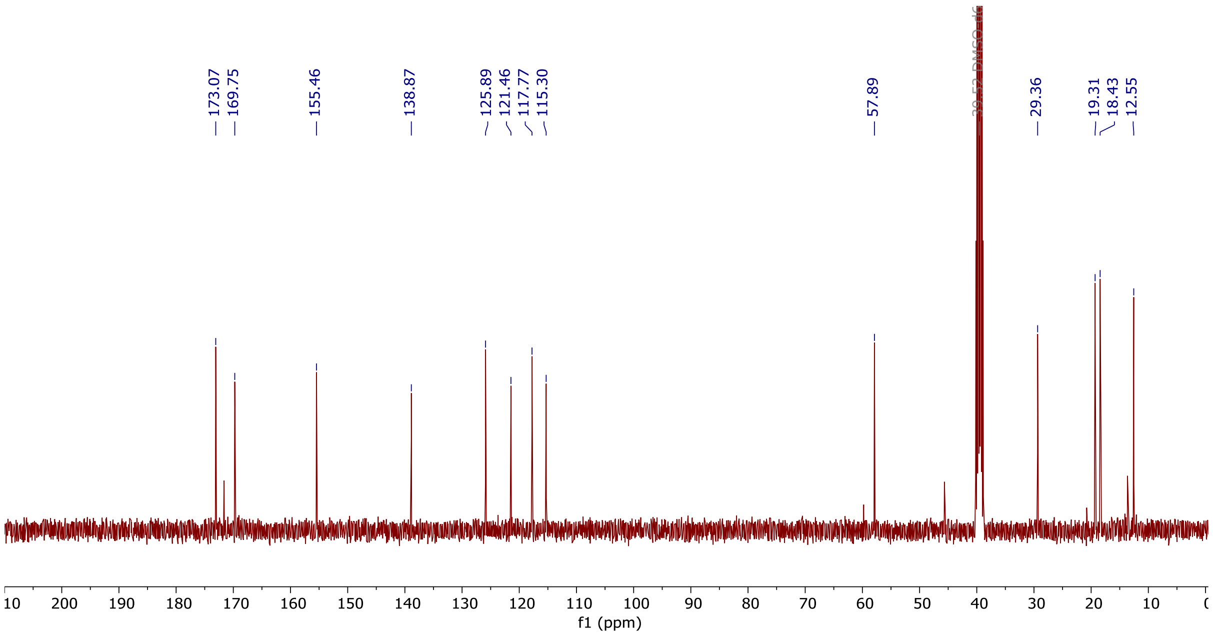

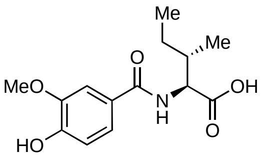

^{1}H NMR spectrum of **17**.

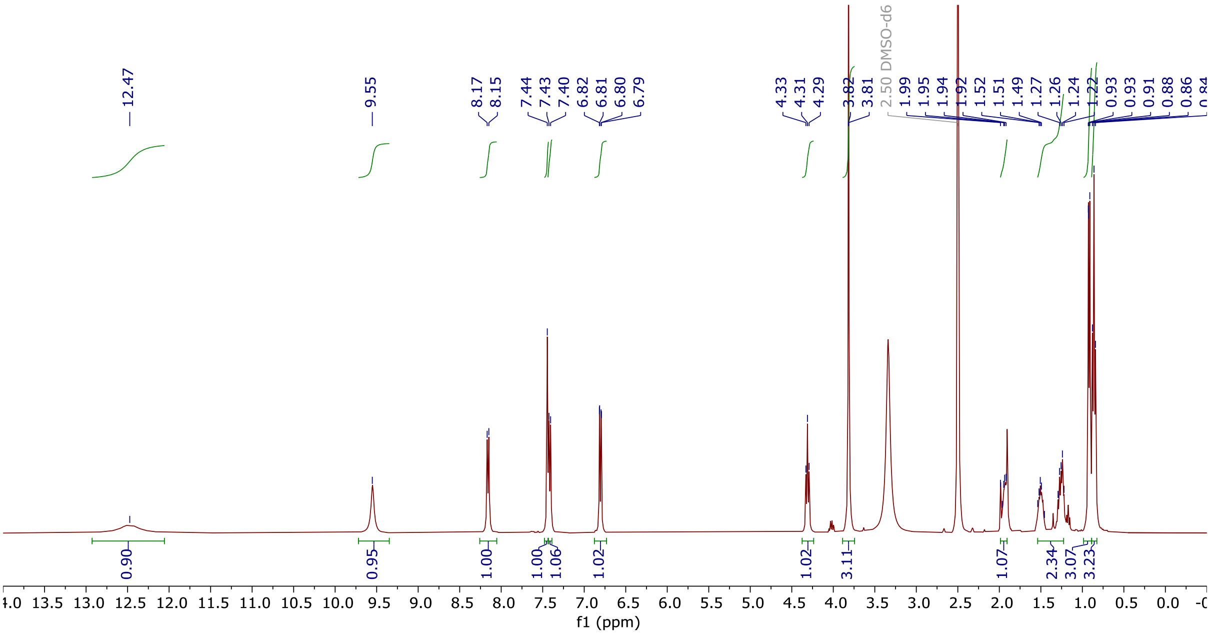

^{13}C NMR spectrum of **17**.

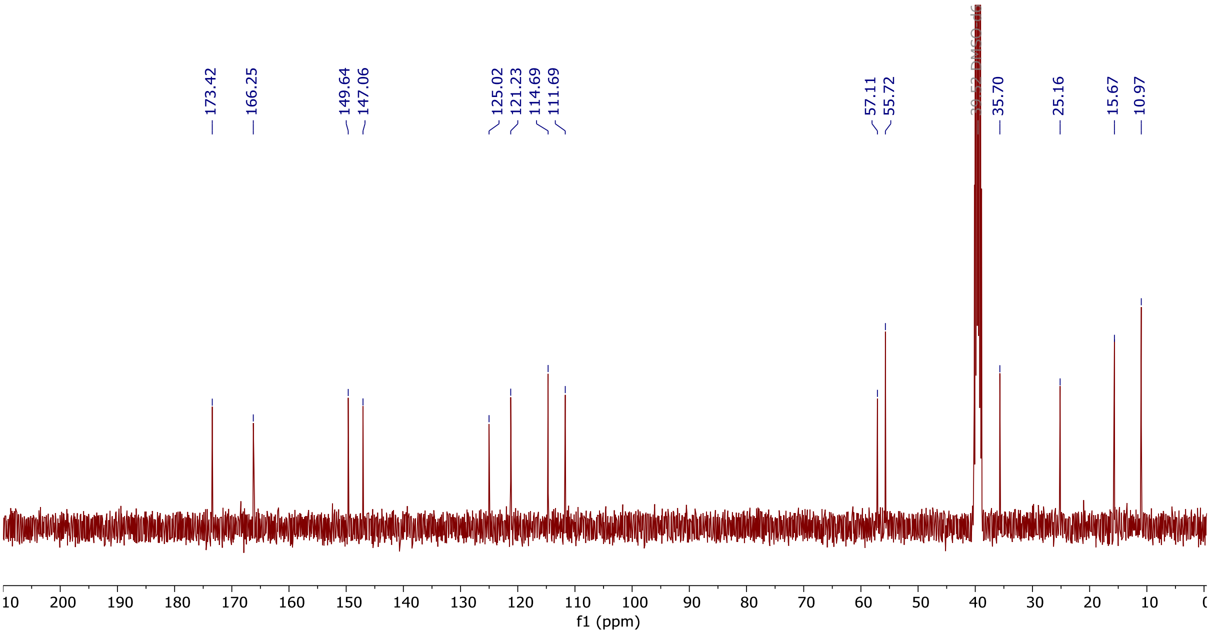

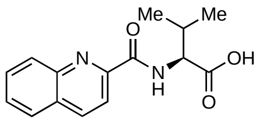

^{1}H NMR spectrum of **18**.

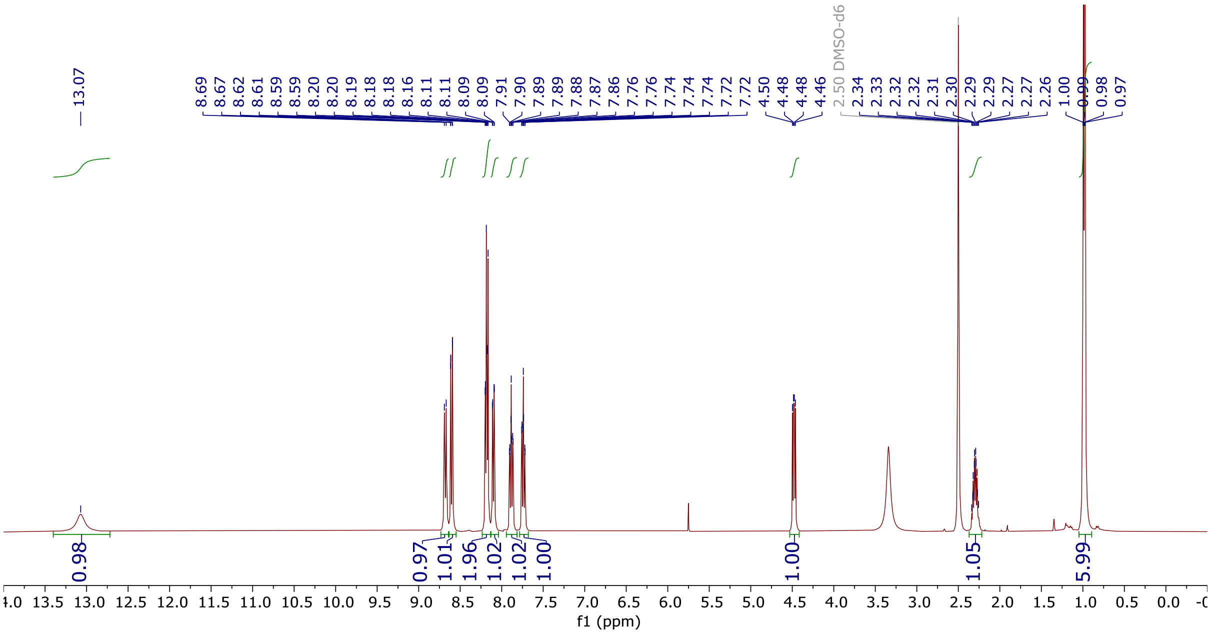

^{13}C NMR spectrum of **18**.

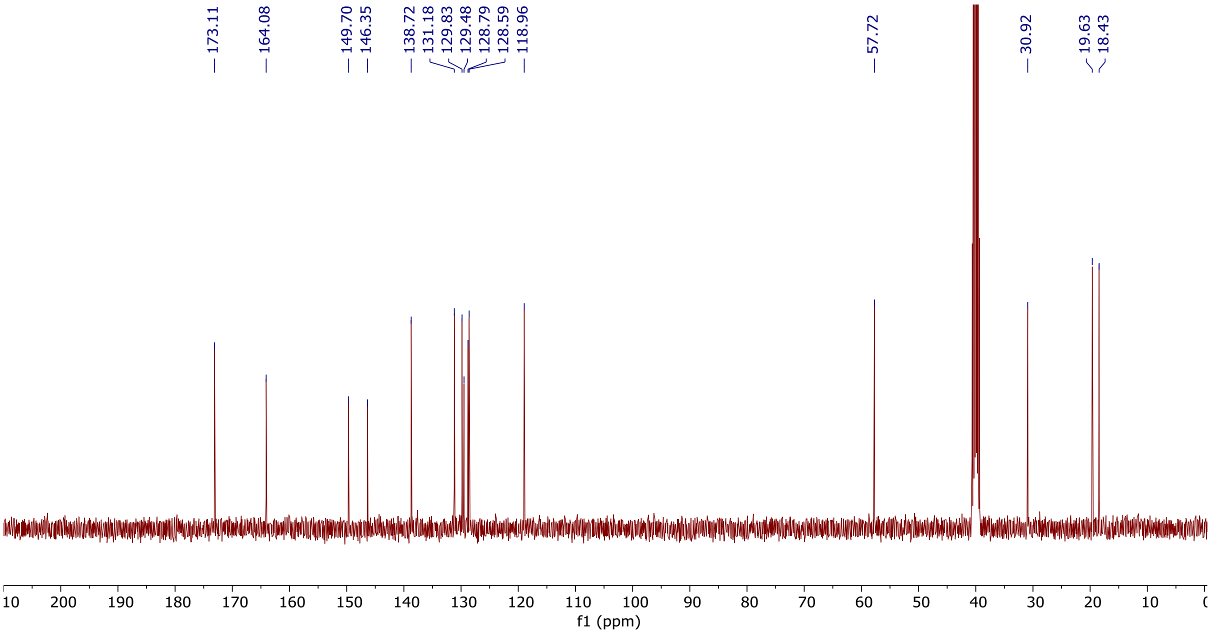

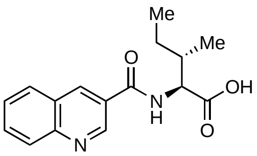

^{1}H NMR spectrum of **19**.

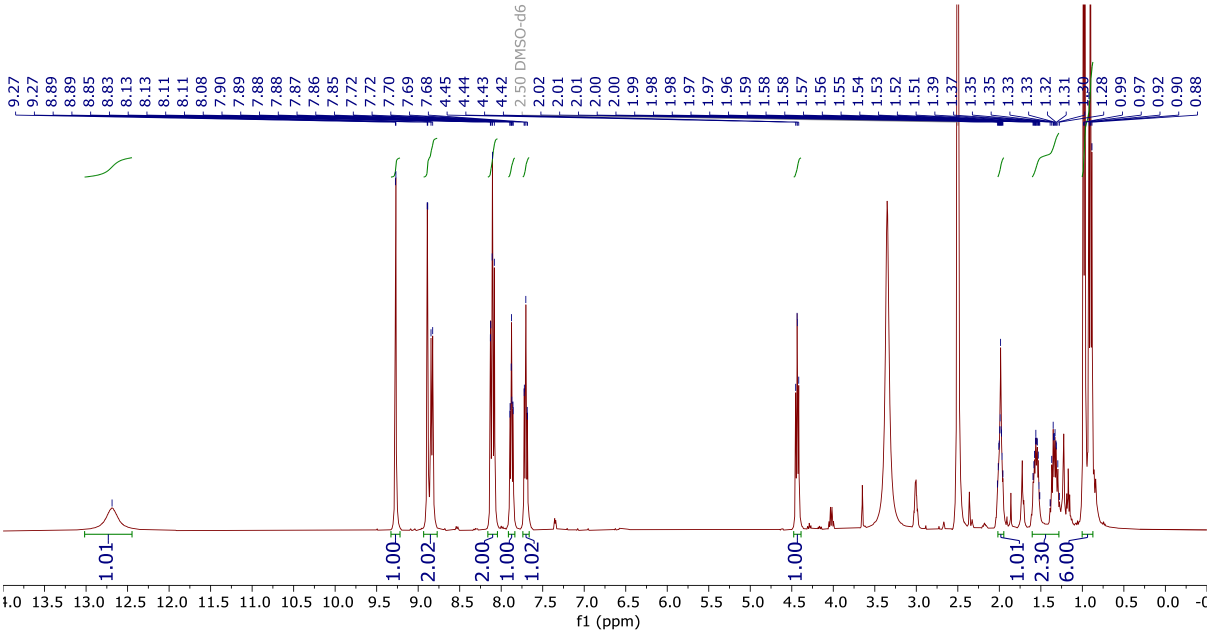

^{13}C NMR spectrum of **19**.

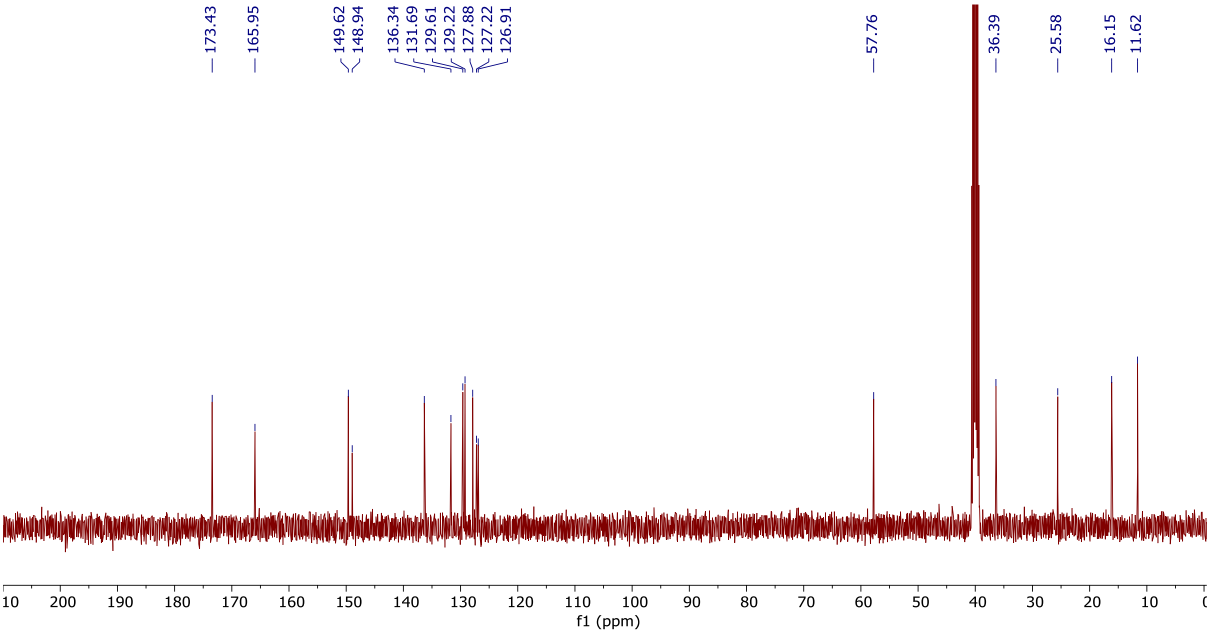

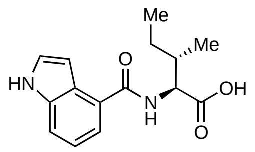

^{1}H NMR spectrum of **20**.

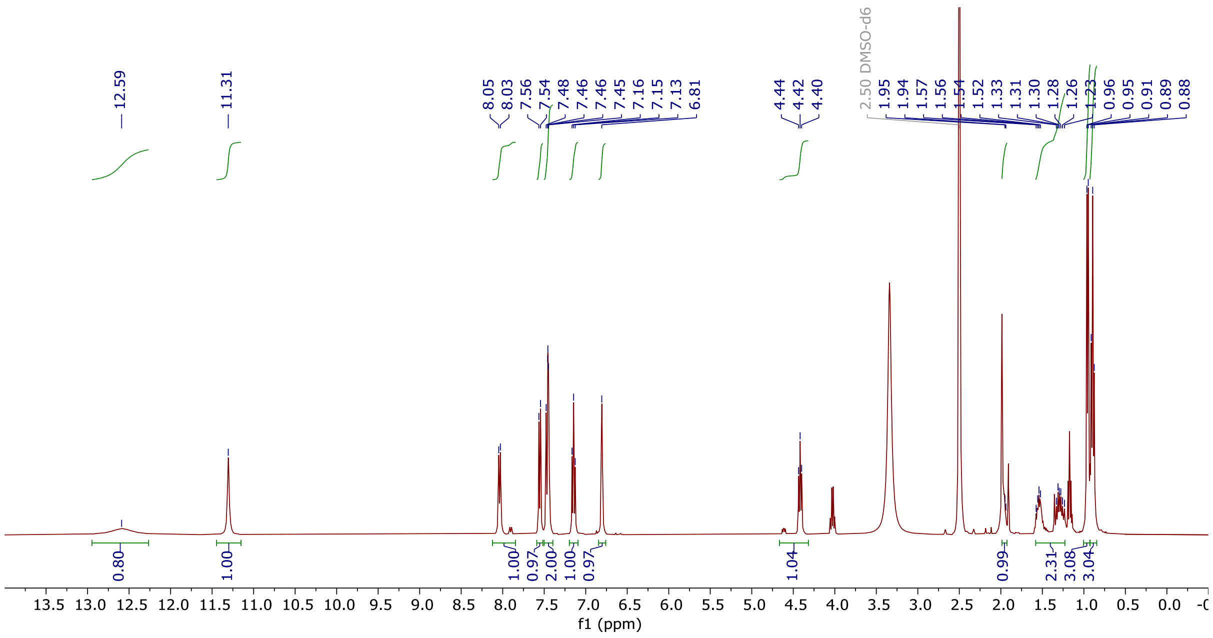

^{13}C NMR spectrum of **20**.

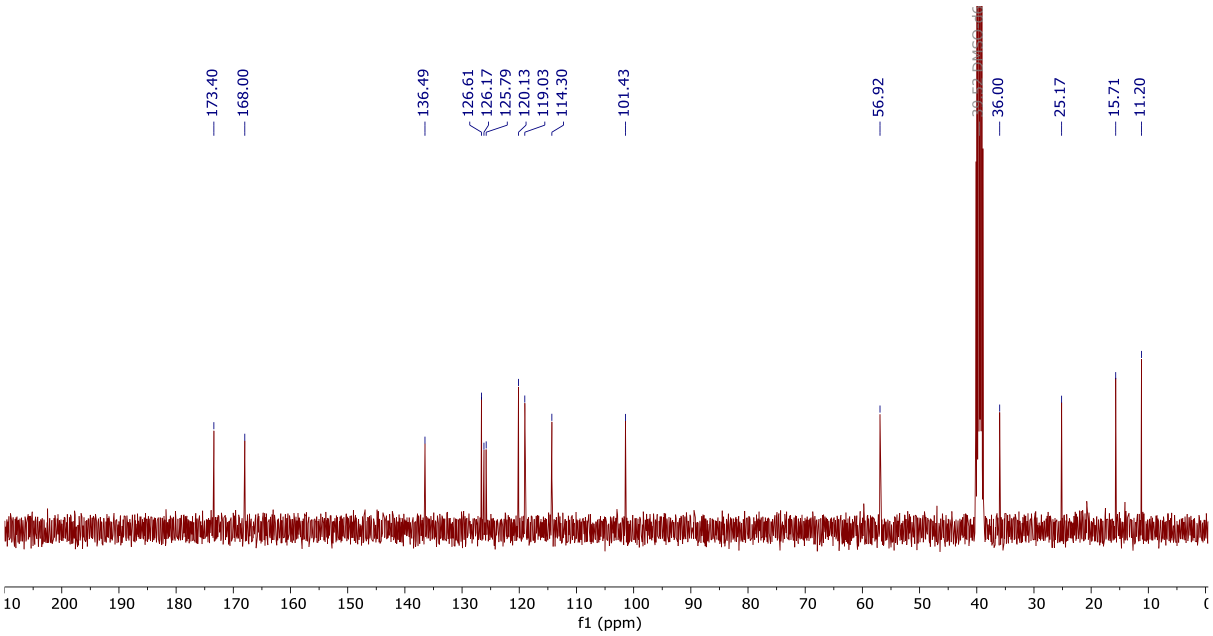

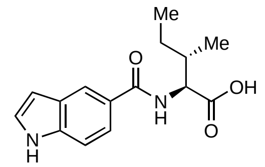

^{1}H NMR spectrum of **21**.

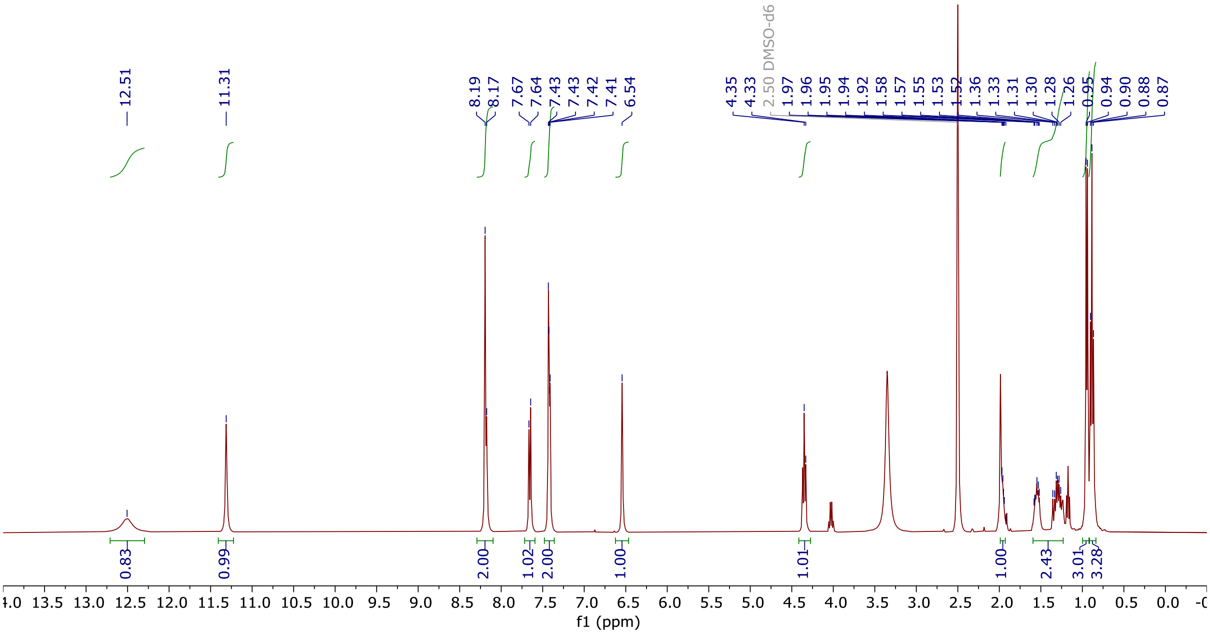

^{13}C NMR spectrum of **21**.

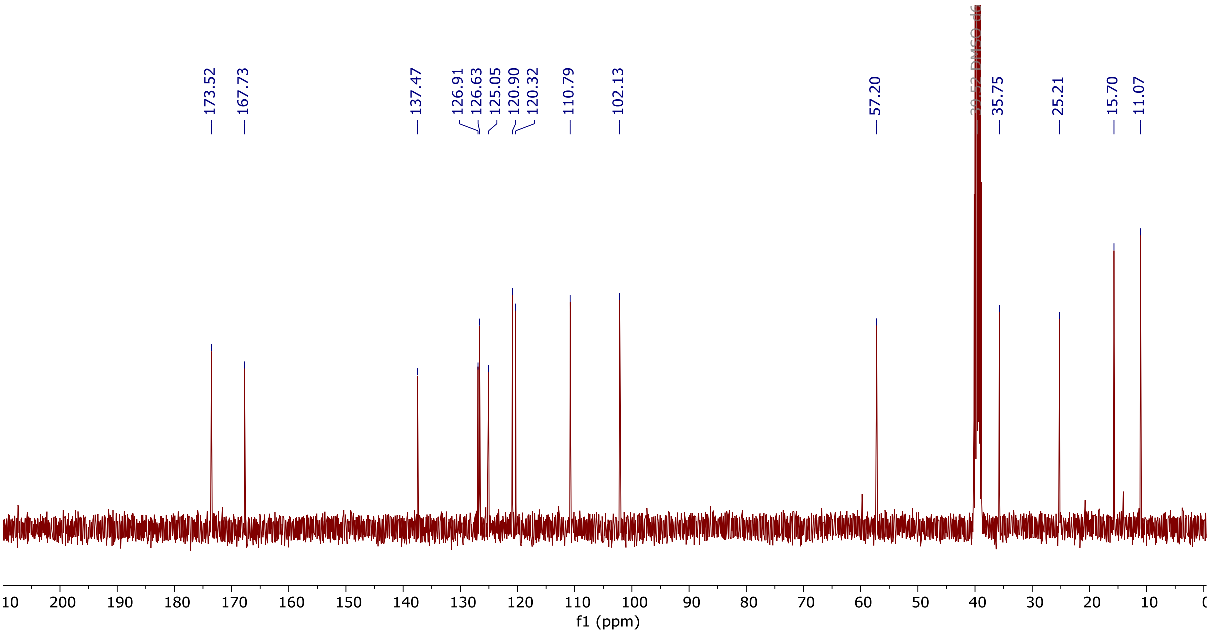

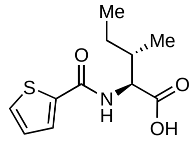

^{1}H NMR spectrum of **22**.

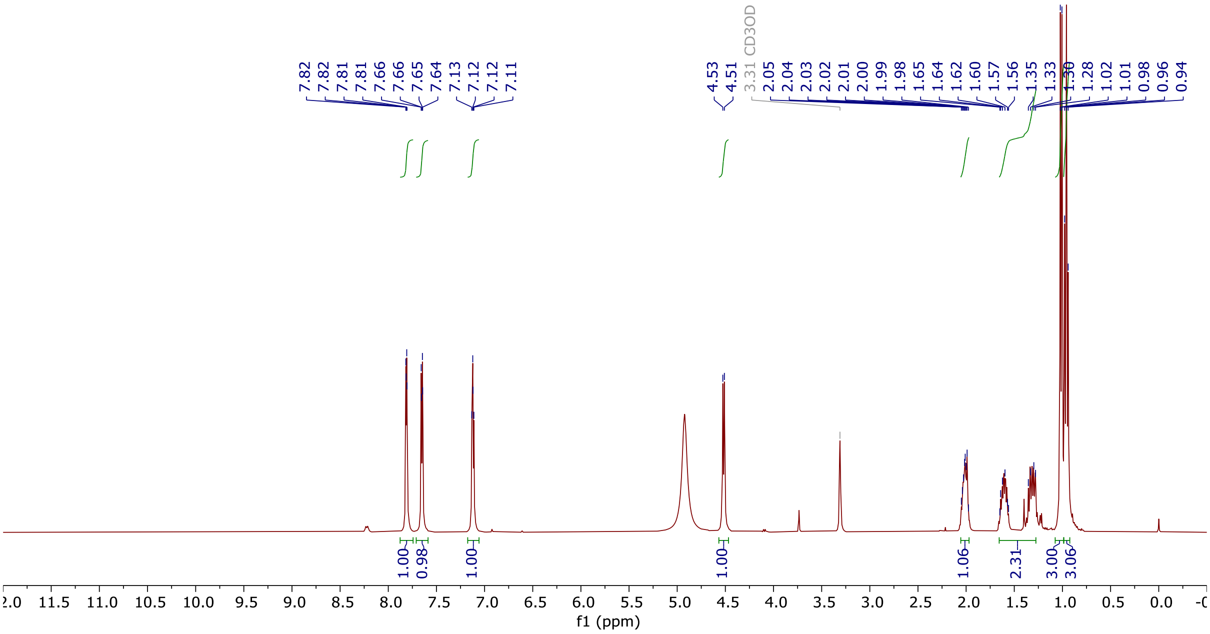

^{13}C NMR spectrum of **22**.

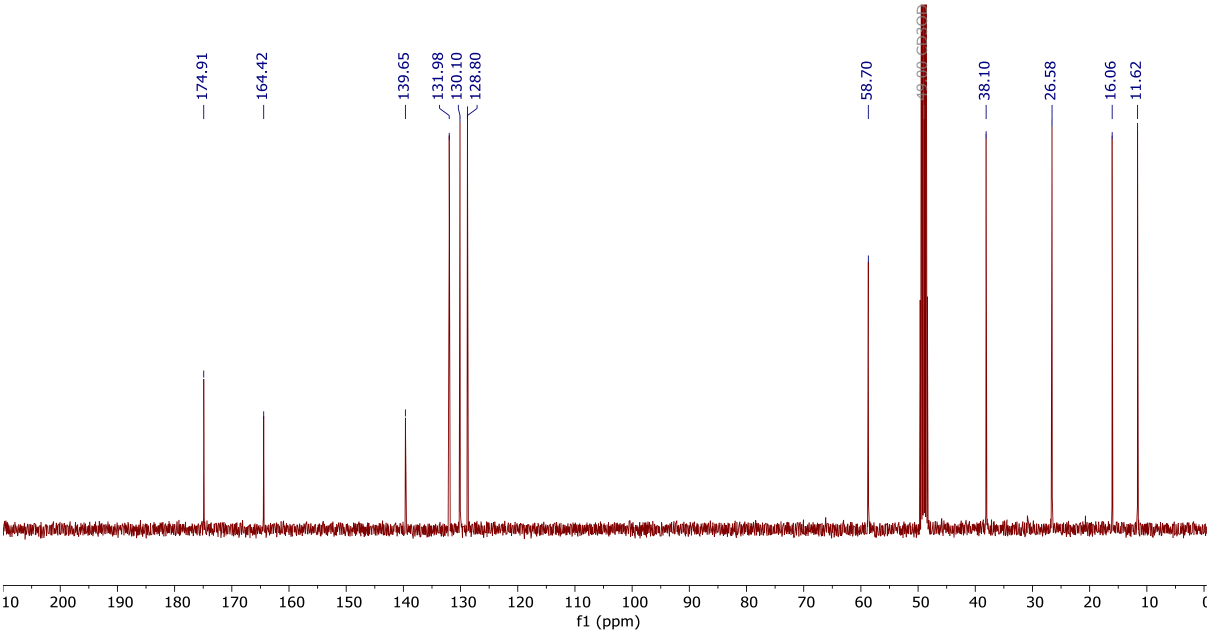

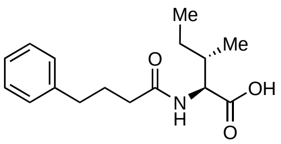

^{1}H NMR spectrum of **23**.

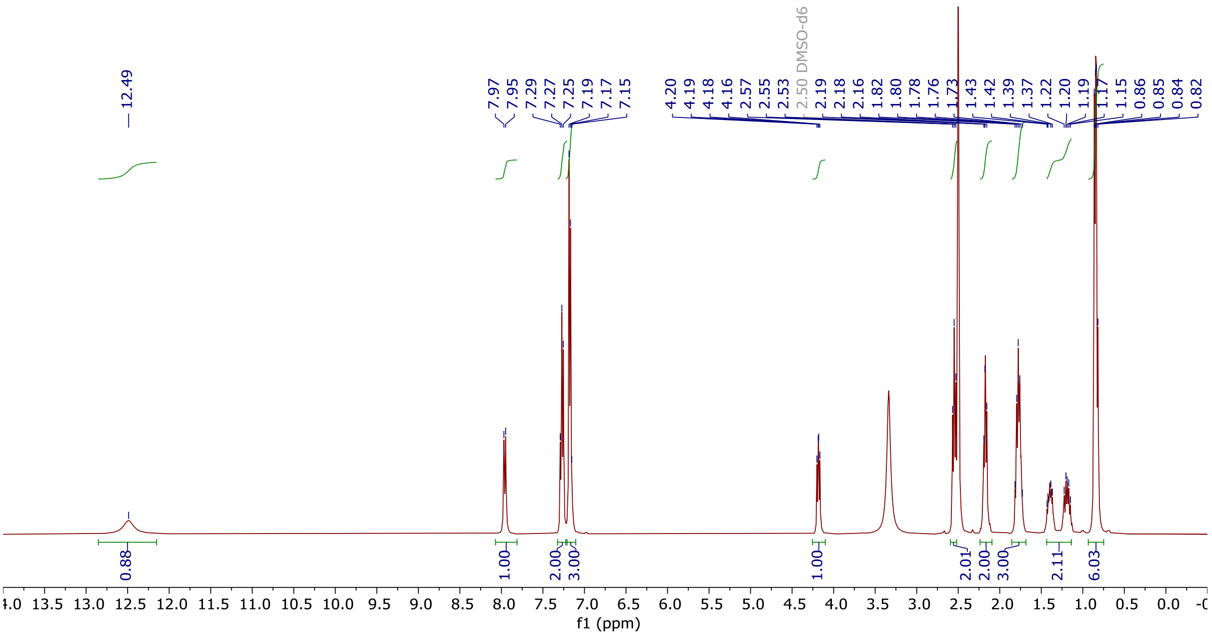

^{13}C NMR spectrum of **23**.

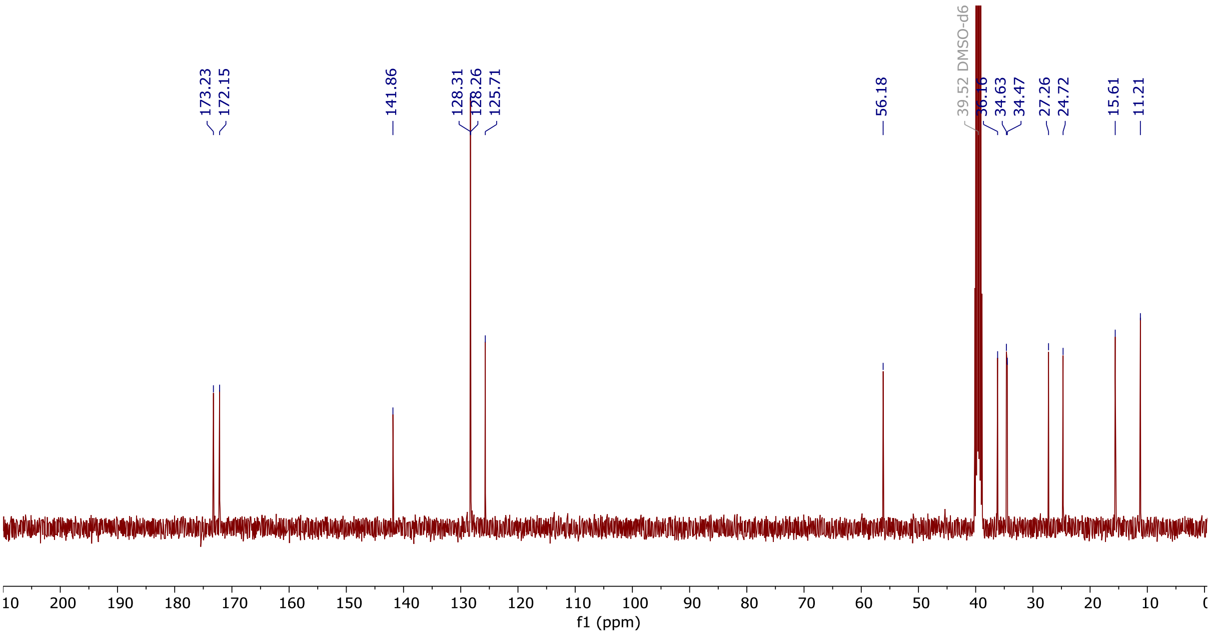

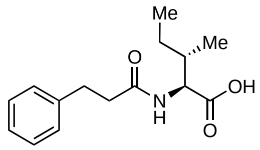

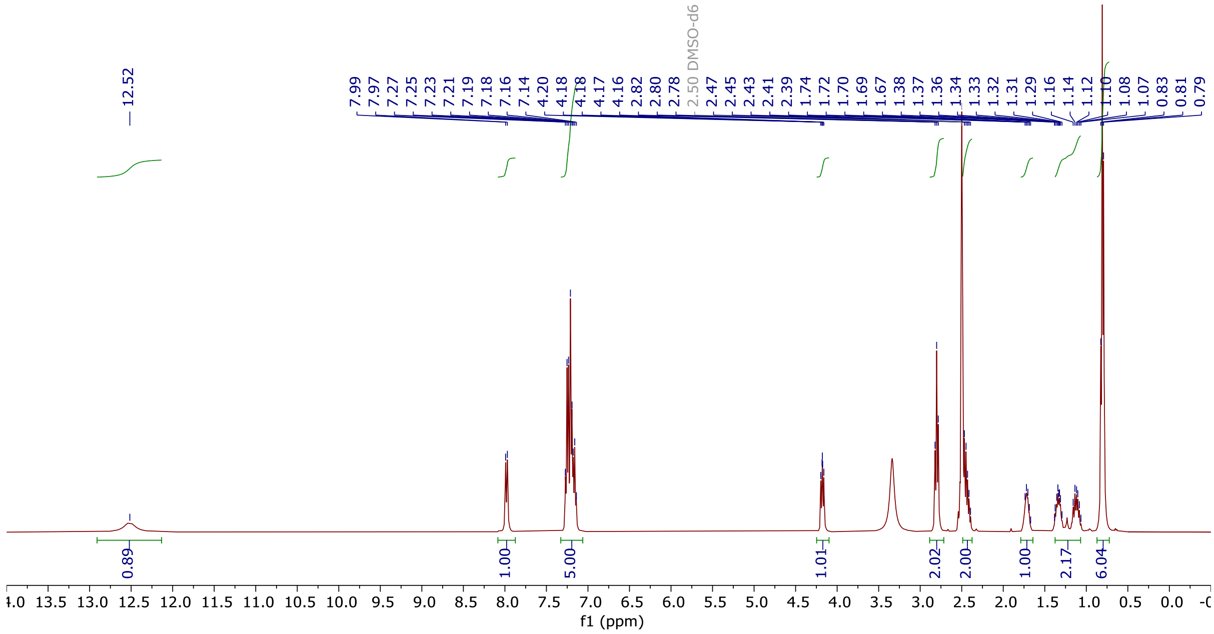

^{1}H NMR spectrum of **24**.

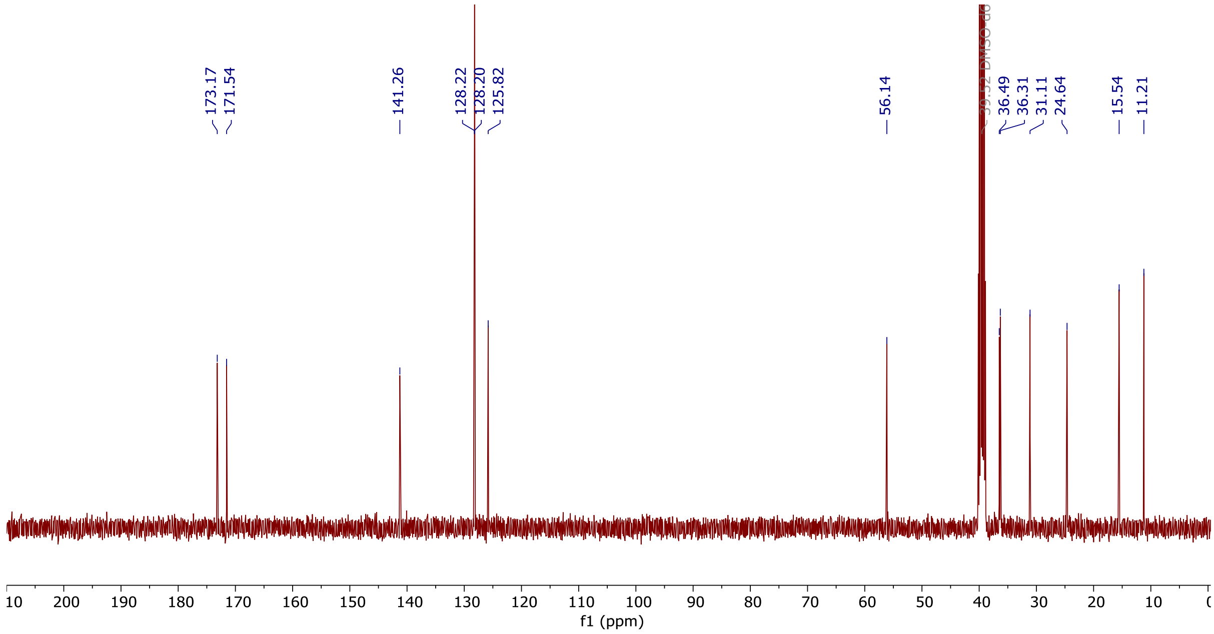

^{13}C NMR spectrum of **24**.

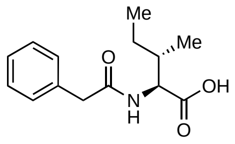

^{1}H NMR spectrum of **25**.

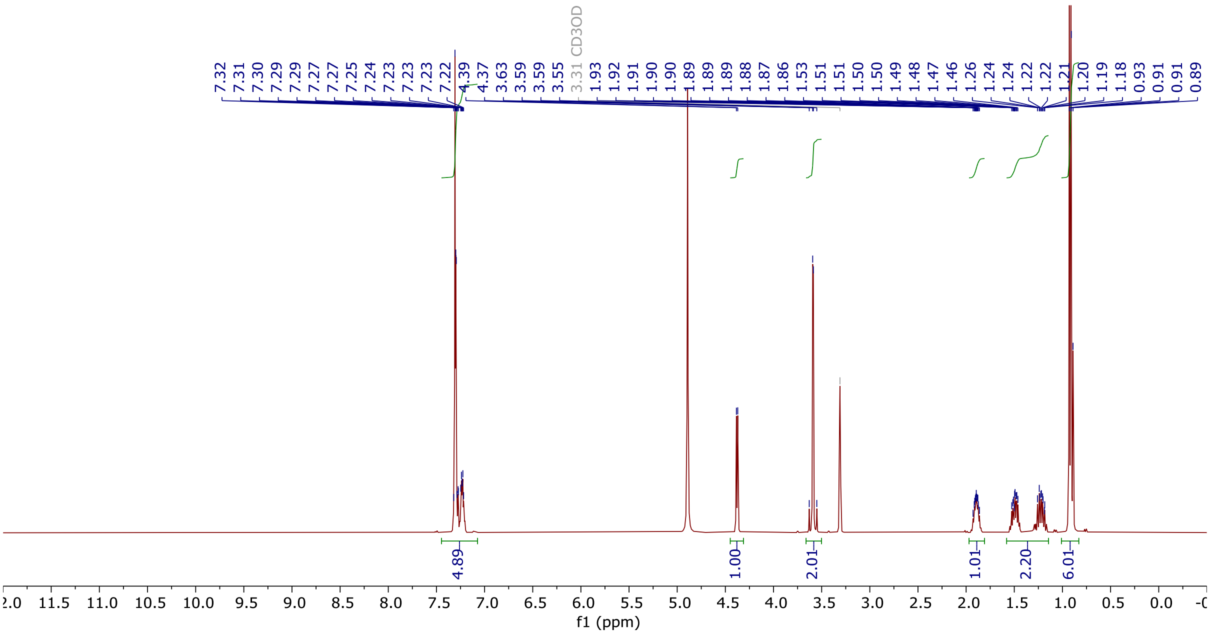

^{13}C NMR spectrum of **25**.

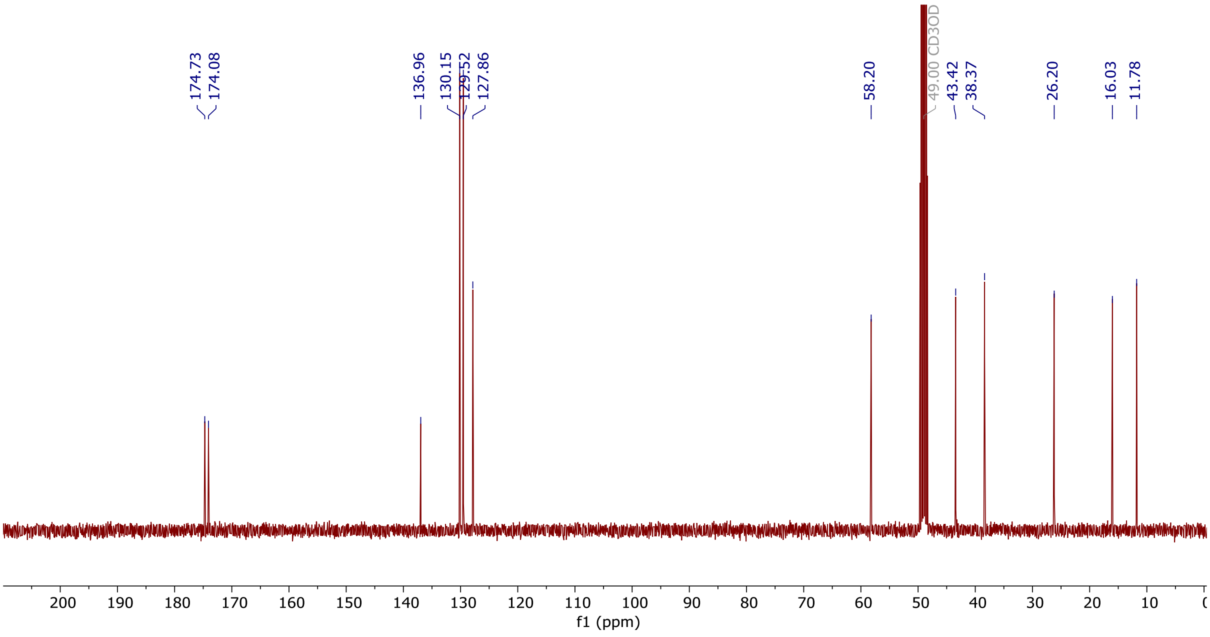

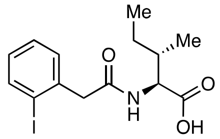

^{1}H NMR spectrum of **26**.

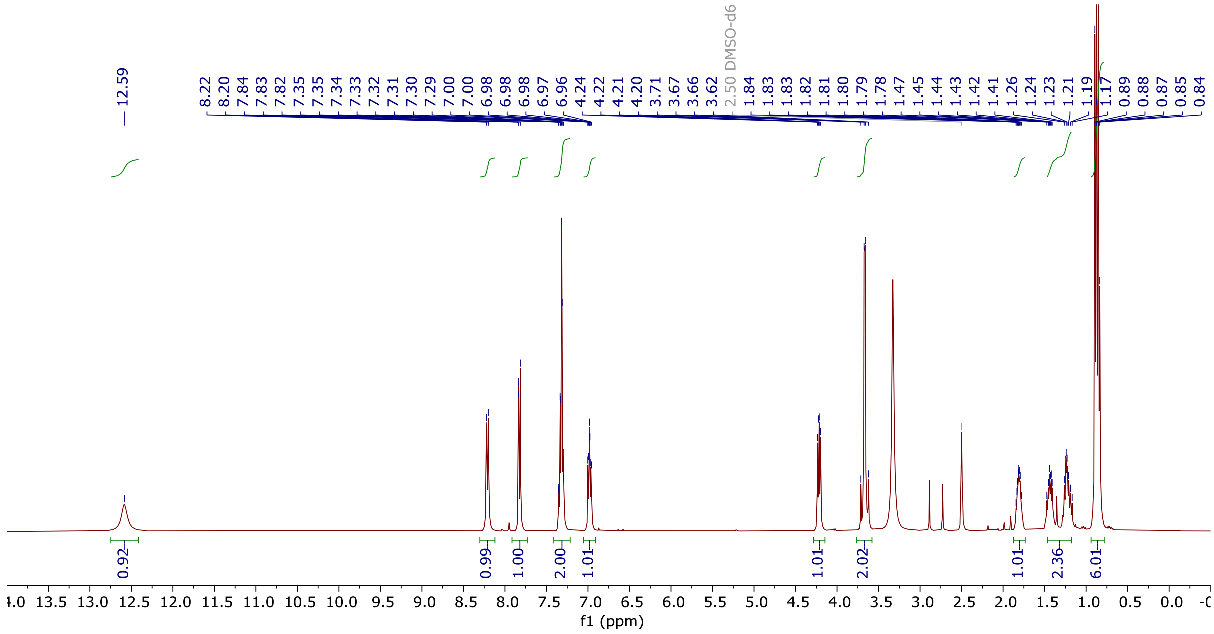

^{13}C NMR spectrum of **26**.

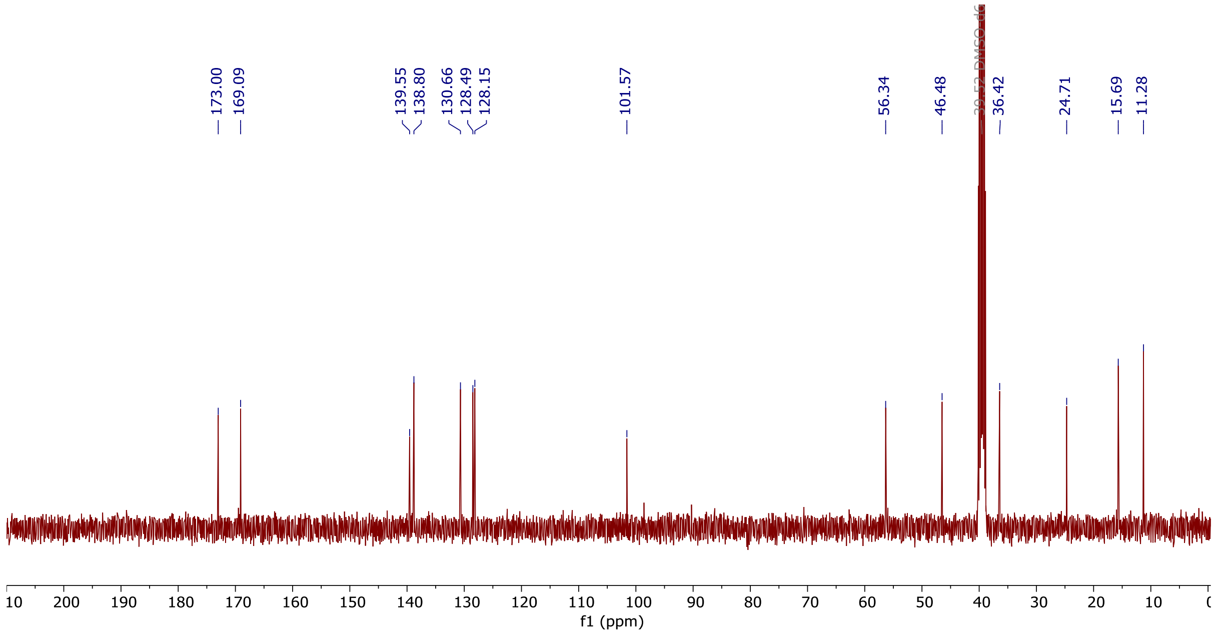

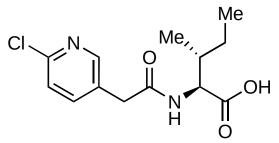

^{1}H NMR spectrum of **27**.

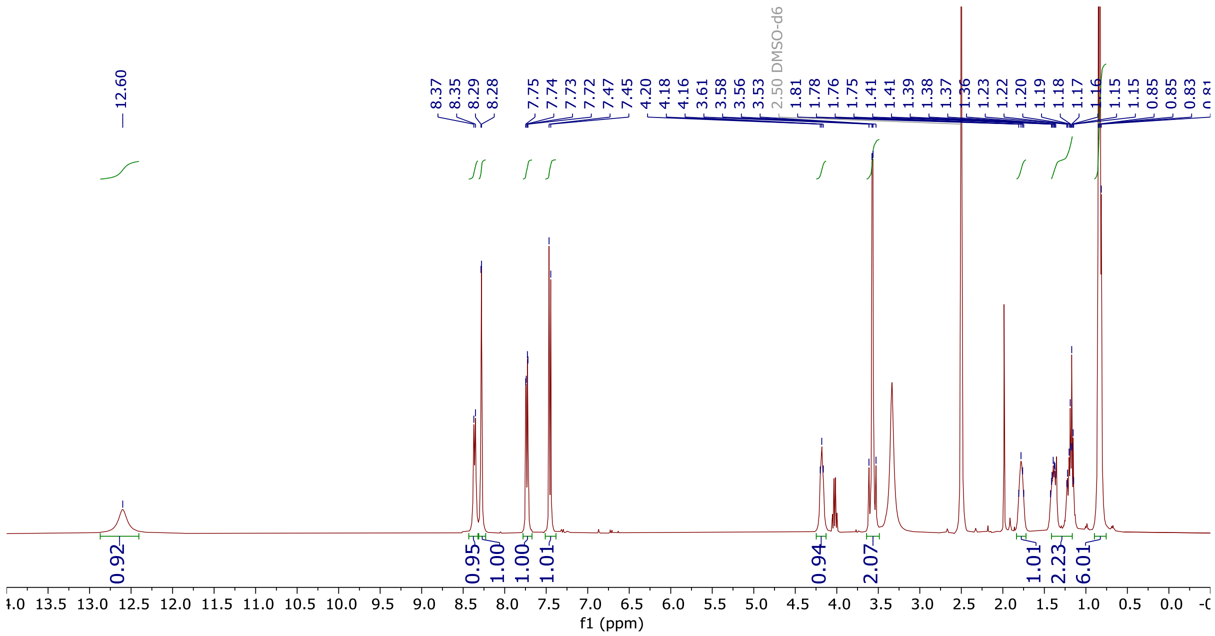

^{13}C NMR spectrum of **27**.

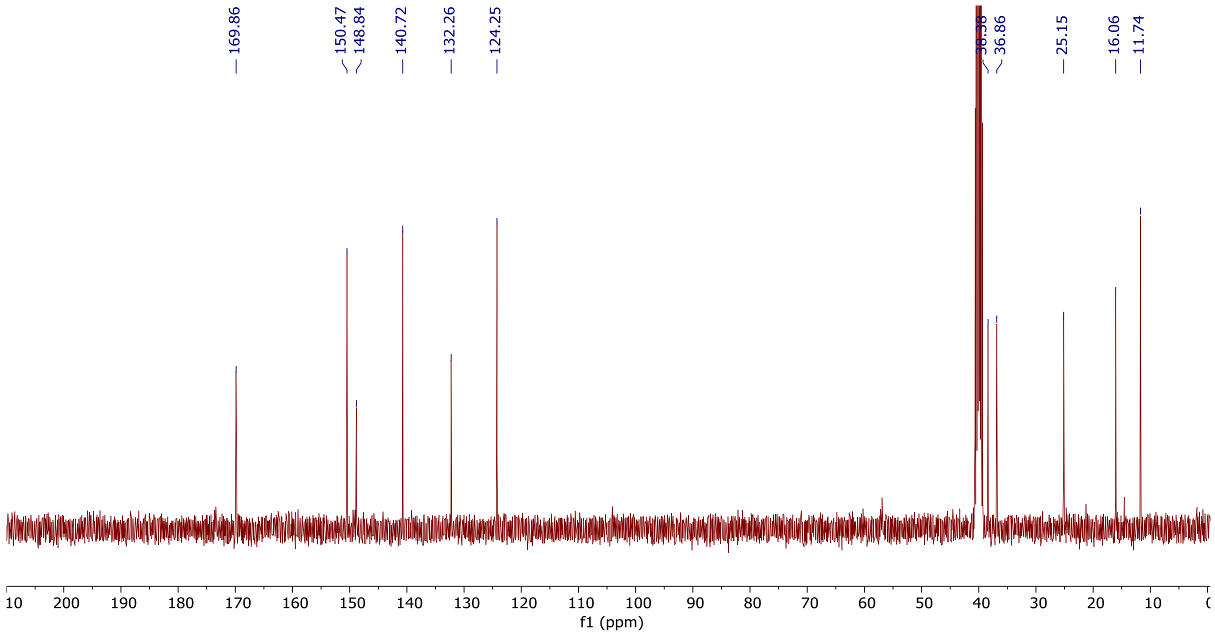

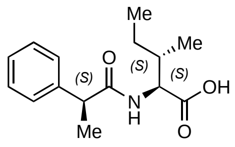

^{1}H NMR spectrum of **28**.

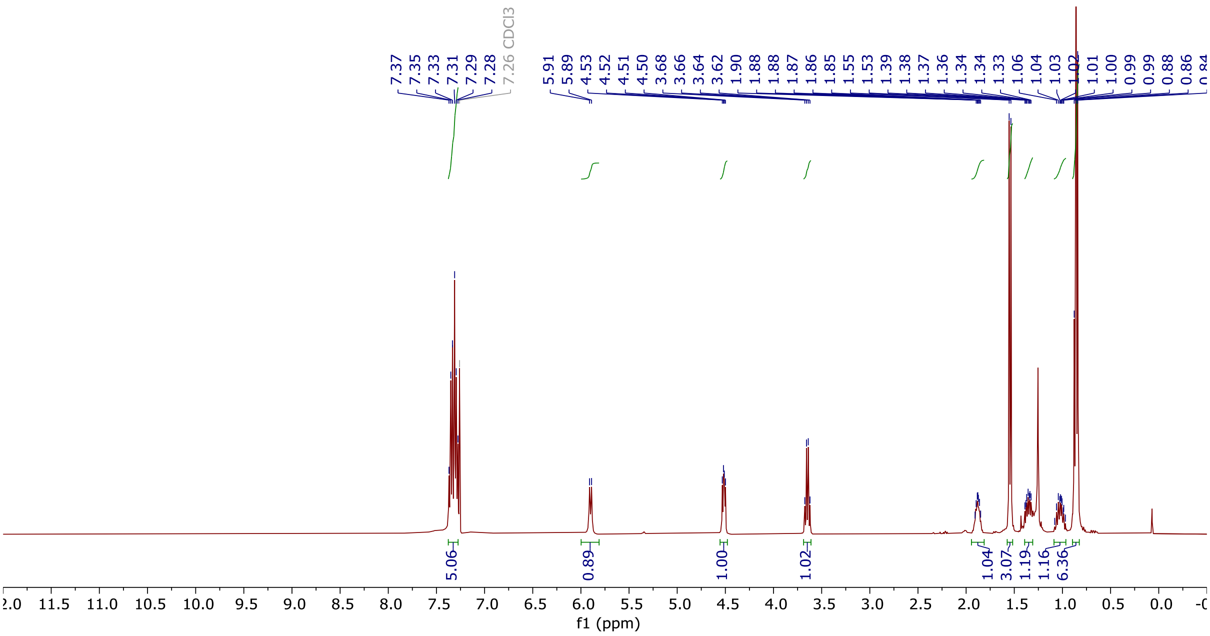

^{13}C NMR spectrum of **28**.

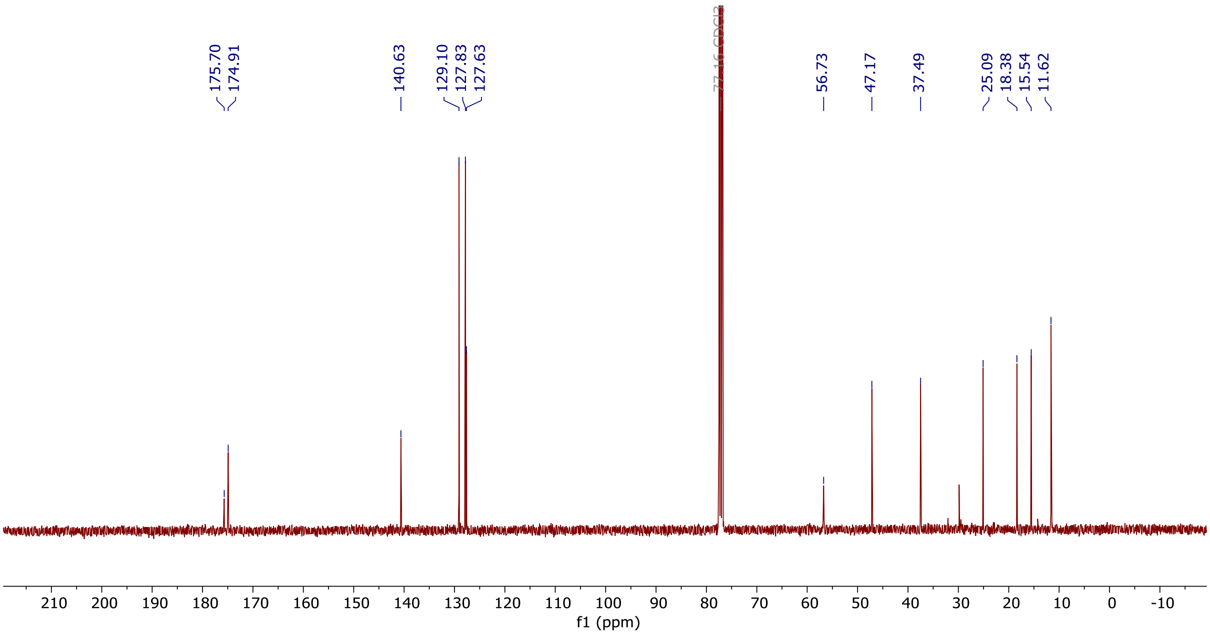

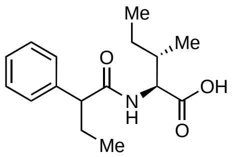

^{1}H NMR spectrum of **29**.

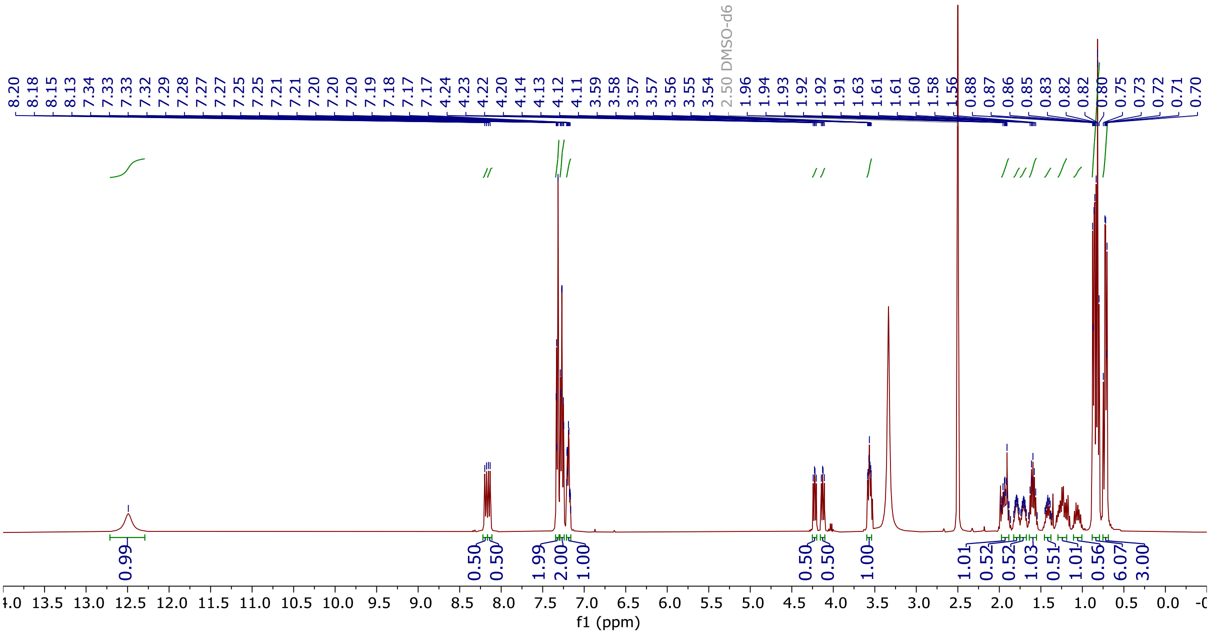

^{13}C NMR spectrum of **29**.

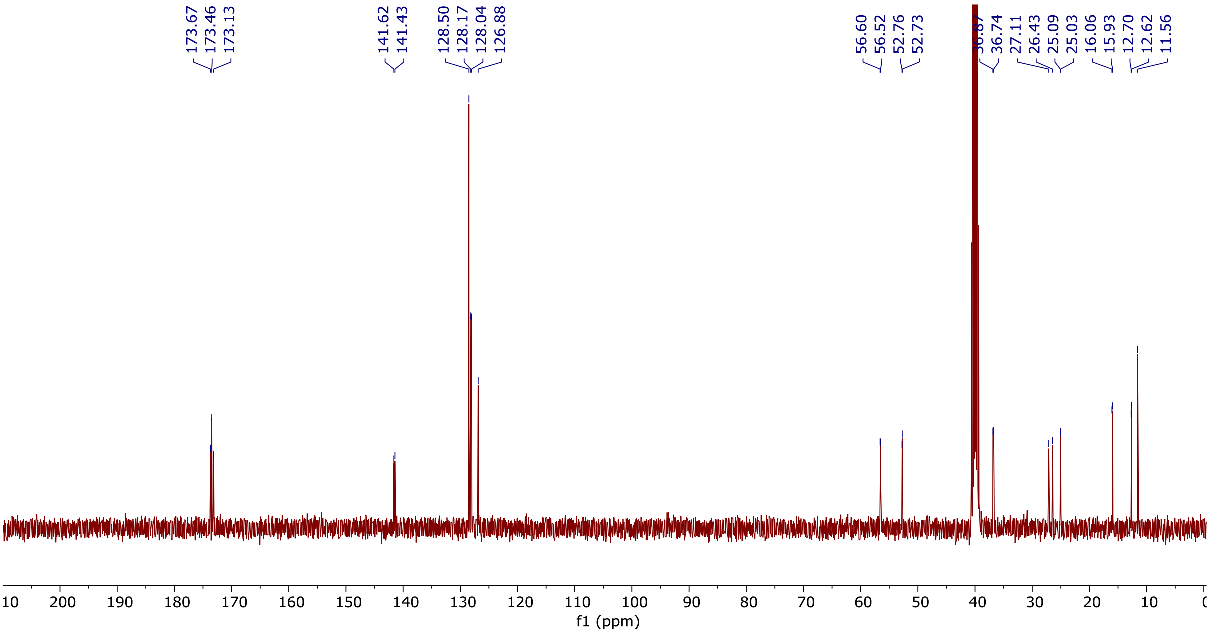

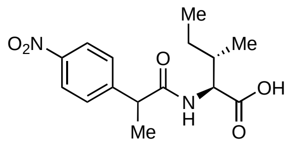

^{1}H NMR spectrum of **30**.

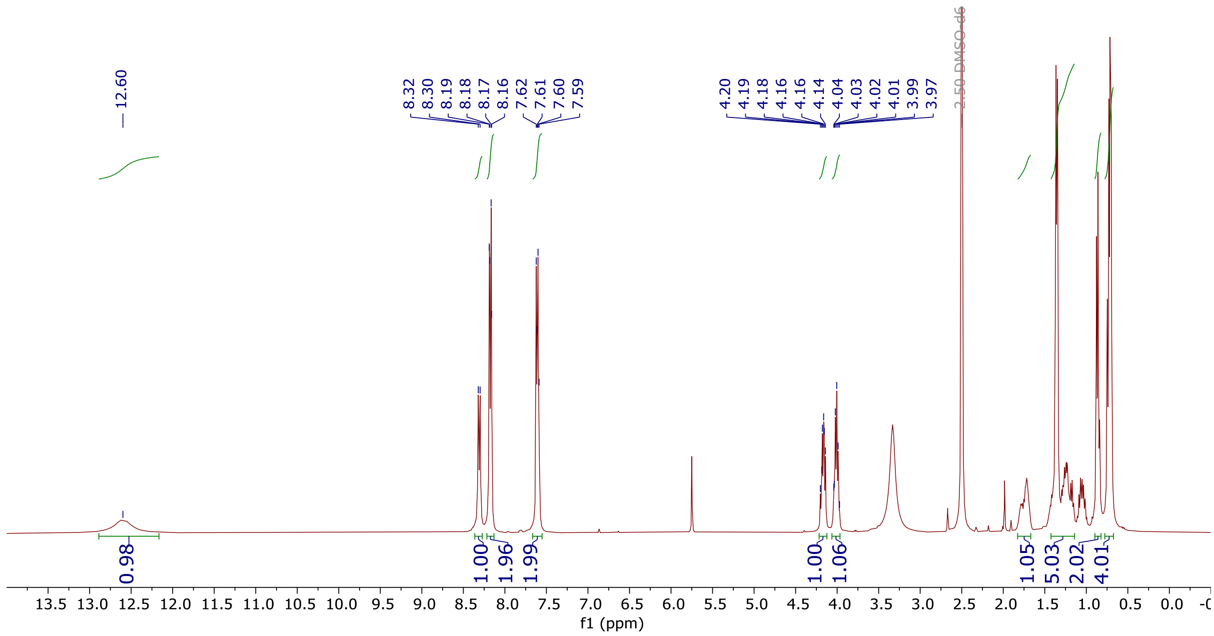

^{13}C NMR spec spectrum of **30**.

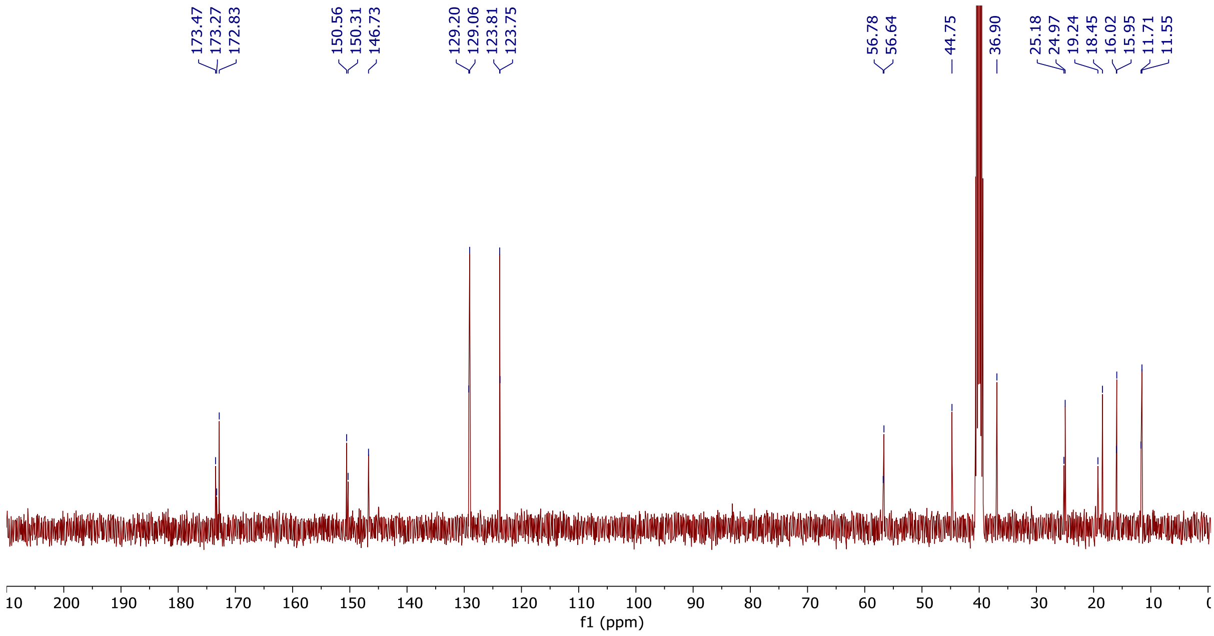

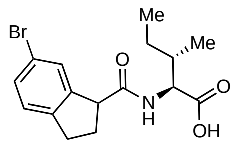

^{1}H NMR spectrum of **31**.

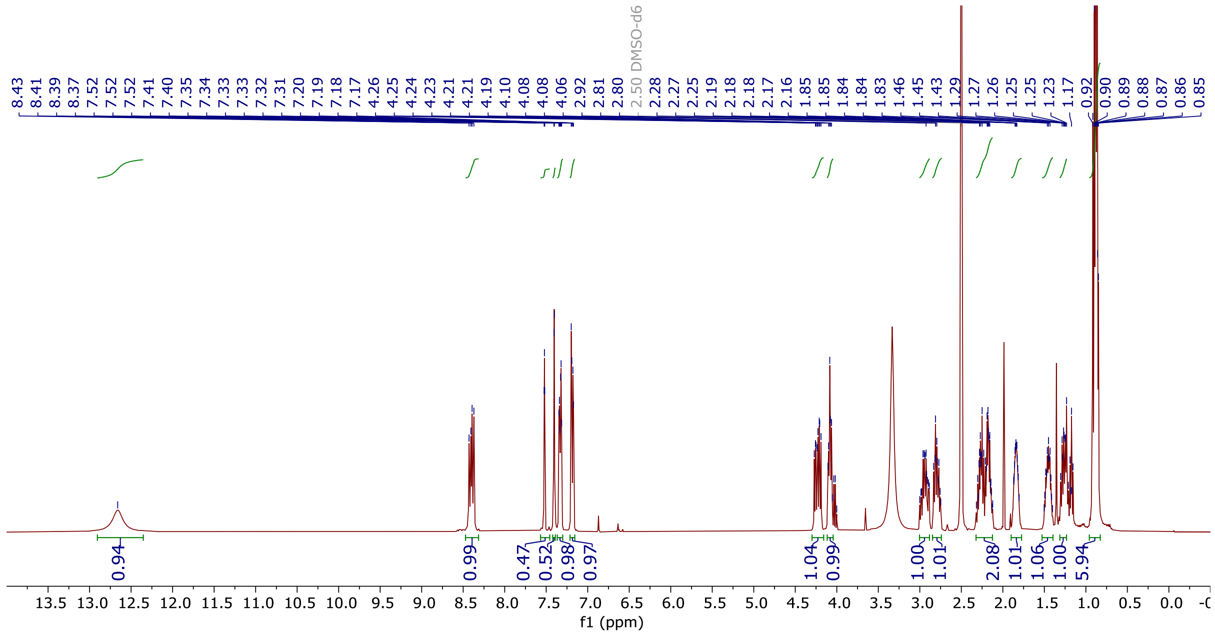

^{13}C NMR spectrum of **31**.

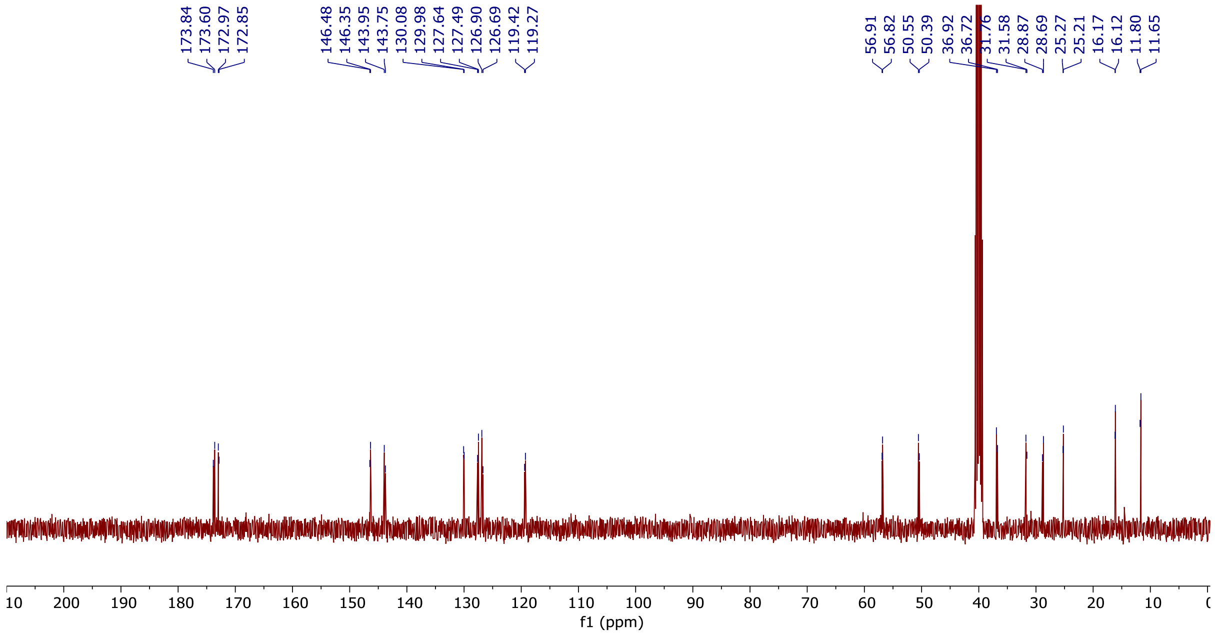

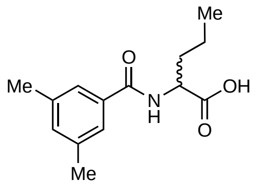

^{1}H NMR spectrum of **32**.

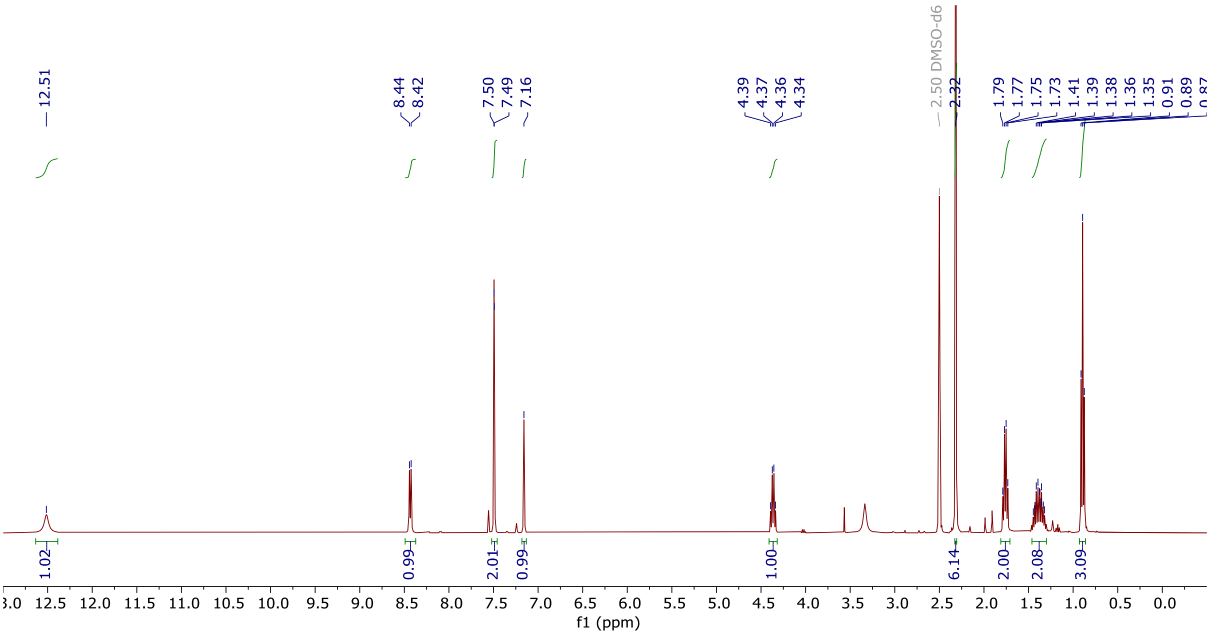

^{13}C NMR spectrum of **32**.

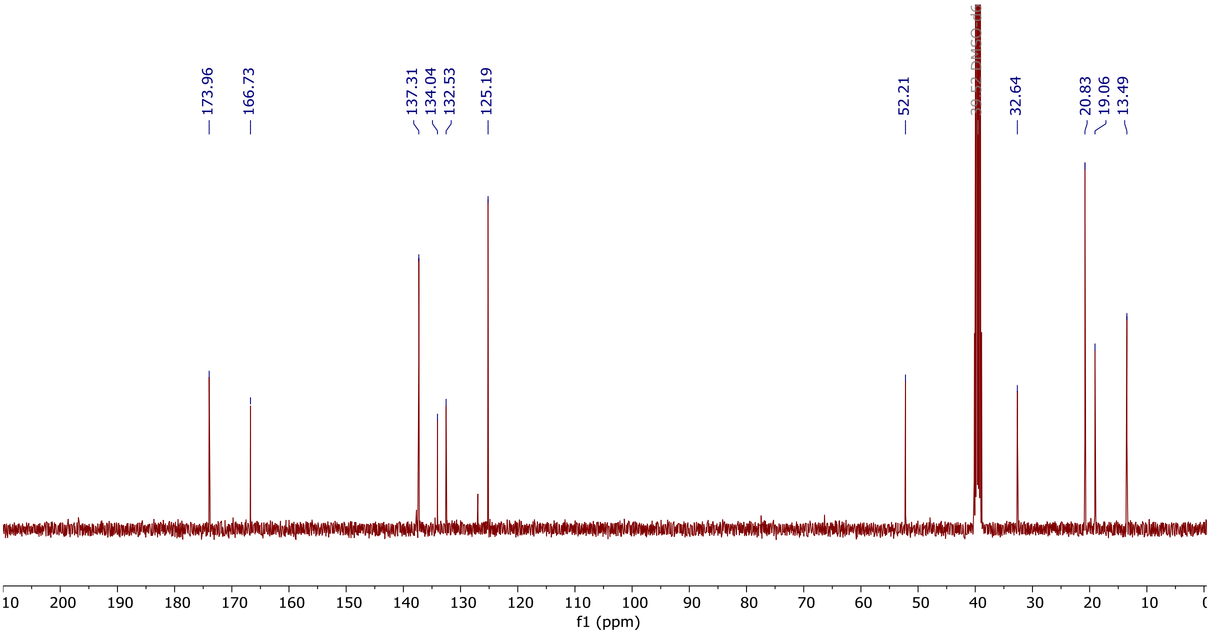

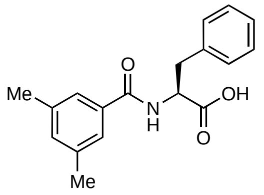

^{1}H NMR spectrum of **33**.

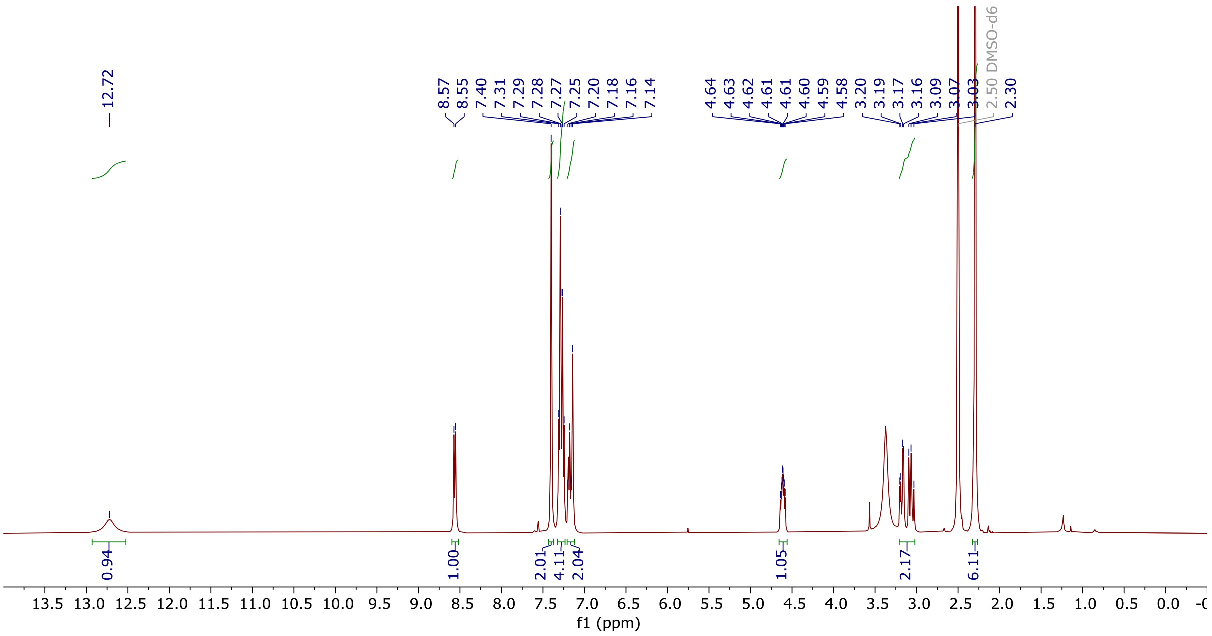

^{13}C NMR spectrum of **33**.

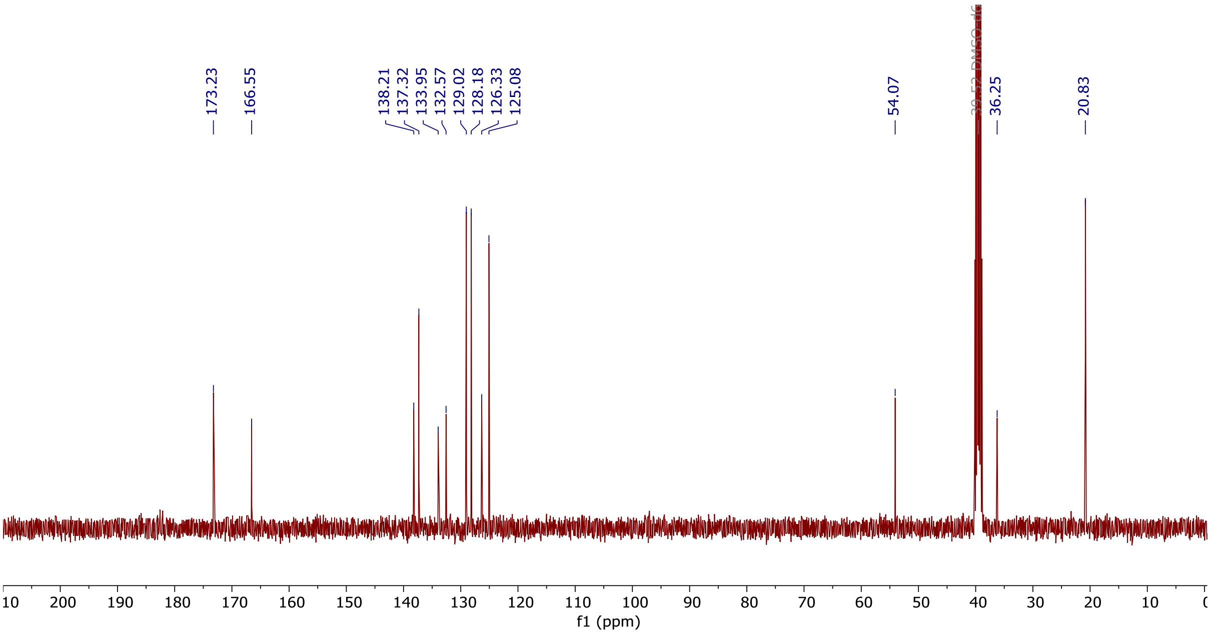

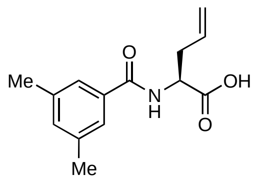

^{1}H NMR spectrum of **34**.

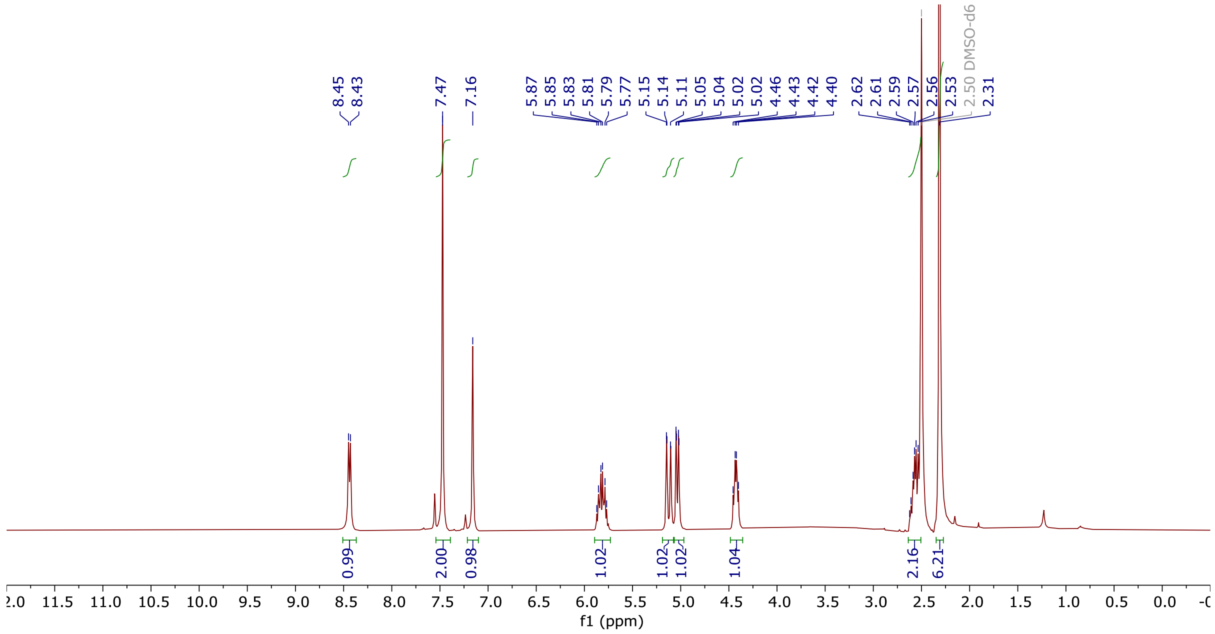

^{13}C NMR spectrum of **34**.

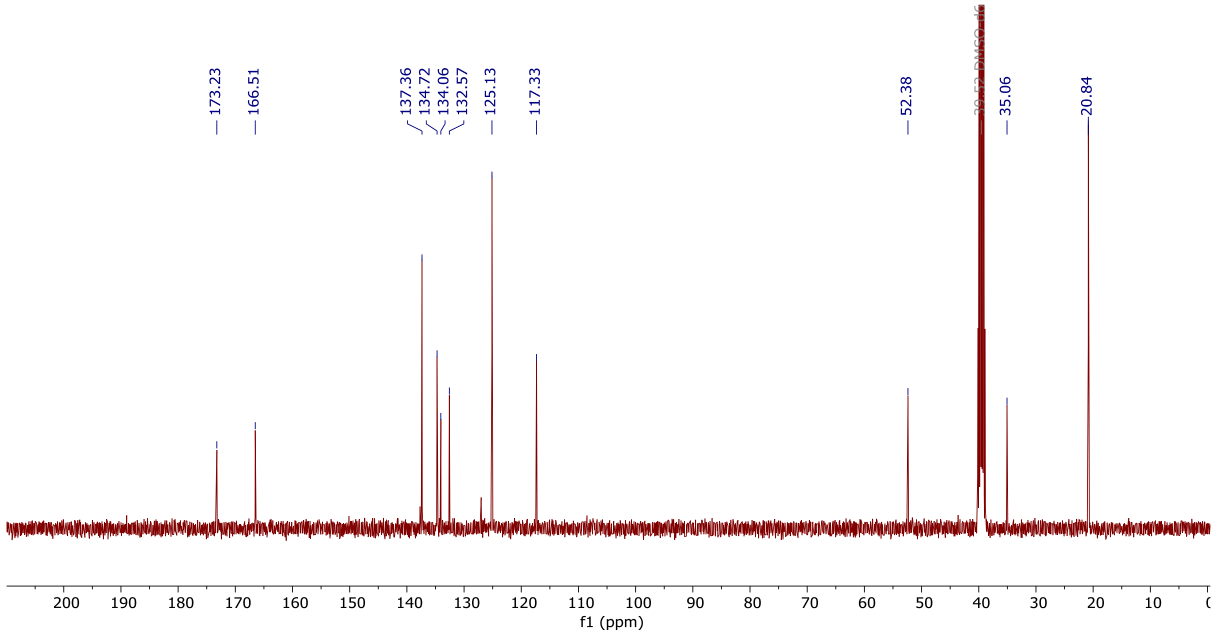

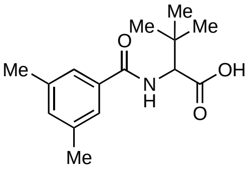

^{1}H NMR spectrum of **35**.

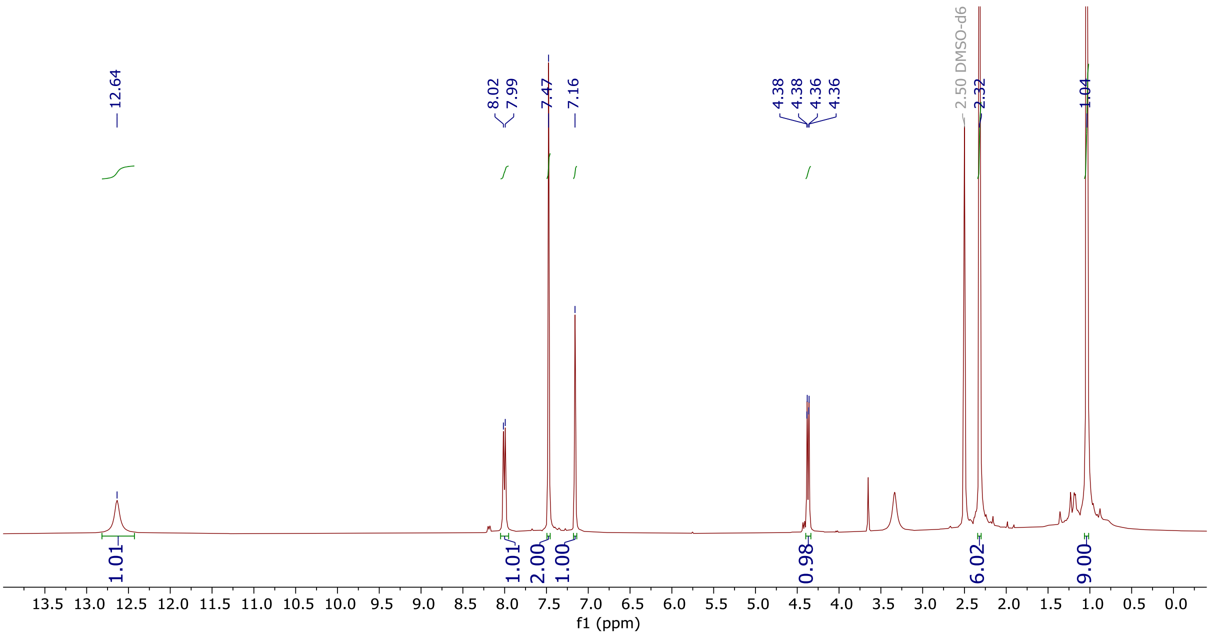

^{13}C NMR spectrum of **35**.

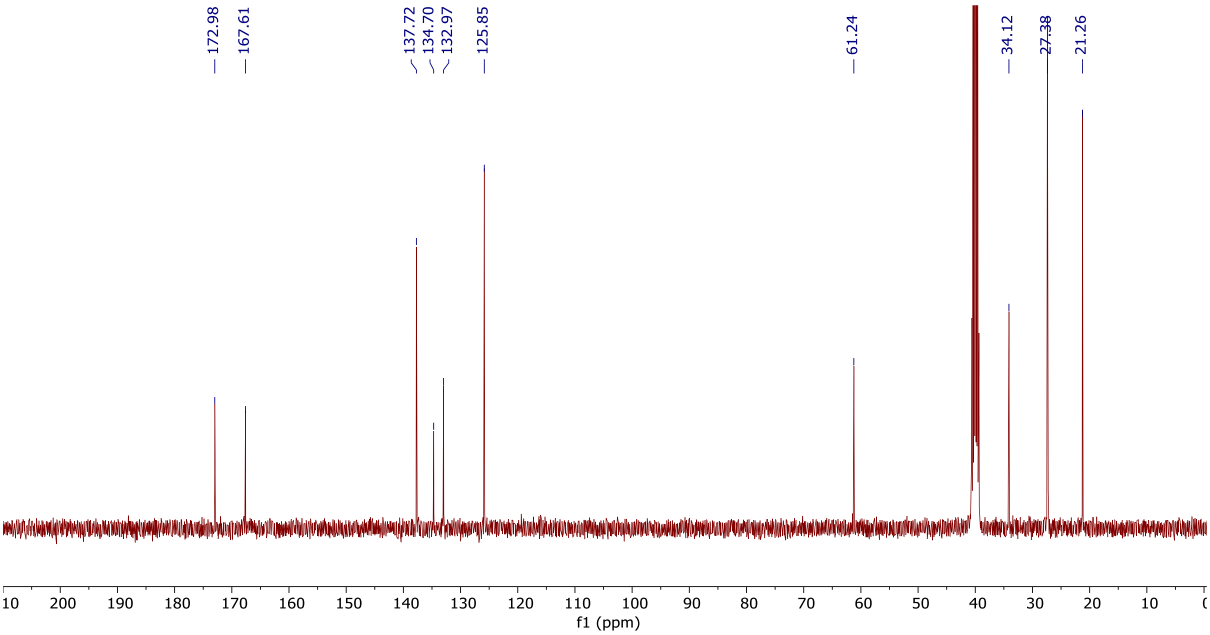

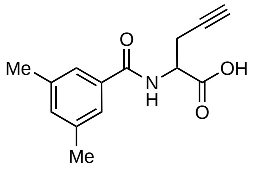

^{1}H NMR spectrum of **36**.

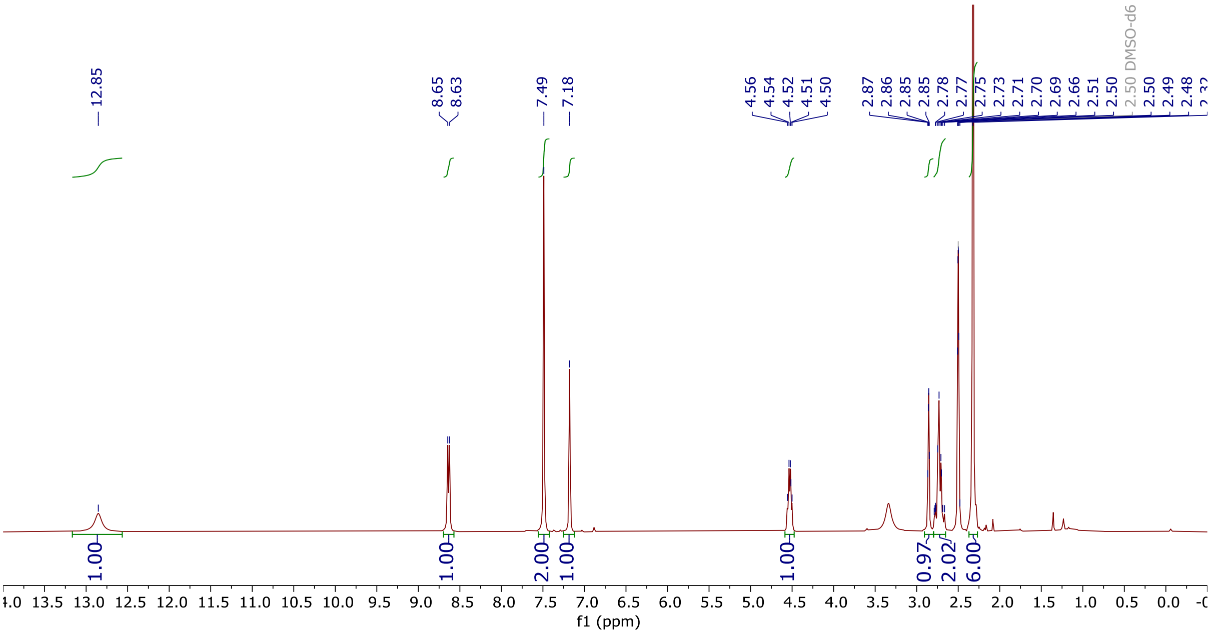

^{13}C NMR spectrum of **36**.

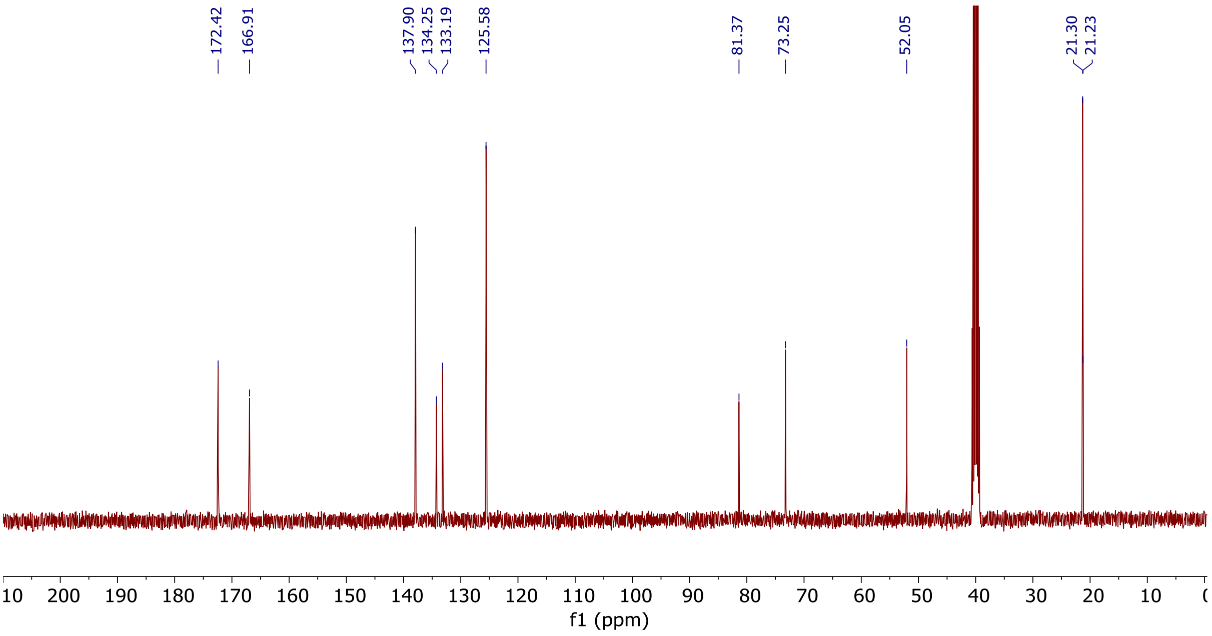

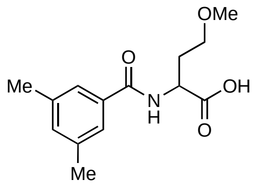

^{1}H NMR spectrum of **37**.

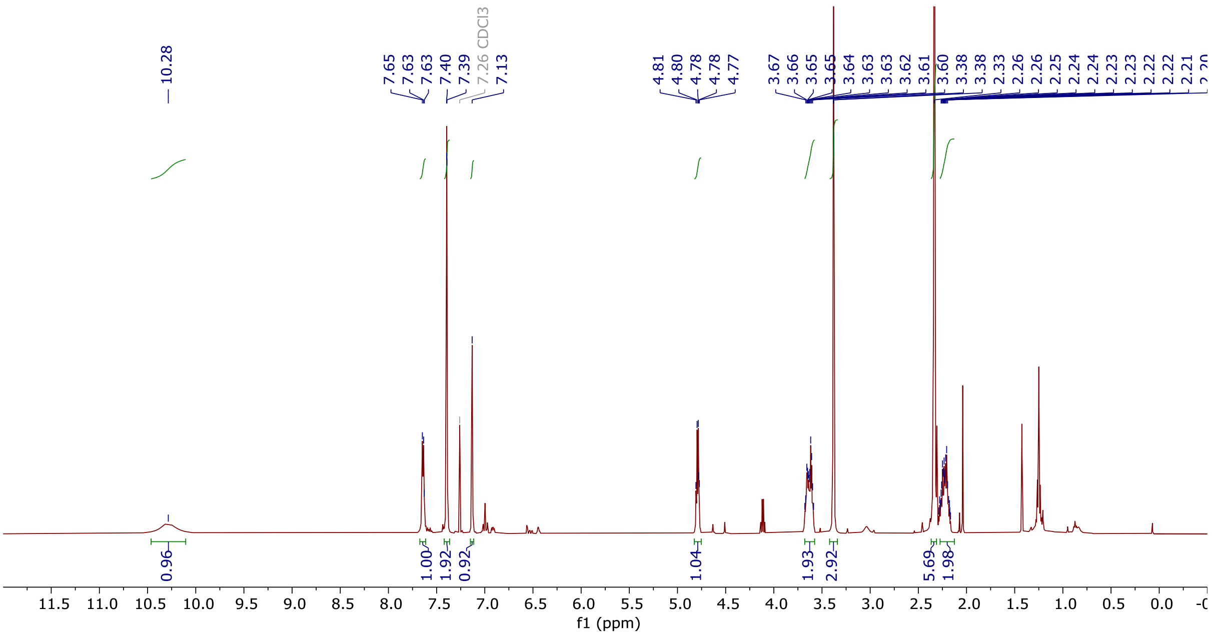

^{13}C NMR spectrum of **37**.

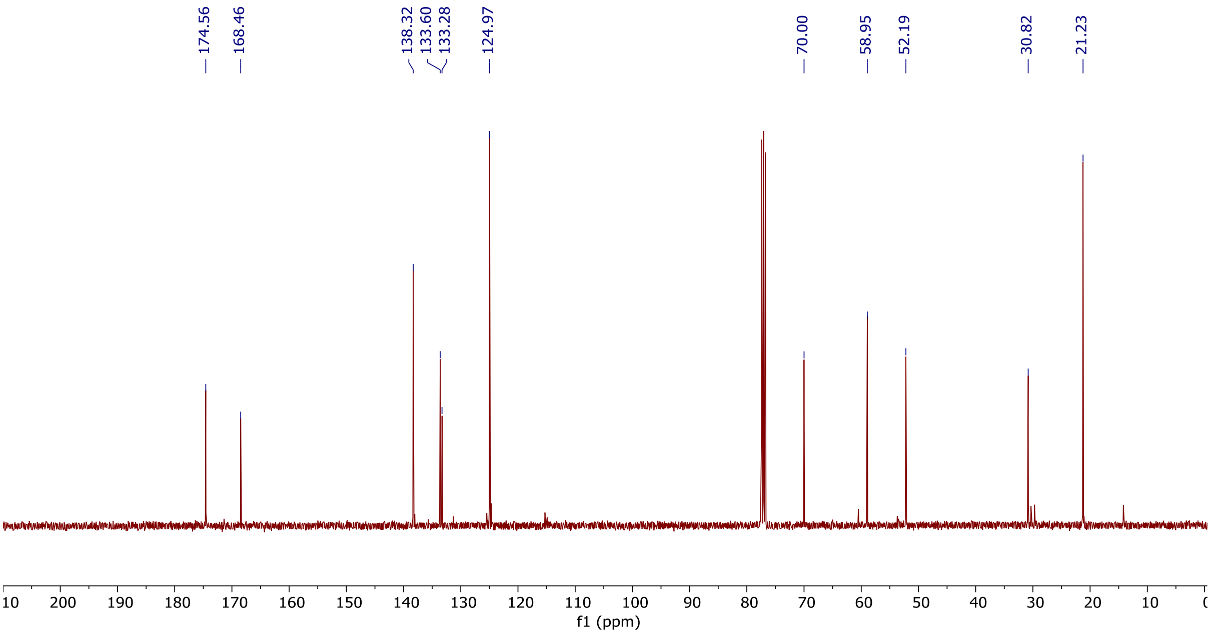

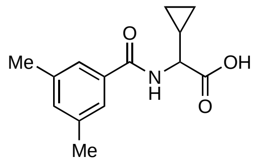

^{1}H NMR spectrum of **38**.

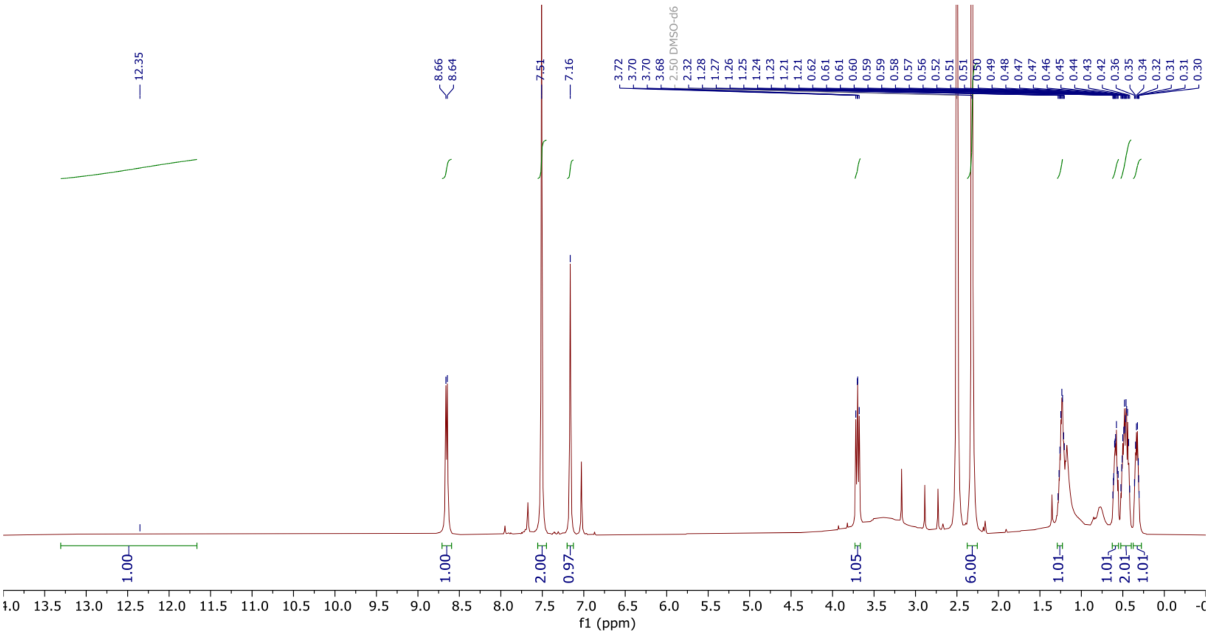

^{13}C NMR spectrum of **38**.

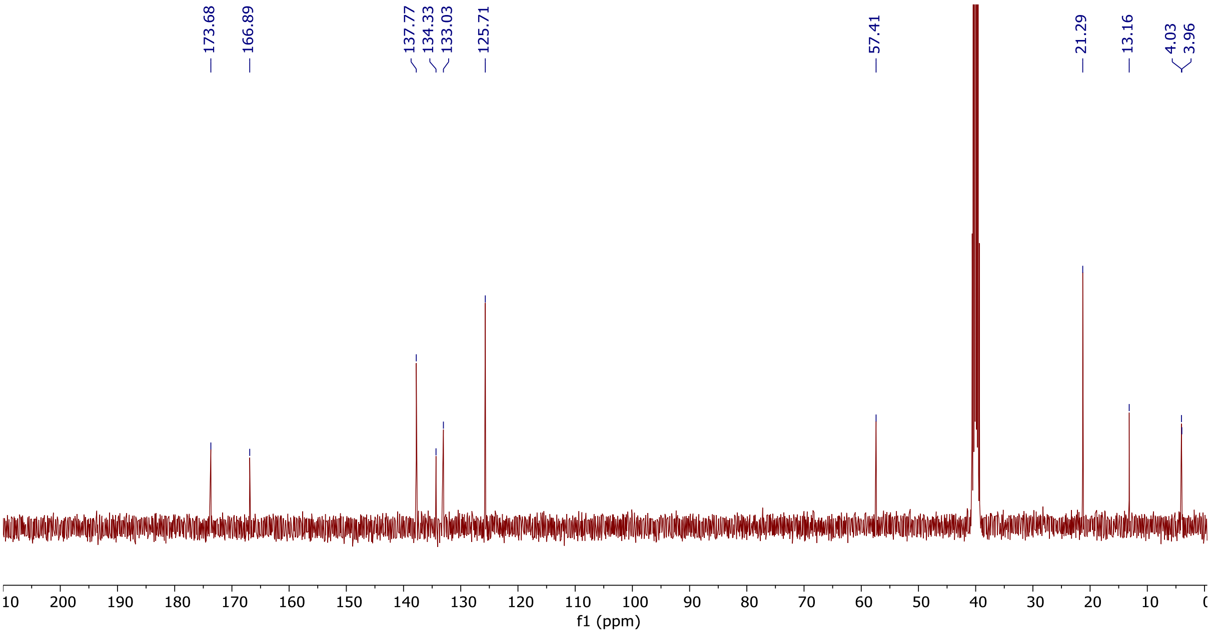

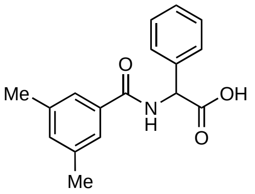

^{1}H NMR spectrum of **39**.

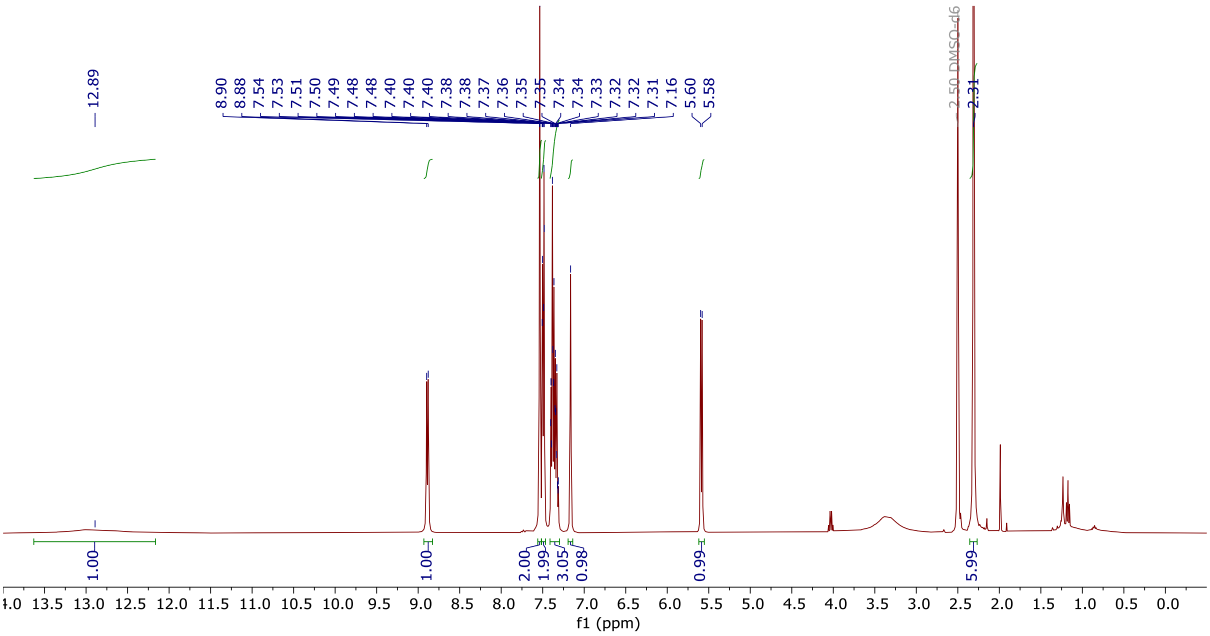

^{13}C NMR spectrum of **39**.

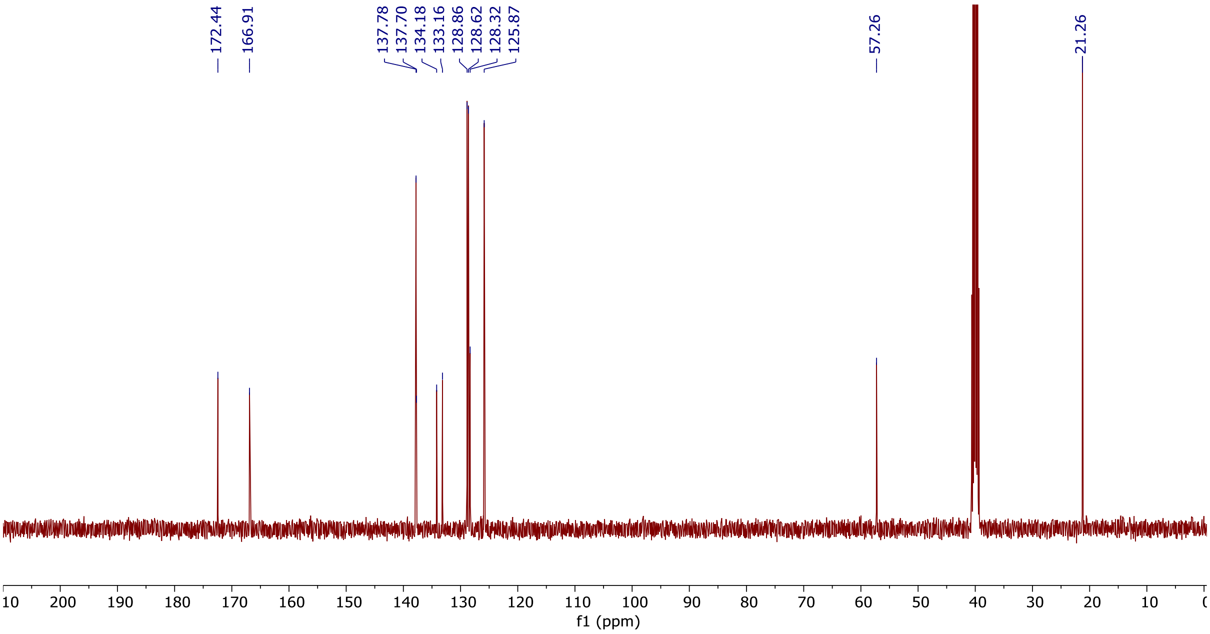

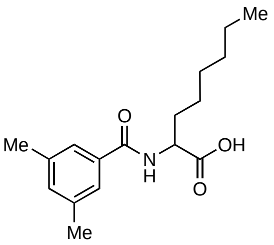

^{1}H NMR spectrum of **40**.

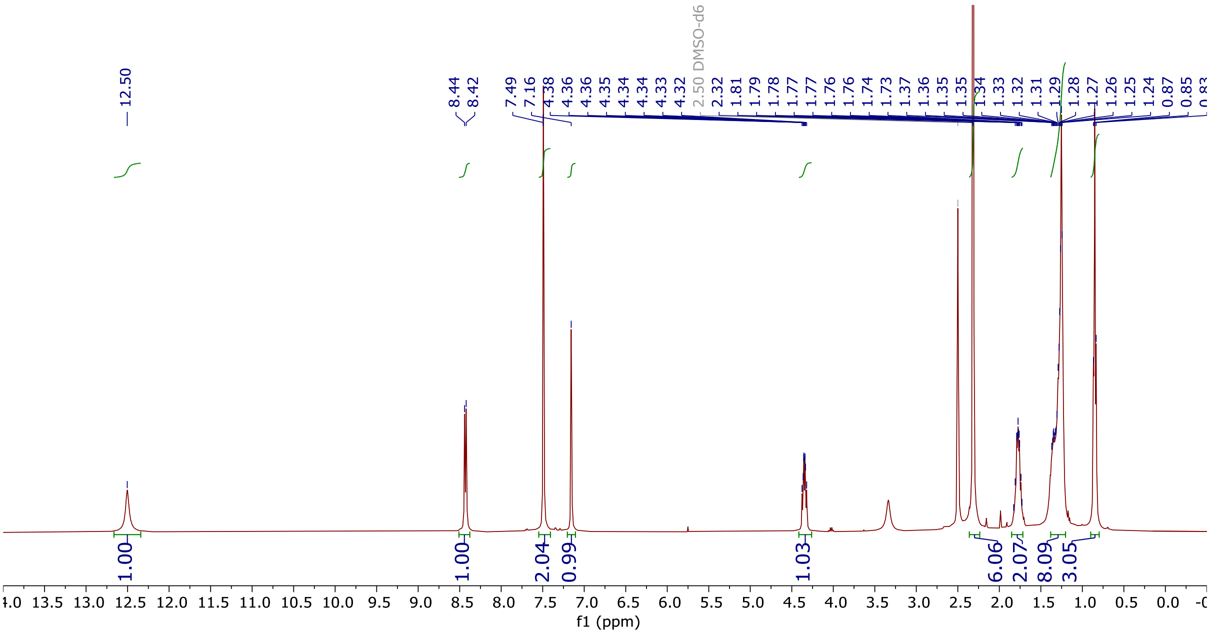

^{13}C NMR spectrum of **40**.

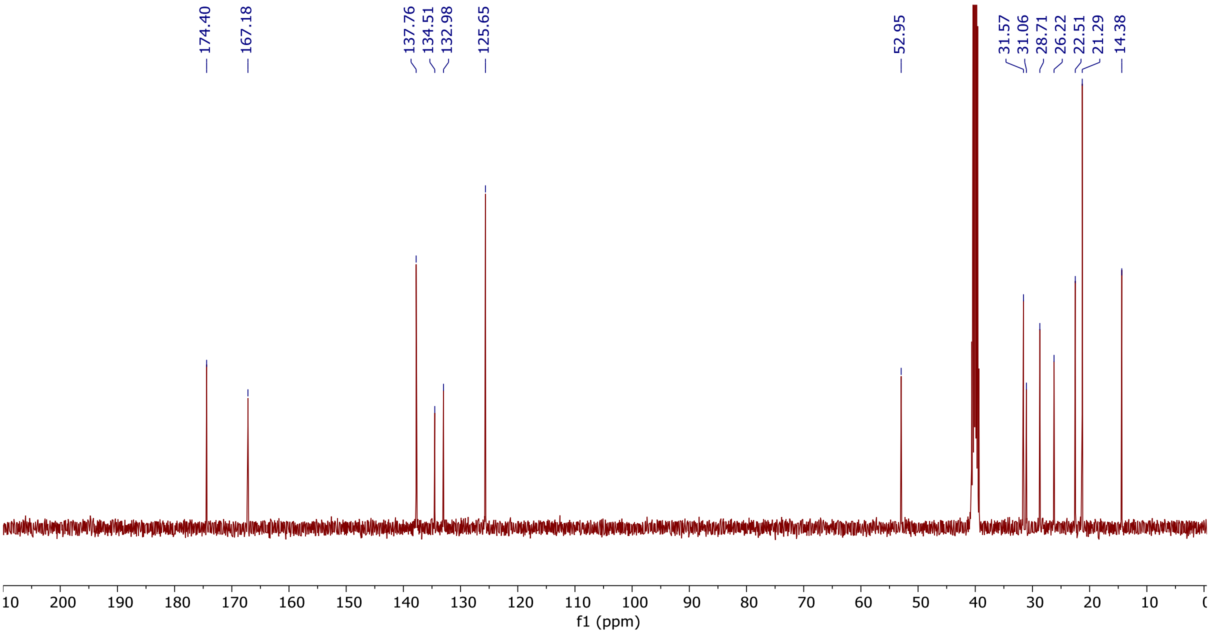

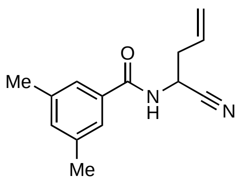

^{1}H NMR spectrum of **41**.

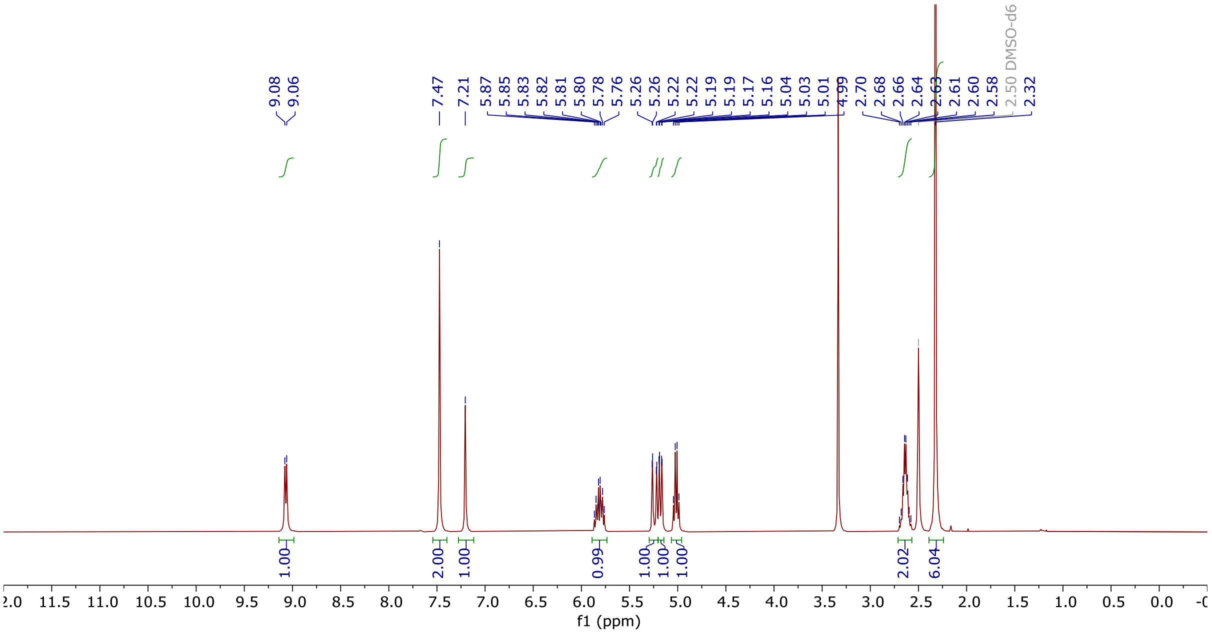

^{13}C NMR spectrum of **41**.

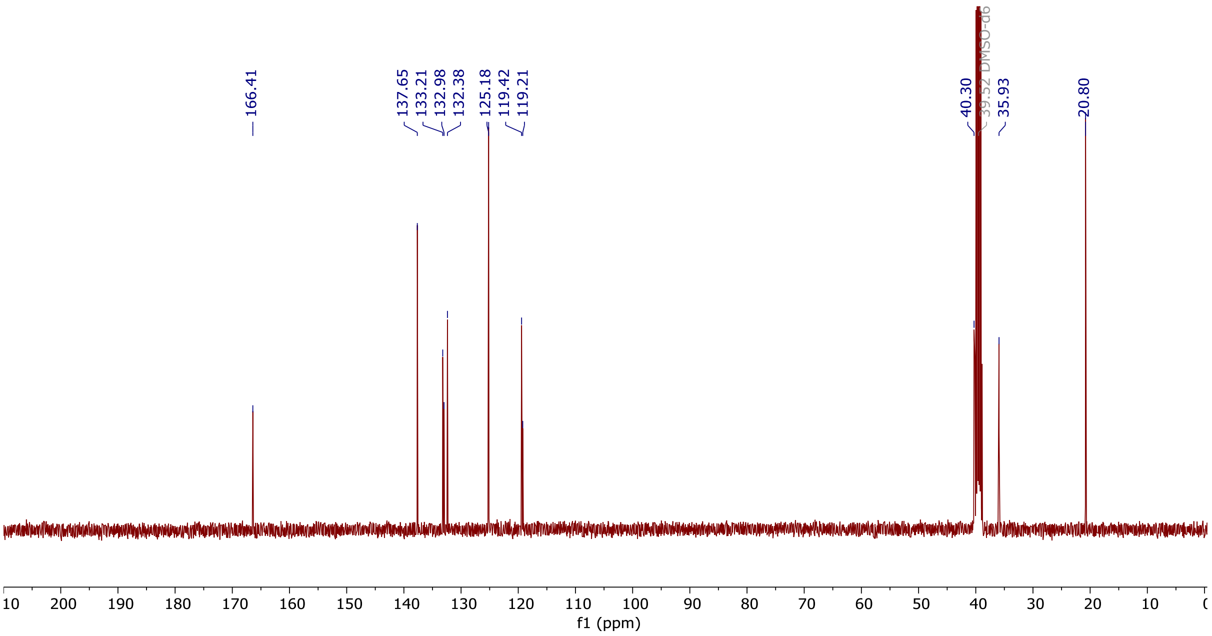

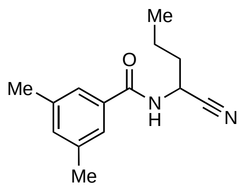

^{1}H NMR spectrum of **42**.

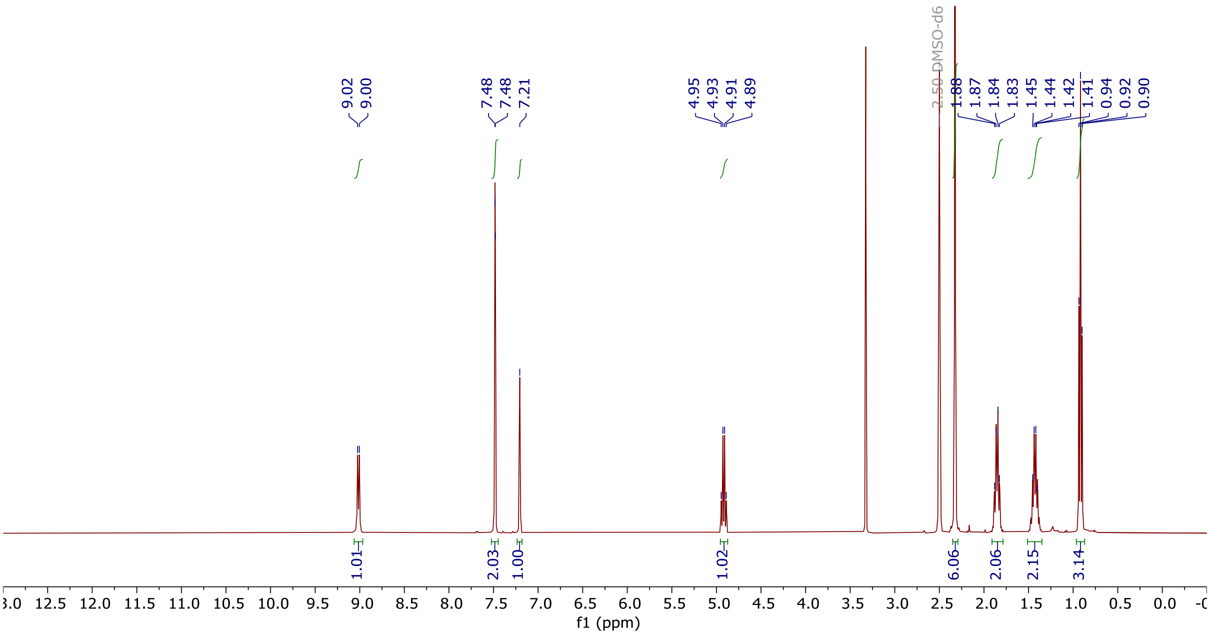

^{13}C NMR spectrum of **42**.

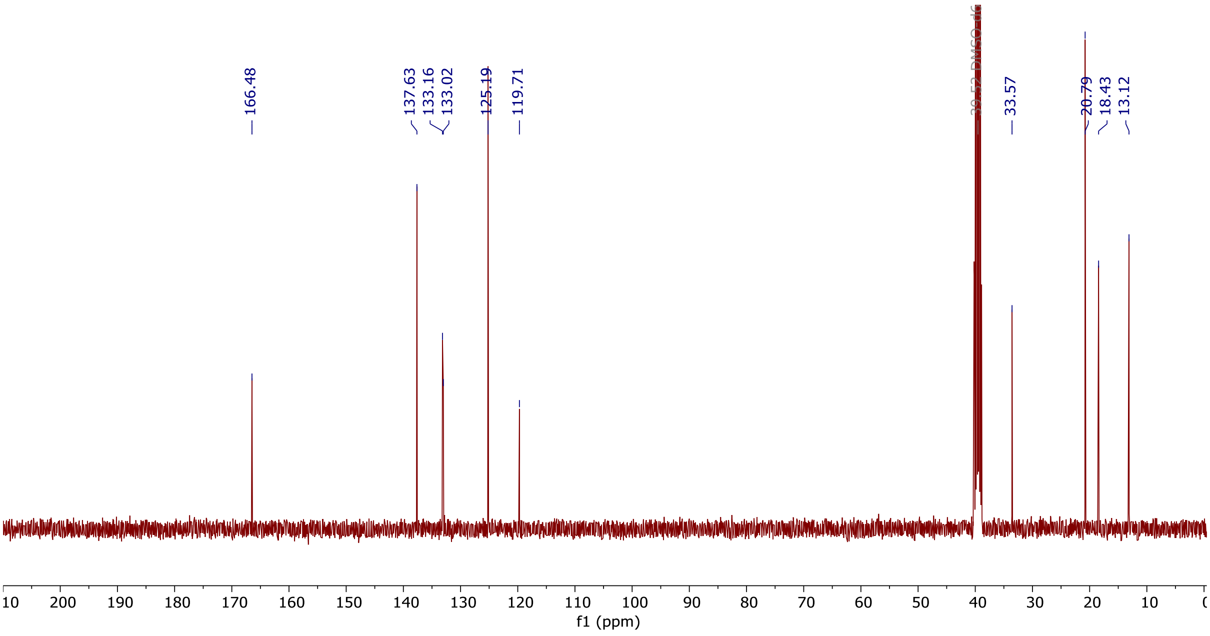

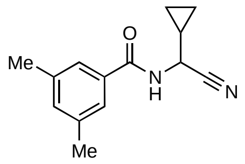

^{1}H NMR spectrum of **43**.

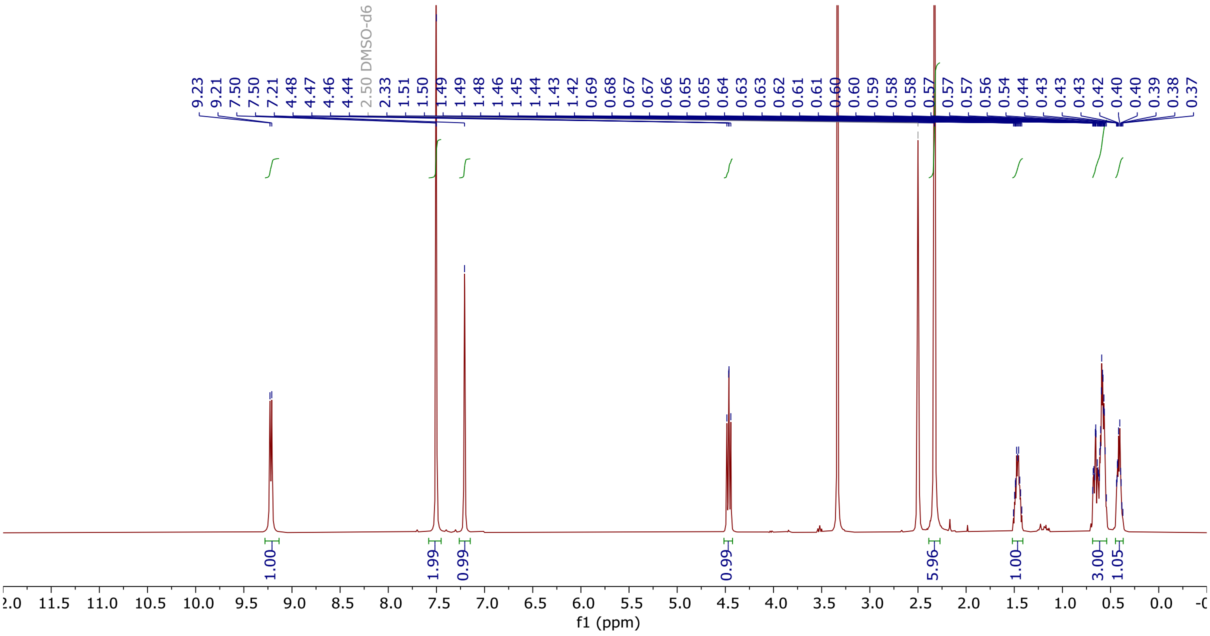

^{13}C NMR spectrum of **43**.

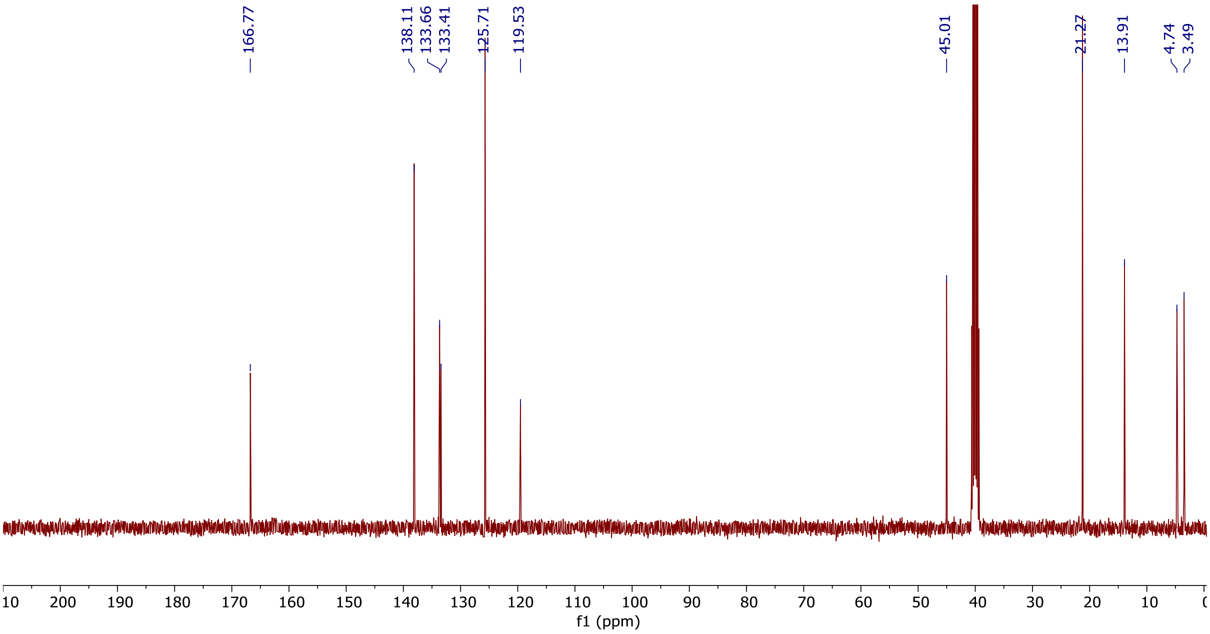

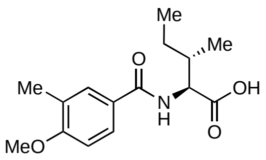

^{1}H NMR spectrum of **44**.

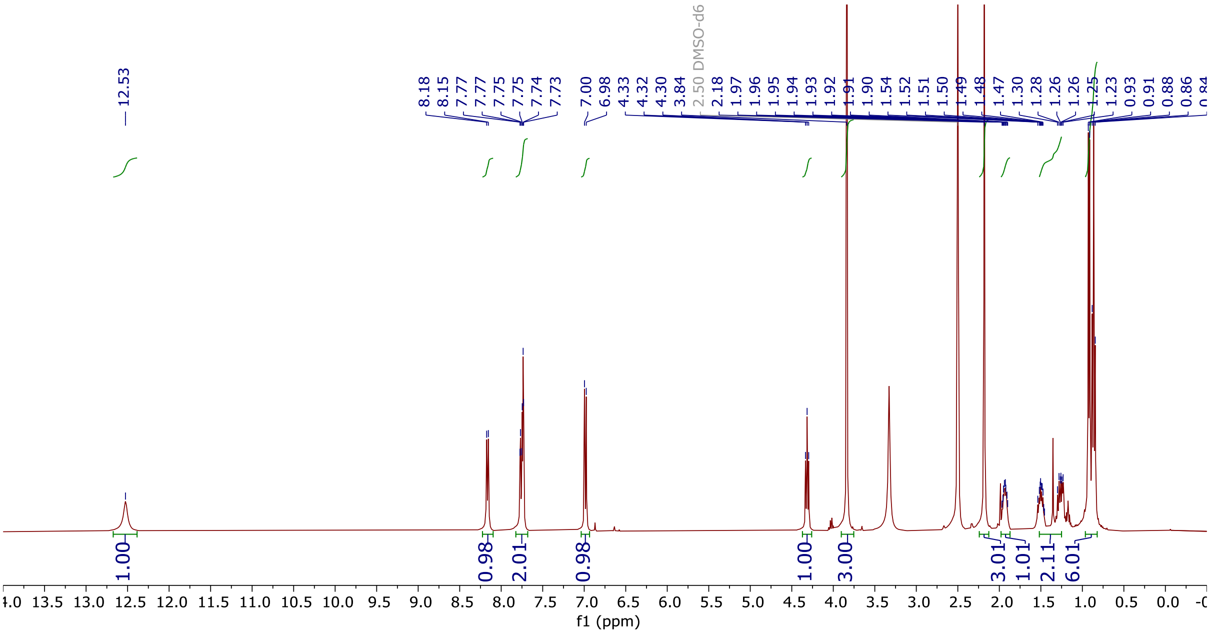

^{13}C NMR spectrum of **44**.

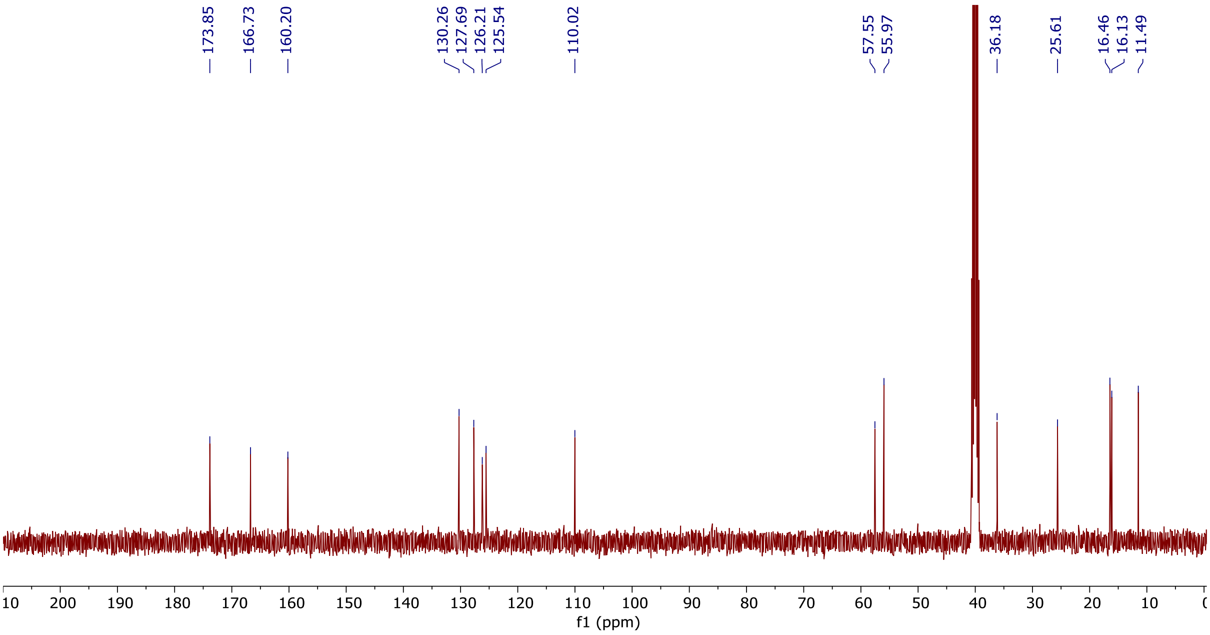

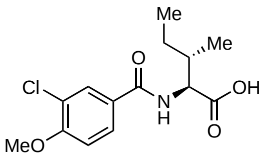

^{1}H NMR spectrum of **45**.

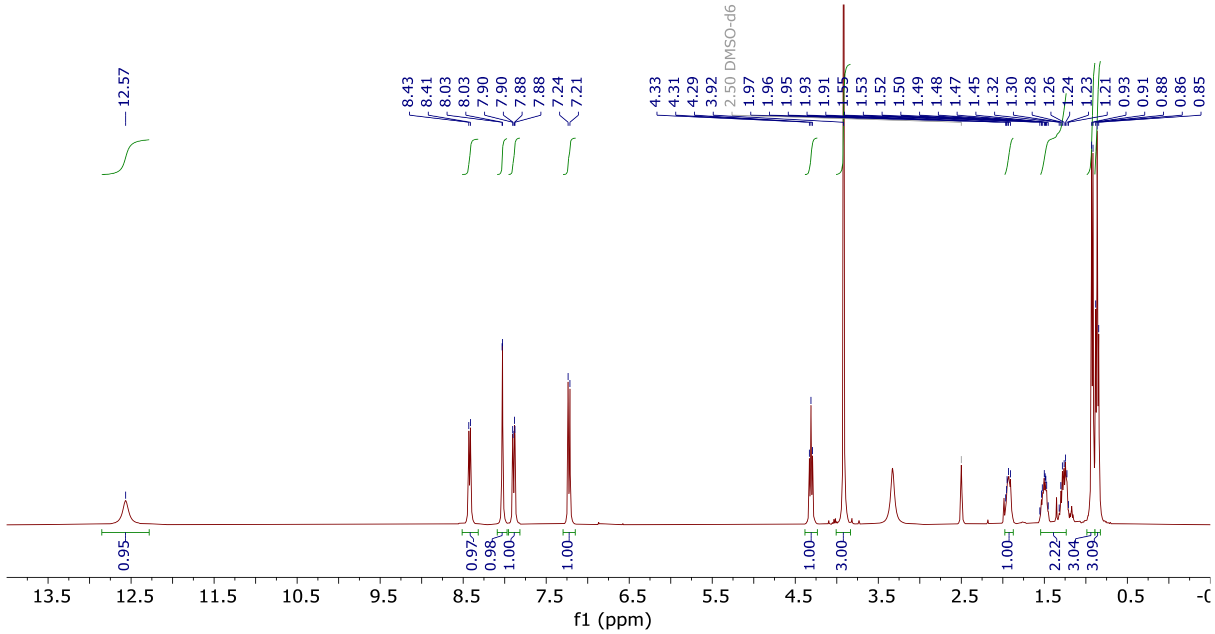

^{13}C NMR spectrum of **45**.

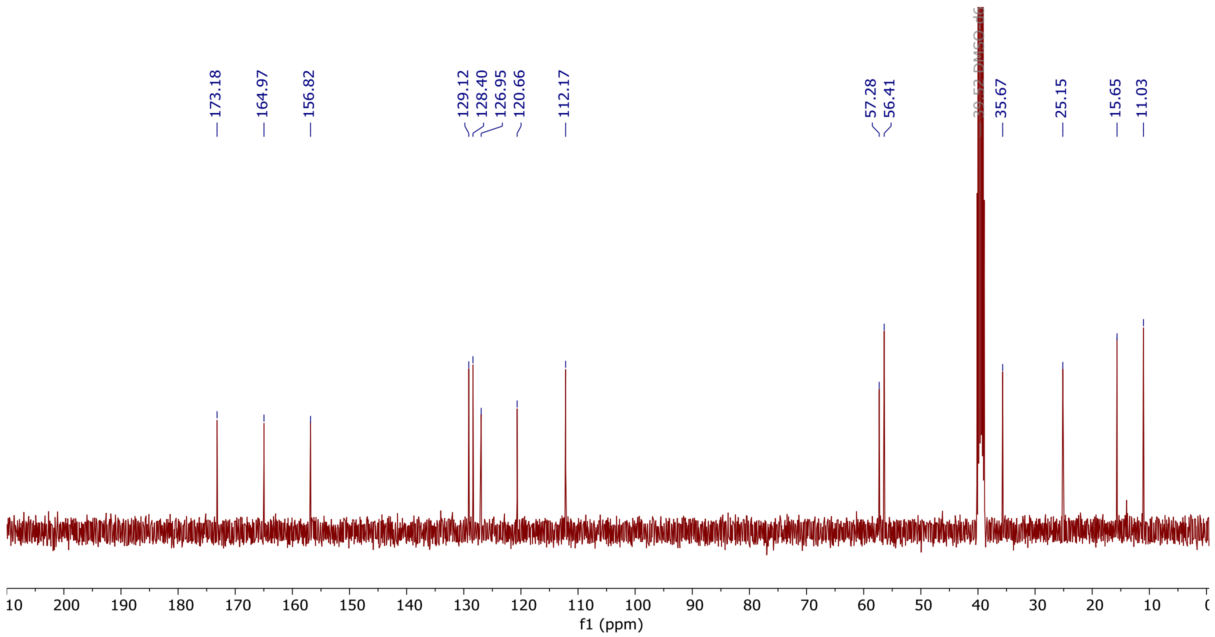

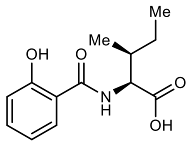

^{1}H NMR spectrum of **46**.

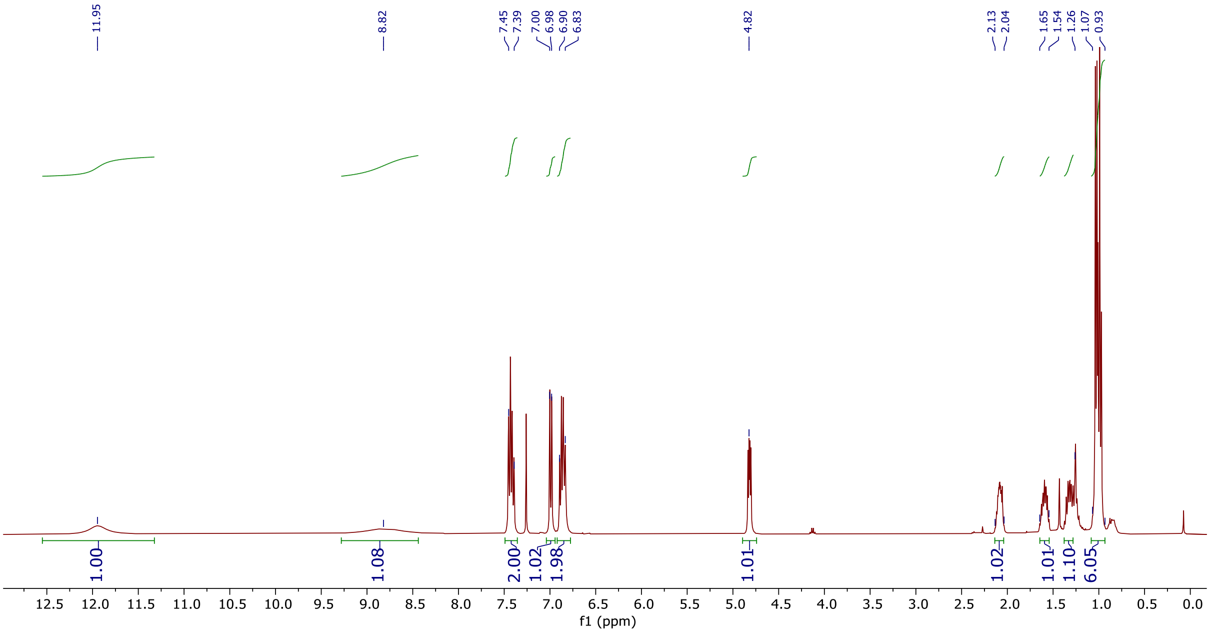

^{13}C NMR spectrum of **46**.

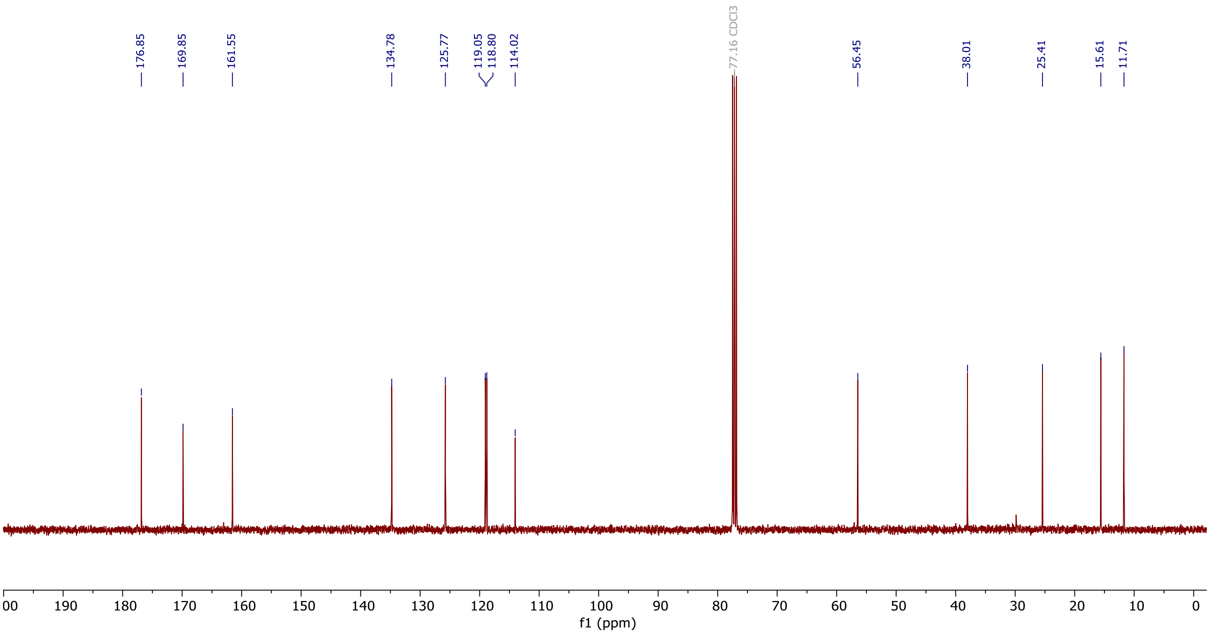

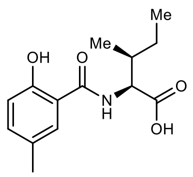

^{1}H NMR spectrum of **47**.

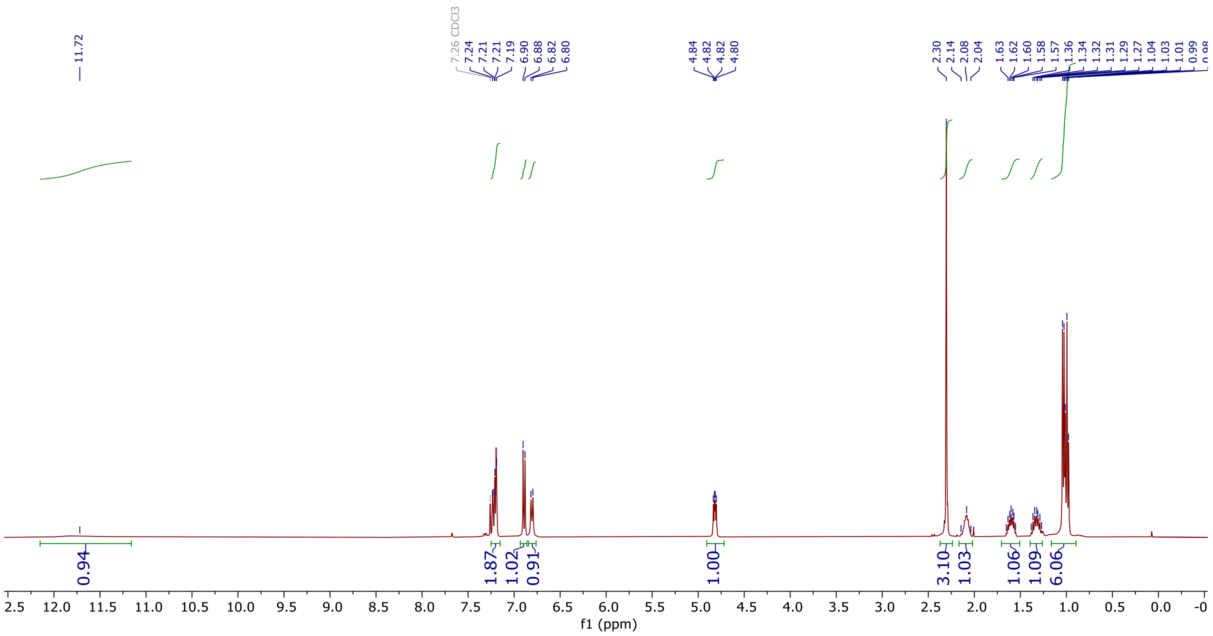

^{13}C NMR spectrum of **47**.

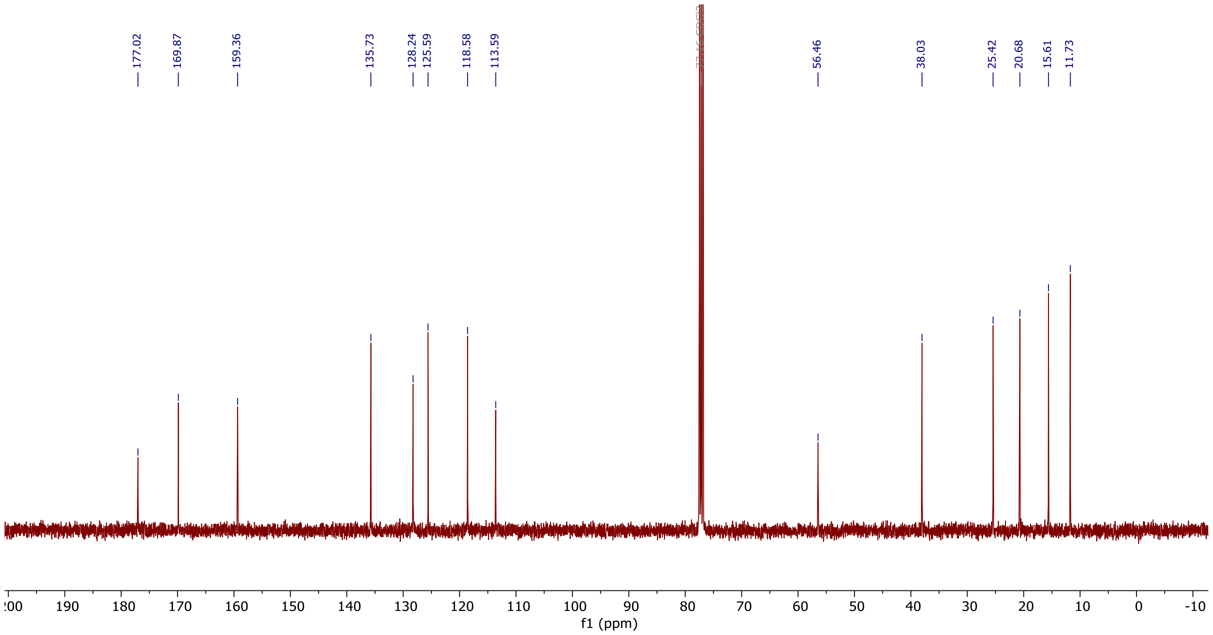

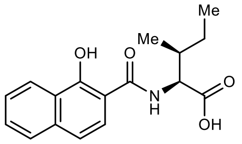

^{1}H NMR spectrum of **48**.

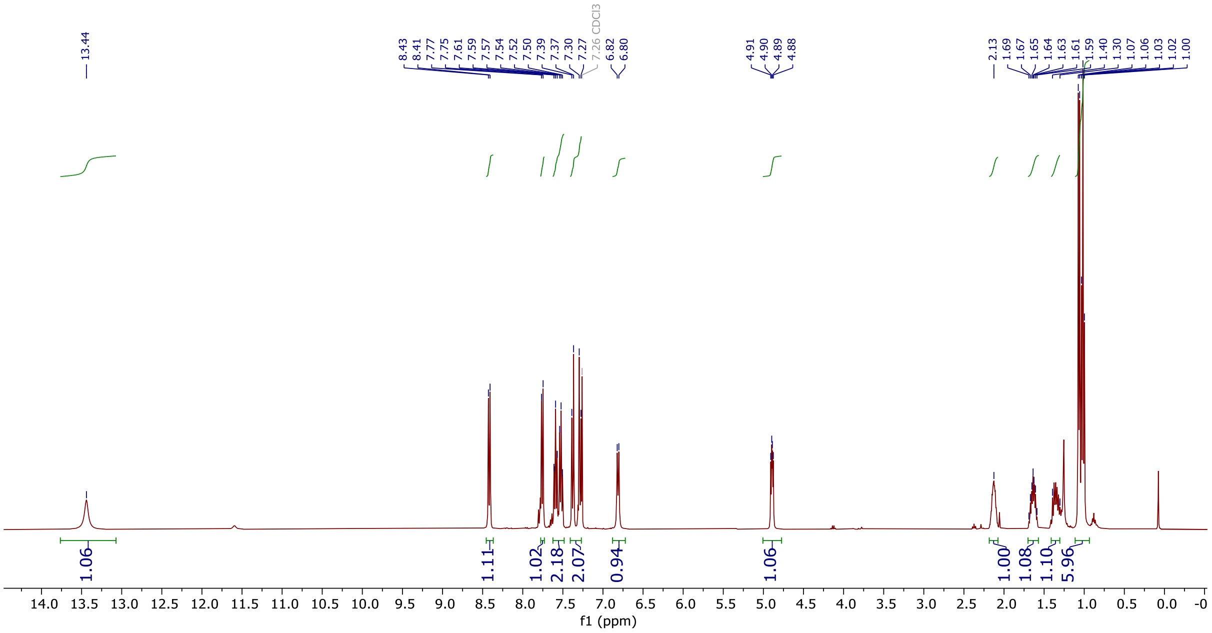

^{13}C NMR spectrum of **48**.

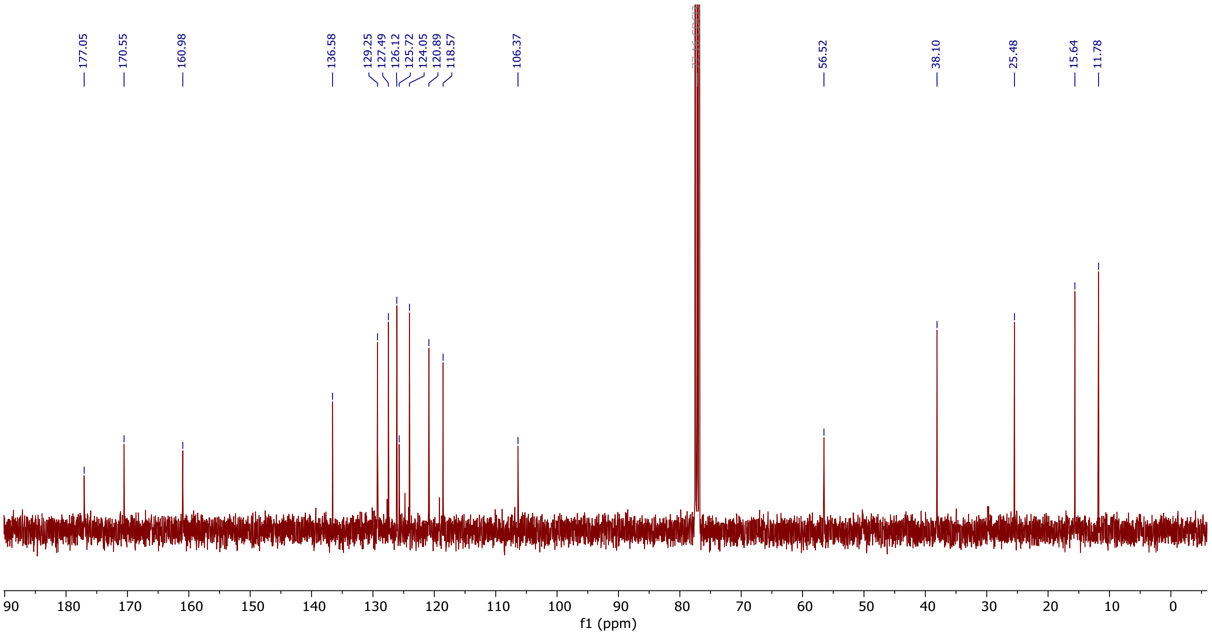

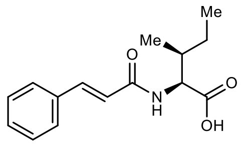

^{1}H NMR spectrum of **49**.

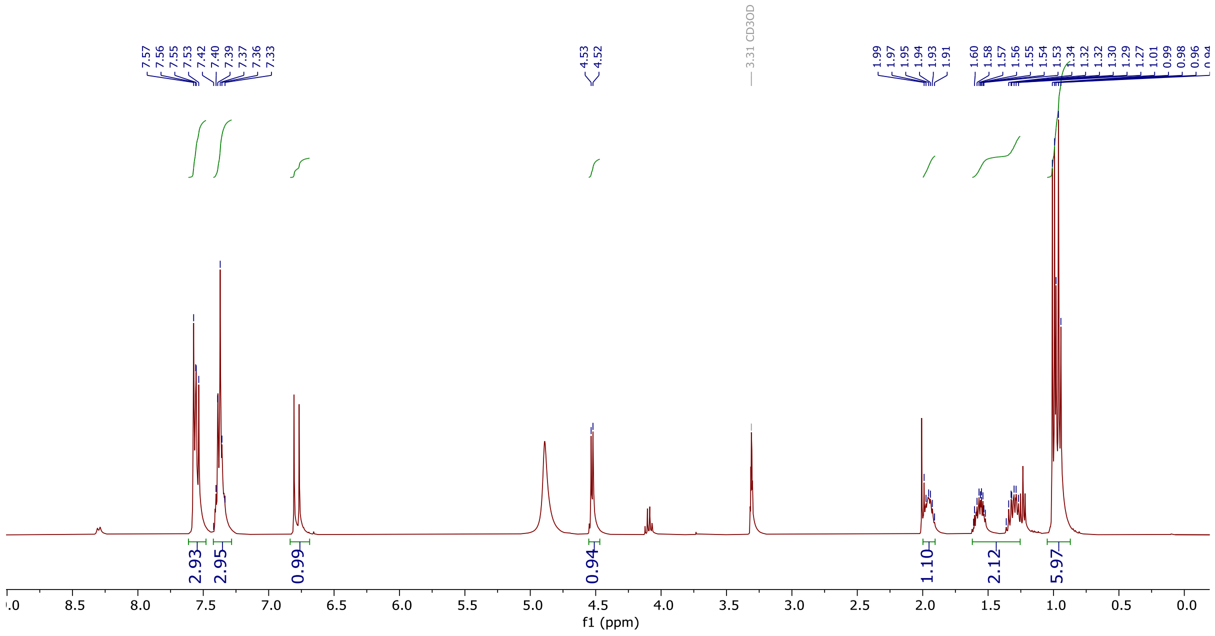

^{13}C NMR spectrum of **49**.

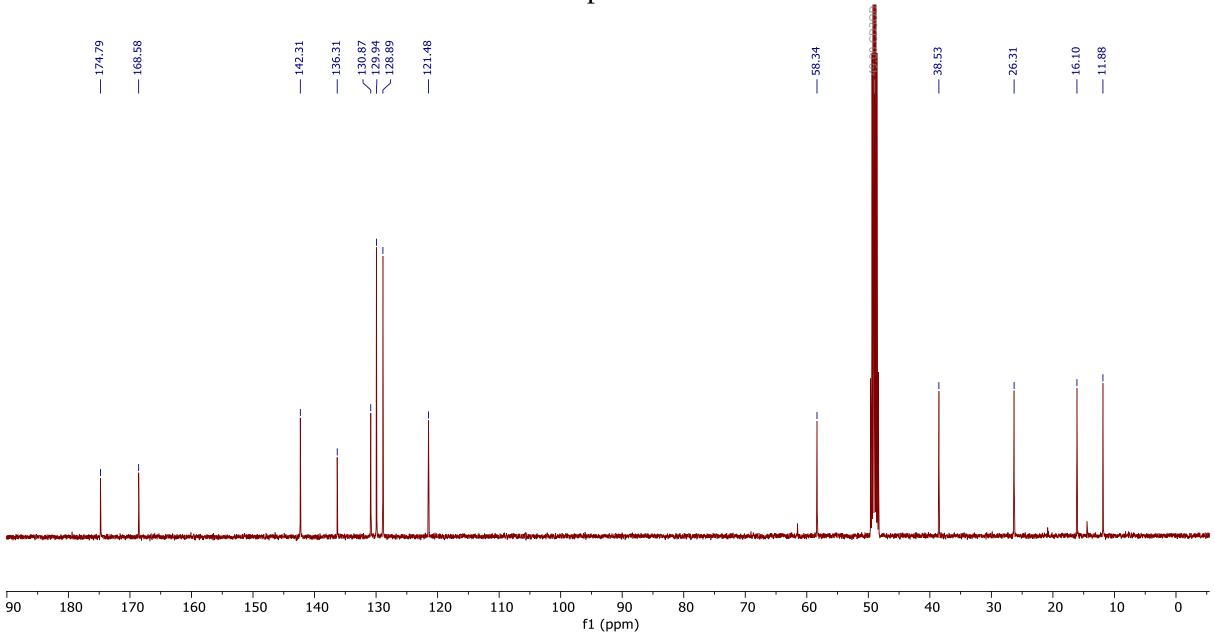

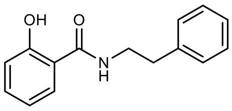

^{1}H NMR spectrum of **50**.

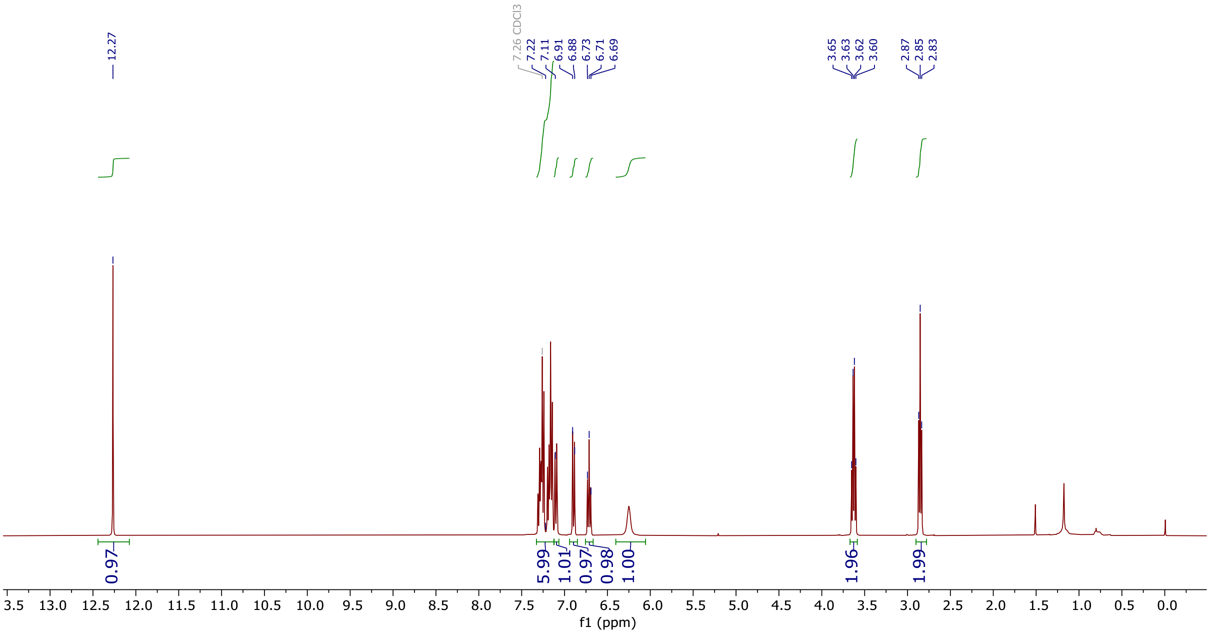

^{13}C NMR spectrum of **50**.

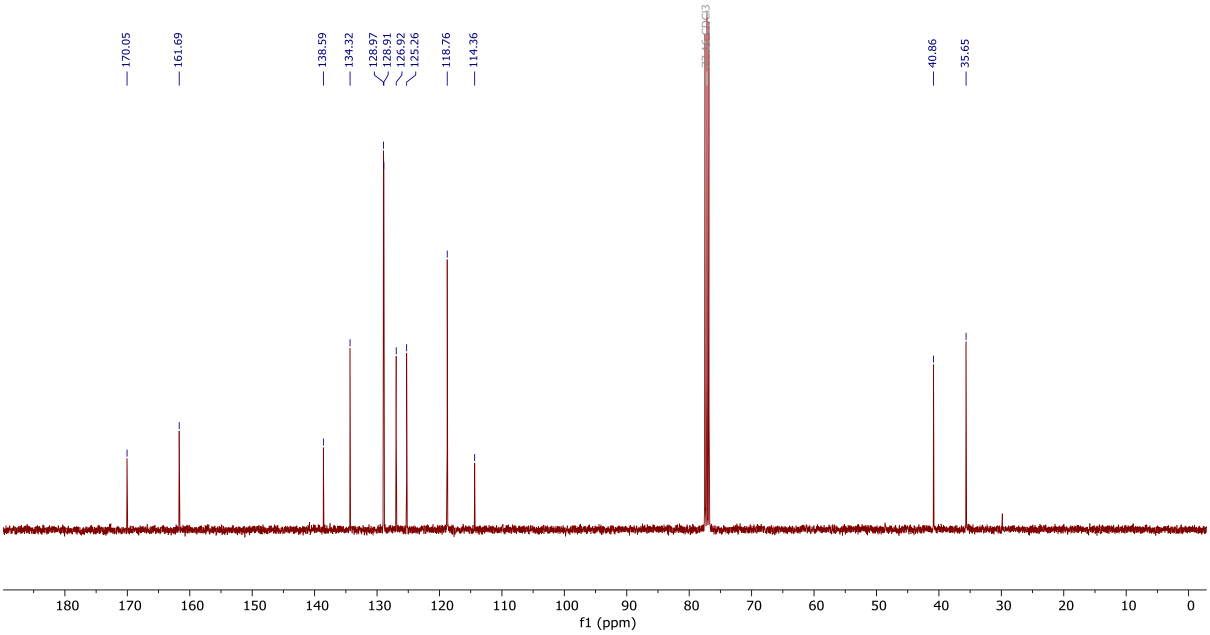

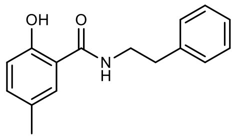

^{1}H NMR spectrum of **51**.

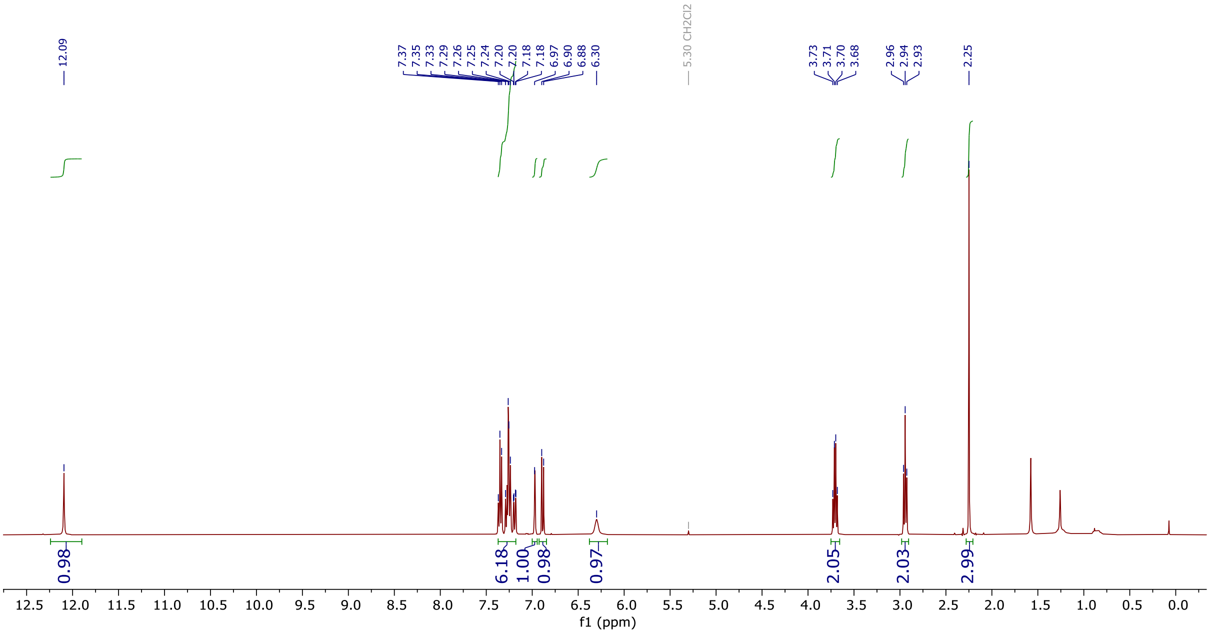

^{13}C NMR spectrum of **51**.

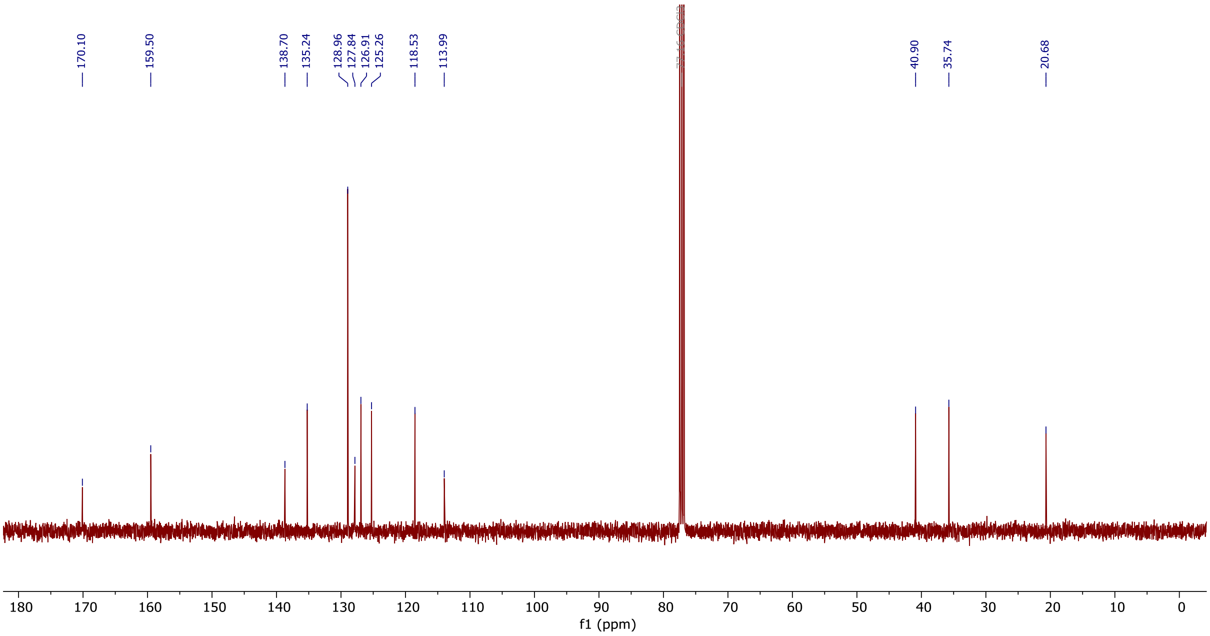

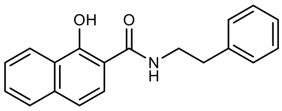

^{1}H NMR spectrum of **52**.

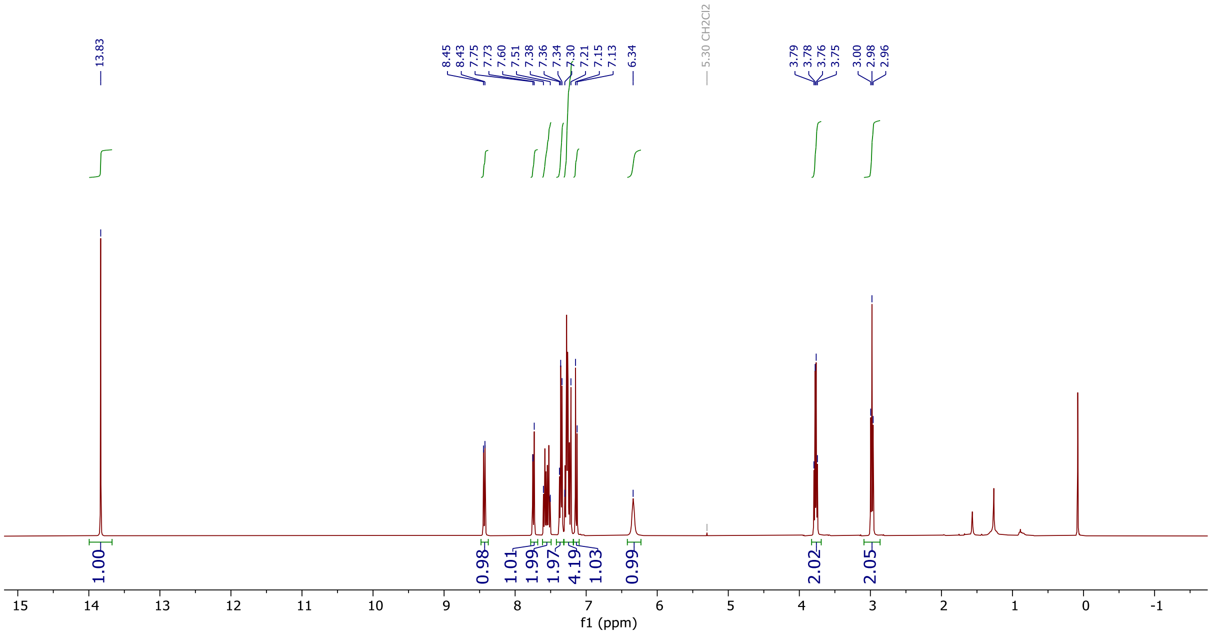

^{13}C NMR spectrum of **52**.

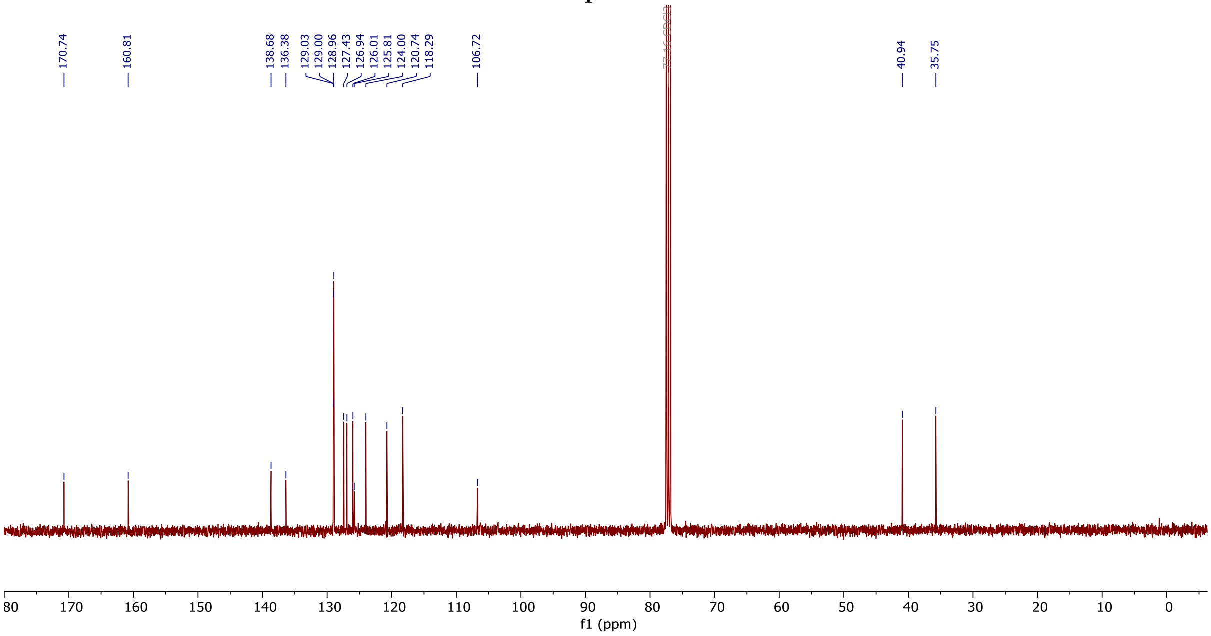

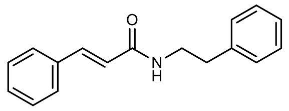

^{1}H NMR spectrum of **53**.

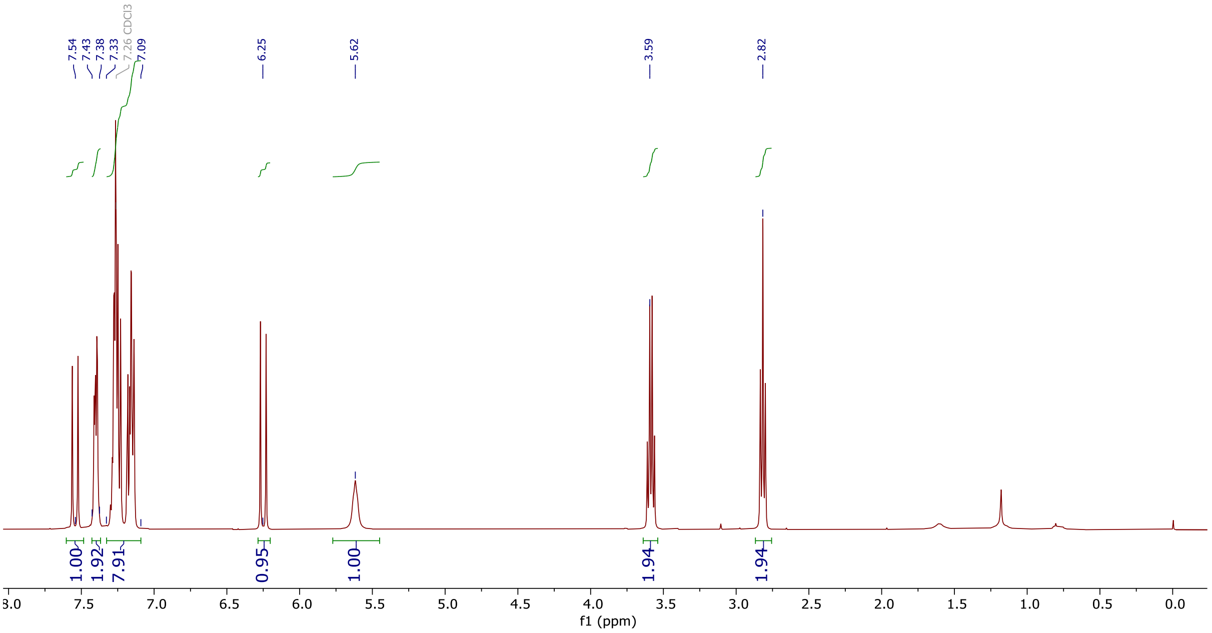

^{13}C NMR spectrum of **53**.

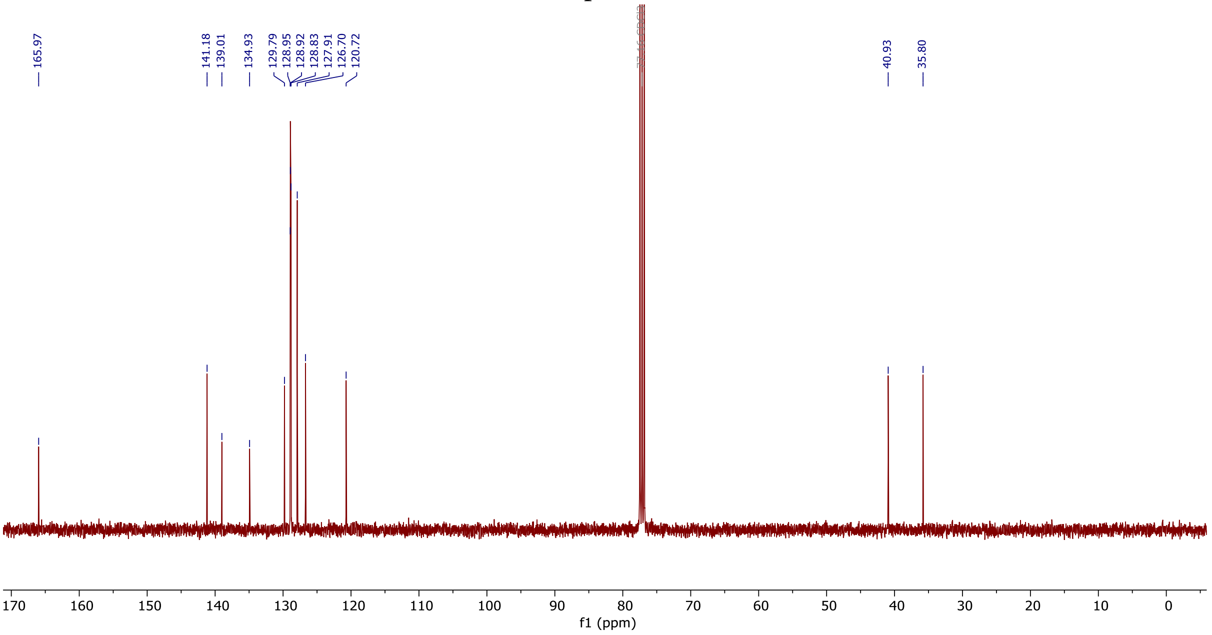

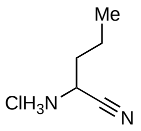

^{1}H NMR spectrum of **54**.

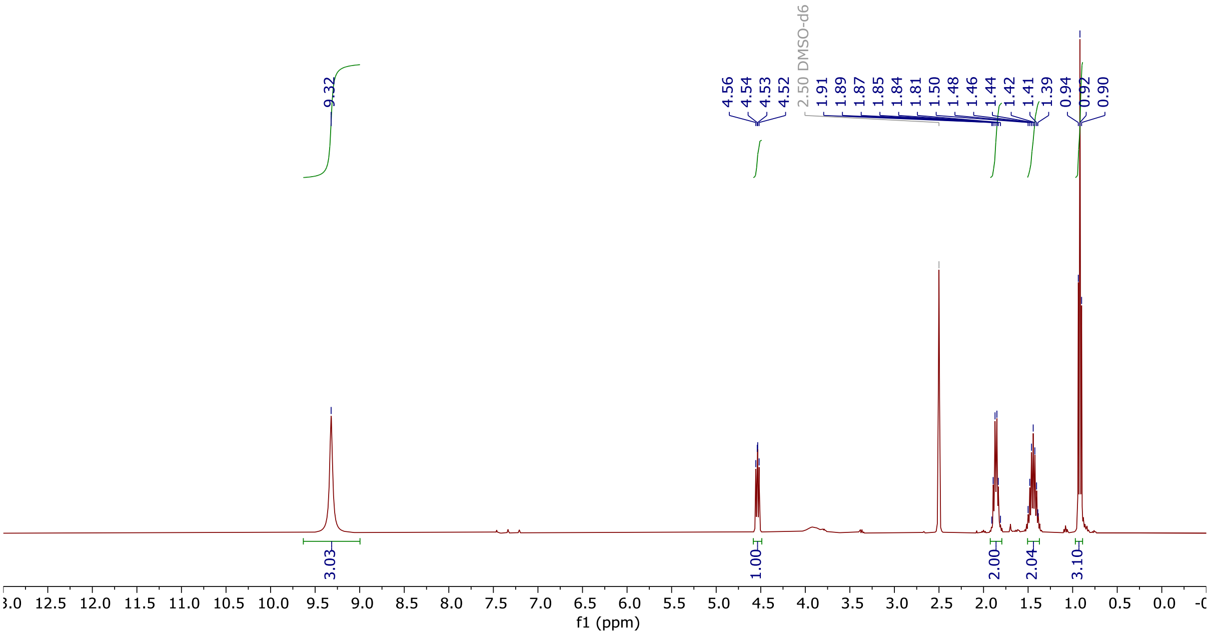

^{13}C NMR spectrum of **54**.

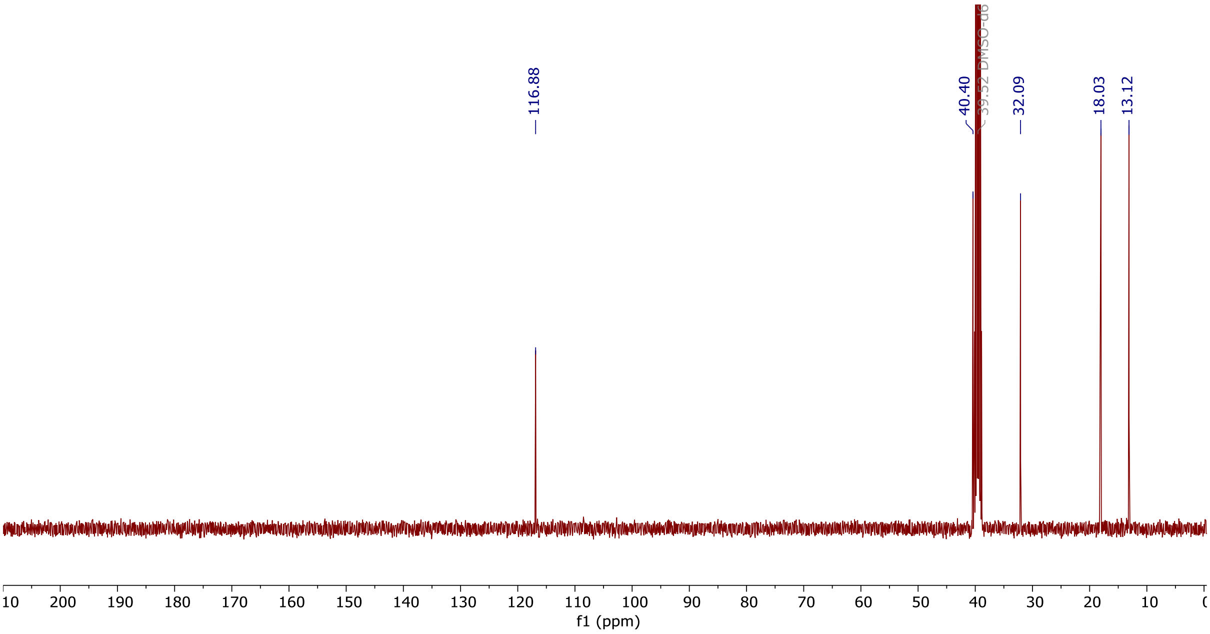

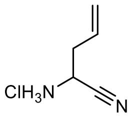

^{1}H NMR spectrum of **56**.

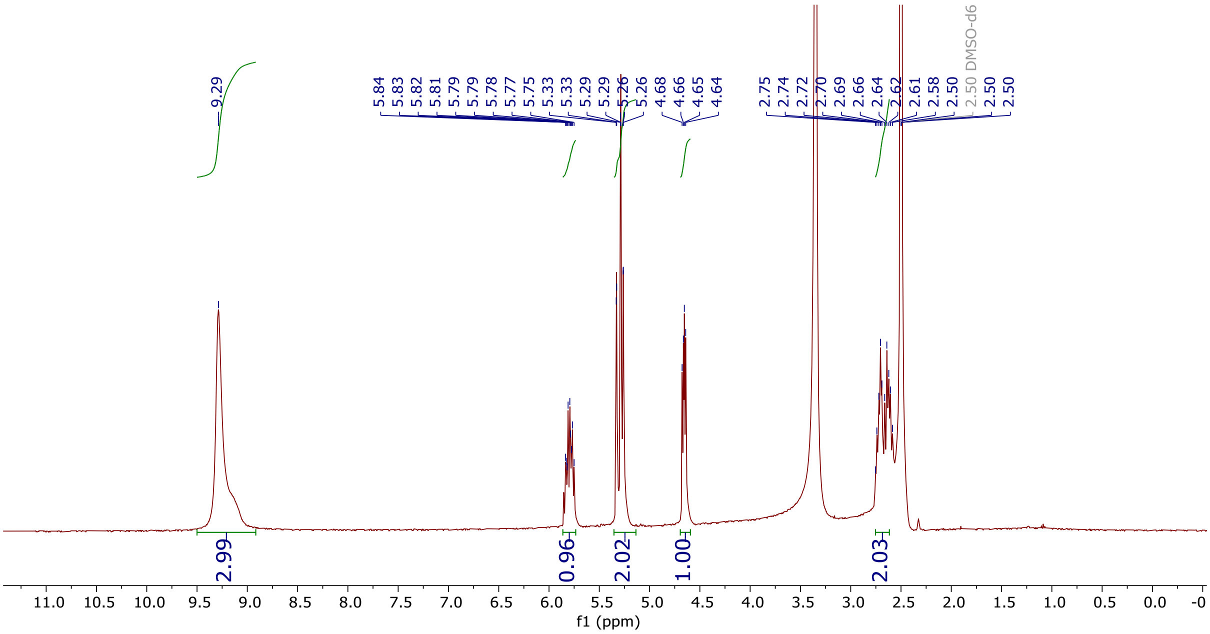

^{13}C NMR spectrum of **56**.

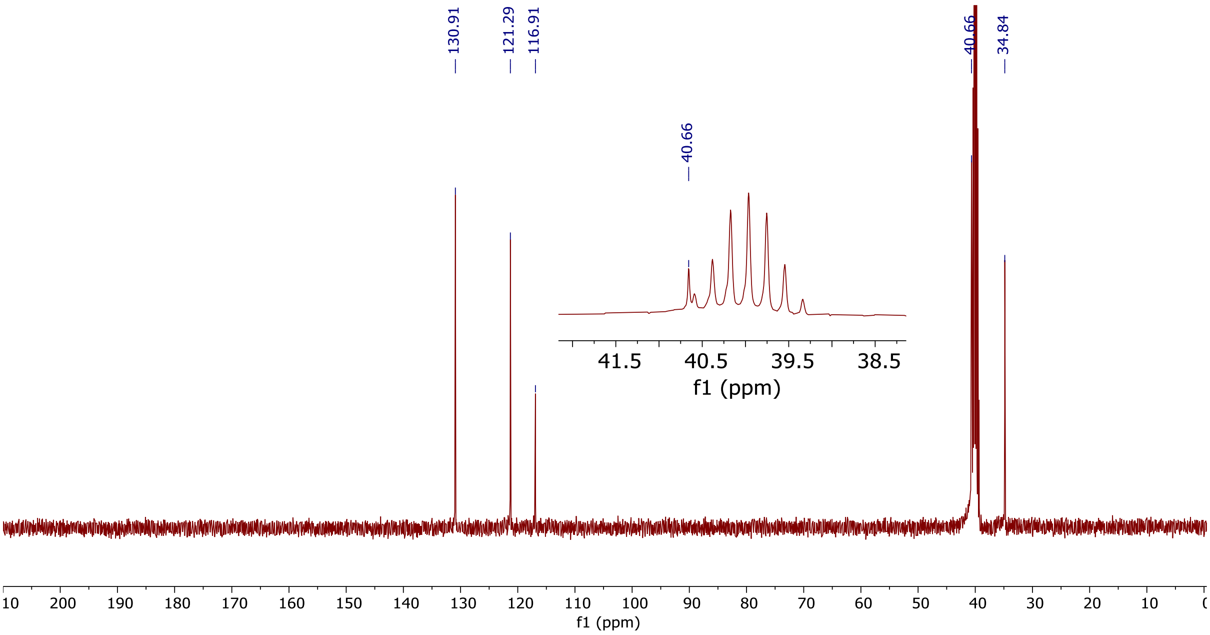

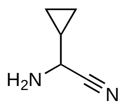

^{1}H NMR spectrum of **60**.

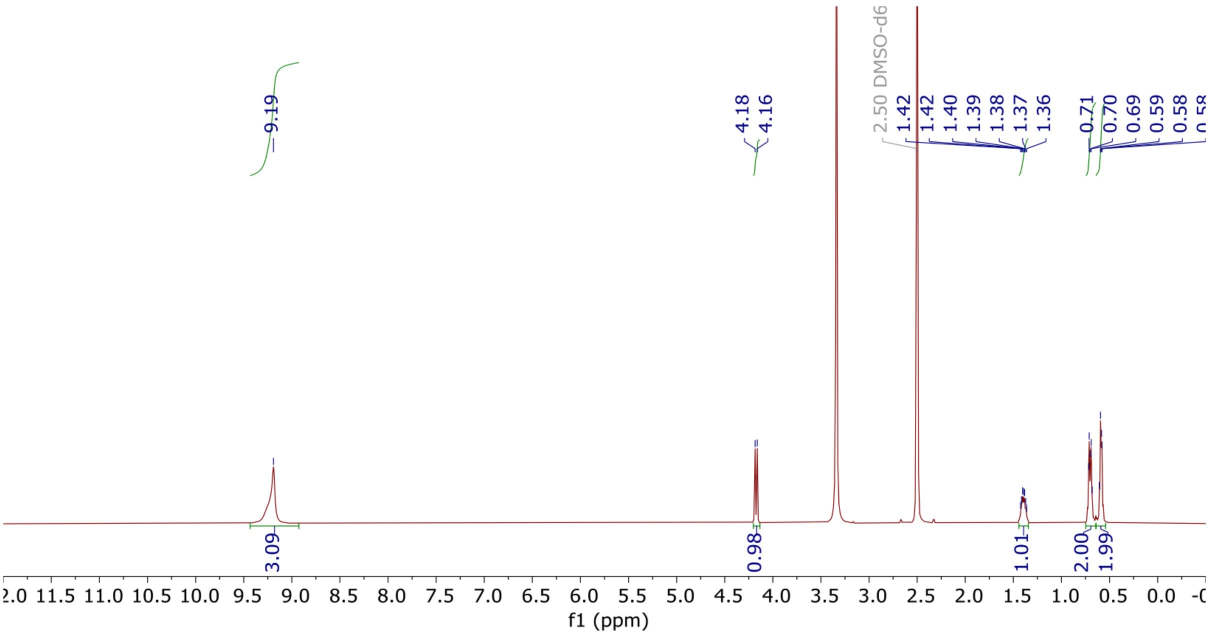

^{13}C NMR spectrum of **60**.

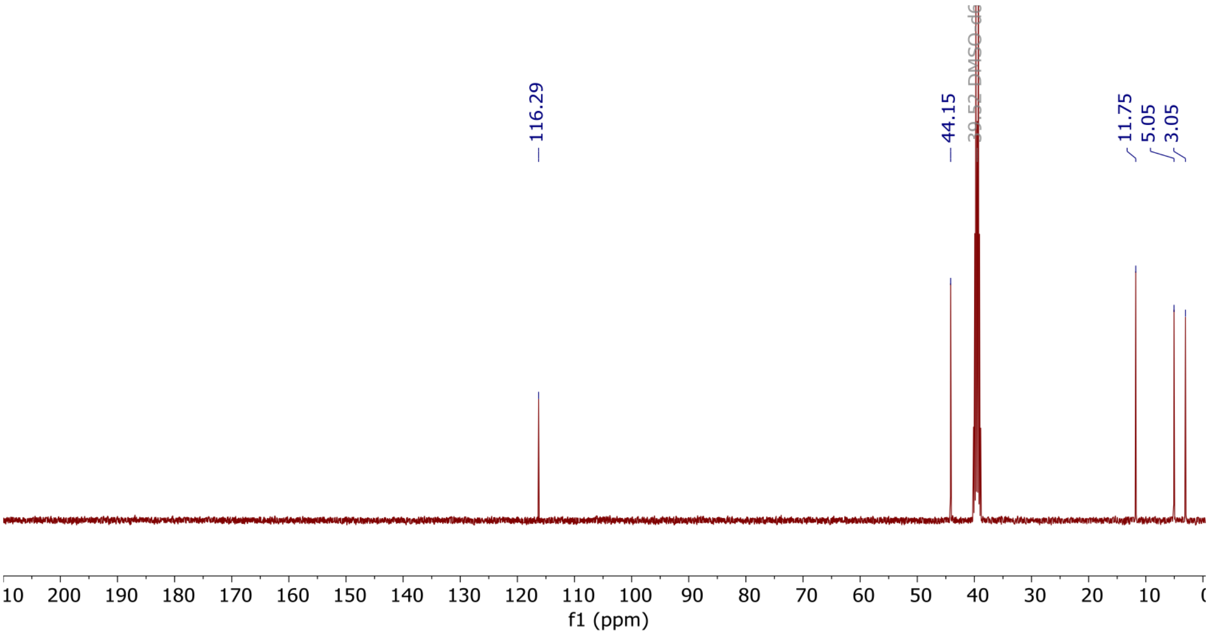

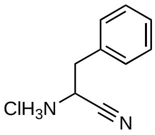

^{1}H NMR spectrum of **55**.

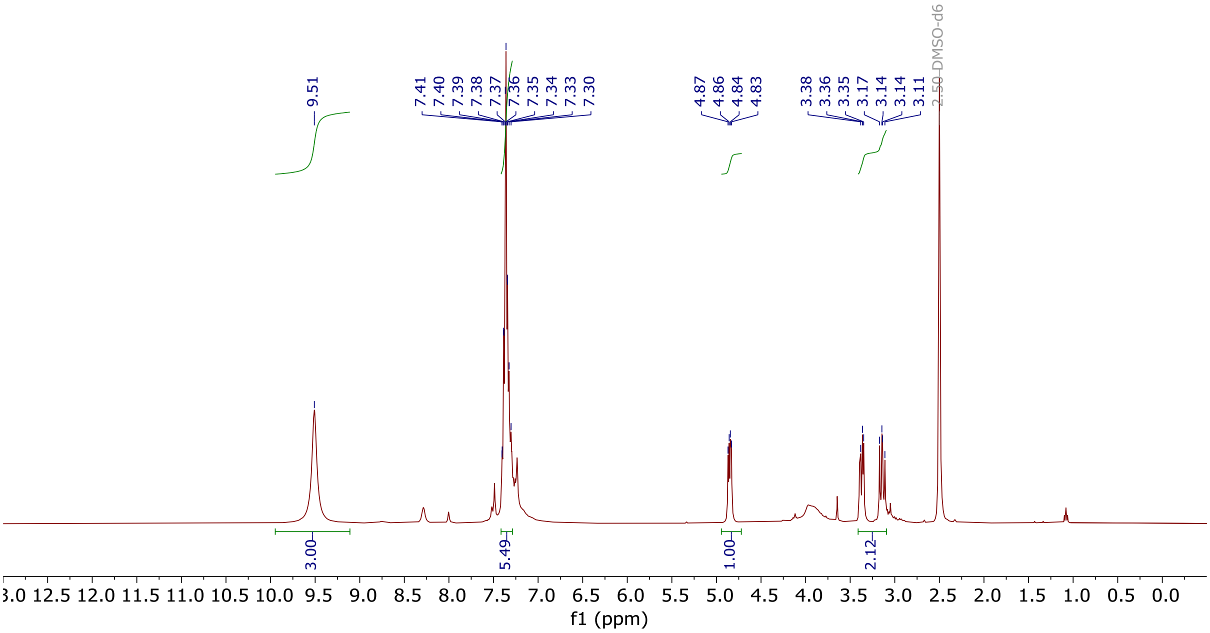

^{13}C NMR spectrum of **55**.

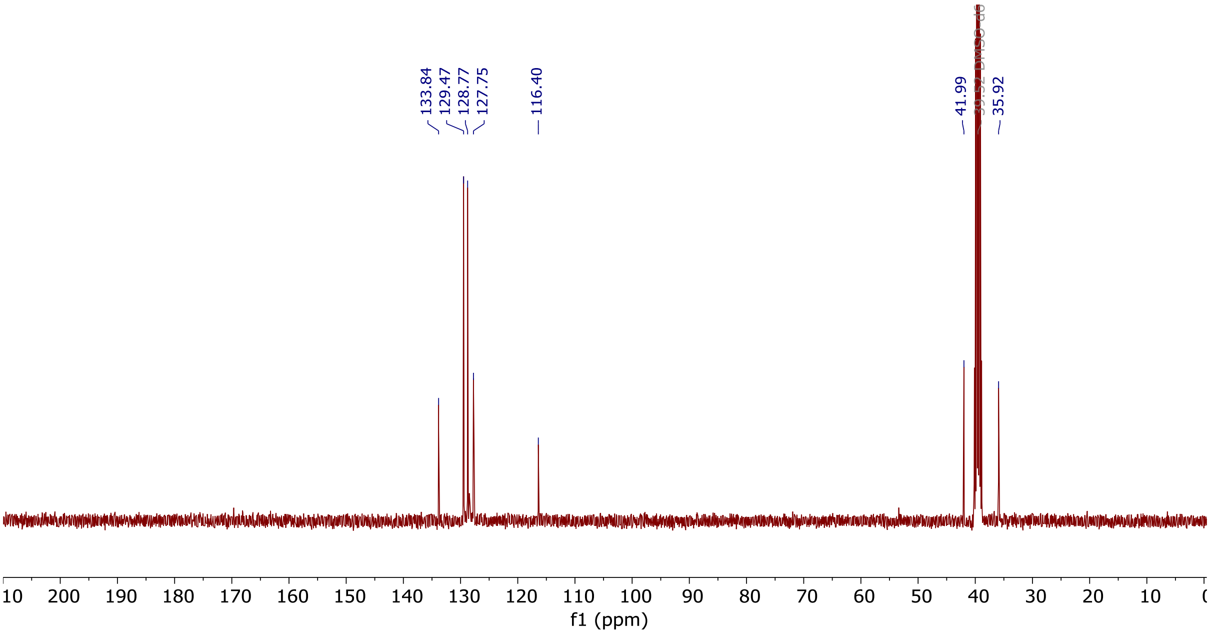

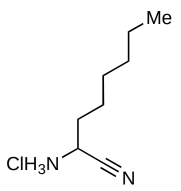

^{1}H NMR spectrum of **61**.

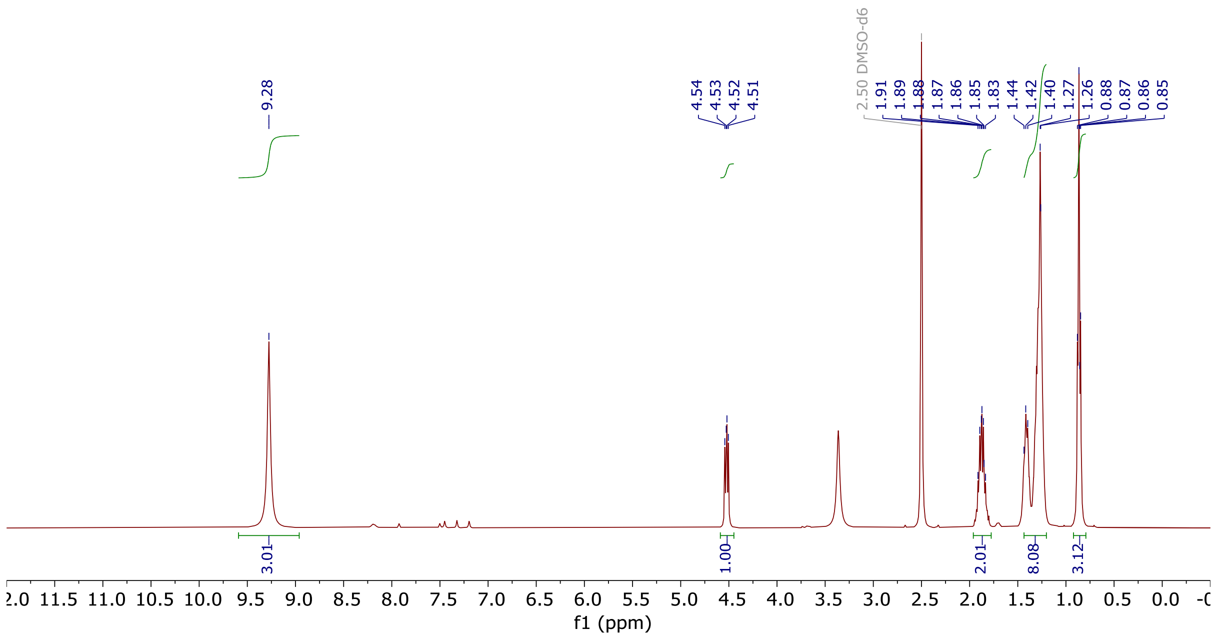

^{13}C NMR spectrum of **61**.

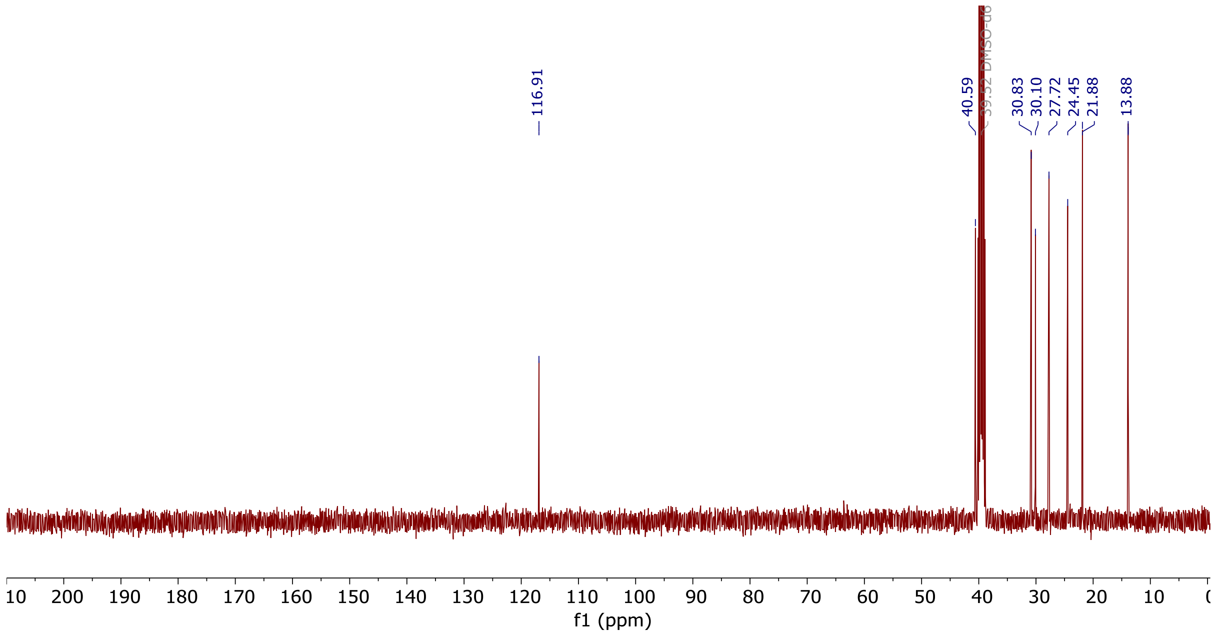

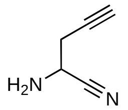

^{1}H NMR spectrum of **58**.

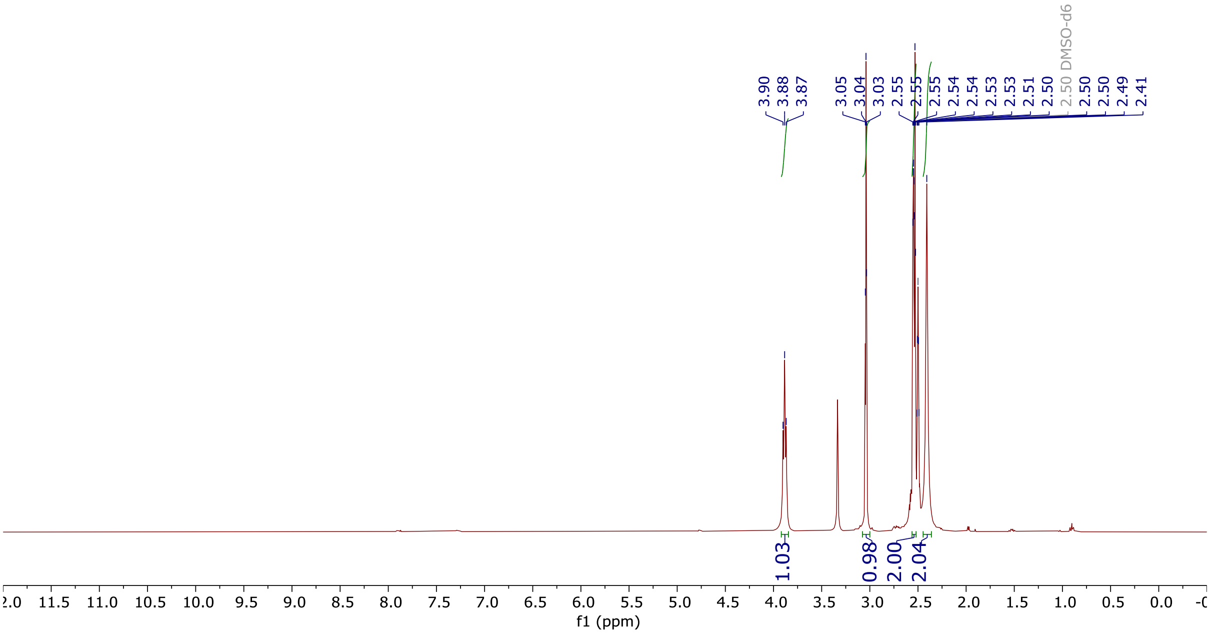

^{13}C NMR spectrum of **58**.

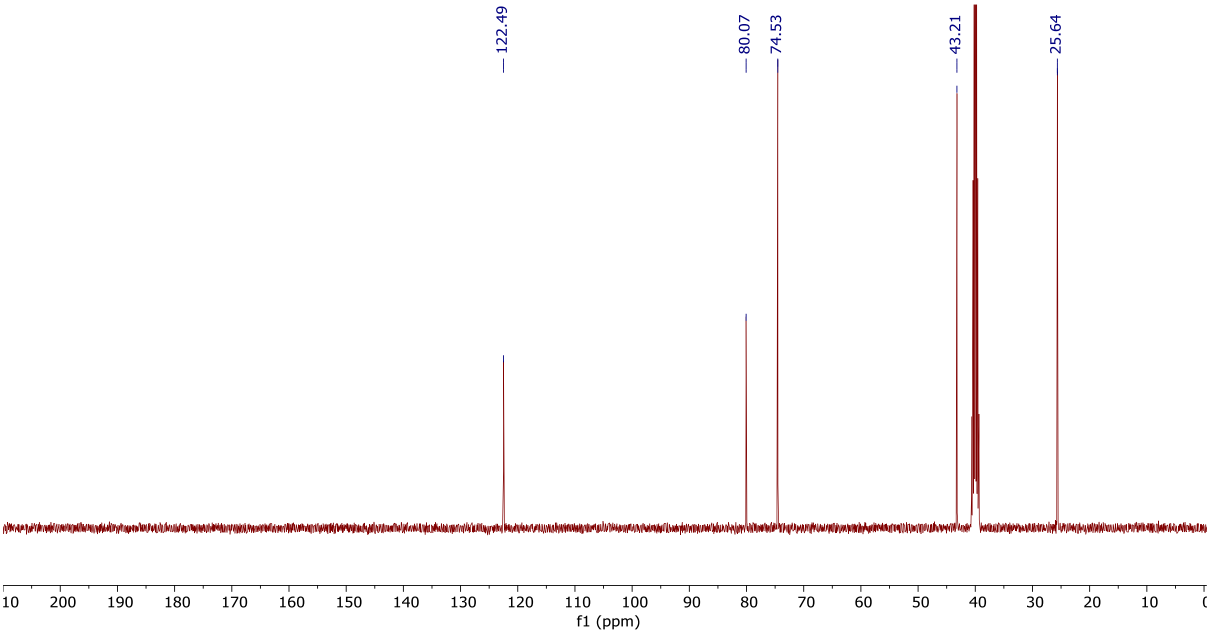

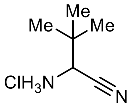

^{1}H NMR spectrum of **57**.

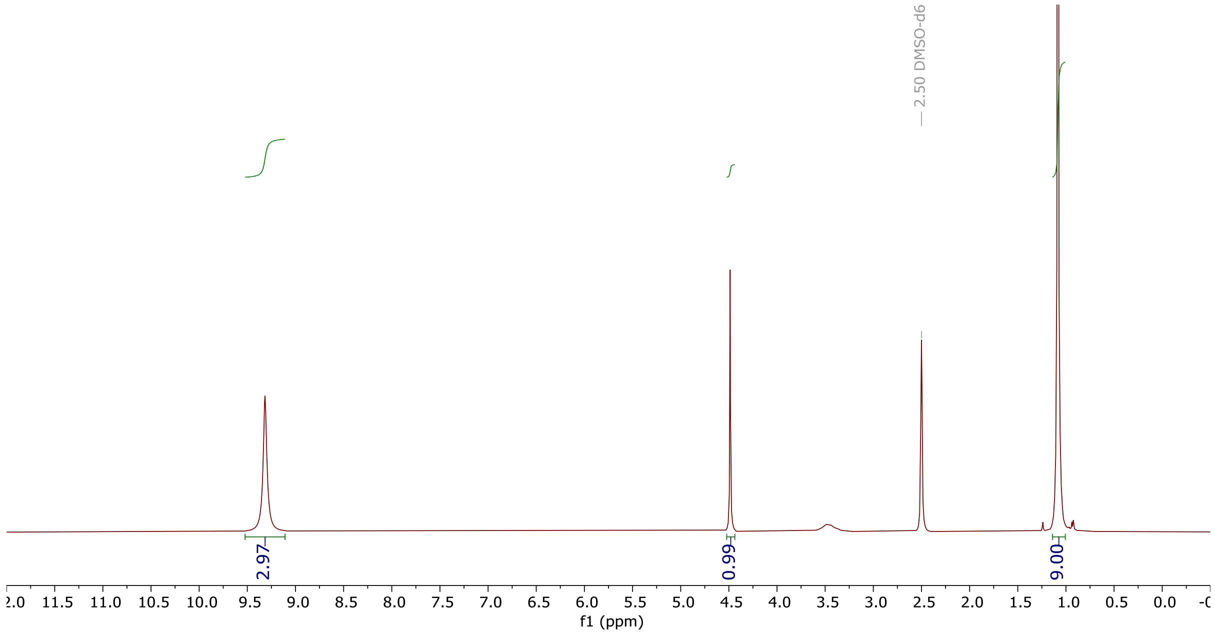

^{13}C NMR spectrum of **57**.

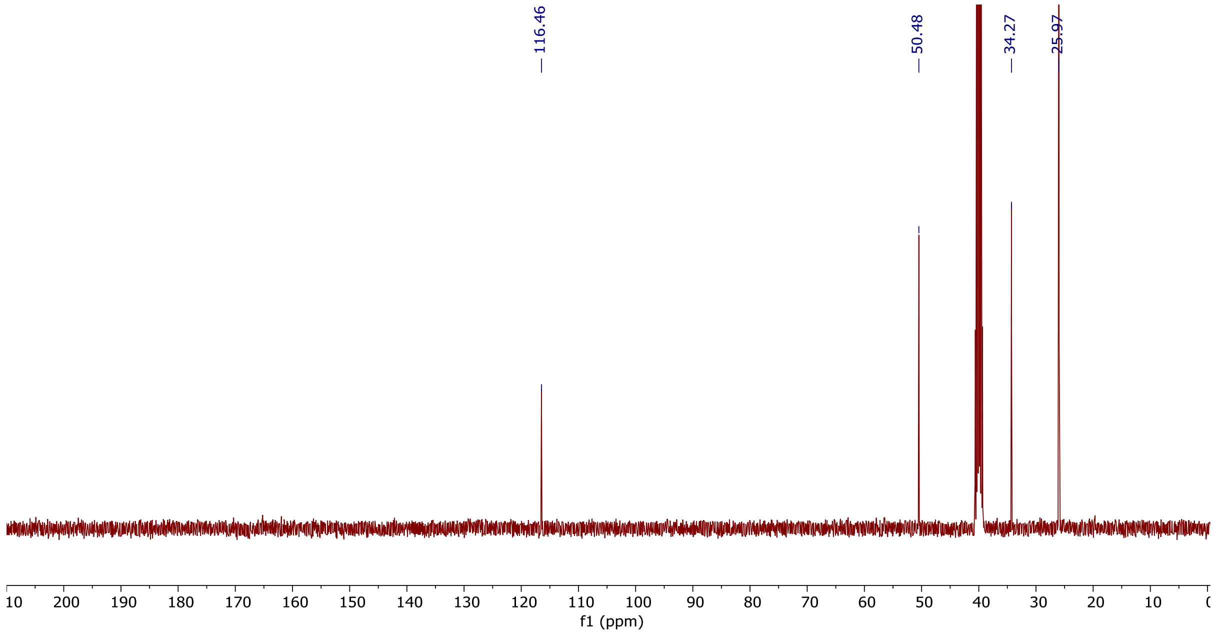

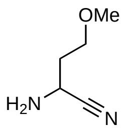

^{1}H NMR spectrum of **59**.

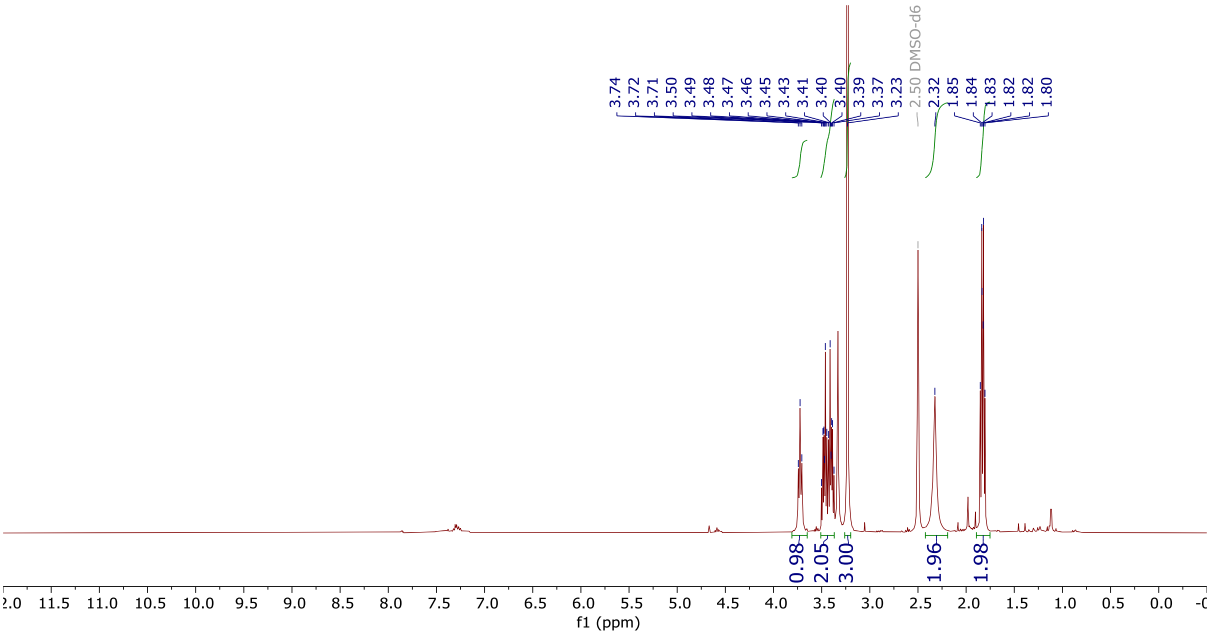

^{13}C NMR spectrum of **59**.

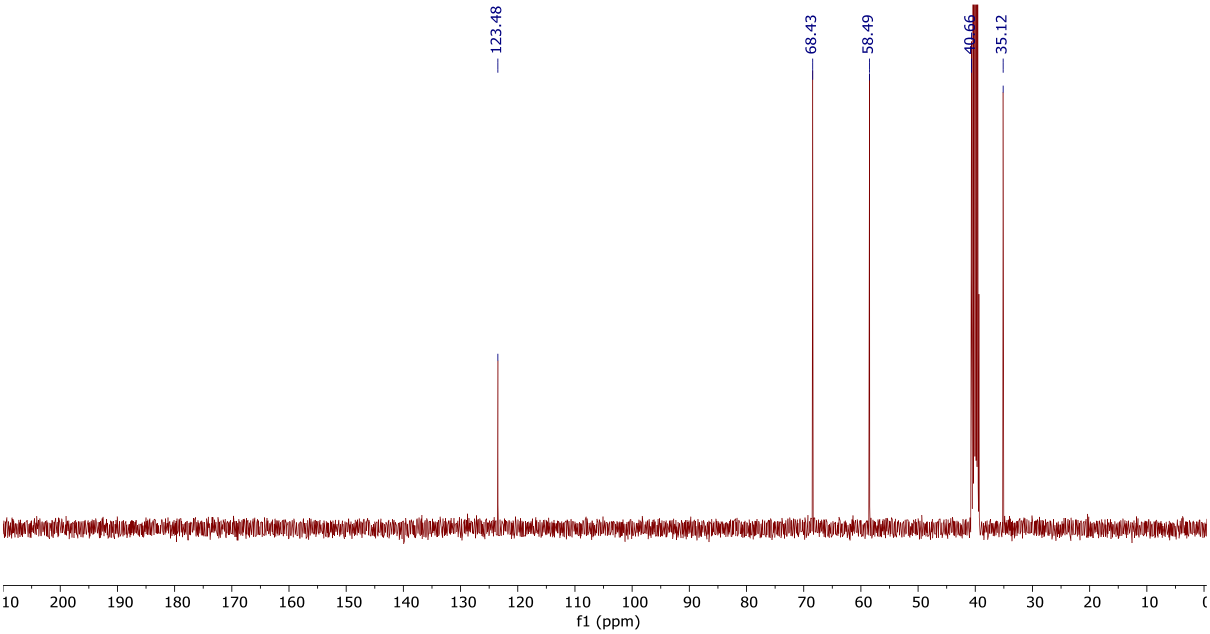

Supplementary reference

[1] J. Sambrook, *Molecular cloning: a laboratory manual*, 3rd edition, Cold Spring Harbor Laboratory Press, **2001**.

[2] E. J. Craven, J. Latham, S. A. Shepherd, I. Khan, A. Diaz-Rodriguez, M. F. Greaney, J. Micklefield, *Nat. Catal.* **2021**, *4*, 385-394.

[3] L. Bering, E. J. Craven, S. A. Sowerby Thomas, S. A. Shepherd, *Nat. Commun.* **2022**, *13*, 380.

[4] M. Winn, M. Rowlinson, F. Wang, L. Bering, D. Francis, C. Levy, J. Micklefield, *Nature* **2021**, *593*, 391-298.

[5] A. Saaret, B. Villiers, F. Stricher, M. Anissimova, M. Cadillon, R. Spiess, S. Hay, D. Leys, *Nat. Commu*n. **2021**, *12*, 5300.

[6] K. Plasch, V. Resch, J. Hitce, J. Popłoński, K. Faber, S. M. Glueck, *Adv. Synth. Catal.* **2017**, *359*, 959-965.

[7] G. Wu, Y. Deng, C. Wu, Y. Zhang, J. Wang, *Angew. Chem. Int. Ed.* **2014**, *53*, 10510-10514.

[8] T. Yoshimura, K. Tomohara, T. Kawabata, *J. Am. Chem. Soc.* **2013**, *135*, 7102-7105.

[9] S. Mai, C. Rao, M. Chen, J. Su, J. Du, Q. Song, *Chem. Commun.* **2017**, *53*, 10366-10369.

[10] B. Li, M. Berliner, R. Buzon, C. K. F. Chiu, S. T. Colgan, T. Kaneko, N. Keene, W. Kissel, T. Le, K. R. Leeman, B. Marquez, R. Morris, L. Newell, S. Wunderwald, M. Witt, J. Weaver, Z. Zhang, Z. Zhang, *J. Org. Chem.* **2006**, *71*, 9045-9050.

[11] F. M. F. Chen, N. L. Benoiton, *J. Org. Chem.* **1979**, *44*, 2299-2300.

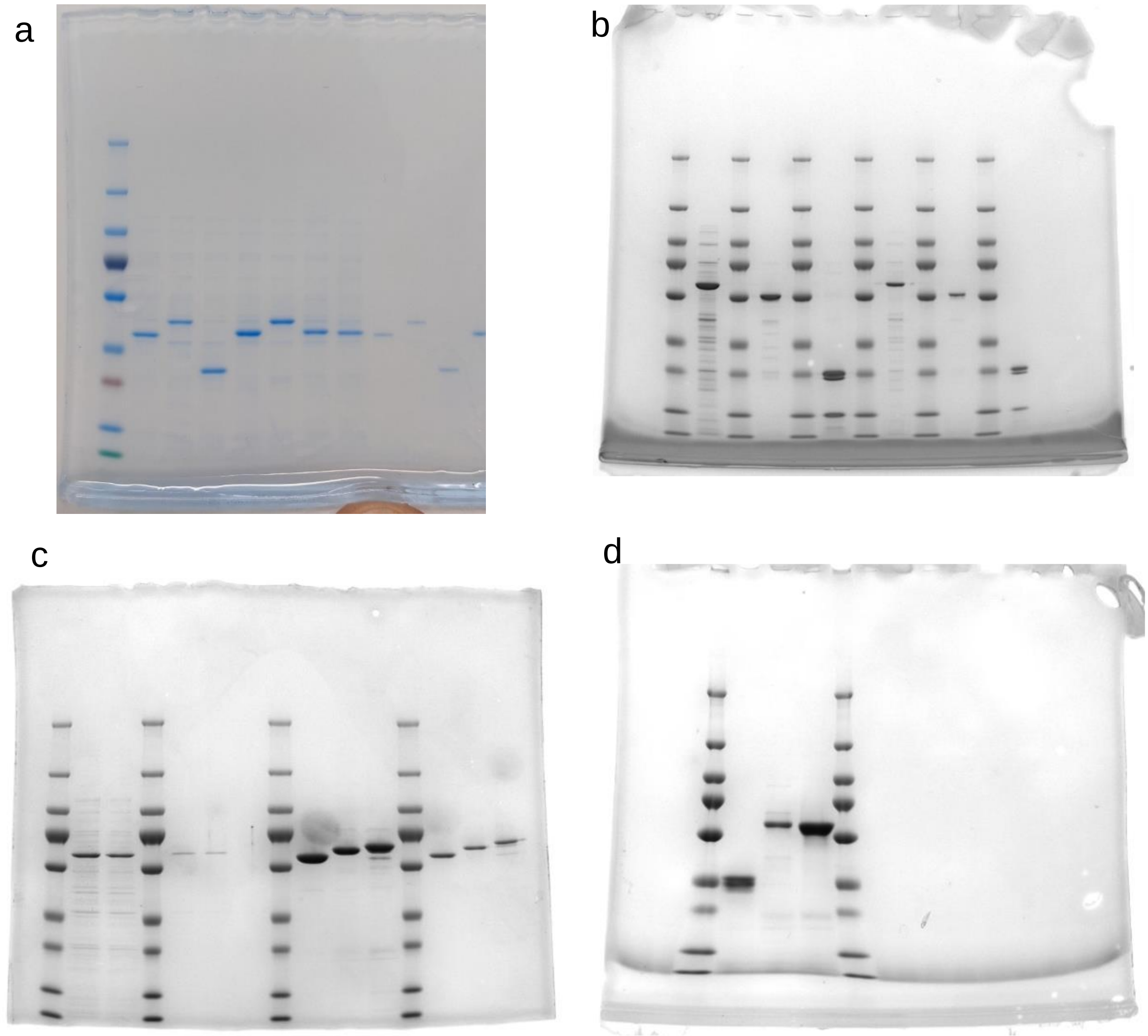

Figure S61. The original scans of SDS-PAGE presented in Figure S1-S3. a, The original scan of SDS-PAGE presented in Figure S1. **b,** The original scan of SDS-PAGE presented in Figure S2. **c & d,** The original scan of SDS-PAGE presented in Figure S3.
